# Supplementary material for: Astronomical calibration of the middle Cambrian in Baltica: global carbon cycle synchronization and climate dynamics
Source: Nat Commun. 2026 Mar 13;17:3912. doi: 10.1038/s41467-026-70651-5 (PMC13129069; doi:10.1038/s41467-026-70651-5)
Supplement: Supplementary file 1 — Supplementary Information [file 41467_2026_70651_MOESM1_ESM.pdf]

SUPPLEMENTARY INFORMATION

# Astronomical calibration of the middle Cambrian in Baltica: Global carbon cycle synchronization and climate dynamics

Valentin JAMART, Damien PAS, Linda A. HINNOV, Jorge E. SPANGENBERG, Thierry ADATTE, Arne T. NIELSEN, Niels H. SCHOVSBO, Nicolas THIBAULT, Michiel ARTS, Allison C. DALEY

***PART I: ADDITIONAL TEXT INFORMATIONS***

**1. MATERIALS AND METHODS**

**1.1. Stratigraphy**

*1.1.1. Geological setting*

**1.2. Geochemical proxies**

*1.2.1. X-ray fluorescence (XRF) core scanning*

*1.2.2. ICP-MS*

**1.3. Cyclostratigraphy and time series analysis**

*1.3.1. Preparation of the data*

*1.3.2. Z-score normalization*

**2. RESULTS & DISCUSSION**

**2.1. Stratigraphy, geochemistry and signal preservation**

*2.1.1. Fossil identification*

*2.1.2. Preservation of the carbon isotope signal*

**2.2. Geochemical events and global correlations**

*2.2.1. Identification of the DICE event in Baltica*

**2.3. Time series analysis**

*2.3.1. Proxy selection*

*2.3.2. Cyclicity in the signal*

*2.3.3.* 173 kyr identification

*2.3.3.1. MTM and EHA*

*2.3.3.2. Zhao et al (2022)'s supplementary data*

*2.3.3.3. Ratio of frequencies*

*2.3.3.4. ASM and average sedimentation rate*

*2.3.4. Age-depth model*

*2.3.5. Hilbert modulation analysis*

**2.4. Astronomically-tuned age model for the Miaolingian Series**

*2.4.1. Duration estimates of Biozones*

*2.4.2. Duration estimates of the Miaolingian Series, stages, and events*

*2.4.2.1. Duration of Series and stages*
*2.4.2.2. DICE event*
*2.4.2.3. Other events*

Supplementary Figures S1-S12
SUPPLEMENTARY REFERENCES

***PART II: DETAILED SPECTRAL ANALYSIS' PROTOCOL***

---

## ***PART I: ADDITIONAL TEXT INFORMATIONS***

# 1. MATERIALS AND METHODS

## 1.1. Stratigraphy

### *1.1.1. Geological setting*

The Albjära-1 core was drilled north of the village of Svalöv in Scania, southern Sweden (Fig. 1C), and is curated at the University of Copenhagen in Denmark. During the Cambrian Period, Scania was located on the external margin (deepest environment) of the Scandinavian Shelf extended northward from northern Poland to Finnmark in northern Norway and eastward from the Oslo area in Norway to the St. Petersburg area in western Russia[1-3].

In the Albjära-1 core, the ASF extends from the Wuliuan *Ptychagnostus gibbus* (agnostoid) to the Tremadocian *Bryograptus kjerulfi* (graptolite) Biozone[2,4]. The ASF has been central to numerous studies in paleontology[e.g.,5,6-9], lithostratigraphy[e.g.,1,10-13], geochemistry[e.g.,5,14,15-17], and in cyclostratigraphy[2,18]. These studies have contributed to the establishment of a robust biostratigraphic framework for Baltica and enabled intercontinental correlations[1,10-13,19,20]. The ASF overlies a significant unconformity (Fig. 2) resulting from the Hawke Bay Event *sensu lato* regression and the major hiatuses associated with it, which significantly affected the geological record of the Scandinavian Shelf[1,4,10]. The Hawke Bay Event *sensu lato* is a major, epeirogenically-induced regressive sea-level event located at the Stage 4–Wuliuan boundary in the sedimentary record of Baltica and several other localities bordering the Iapetus Ocean[10,21,22].

Directly underlying the ASF is an approximately 13 cm-thick interval consisting of light gray, glauconitic and partly phosphoritic greywacke that locally displays macropyrite mineralization (Fig. S1). These sedimentary features suggest a phase of renewed drowning of the shelf[1]. A comparison of the lithological succession with the Andrarum-1 and Gislövshammar boreholes in Scania, as described by Westergård[11], indicates that the greywacke observed in the Albjära-1 core likely belongs to the Age 3-4 *Holmia kjerulfi* Biozone. According to Nielsen and Schovsbo[1] this corresponds to the Gislöv Formation. The limited extent of the Gislöv Formation in the Albjära-1 core is associated with the hiatuses and erosion caused by the Hawke Bay Event *sensu lato*. We interpret this 13 cm-thick sequence boundary as the record of the Hawke Bay Event *sensu lato* in the core (see also Nielsen and Schovsbo[1]).

The Hardeberga Formation (Fig. 2) is ~ 5 m thick and is characterized by light gray sandstones with evaporite pseudomorphs in its upper part. This formation extends from the Age 2 *Platysolenites antiquissimus* (Small Shelly Fossil) to the lower part of the Age 3 *Schmidtiellus mickwitzi* (trilobite) Biozone[1,20].

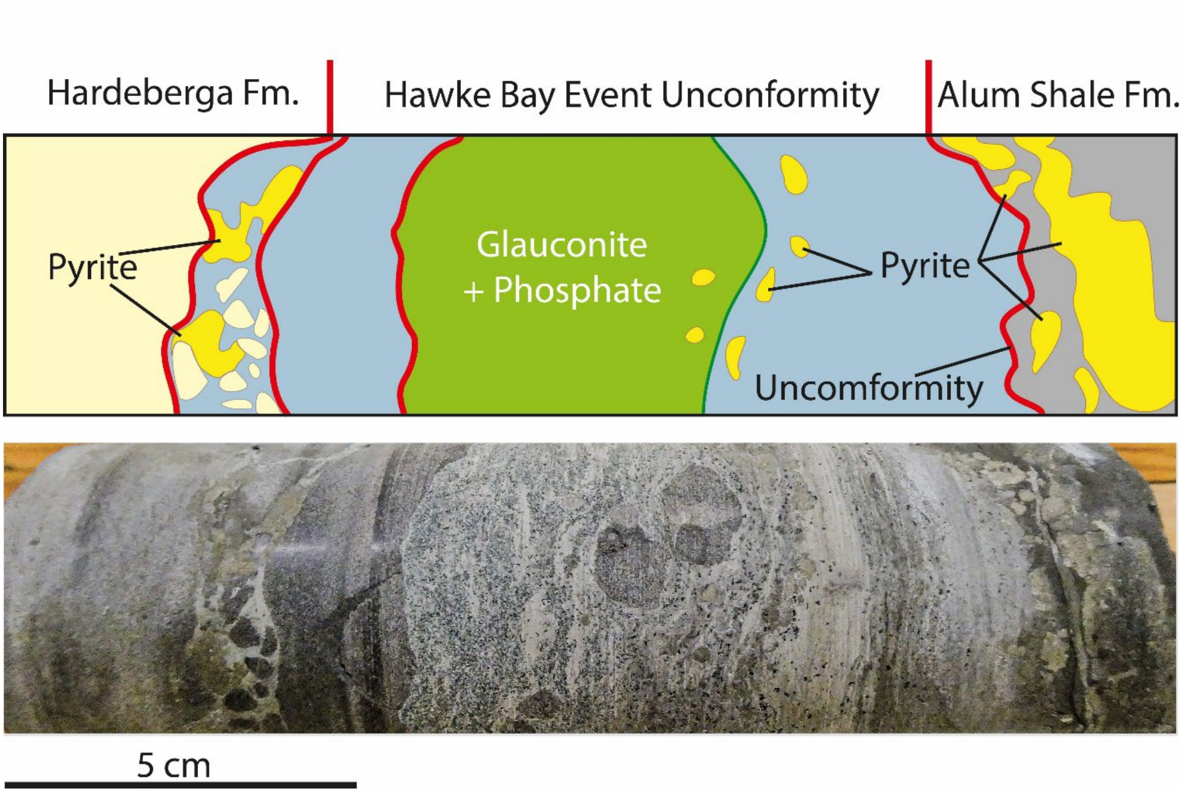


**Figure S1**: Close-up view of the Hawke Bay unconformity *sensu lato*.

## *1.2.* Geochemical proxies

### *1.2.1.* *X-ray fluorescence (XRF) core scanning*

The Albjära-1 Core Lower part (ACL) was scanned using an Itrax core scanner at the University of Stockholm in Sweden. The Itrax core scanner parameters are as follows: Molybdenum tube as X-ray source, 40 kV voltage, 10 mA current, 1000 microns (1mm) step size and XRF exposure time of 20 seconds. Prior to the scanning, a chlorine-rich dough was applied to the cracks and gaps of the core to easily identify and remove them in the resulting XRF dataset. The result in a total measurement of 28 chemical elements (e.g., Al, Ti, Si, K, Zr, Ca) in count per second (cps).

To calibrate the scanner output (cps), 50 pressed powder pellets, with a known elemental concentration, as measured by ICP-MS, were scanned under identical parameters, except for a reduced step size of 500 µm. Only those standard pellets with elemental trends consistent with the corresponding core intervals were used for the final calibration.

### *1.2.2.* *ICP-MS*

The Ultratrace 4 – Near Total Digestion – ICP-MS method protocol, as presented by Activation Laboratories Ltd., is as follows: A 0.25 g sample is digested with four acids beginning with hydrofluoric, followed by a mixture of nitric and perchloric acids, heated using precise

programmer-controlled heating in several ramping and holding cycles which takes the samples to dryness. After dryness is attained, samples are brought back into solution using hydrochloric and nitric acids. This digestion may not be completely total if resistant minerals are present. As, Sb and Cr may be partially volatilized. Digested samples are diluted and analyzed by an ICP-MS. One blank is run for every 40 samples. In-house control is run every 20 samples. Digested standards are run every 80 samples. After every 15 samples, a digestion duplicate is analyzed. Instrument is recalibrated every 80 samples. An in-lab standard (traceable to certified reference materials) or certified reference materials are used for quality control.

### 1.3. Cyclostratigraphy and time series analysis

#### *1.3.1. Preparation of the data*

Before conducting the time series analysis, we compared the XRF data of the detrital elements and Ca from the overlapping portion of the dataset of Zhao, et al.[2] and our own dataset. This allowed us to identify a distinctive 5 cm calcite veins framework at 212.22 m, which was clearly visible in the XRF core scanning data and was used as a correlative marker. This alignment serves as the reference point for constructing our depth model.

To anchor our astronomical time scale (ATS), we used the astronomical age of 499.9 ± 0.9 Ma located at a depth of 212.49 m from the calibrated ATS of Zhao, et al.[2].

To ensure consistency with downhole measurements, we compared our depth-referenced dataset with the in-well gamma-ray (GR) and resistivity logs, which correspond to the *in situ* depth recorded during the drilling.

This comparison revealed a systematic offset of + 0.18 m (= "Corrected Depth" in Fig. S5), indicating that corrections were necessary to align key lithological features, specifically, prominent limestone horizons, with those occurring in the GR and resistivity logs, in both the Zhao, et al.[2] depths and our measurements. This alignment also helped identify potential anomalies in detrital input trends.

Although XRF core scanning was conducted on the interval between 232.62 and 237.4 m, this portion was not studied due to the presence of a major unconformity between 232.616 and 232.745 m. This unconformity interrupts the stratigraphic continuity and prevents reliable time-series interpretation.

After anchoring both datasets, we prepared the ACL data as follows:

**1)** The thickness of the anthraconite concretions (> 2mm, < 25% Si, and > 15% Ca) was reduced to 20%, in accordance with the methods of Sørensen, et al.[18] and Zhao, et al.[2]. The thickness of the syndepositional limestones (*cf*. Andrarum and Exsulans limestones, Fig. 2) was not reduced. The resulting depths are anchored in direct continuity with the adjusted depths reported in Zhao, et al.[2]. These adjusted depths are referred to as depth [m] AD for the remainder of this work. The anchoring between the ACL and the ACU, which is located at 212.22 m, corresponds to 73.452 m AD. Therefore, the ACU ranges from 0 to 73.72 m AD, and the ACL ranges from 71.41 to 98.395 m AD.

2) To minimize biases related to the lithology in time series analysis, caused by significant differences in detrital element concentrations between contrasting lithologies (e.g., shale versus limestone), the entire dataset was divided into 24 intervals (see Supplementary datasets). These intervals correspond to major lithological shifts that strongly influence the detrital signal and affect normalization. We then applied a Z-score normalization within each interval (see Section 1.3.2 Z-score normalization for more details).
3) To support the detection and interpretation of major Milankovitch cycles, we recalculated the expected duration of short and long eccentricity, obliquity and precession cycles by using the astronomical model of Wu, et al.[27] for the 497-505 Ma interval (Supplementary datasets). We used a precession constant (k) of 59.78 arcsec/year, as presented by Wu, et al.[23], and derived from the Alum Shale Formation, based on data from Sørensen, et al.[18].

### *1.3.2. Z-score normalization*

The Alum Shale Formation (ASF) recorded in both Zhao, et al.[2] and the dataset of this study, is characterized by black shale deposits disrupted by a few limestone horizons. This results in high variability in the detrital elements, such as Ti values ranging from 0.3 to 0.8 % in black shales and from 0.1 to 0.2 % in limestones. If this significant range of variability between lithologies, is not addressed, it will impact the results of the time series analysis.

One solution to address the limestone issue is to remove the data and/or create gaps in the dataset before conducting time series analysis on discrete intervals separated by these gaps. However, we believe that this solution could result in the addition of temporal gaps, as the thickness corresponding to the limestone horizons will not be taken into account.

Another solution is to integrate the limestone horizons. To accomplish this, we applied a Z-score normalization to the dataset.

Z-score normalization is the most frequently used standardization function in signal processing when high variability is observed[e.g.,24-26]. The Z-score function can be formulated as follows:

$$\text{Z-score} = \frac{X - \mu}{\sigma}$$

Where X is the measured data, μ is the mean value of the selected interval including X, and σ is the standard deviation of the selected interval including X.

First, we divided the 62.111-93.610 m AD composite dataset (ACU + ACL) into 13 intervals corresponding to major shifts from shale to limestone lithologies. Then, we applied Z-score normalization. After normalization, we noticed that the variability within the limestone intervals was still significant. We subdivided these intervals into 11 smaller ones, resulting in a total of 24 intervals for the entire composite dataset. Prior performing the Z-score normalization, we removed a few data points located in transition zones between two significant lithological changes. The Z-score-normalized dataset with the Z-score function applied to Al, Si, Ti, K, Zr, and Ca is available in Supplementary datasets.

## 2. RESULTS & DISCUSSION

### *2.1.* Stratigraphy, geochemistry and signal preservation

#### *2.1.1. Fossil identification*

The combination of literature and the newly identified cranidia and pygidia of agnostid and trilobite species along the core, which are representative of the Drumian Stage of Baltica, enabled us to precisely determine the Wuliuan–Drumian and Drumian–Guzhangian boundaries in the ACL (Fig. 2, S2).

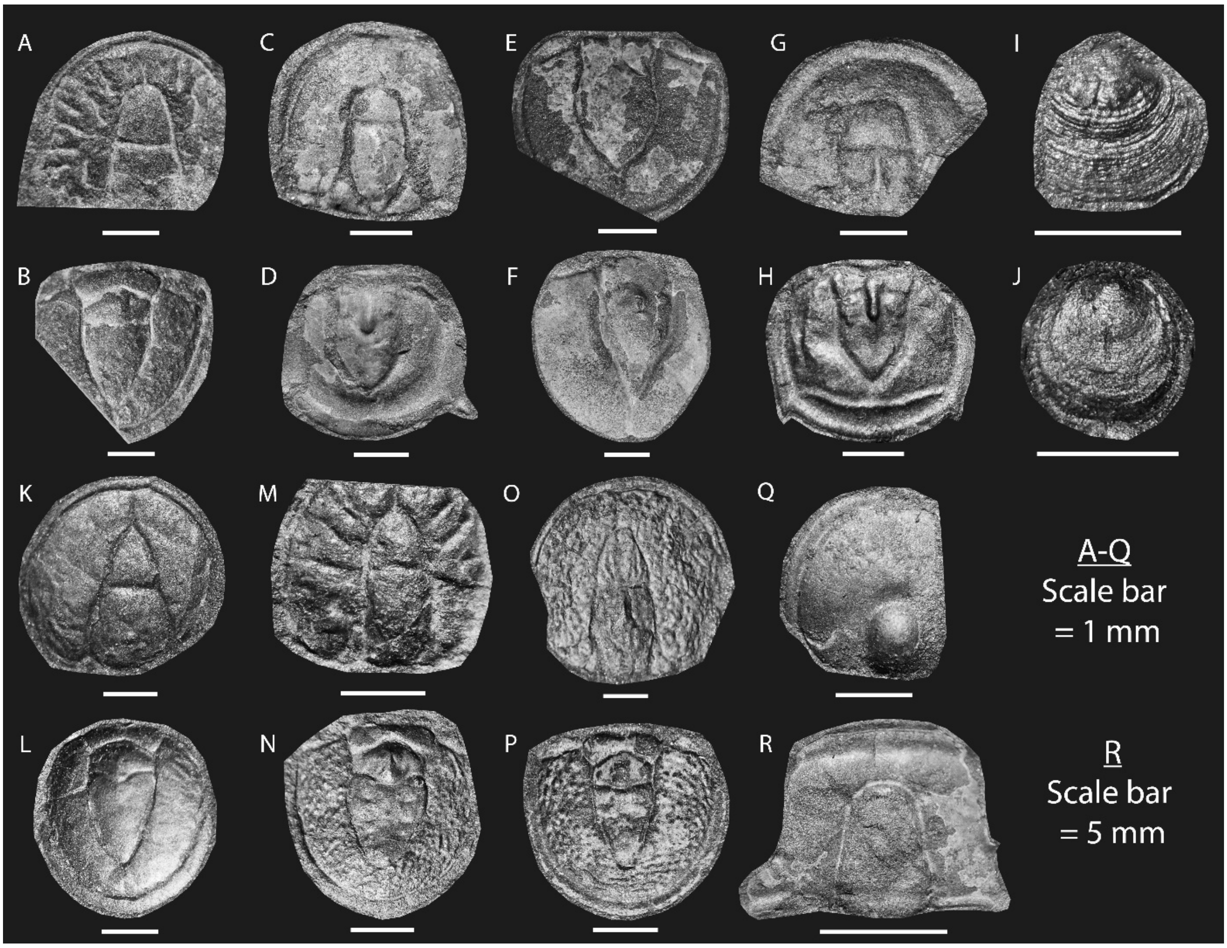


**Figure S2**: Agnostoids, trilobite and brachiopods from the Albjära-1 core. A-B – *Acidusus atavus* = *Ptychagnostus atavus*[27]. Specimens A and B are from 227.815 m. C-D – *Acadagnostus ferox* (Tullberg, 1880)[27]. Specimen C is from 219.69 m, specimen D is from 217.12 m. E – *Svenax pusillus*? (Tullberg, 1880)[27]. Specimen E is from 221.19 m. F – *Doryagnostus incertus* (Brøgger, 1878)[28]. Specimen F is from 219.75 m. G-H – *Diplagnostus planicauda bilobatus* Kobayashi (1939)[29]. Specimen G is from 228.64 m, specimen H is from 217.12 m. I-J – Brachiopods indet. Specimens I and J are from 215.09 m. K-L – *Lejopyge lundgreni* (Tullberg, 1880)[27]. Specimen K is from 216.84 m, specimen L is from 215.09 m. M-N – *Ptychagnostus affinis* (Brøgger, 1878)[28]. Specimen M and N are from 224.73 m. O-P – *Ptychagnostus punctuosus* (Angelin, 1851)[30]. Specimen O is from 220.70 m, specimen P is from 219.36 m. Q – *Hypagostus* sp. (*mammillatus* (Brøgger, 1878) or *parvifrons* (Linnarsson, 1869))[28,31]. Specimen Q is from 226.95 m. R – *Parasolenopleura linnarssoni* (Brøgger, 1878)[28]. Specimen R is from 225.05 m.

Regarding the base of the *L. laevigata* Biozone, in Baltica, it is inferred that this biozone coincides with the base of the local *Solenopleura brachymetopa* Biozone, which corresponds

to the base of the Andrarum limestone in Scania[4,8,13,32]. The base of the Andrarum limestone in the ACL is located at 215.07 m (= 76.305 m AD).

Concerning the *P. atavus* Biozone, we identified the lowest occurrence of *P. atavus* at 228.7 m (= 89.695 m AD). In Baltica and Siberia, however, the lower part of the *P. atavus* Biozone coincides with the *Tomagnostus fissus* Biozone[4,20,33]. *T. fissus* is identified in the Albjära-1 core at ~ 229.3 m (= 90.295 m AD) based on correlation with Lauridsen[34], which allowed us to delineate the Wuliuan–Drumian boundary in the ACL

### *2.1.2. Preservation of the carbon isotope signal*

The $\delta^{13}C_{org}$ values recorded in the ACL fall within the expected range of Cambrian values, suggesting a limited metamorphic imprint on the organic carbon isotopes[35,36] (Fig. S3A).

To evaluate primary organic signal preservation, one must examine the correlation between the $\delta^{13}C_{org}$ and TOC values[37-39]. The absence of a statistically significant and positive trend ($R^2$ = 0.08, Fig. S3B) between $\delta^{13}C_{org}$ and TOC values suggests that the $\delta^{13}C_{org}$ signal is preserved.

The analysis of Rock-Eval-derived HI/OI ratio and $T_{max}$ can be conducted to estimate the thermal maturity of the organic carbon data[40]. According to Peters[40] the interpretation of T max is valid only if $S_2$ peak values are above a threshold value of 0.2. In ACL, 73 of the $S_2$ peak values, obtained through Rock-Eval pyrolysis exceed the 0.2 threshold (see Supplementary datasets). However, the pyrograms resulting from the Rock-Eval analysis show that the $S_2$ peaks are difficult to identify, resulting in extremely low HI values. Consequently, the $T_{max}$ values cannot be used with sufficient confidence and will not be discussed. Nonetheless, based on the work of Buchardt and Lewan[41], Sanei, et al.[42] and Zhao, et al.[43], the ASF have experienced a $T_{max}$ around 400°C, which is indicative of late diagenesis to low-grade metamorphic conditions. The average reflectance of vitrinite-like particles is 2.1% Ro – dry gas window – measured on five samples from the Tremadocian to Furongian interval[44]. Regarding the HI/OI ratio, most of the data exhibiting low HI and OI values are located in the lower left corner of a modified pseudo-van Krevelen diagram, confirming that the organic matter (OM) of the rocks is mature to overmature[40].

The clay mineral assemblage in the <2 µm size fraction along the ACL (Fig. S3C) consists primarily of illite (38–68%), chlorite (24–48%), and illite–smectite mixed-layer phases (IS) containing 10–21% smectite layers. Illite–smectite mixed layers are present in all samples, and their smectite content was estimated using the method described by Moore and Reynolds[45]. The average smectite content of the IS fraction is approximately 16%. These mixed-layer clays represent intermediate transformation phases in the progressive conversion of smectite to illite, a process known as illitization, and are indicative of deep diagenetic to anchizone conditions. The degree of illite crystallinity, expressed through the Kübler Index[46], calculated on the <2 µm fraction, ranges from 0.32 to 0.40 Δ°2θ, suggesting deep diagenesis extending into the base of the anchizone. These values correspond to paleotemperatures that potentially reached but not exceeded ~300 °C. This implies a burial depth of approximately 4–6 km, assuming a geothermal gradient of 25–30 °C/km (see also Schovsbo, et al.[47] for 1D thermal models of the area). The absence of kaolinite and smectite is consistent with these

interpretations. Geochemical data from Rock-Eval pyrolysis support these interpretation, revealing low Hydrogen Index (HI) values (2–60 mg HC/g TOC) and elevated Tmax values (>450 °C). These values are characteristic of advanced kerogen transformation and burial-related thermal maturation.

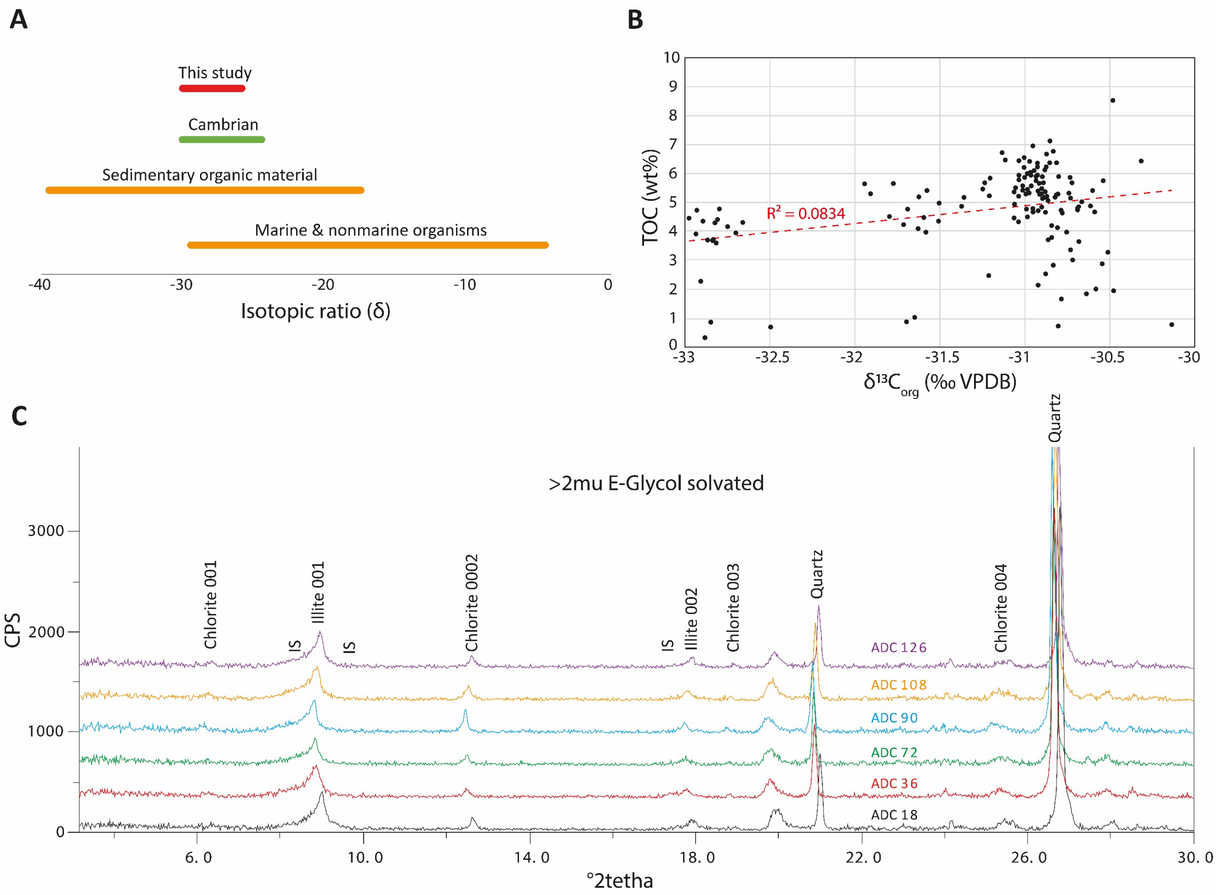


**Figure S3:** Signal preservation and organic matter maturation. A – $\delta^{13}C_{org}$ range in the different reservoirs, value range of the Cambrian Period, and this study; B – TOC versus $\delta^{13}C_{org}$ graph; C – XRD graphs and identification of the clay minerals.

## 2.2. Geochemical events and global correlations

### *2.2.1. Identification of the DICE event in Baltica*

The Drumian Carbon isotope Excursion (DICE) is defined as a negative carbon isotope excursion (CIE) situated approximately at the base of the Drumian Stage[20,48-50]. The peak of the excursion typically records $\delta^{13}C_{carb}$ values around -2 ‰, though values can range from -1 ‰ in West Gondwana[51] to -4 ‰ in Siberia and East Laurentia[52,53]. Based on chemo- and biostratigraphic data, the DICE roughly corresponds with the Wuliuan–Drumian stage boundary in South China[38,49,50,54,55], North China[54-58], Tarim[59,60], Laurentia[48,52,59-63], Siberia[52], North Gondwana[64,65], West Gondwana[51], and potentially East Gondwana[66,67].

The first appearance datum (FAD) of the agnostid *Ptychagnostus atavus* (= *Aciduscus atavus*) is the criterion used to define the Wuliuan–Drumian boundary[20,48]. In the Albjära-1 core, the lowest occurrence of *P. atavus* is identified at ~ 229.3 m (90.295 m AD), which allows

for the delineation of the Wuliuan–Drumian boundary in the ACL (see section 2.1.1. Fossils identification).

Due to the combination of high TOC and low carbonate content in the core, only $\delta^{13}C_{org}$ data could be obtained. However, $\delta^{13}C_{org}$ alone is typically insufficient for global chemostratigraphic correlation because $\delta^{13}C_{carb}$ is the standard reference for such comparisons. This makes direct correlation with global records more challenging. Nevertheless, several studies demonstrate that the global trends in both $\delta^{13}C_{carb}$ and $\delta^{13}C_{org}$ data fluctuate simultaneously in response to major perturbations in the carbon cycle, such as the DICE[38,51,58].

The precise location and identification of the DICE in Baltica is unclear in the literature and global chemostratigraphic correlations are difficult. To address this issue and identify the DICE in the ACL, we compared our $\delta^{13}C_{org}$ data with studies conducted in Baltica and surrounding areas by Ahlberg, et al.[5], Álvaro, et al.[14], Lehnert, et al.[15], Lundberg[16], and Shembilu and Azmy[53] (Fig. S4).

The data published by Ahlberg, et al.[5] for the Andrarum-3 drill core (Scania, Baltica) show some similarities, such as a plateau around - 30.2‰ in the *G. nathrosti* and *P. punctuosus* biozones. Then a slight positive shift up to – 30.0‰ is observed in the upper *P. atavus* Biozone before the values shift back to more negative values. The peak of the excursion is ~ - 30.5‰. However, it is difficult to precisely correlate our data with that presented in Ahlberg, et al.[5] because a "U"-shaped excursion is not observed in the Albjära-1 core, which hinders precise identification of the DICE. Additionally, the sampling rate is significantly different, ~1 m in Ahlberg, et al.[5] vs 15 cm in this study. Furthermore, based on the correlation with the stratigraphic column that Westergård[11] established for the Andrarum-1 borehole (located 200 m away from Andrarum-3 borehole), the excursion observed by Ahlberg, et al.[5] is located in the middle of the *P. atavus* Biozone. Comparing it with other sections in Baltica (Fig. S4) reveals that the weak amplitude (< 0.5‰) and different positioning (middle *P. atavus* vs *P. gibbus*) of the excursion in Ahlberg, et al.[5] suggest that it is unlikely to be the DICE.

In Álvaro, et al.[14], a negative excursion of 9‰ amplitude in $\delta^{13}C_{carb}$ is identified in the Brantevik section (Scania, Baltica) between the Exsulans and Forsemölla limestone beds (Fig. S4). However, Álvaro, et al.[14] elucidated that the few data points delineating this negative excursion are situated below and above unconformities/discontinuities. The combination of the limited data points and the presence of unconformities make it difficult to reliably identify this excursion as the DICE.

In Lehnert, et al.[15], a prominent $\delta^{13}C_{org}$ negative excursion is identified in the Almbacken drill core (Scania, Baltica) between the Exsulans and Forsemölla limestone beds, in a trilobite/agnostoid barren interval, with a peak value at ~ -33‰ (Fig. S4). This excursion is located in the *P. gibbus* Biozone and is followed by a positive shift of 1.5‰. Our $\delta^{13}C_{org}$ curve mirrors the unpublished curve of Lehnert, et al.[15].

In Lundberg[16], a prominent negative $\delta^{13}C_{org}$ excursion is identified in the Tomten-1 core (Västergötland, Baltica) below the Exsulans Limestone in the *P. gibbus* Biozone, peaking at ~ -

33.75‰ (Fig. S4). This excursion is followed by a 2‰ positive shift. Our $\delta^{13}C_{org}$ curve closely resembles the Lundberg[16] curve.

In Shembilu and Azmy[53], the observed trend in the $\delta^{13}C_{carb}$ record of the NF-02 core (Newfoundland, East Laurentia) is similar to that observed in the Albjära-1 core (Fig. S4). However, some differences can be noted: First, the Laurentian record is in $\delta^{13}C_{carb}$ and is therefore not directly comparable to the $\delta^{13}C_{org}$ presented in this study. Second, the sampling rate is significantly different: ~ 5m in Shembilu and Azmy[53] versus 15 cm in this study. Third, the base of the *P. atavus* Biozone is located on the upper shoulder of the excursion in Shembilu and Azmy[53], compared to this study where it is located close to the maximum of the CIE, immediately above the -33‰ negative plateau.

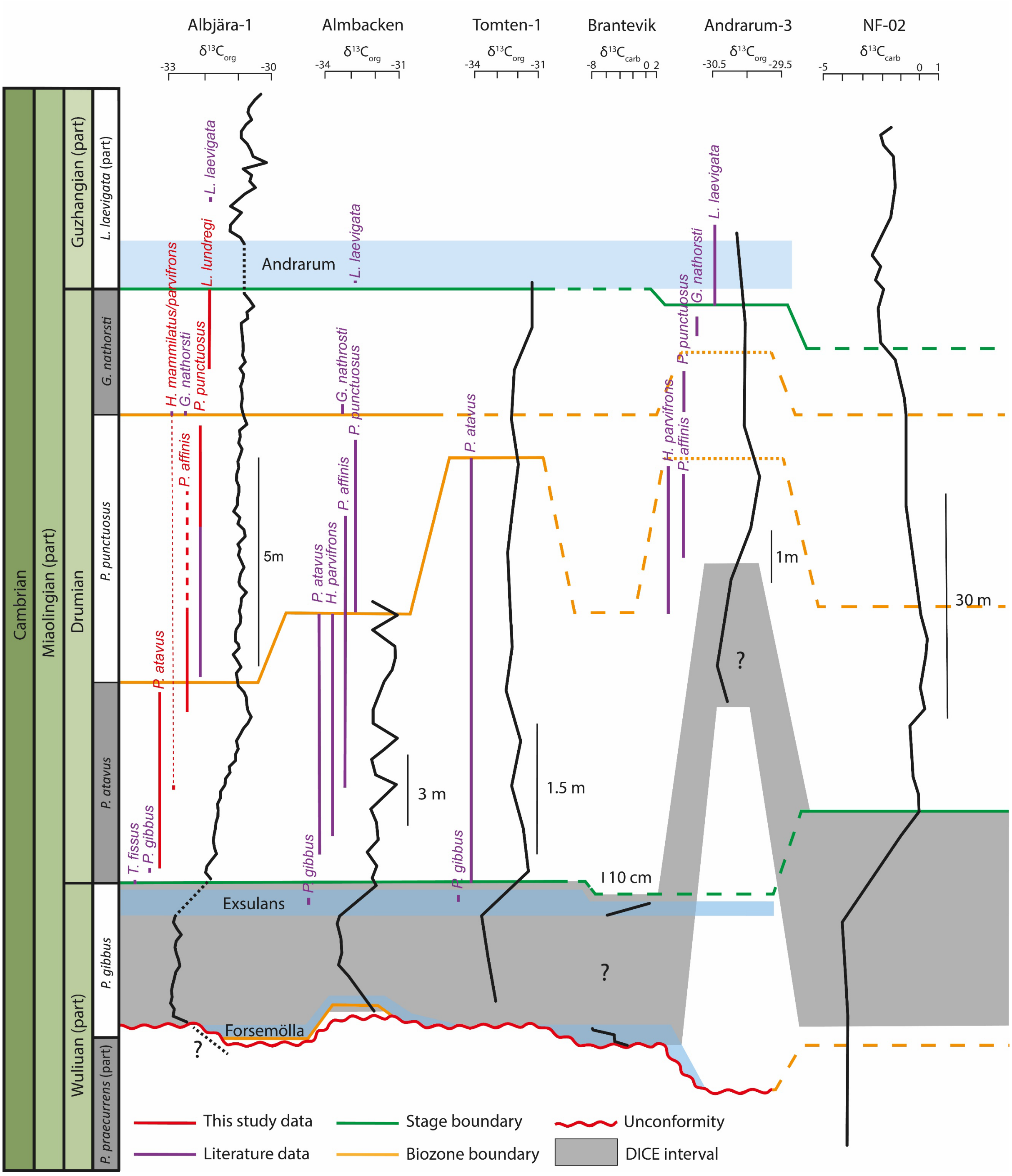

**Figure S4**: Comparison of the localities that recorded the DICE in the surrounding of the Baltic craton. Albjära-1 (this study); Almbacken[15]; Tomten-1[16]; Brantevik[14]; Andrarum-3[5]; NF-02[53]. For Baltica localities, the base of the Andrarum and Exsulans Limestones Beds have been used to tie and compare the carbon isotope curves.

## 2.3. Time series analysis

### *2.3.1. Proxy selection*

This study focuses on the detrital elements to identify Milankovitch cycles in the Miaolingian Series of Baltica in the Albjära-1 core ranging from 63.273 to 95.611 m AD. The detrital elements measured in this study (73.452-95.611 m AD) are Al, Si, Ti, K and Zr. Only Al, Ti, and Si data cover the entire Miaolingian Series in the Albjära-1 core, as K and Zr data are not included in the supplementary file of Zhao, et al.[2] for the 63.273-73.452 m AD portion of the ACU.

In this study, Ti was preferred due to its better expressed cyclicity, short residence time in the ocean (< 1 kyr), inert behavior to redox changes in seawater, and invariance to biological cycles (not an essential element for life)[68,69].

Ti is preferred over Al to avoid any misinterpretation due to the light mass of Al, which makes it close or below the limit of detection of the XRF core scanner technique.

Ti is preferred to Si in order to avoid errors in the calibration of the Si data. In the acid digestion procedure for ICP-MS, Si was not measured due to the digestion method. However, we recalculated the Si concentration (in %) as follows: Si = 100% - concentration of the other elements (in %).

Liu, et al.[69] demonstrated that, during late diagenesis (high thermal maturity) of black shales, Ti can be partially solubilized which could lead to misinterpretation of XRF results. However, the porosity of these rocks is limited, as is the migration of Ti that precipitates rapidly. This minimizes the possible errors in interpreting the results[69,70].

### *2.3.2. Cyclicity in the signal*

Sinnesael, et al.[71] recommend identifying any visible cyclicity or unconformity in the signal for detrital elements, through an overview of the tendencies observed in the depth domain prior to performing time series analysis.

This study reveals that GR and resistivity curves along the ACL exhibit patterns consistent with variations in detrital proxies (Ti, Si, Al, K). Collectively, these findings suggest minimal post-depositional alteration and support the use of the detrital proxies in time-series analysis (Fig. S5).

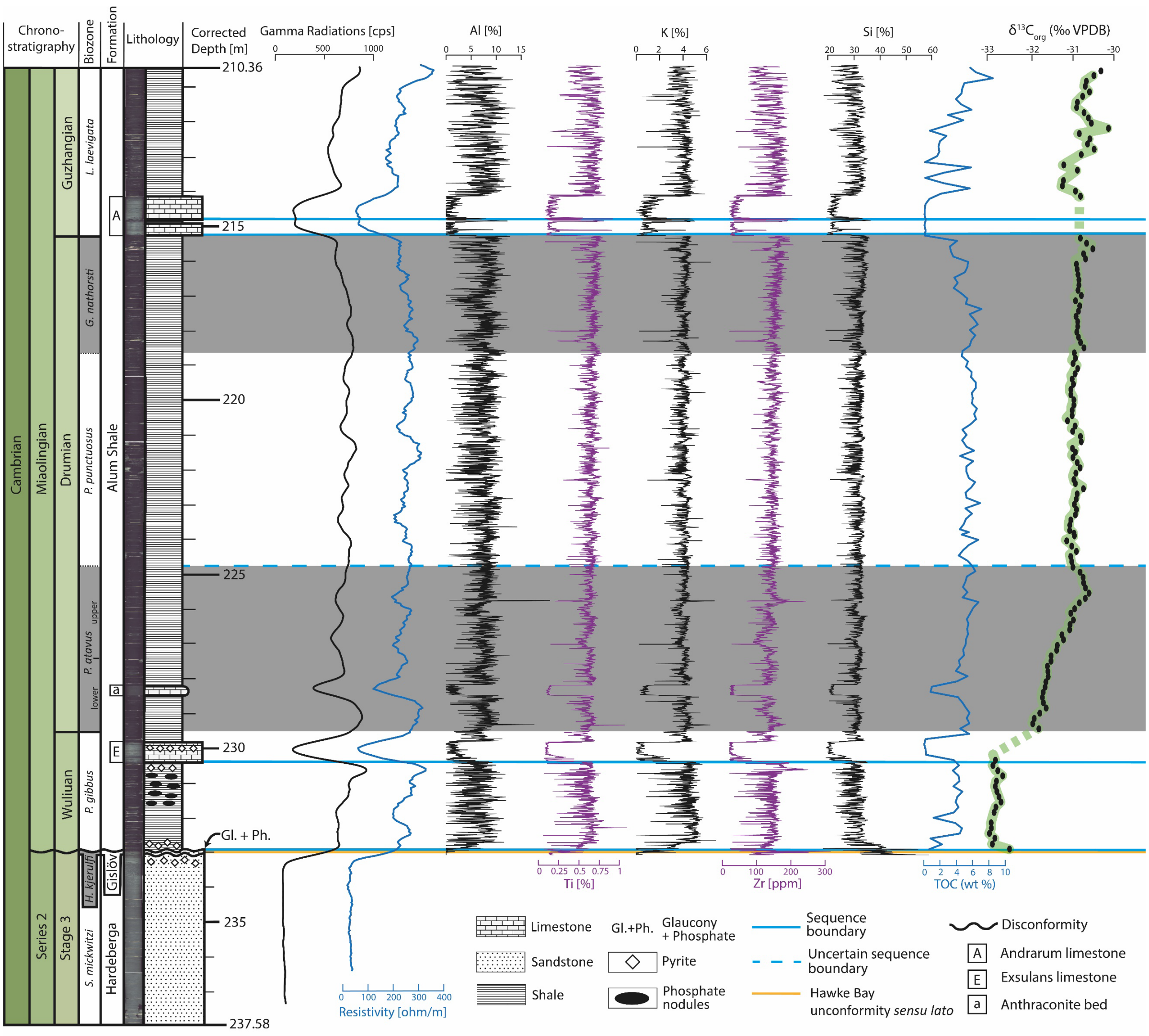


**Figure S5**: Visualization of the variations in depth domain of Gamma Radiation (GR), Formation Resistivity, detrital elements (Al, Ti, K, Zr, Si), TOC and $\delta^{13}C_{org}$ curves along the Alum Shale part of the ACL. The depths are corrected by adding +0.18 m to fit with the GR and resistivity curves. Well log curves are from Schovsbo and Nielsen[4].

### 2.3.3. <u>173 kyr identification</u>

We carried out a careful study and comparison of literature, MTM, EHA, ratio of frequencies and ASM results to evaluate and validate the record of the 173 kyr cycle in the signal in the ACL.

The 173 kyr long obliquity cycle is thought to arise from modulation in orbital inclination, which is driven by interactions between obliquity-related secular frequencies s3 and s6. These frequencies are associated with the orbital motions of Earth and Saturn, respectively[e.g.,72-74].

Zeebe and Kocken[75] outlined that the $I_{173}$ metronome can fluctuate or alternate between ~151 kyr (s4-s6) and ~173 kyr (s3-s6) due to variations in the s3 and s4 secular frequencies. They also suggested that the $I_{173}$ should only be used in sections where s3 and s4 appear stable and should not be used for extended periods.

The 405 kyr long-eccentricity metronome is generally preferred because it is more stable over time[e.g.,76,77]. However, as Laskar[74] and Zeebe and Lantink[78] explained, this cycle is not always observable, and its stability is questioned for sedimentary records older than a few hundred million years.

In the ACU, Zhao, et al.[2] identified the 405 kyr cycle with confidence in the Furongian Series (1.75 to 3 m periodicities). In the Guzhangian Stage, the 405 kyr cycle is still occurring, but it shows decreasing power and becomes more difficult to accurately identify in the EHA and 3π-MTM presented in their supplementary Fig. 4. In our data spanning from the early Guzhangian to the late Wuliuan stages, the power of the 1.75-3 m cycle, which is interpreted as the 405 kyr eccentricity, is weak to nearly absent. In contrast, the other orbital components identified by Zhao, et al.[2] are clearly expressed in similar frequency bands. The weakness of the 405 kyr cycle in our record may be attributed to its predicted instability during this period[75]. Additionally, in the astronomical solution R44 from Zeebe and Lantink[78], the 405-kyr cycles is observed at 528 Ma, but is dampened in power compared to the short-eccentricity cycle. This is similar to what we observed in the MTM of all the detrital elements. Due to the high latitude positioning of Baltica during the Miaolingian Epoch, the predicted dampening of power by the R44 astronomical solution would result in an even weaker expression of the 405 kyr cycle. While the 405 kyr cycle can appear weak compared to other cycles in high latitude localities, it has been observed with strong power in Lower Cambrian strata from localities near the equator[79,80].

The weakness of the 405 kyr cycle observed in our dataset could also suggest that its presence may be episodic, perhaps depending on when it became stable during the Cambrian. This hypothesis remains to be tested and would require expanded stratigraphic coverage across the entire Cambrian Period in various paleogeographic settings.

#### *2.3.3.1. MTM and EHA*

The 2π-MTM spectrum of the detrital elements revealed significant frequencies that align with observation in Zhao, et al.[2] and recalculation based on the data of Wu, et al.[23] (Fig. 4, 5AB, S6).

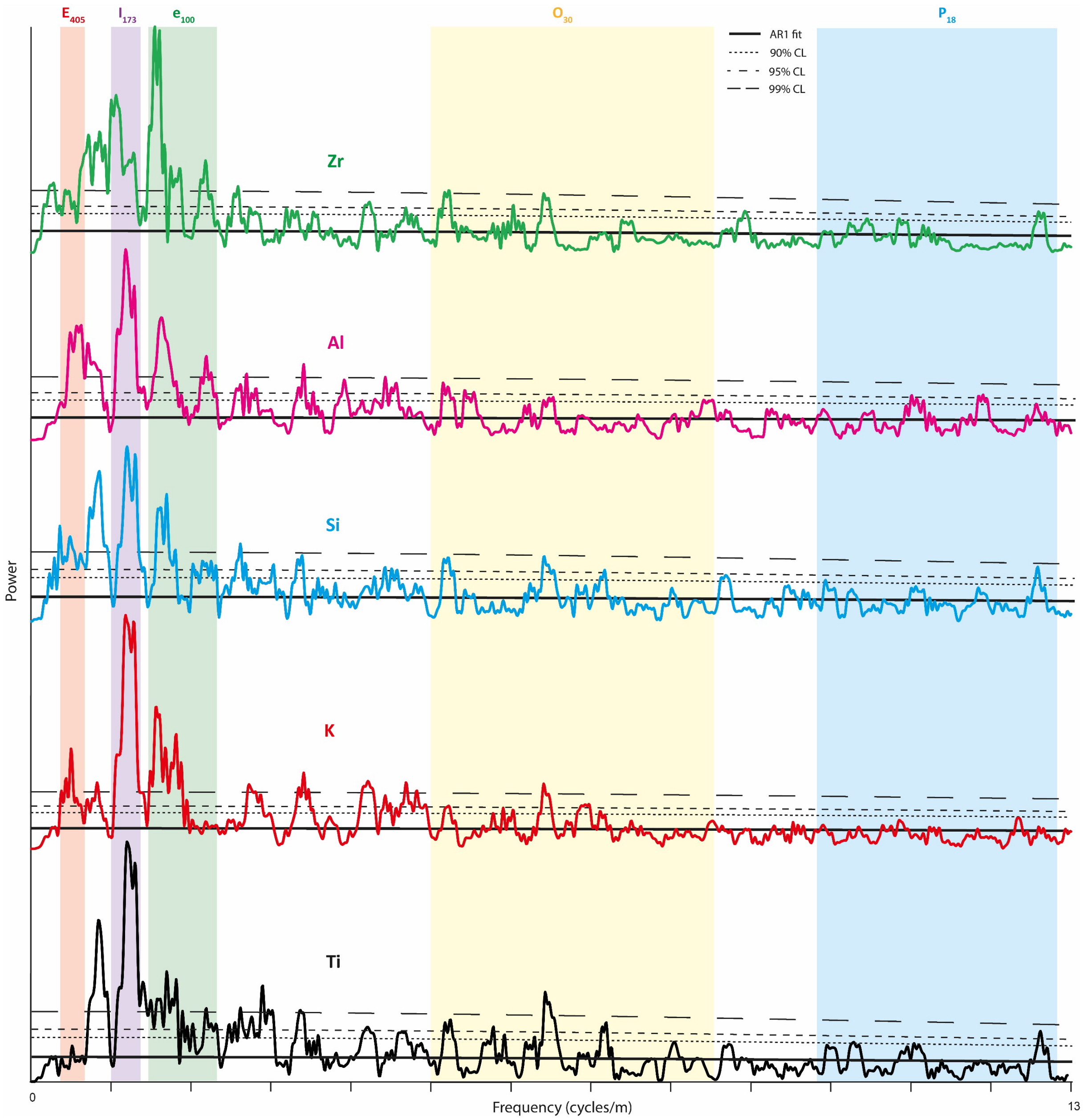


**Figure S6**: 2π MTM spectrum of the detrital elements (5 mm resampling and 20% LOWESS detrending) allowing the identification of Milankovitch bands.

Although the MTM is a powerful tool, it does not allow us to determine if the observed cyclicities occur continuously throughout the record or if they correspond to repeated, isolated events not linked to Milankovitch forcing. To identify the continuity of the cycles, on the Ti record, we used the EHA method, which confirmed the presence of eccentricity, obliquity and precession in the signal (Fig. 4). Similar observations to those shown in the EHA are observed in the CWT (Fig. S7).

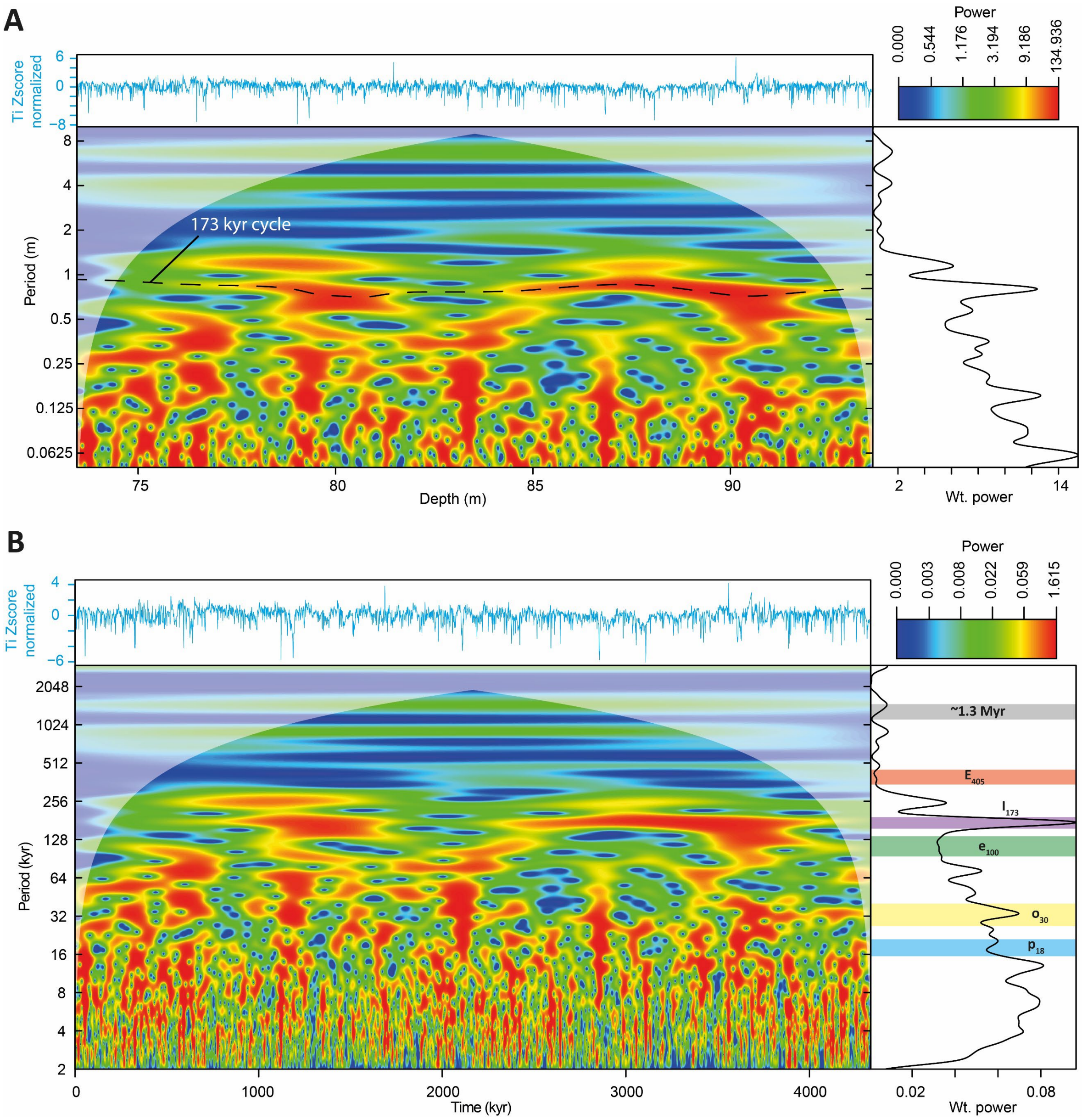


**Figure S7**: CWT in depth and time domains. A – Depth domain. B – Time domain.

### *2.3.3.2. Zhao et al (2022)'s supplementary data*

Spectral analysis of MTM, EHA, and CWT revealed that the power in the 1-1.35 cycles/m band of the ACL is above 99 % CL and is assumed to correspond to the $I_{173}$ cycle.

To verify our results are not artifacts, we compared them with the data presented by Zhao, et al.[2]. In their work, they identified strong obliquity but did not identify the 173 kyr cycle. However, we noticed a significant peak with a power slightly above the 90 % CL in a 1-1.5 cycles/m band in the Guzhangian–Paibian interval (subset IV) of their supplementary figure 4.

We interpret that this amount of power in a band close to our observation (1-1.32 cycles/m) as likely representing the $I_{173}$ cycle. The presence of the $I_{173}$ up to the Paibian Stage confirms that what we observe is unlikely to be an artifact resulting from data preparation and acquisition.

### 2.3.3.3. *Ratio of frequencies*

We conducted a comparison of the > 90% CL frequency ratios with $l_{173}$ on a total of 11 frequencies, which were estimated to correspond to long and short eccentricity, obliquity, and precession bands. The analysis revealed that all the 11 frequencies align on a line ($r^2 = 0.9997$) ranging from 0 kyr to 173 kyr, which suggests a reliable attribution of these frequencies to their corresponding astronomical cycles (Fig. S8).

| | Theoritical | | This study | |
|---|---|---|---|---|
| | Cycle duration | Ratio | Frequency | Ratio |
| $l_{173}$ | 173 | 1 | 1.151 | 1 |
| $e_{100}$ | 131.9 | 0.762 | 1.458 | 0.789 |
| | 124 | 0.717 | 1.548 | 0.744 |
| | 99.6 | 0.576 | 1.964 | 0.586 |
| | 95 | 0.549 | 2.093 | 0.550 |
| $o_{30}$ | 38.8 | 0.224 | 5.228 | 0.220 |
| | 32.1 | 0.186 | 6.240 | 0.184 |
| | 31.6 | 0.183 | 6.240 | 0.184 |
| | 31.3 | 0.181 | 6.240 | 0.184 |
| $p_{18}$ | 19.9 | 0.115 | 10.317 | 0.112 |
| | 19.3 | 0.112 | 10.317 | 0.112 |

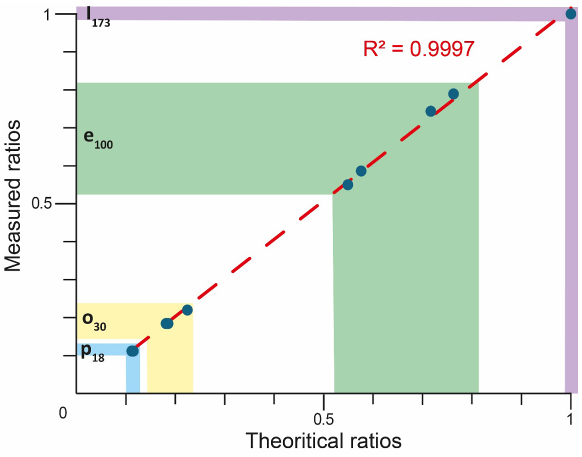


**Figure S8**: ratios of frequencies.

### 2.3.3.4. *ASM and average sedimentation rate*

We conducted the ASM analysis on the untuned dataset by comparing the frequencies > 90% CL located within Milankovitch bands with 17 Milankovitch frequencies from the literature for long and short eccentricity, obliquity and precession cycles (see supplementary datasets for more information).

This resulted in an average sedimentation rate of 0.493 cm/kyr (Fig. S9), which perfectly matches the excepted sedimentation rate of 0.4-0.6 cm/kyr for the Alum Shale Formation[e.g., 2,3,18]. The concordance of the ASM with the literature suggests a reliable identification of the astronomical cycles, including $l_{173}$.

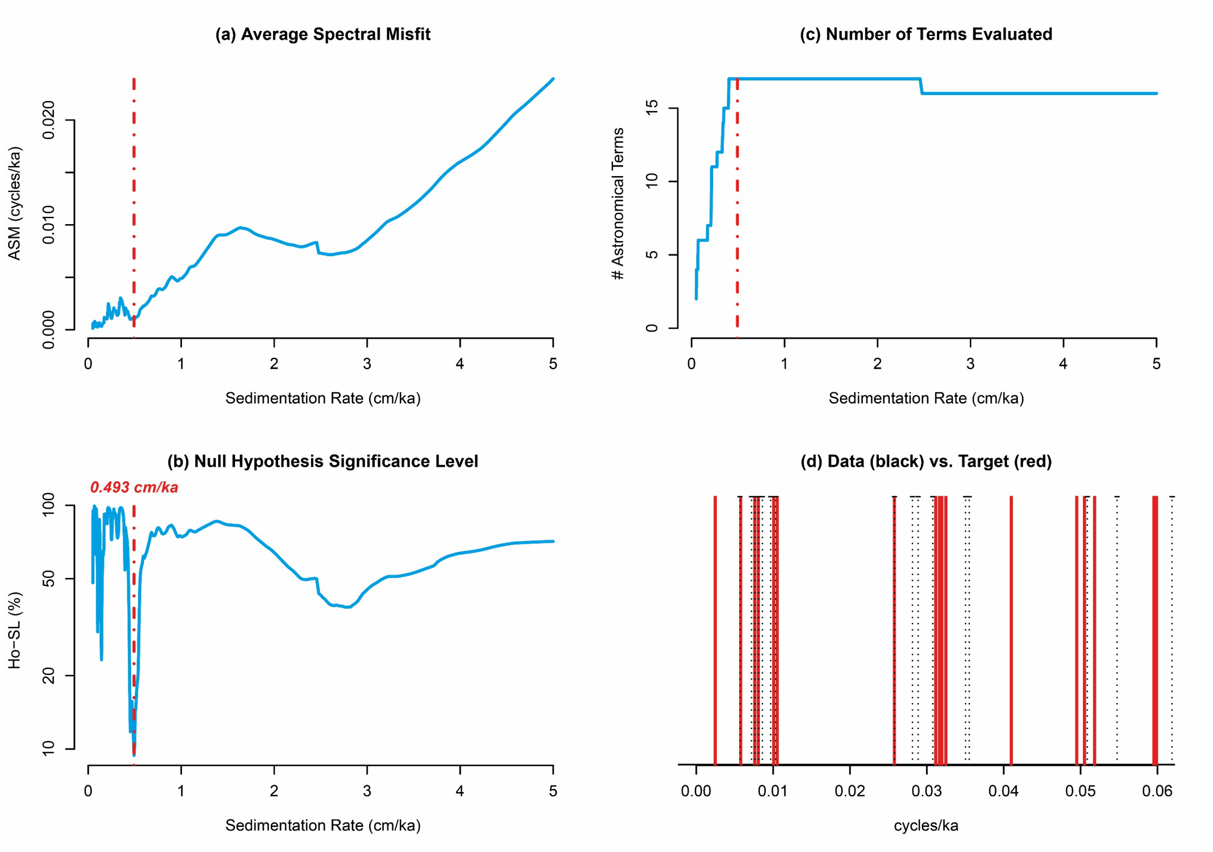


**Figure S9**: Average Spectral Misfit analysis performed on 17 Milankovitch-band frequencies targets.

We filtered and tuned the minima of the tracked frequency band (0.76-1m) for the detrital elements Al, Si, Ti, and K in the CWT to the 173 kyr cycle. This allowed us to calculate sedimentation rates for all of the detrital elements (Fig. S10). The mean sedimentation rate we obtain was 0.465 cm/kyr, which is consistent with the 0.493 cm/kyr ASM estimation and the literature.

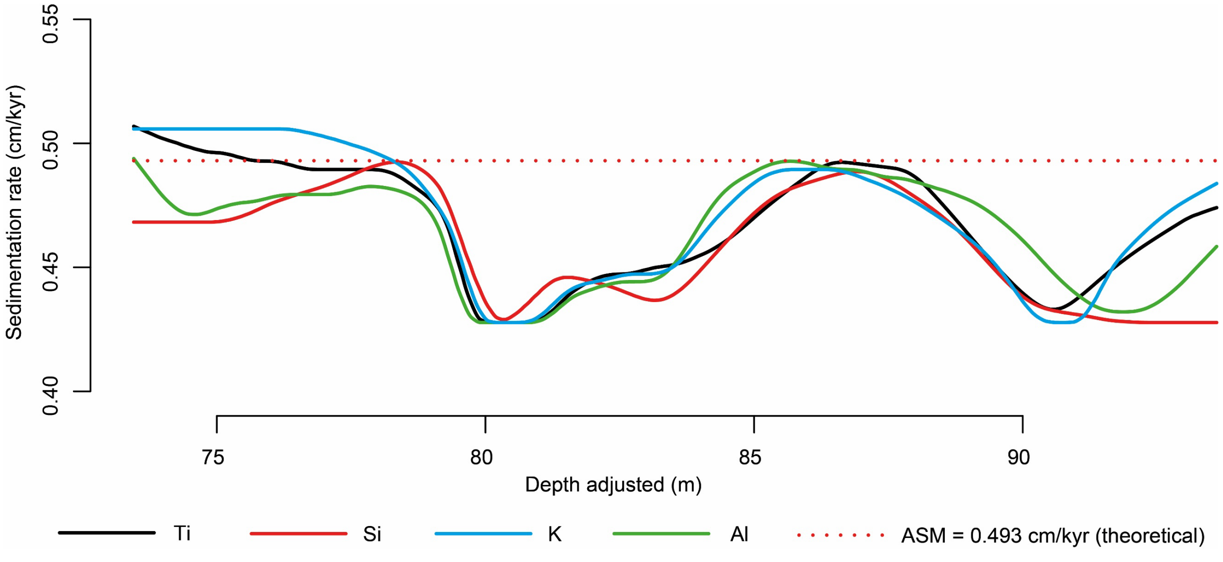


**Figure S10**: Sedimentation rate of the detrital elements and comparison with the ASM expectation.

### 2.3.4. Age-depth model

Tracking the powerful 0.76-1m band (173 kyr cycle) in the CWT for the Al, Si, Ti, and K elements enables us to construct a depth model of 173 kyr cycle tracking with reduced period uncertainty (Fig. S11).

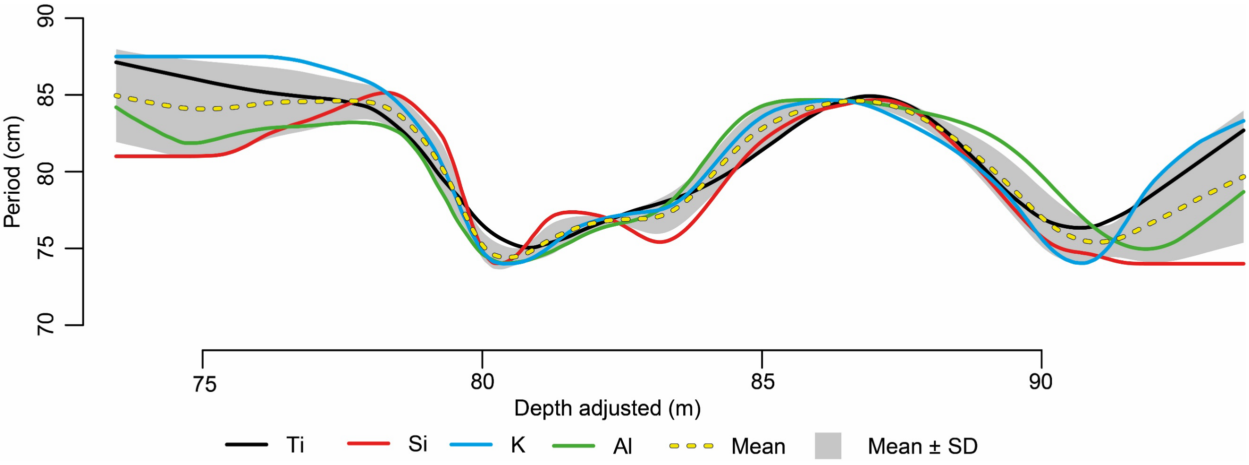


**Figure S11:** tracking of the 173 kyr in depth model for four detrital elements.

Tuning our depth model to the 173 kyr cycles allows us to build an age-depth model with a reduced cumulative uncertainty of maximum ± 167 kyr over the 4.3 Myr recorded in the core (Fig. S12).

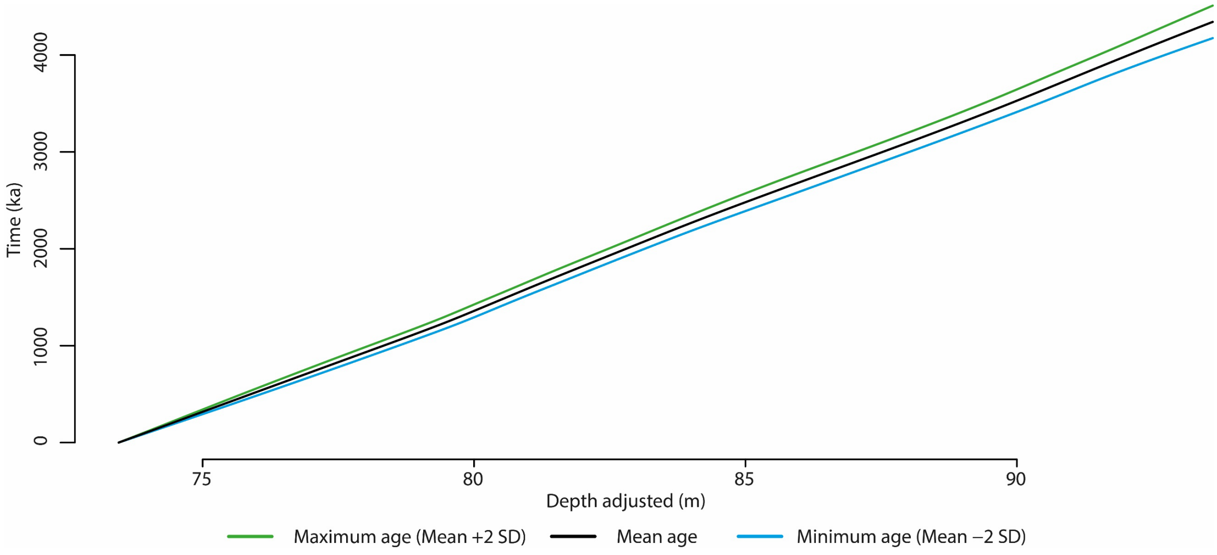


**Figure S12:** Robust age-depth model with low cumulative uncertainty.

### 2.3.5. Hilbert modulation analysis

Based on the literature and the spectral analysis observations (MTM, EHA, CWT, and ratios of frequencies), a strong obliquity signal is present in the ACL. The $l_{173}$ cycle and the ~ 1.2 Myr long obliquity cycles are theoretically the result of the modulation of the obliquity band[e.g.,72,74,81]. Therefore, to test the phase relationship between the obliquity band, the $l_{173}$ cycle, and the ~ 1.2 Myr cycle, we conducted an amplitude modulation analysis using the

Hilbert transform. This provides additional support for reliably identifying the Inclination metronome.

To test the phase relationship between the obliquity band and the $I_{173}$ metronome, the two cycles were isolated using a Taner filter respectively of 28-40 kyr and 155-195 kyr (Fig. 5C). The amplitude modulation of the obliquity band was extracted using the Hilbert transform function. Then, the $I_{173}$ metronome cycle was isolated from the amplitude modulation extracted from the obliquity band and compared to the $I_{173}$ metronome cycle isolated from the dataset. This analysis revealed a phase relationship between obliquity and $I_{173}$ in the lower part of the ACL (1.8-4 Myr), suggesting a reliable identification of the $I_{173}$ cycle. However, in the upper part of the ACL (0-1.8 Myr), a limited antiphase relationship in the obliquity is observed that tends to diminish over time. This antiphase relationship begins suddenly at ~ 1.8 Myr. This shift could be linked to one or more of the following: 1) poor preservation of the obliquity signal, 2) an unseen gap in the sedimentary record that would have caused the disappearance of a few obliquity cycles, which would have disrupted the Hilbert envelope, 3) overlap or interference of other events in the obliquity band at ~1.8 Myr, or 4) a non-direct response of the environment to obliquity forcing.

To test the phase relationship between the $I_{173}$ (155-195 kyr) and ~ 1.2 Myr cycles (1150-1300 kyr) on the non-detrended time series, the protocol explain above was conducted (Fig. 5C). The results suggest a reliable identification of the $I_{173}$ cycle along the ACL, given the phase relationship between the two cycles.

On a long timescale (173 kyr–1.2 Myr cycle comparison), a phase relationship is observed for the entire ACL, whereas at a shorter timescale (obliquity–173 kyr cycle comparison), a limited antiphase relationship is observed. These observations support the idea of a local and limited perturbation of the obliquity record as the long obliquity modulations are not significantly affected.

## 2.4. Astronomically-tuned age model for the Miaolingian Series

### *2.4.1. Duration estimates of Biozones*

Based on our astronomical timescale, the duration of the *Ptychagnotus forchhammeri* Biozone (*L. laevigata* + *Agnostus pisiformis* biozones) is estimated at 3.165 Myr, which is consistent with the ~3 Myr extrapolation proposed by Zhao, et al.[2]. The *Goniagnostus nathorsti* Biozone is estimated to span 691 ± 24 kyr, which closely matches the ~676 kyr duration estimated by Fang, et al.[82] for its equivalent interval (*G. nathorsti* + *Lejopyge armata* biozones) at the Guzhangian GSSP in South China. The *Ptychagnostus punctuosus* Biozone is estimated to represent 1,326 ± 32 kyr in duration. The *Ptychagnostus atavus* Biozone span 992 ± 22 kyr. The *Ptychagnostus gibbus* Biozone (part) is estimated to last 746 ± 49 kyr.

#### 2.4.2. Duration estimates of the Miaolingian Series, Stages and events

##### 2.4.2.1. Duration of Series and stages

Identifying the Wuliuan, Drumian, and Guzhangian boundaries allows us to delineate the Miaolingian Series in the Albjära-1 core. The Guzhangian–Paibian boundary is defined by the FAD of the agnostid *Glyptagnostus reticulatus*, which coincides with the onset of the SPICE[20,83,84]. In Baltica, this corresponds to the base of *Olenus* Biozone[2,85] and is identified at 62.273 m AD (= 497.277 +1.2/-0.9 Ma). The Guzhangian–Drumian boundary coincides with the base of the Andrarum limestone, which is located at 76.306 m AD (= 500.443 ± 0.941 Ma).

Therefore, the Guzhangian Stage is estimated to span approximately 3.165 Myr. This ~3 Myr duration aligns with the extrapolation proposed by Zhao, et al.[2]. Our estimate of the base of the Guzhangian Stage (~ 500.44 Myr) closely aligns with the estimated age of ~ 500.5 Ma reported in both the GTS 2020[20] and ICS 2024/12 timescales[86]. However, recent studies conducted in Laurentia[87,88] suggest a significantly younger age of ~494.4 Ma for the upper boundary of the Guzhangian Stage, compared to the ~497.3 Ma proposed by Zhao et al.[2]. This estimate, derived from maximum deposition ages of detrital zircons, is less precise than radiometric ages from ash beds, yet it provides a useful chronostratigraphic constraint.

Cothren, et al.[87] pointed out the nearly 3 Myr difference between the maximum age of deposition (MAD) and the age determine by the cyclostratigraphic study of Zhao, et al.[2]. They stated that the difference is likely due to incorrect tuning during the cyclostratigraphic study.

In 2022, when these MAD and ATS data were the only ones available, this explanation was plausible, as the Drumian and Wuliuan stages duration estimates were not precisely time constrained due to the limited number of radioisotope ages located within the Drumian Stage that were used to calibrate the entire Miaolingian Series.

Nonetheless, if the GTS 2020 stage durations estimate are correct (Guzhangian ~ 3.5 Myr; Drumian ~ 4.0 Myr; Wuliuan ~ 4.5 Myr)[20], the upper Guzhangian boundary's age of ~494.4 Ma would result in the base of the Guzhangian being at ~497.9 Ma, the base of the Drumian being at ~501.9 Ma and the base of the Wuliuan being at ~ 506.4 Ma. The base of the Wuliuan would align with the recent estimate of ~506.3 Ma for the Series-Miaolingian boundary from Karlstrom, et al.[89], Sundberg, et al.[90], Landing, et al.[91]. However, the base of the Drumian Stage would differ significantly from both the lowermost Drumian volcanic ash dated at 503.14 ± 0.13 Ma, as presented in Landing, et al.[92] as well as with the 505 ± 1 Ma age estimates from the upper Wuliuan of Spain[93]. Regarding the top of the Drumian Stage (= base of Guzhangian Stage), the ~497.9 Ma age estimate presented by Farrel et al.[88] does not align with both the upper and uppermost Drumian U-Pb age of 501.44 ± 0.94 Ma from Avalonia presented[94,95] nor with the uppermost Drumian U-Pb age of 500.9 ± 0.9 Ma from Spain[93].

In this study, we conducted a spectral analysis and built and ATS. This allowed us to determine the duration of the Drumian Stage (~3.0 Myr) and extrapolate on the duration of the Wuliuan Stage (~3.5 Myr). See below for more information. Anchoring our ATS to the U-Pb maximum deposition age of 494.35 ± 0.46 Ma at the base of the SPICE, as reported by Cothren et al.[87], results in the following age estimates: the base of the Guzhangian, Drumian, and Wuliuan stages would be respectively at 497.55 ± 0.94 Ma, 500.75 ± 1.02 Ma and ~ 504.25 Ma.

While these alternative ages are generally consistent with the estimates proposed by Farrell, et al.[88], they differ from those adopted in the GTS 2020[20] and the ICS[86]. This divergence could be linked to zircon contamination during transport or after deposition. This contamination could have resulted in a limited Pb loss and significantly younger ages, such as from metamorphic overprinting or interaction with diagenetic fluids[96]. Farrell et al.[88] observed a complex zircon spectrum for some sample and it is suggested by Farrell et al.[88] that it results from possible contamination or remaining Pb loss not entirely eliminated by chemical abrasion. Regarding the ages presented by Cothren et al.[87] on which the analysis has been performed are limited. For age 1 (494.35 ± 0.46 Ma), six zircon grains around 494.4 Ma were selected. For this age group, the population displays a bimodal distribution, with the largest group of zircon having ages around 499 Ma. The continuum observed between 499 to 494.5 Ma might be an indicator of contamination or Pb loss. For age 2 (494.16 ± 0.46 Ma), ten zircon grains corresponding to the majority of the estimated age are centered around 494.2 Ma. For this age group, only one dominant peak is observed, which suggests that there is no contamination or Pb loss for this group. For age 3 (492.84 ± 0.73 Ma), three zircon grains were selected. For this age group, most of the peaks observed in zircon ages are centered around 495 Ma, with ages ranging from 493 to 497 Ma. The slightly younger ages observed among the three selected grains are potentially contaminated or have experienced Pb loss

Conversely, when we anchor our ATS to the one presented in Zhao et al.[2], the bases of the Guzhangian, Drumian, and Wuliuan stages are, respectively, at 500.44 ± 0.94 Ma, 503.45 ± 1.02 Ma, and ~ 506.9 ± 1.07 Ma. These ages align with the ages of 500.55 ± 0.9 Ma[95] (uppermost Drumian) 500.9 ± 0.9 Ma (uppermost Drumian[93]), 501.44 ± 0.10 Ma (upper Drumian[94]), 503.14 ± 0.13 Ma (lowermost Drumian[92]), 505 ± 1 Ma (upper Wuliuan[93]) and ~506.3 Ma (Cambrian Stage 4 – Wuliuan boundary[89-91]). Nevertheless, it cannot be ruled out that the ages of Zhao et al.[2] may be younger in the future as the anchor point at the Cambrian-Ordovician boundary is an estimated age and not a direct U-Pb measurement. Thus, the uncertainty of the ages around 0.8 to 1.1 Ma should allows for the age of the stages to be younger without contradicting with the astronomical study of Zhao et al.[2].

Therefore, in this study, we chose to anchor our ATS to the data published by Zhao, et al.[2], which provides robust stratigraphic coherence and better aligns with current global and regional chronostratigraphic expectations.

Further studies should be conducted in Laurentia and other regions to confirm the duration of the Paibian and Guzhangian stages.

The Guzhangian–Drumian boundary is located at 76.306 m AD (= 500.443 ± 0.941 Ma), and the Drumian–Wuliuan boundary is identified at 90.295 m AD (= 503.451 ± 1.018 Ma). Thus, the Drumian Stage is estimated at 3.009 ± 0.078 Myr in duration, aligning with the theoretical expectation proposed by Farrell, et al.[88]. However, our estimate is 1 Myr shorter than the duration suggested by the GTS 2020[20] and the ICS timescale[86]. It should be noted that only two radioisotope ages were used as inputs for the Drumian Stage duration estimate in the GTS; one of these ages has an uncertainty of 2.4 Myr. Therefore, our estimate is reasonable and falls within the expected margin of error for current global standards. Our estimation of the

base of the Drumian Stage (~503.45 Ma), closely matches with the absolute age of 503.14 ± 0.13 Ma recorded at the base of the Drumian[90,92,97].

The preserved portion of the Wuliuan Stage located above the Hawke Bay Event *sensu lato* is estimated at 745.96 ± 48.9 kyr and the base of the Alum Shale Formation in the Albjära-1 core is estimated at 504.197 ± 1.067 Ma.

The sum of the durations of the Wuliuan, Drumian, and Guzhangian stages yields an estimate of 6.92 +0.386/-0.167 Myr for the Miaolingian Series in the Albjära-1 core. This estimate ranges from 504.197 ± 1.067 Ma to 497.277 +1.2/-0.9 Ma. It is important to note that this estimate reflects an incomplete record in Baltica because part of the Wuliuan was eroded due to the Hawke Bay Event *sensu lato*.

According to the GTS 2020[20], the *P. gibbus* Biozone, which is nearly complete in the ACL, represents about one-fifth of the Wuliuan Stage and is estimated to last ~750 kyr if complete. Extrapolating this proportion, the Wuliuan Stage would be ~3.5 Myr in duration. This would place the base of the Wuliuan Stage at ~ 506.9 ± 1.1 Ma, which is 2 Myr earlier than the ~509 Ma estimate from the GTS 2020[20] and the ICS[90]. Nevertheless, this earlier age aligns with the recent stratigraphic and geochronologic data from Karlstrom et al.[89], Sundberg et al.[90], and Landing et al.[91], which estimate the base of the Wuliuan to be at ~506.3 Ma. Continued refinement through an integrated approach that includes cyclostratigraphy, radiometric dating, and biostratigraphy will be essential to better constrain the lower boundary of the Miaolingian Series.

#### *2.4.2.2. DICE event*

The correlations presented in section 2.2.1 “Identification of the DICE event in Baltica”, allow us to reliably identify the DICE event in Baltica, which we estimate to have lasted ~750-800 kyr and to be essentially located within the *P. gibbus* Biozone. New cyclostratigraphic studies would be necessary to precisely determine the timing of the DICE in sections where the excursion is fully expressed and well constrained.

#### *2.4.2.3. Other events*

Based on the literature and our ATS, we were also able to estimate the missing time in the Albjära-1 core due to the Hawke Bay unconformity *sensu lato*. According to Nielsen and Schovsbo[1,10], the Læsa Formation is not recorded in northern and northeastern Scania. Consequently, there are no formations between the top of the Hardeberga Formation (= base of the *Schmidtiellus mickwitzi* Biozone) estimated at ~ 518.5 Ma (GTS2020) and the base of the Alum Shale Formation (= *Ptychagnostus gibbus* Biozone) estimated in this study at 504.197 ± 1.07 Ma. The resulting missing time is estimated at ~13 Myr.

## SUPPLEMENTARY REFERENCES


1 Nielsen, A. T. & Schovsbo, N. H. The lower Cambrian of Scandinavia: Depositional environment, sequence stratigraphy and palaeogeography. *Earth-Sci Rev* **107**, 207-310, doi:10.1016/j.earscirev.2010.12.004 (2011).

2 Zhao, Z. F. *et al.* Synchronizing rock clocks in the late Cambrian. *Nat Commun* **13**, doi:10.1038/s41467-022-29651-4 (2022).

3 Nielsen, A. T. & Schovsbo, N. H. The Cambro-Ordovician Alum Shale Fm og Scandinavia: Distribution, depositional environment and stratigraphy. 1-58 (2013).

4 Schovsbo, N. H. & Nielsen, A. T. *The Albjära-1, Gislövshammar-2 and Fågeltofta-2 scientific wells, Scania, southern Sweden. Biostratigraphy, log correlation and TOC analysis.*, Vol. 55 1-61 (2012).

5 Ahlberg, P. *et al.* Cambrian high-resolution biostratigraphy and carbon isotope chemostratigraphy in Scania, Sweden: first record of the SPICE and DICE excursions in Scandinavia. *Lethaia* **42**, 2-16, doi:10.1111/j.1502-3931.2008.00127.x (2009).

6 Cederström, P., Geyer, G., Ahlberg, P., Nilsson, C. H. & Ahlgren, J. Ellipsocephalid trilobites from Cambrian Series 2 and Stage 4, with emphasis on the taxonomy, morphological plasticity and biostratigraphic significance of ellipsocephalids from Scania, Sweden. *Fossil and Strata* **67**, 1-131, doi:10.18261/9788215065779-2022-01 (2022).

7 Hammer, O. & Svensen, H. H. Biostratigraphy and carbon and nitrogen geochemistry of the SPICE event in Cambrian low-grade metamorphic black shale, Southern Norway. *Palaeogeogr Palaeocl* **468**, 216-227, doi:10.1016/j.palaeo.2016.12.016 (2017).

8 Weidner, T. & Nielsen, A. T. The Middle Cambrian Paradoxides paradoxissimus Superzone on Oland, Sweden. *Gff* **131**, 253-268, doi:10.1080/11035890903189827 (2009).

9 Wolvers, H. M. & Maletz, J. The benthic graptolite (Opik, 1933) from the Middle Cambrian of Krekling, Oslo Region, Norway. *Norw J Geol* **96**, 311-318, doi:10.17850/n096-4-02 (2016).

10 Nielsen, A. T. & Schovsbo, N. H. The regressive early-mid Cambrian 'Hawke Bay Event' in Baltoscandia: Epeirogenic uplift in concert with eustasy. *Earth-Sci Rev* **151**, 288-350, doi:10.1016/j.earscirev.2015.09.012 (2015).

11 Westergård, A. H. in *Lunds Geologiska Fältklubb 1892-1942, 185-204* (1942).

12 Nielsen, A. T. & Schovsbo, N. H. Cambrian to basal Ordovician lithostratigraphy in southern Scandinavia. *B Geol Soc Denmark* **53**, 47-92 (2006).

13 Westergård, A. H. Agnostidea of the Middle Cambrian of Sweden. *Sveriges Geologiska Undersökning* **Serie C 477**, 1-141 (1946).

14 Álvaro, J. J., Ahlberg, P. & Axheimer, N. Skeletal carbonate productivity and phosphogenesis at the lower-middle Cambrian transition of Scania, southern Sweden. *Geol Mag* **147**, 59-76, doi:10.1017/S0016756809990021 (2010).

15 Lehnert, O., Ahlberg, P., Calner, M. & Joachimski, M. M. The Drumian Isotopic Carbon Excursion (DICE) in Scania, southern Sweden : a mirror of the onset of the Marjumiid Biomere at a time of increased primary production? *[Publication information missing]*, 172-174 (2013).

16 Lundberg, F. Cambrian stratigraphy and depositional dynamics based on the Tomten-1 drill core, Falbygden, Västergötland, Sweden. *Dissertation in Geology at Lund University* **No. 462**, 41 pp. (2016).

17 Zhao, Z. F. *et al.* Dynamic oceanic redox conditions across the late Cambrian SPICE event constrained by molybdenum and uranium isotopes. *Earth Planet Sc Lett* **604**, doi:10.1016/j.epsl.2023.118013 (2023).

18 Sørensen, A. L. *et al.* Astronomically forced climate change in the late Cambrian. *Earth Planet Sc Lett* **548**, doi:10.1016/j.epsl.2020.116475 (2020).

19 Geyer, G. A comprehensive Cambrian correlation chart. *Episodes* **42**, 321-332, doi:10.18814/epiiugs/2019/019026 (2019).

20 Peng, S. C., Babcock, L. E. & Ahlberg, P. in *Geological Time Scale 2020* Vol. 2 (eds F. M. Gradstein, J. G. Ogg, M. D. Schmitz, & G. M. Ogg) Ch. 19, 565-629 (Elsevier, 2020).
21 Landing, E., Webster, M. & Bowser, S. S. Terminal Ediacaran-Late Ordovician evolution of the NE Laurentia palaeocontinent: rift-drift-onset of Taconic Orogeny, sea-level change and 'Hawke Bay' onlap (not offlap). *Geological Society, London, Special Publications* **542**, doi:10.1144/sp542-2023-4 (2023).
22 Palmer, A. R. & James, N. P. in *The Caledonides in the USA* Vol. 2 (ed D. R. Wones) 15-18 (Department of Geological Sciences, Virginia Polytechnic Institue and State University Memoirs, 1980).
23 Wu, Y. J., Malinverno, A., Meyers, S. R. & Hinnov, L. A. A 650-Myr history of Earth's axial precession frequency and the evolution of the Earth-Moon system derived from cyclostratigraphy. *Sci Adv* **10**, doi:10.1126/sciadv.ado2412 (2024).
24 Bluman, A. G. *Elementary statistics: a step by step approach*. 9th edn, (2014).
25 Wang, X., Liesaputra, V., Liu, Z., Wang, Y. & Huang, Z. An in-depth survey on Deep Learning-based Motor Imagery Electroencephalogram (EEG) classification. *Artif Intell Med* **147**, 102738, doi:10.1016/j.artmed.2023.102738 (2024).
26 Wu, H., Hayes, M. J., Weiss, A. & Hu, Q. An evaluation of the Standardized Precipitation Index, the China-Z Index and the statistical Z-Score. *Int J Climatol* **21**, 745-758, doi:10.1002/joc.658 (2001).
27 Tullberg, S. A. Om Agnostus-arterna i de kambriska aflagringarne vid Andrarum. *Fossils and Strata* **32**, 1-202 (1880).
28 Brøgger, W. C. Om Paradoxidesskifrene ved Krekling. *Nyt Magazin for Naturvidenskaberne* **24**, 18-88 (1878).
29 Kobayashi, T. On the agnostids (Part 1). *Journal of the Faculty of Science, Imperial University of Tpkyo, Section II* **5**, 69-198 (1939).
30 Angelin, N. P. *Palaeontologia Scandinavica. I: Iconographia crustaceorum formationis transitionis*. (Fasc. 1, 24 pp. Lund, 1851).
31 Linnarsson, J. G. O. Om Vestergötlands cambriska och siluriska aflagringar. *Kongliga Svenska Vetenskaps-Akademiens Handlingar* **8**, 1-89 (1869).
32 Ahlberg, P. *et al.* Integrated Cambrian biostratigraphy and carbon isotope chemostratigraphy of the Grönhögen-2015 drill core, Öland, Sweden. *Geol Mag* **156**, 935-949, doi:10.1017/S0016756818000298 (2019).
33 Høyberget, M. & Bruton, D. L. Middle Cambrian trilobites of the suborders Agnostina and Eodiscina from the Oslo Region, Norway. *Palaeontographica* **286**, 1-100 (2008).
34 Lauridsen, B. W. The Cambrian-Tremadoc interval of the Albjära-1 drill-core, Scania, Sweden (unpublished thesis). *University of Copenhagen* (2000).
35 Krissansen-Totton, J., Buick, R. & Catling, D. C. A statistical analysis of the carbon isotope record from the Archean to Phanerozoic and implications for the rise of oxygen. *Am J Sci* **315**, 275-316, doi:10.2475/04.2015.01 (2015).
36 Sharp, Z. *Principles of stable isotope geochemistry*. 2nd edn, (University of New Mexico, 2017).
37 Kaufman, A. J. & Knoll, A. H. Neoproterozoic variations in the C-isotopic composition of seawater - stratigraphic and biogeochemical implications. *Precambrian Res* **73**, 27-49, doi:10.1016/0301-9268(94)00070-8 (1995).
38 Li, D. *et al.* A paired carbonate-organic $\delta^{13}C$ approach to understanding the Cambrian Drumian carbon isotope excursion (DICE). *Precambrian Res* **349**, doi:10.1016/j.precamres.2019.105503 (2020).
39 Álvaro, J. J. in *Carbon Isotope Stratigraphy* Vol. 5 *Stratigraphy & Timescales* (ed M. Montenari) Ch. 5, 269-317 (Elsevier, 2020).
40 Peters, K. E. Guidelines for Evaluating Petroleum Source Rock Using Programmed Pyrolysis. *Aapg Bull* **70**, 318-329 (1986).
41 Buchardt, B. & Lewan, M. D. Reflectance of vitrinite-like macerals as a thermal maturity index for Cambrian Ordovician Alum Shale, Southern Scandinavia. *Aapg Bull* **74**, 394-406 (1990).

42 Sanei, H., Petersen, H. I., Schovsbo, N. H., Jiang, C. & Goodsite, M. E. Petrographic and geochemical composition of kerogen in the Furongian (U.Cambrian) Alum Shale, central Sweden: Reflections on the petroleum generation potential. *Int J Coal Geol* **132**, 158-169, doi:10.1016/j.coal.2014.08.010 (2014).

43 Zhao, Z. F. *et al.* High-resolution carbon isotope chemostratigraphy of the middle Cambrian to lowermost Ordovician in southern Scandinavia: Implications for global correlation. *Global Planet Change* **209**, doi:10.1016/j.gloplacha.2022.103751 (2022).

44 Schovsbo, N. H. Maturity database for the Lower Palaeozoic in Scandinavia. ArcGis project version 1.0. 1-29 (2011).

45 Moore, D. M. & Reynolds, R. J. X-ray Diffraction and the Identification and Analysis of Clay Minerals. 332 pp (1989).

46 Kübler, B. La cristallinité de l'illite et les zones tout à fait supérieures du métamorphisme. I. *Etages tectoniques, Colloque de Neuchâtel 1966*, Edition de la Baconnière, Neuchâtel, Switzerland. 105-121. (1967).

47 Schovsbo, N. H., Mathiesen, A. & Nielsen, A. T. Thermal modelling of the Lower Palaeozoic shales on Zealand and Scania., 1-36 (2015).

48 Babcock, L. E., Robison, R. A., Rees, M. N., Peng, S.-C. & Saltzman, M. R. The Global boundary Stratotype Section and Point (GSSP) of the Drumian Stage (Cambrian) in the Drum Mountains, Utah, USA. *Episodes* **30**, 85-95, doi:10.18814/epiiugs/2007/v30i2/003 (2007).

49 Zhu, M.-Y., Babcock, L. E. & Peng, S.-C. Advances in Cambrian stratigraphy and paleontology: Integrating correlation techniques, paleobiology, taphonomy and paleoenvironmental reconstruction. *Palaeoworld* **15**, 217-222, doi:10.1016/j.palwor.2006.10.016 (2006).

50 Gu, Q., Xing, F., Azmy, K., Jin, X., Chen, X., Zhang, C., Qian, H. & Wen, J. A comprehensive analysis of the Cambrian Drumian carbon isotope excursion (DICE): Insights from paleoclimate, paleoenvironment and global correlations. *Sediment Geol* **481**, 1-15, doi: 10.1016/j.sedgeo.2025.106875 (2025).

51 Jamart, V. *et al.* The Cambrian ROECE and DICE carbon isotope excursions in western Gondwana (Montagne Noire, southern France): Implications for regional and global correlations of the Miaolingian Series. *Palaeogeogr Palaeocl* **670**, 1-14, doi:10.1016/j.palaeo.2025.112951 (2025).

52 Kouchinsky, A. *et al.* Early-middle Cambrian stratigraphy and faunas from northern Siberia. *Acta Palaeontol Pol* **67**, 341-464, doi:10.4202/app.00930.2021 (2022).

53 Shembilu, N. & Azmy, K. Carbon-isotope stratigraphy of the middle-upper Cambrian in eastern Laurentia: Implications for global correlation. *Mar Petrol Geol* **128**, doi:10.1016/j.marpetgeo.2021.105052 (2021).

54 Dong, Y. X. *et al.* The paleoenvironmental evolution of the Cambrian Miaolingian Epoch in South China. *Palaeogeogr Palaeocl* **661**, doi:10.1016/j.palaeo.2024.112713 (2025).

55 Li, Z., Yang, X. L., Yuan, Y., Hu, Y. & Wang, D. Z. Carbon isotope fluctuations from the Cambrian Jialao Formation in Jianhe County, Guizhou Province, China: Implications for stratigraphic correlation. *Palaeogeogr Palaeocl* **661**, doi:10.1016/j.palaeo.2024.112680 (2025).

56 Zhu, M.-Y., Zhang, J. M., Li, G. X. & Yang, A. H. Evolution of C isotopes in the Cambrian of China: implications for Cambrian subdivision and trilobite mass extinctions. *Geobios-Lyon* **37**, 287-301, doi:10.1016/j.geobios.2003.06.001 (2004).

57 Zuo, J., Zhu, X., Chen, Y. & Zhai, W. Carbon isotope from shallow marine system in North China: Implications for stratigraphical correlation and sea-level changes in Cambrian. *J Earth Sci-China* **34**, 1777-1792, doi:10.1007/s12583-021-1463-6 (2023).

58 LeRoy, M. A., Gill, B. C., Sperling, E. A., McKenzie, N. R. & Park, T. Y. S. Variable redox conditions as an evolutionary driver? A multi-basin comparison of redox in the middle and later Cambrian oceans (Drumian-Paibian). *Palaeogeogr Palaeocl* **566**, doi:10.1016/j.palaeo.2020.110209 (2021).

59 Wu, Y. S. *et al.* Evolution patterns of seawater carbon isotope composition during the Cambrian and their stratigraphic significance. *Geol J* **56**, 457-474, doi:10.1002/gj.3957 (2021).

60 Yang, X., Li, Z., Gao, B. & Zhou, Y. The Cambrian Drumian carbon isotope excursion (DICE) in the Keping area of the northwestern Tarim Basin, NW China. *Palaeogeogr Palaeocl* **571**, doi:10.1016/j.palaeo.2021.110385 (2021).
61 Faggetter, L. E. *et al.* Sequence stratigraphy, chemostratigraphy and facies analysis of Cambrian Series 2-Series 3 boundary strata in northwestern Scotland. *Geol Mag* **155**, 865-877, doi:10.1017/S0016756816000947 (2016).
62 Montañez, I. P., Osleger, D. A., Banner, J. L., Mack, L. E. & Musgrove, M. Evolution of the Sr and C Isotope Composition of Cambrian Oceans. *GSA Today* **10**, 7 (2000).
63 Howley, R. A. & Jiang, G. The Cambrian Drumian carbon isotope excursion (DICE) in the Great Basin, western United States. *Palaeogeogr Palaeocl* **296**, 138-150, doi:10.1016/j.palaeo.2010.07.001 (2010).
64 Pagès, A. & Schmid, S. Euxinia linked to the Cambrian Drumian carbon isotope excursion (DICE) in Australia: Geochemical and chemostratigraphic evidence. *Palaeogeogr Palaeocl* **461**, 65-76, doi:10.1016/j.palaeo.2016.08.008 (2016).
65 Schmid, S. Chemostratigraphy and palaeo-environmental characterisation of the Cambrian stratigraphy in the Amadeus Basin, Australia. *Chem Geol* **451**, 169-182, doi:10.1016/j.chemgeo.2017.01.019 (2017).
66 Gomez, F. J., Ogle, N., Astin, R. A. & Kalin, R. M. Paleoenvironmental and carbon-oxygen isotope record of middle Cambrian carbonates (la laja formation) in the Argentine precordillera. *J Sediment Res* **77**, 826-842, doi:10.2110/jsr.2007.079 (2007).
67 Gomez, F. J. & Astini, R. A. Sedimentology and sequence stratigraphy from a mixed (carbonate-siliciclastic) rift to passive margin transition: The early to middle Cambrian of the Argentine Precordillera. *Sediment Geol* **316**, 39-61, doi:10.1016/j.sedgeo.2014.11.003 (2015).
68 Aarons, S. M. *et al.* Titanium transport and isotopic fractionation in the Critical Zone. *Geochim Cosmochim Ac* **352**, 175-193, doi:10.1016/j.gca.2023.05.008 (2023).
69 Liu, Z. R. R., Zhou, M. F., Williams-Jones, A. E., Wang, W. & Gao, J. F. Diagenetic mobilization of Ti and formation of brookite/anatase in early Cambrian black shales, South China. *Chem Geol* **506**, 79-96, doi:10.1016/j.chemgeo.2018.12.022 (2019).
70 Gao, F. L. *et al.* Pore characteristics and dominant controlling factors of overmature shales: A case study of the Wangyinpu and Guanyintang Formations in the Jiangxi Xiuwu Basin. *Interpretation-J Sub* **6**, T393-T412, doi:10.1190/Int-2017-0128.1 (2018).
71 Sinnesael, M. *et al.* The Cyclostratigraphy Intercomparison Project (CIP): consistency, merits and pitfalls. *Earth-Sci Rev* **199**, doi:10.1016/j.earscirev.2019.102965 (2019).
72 Boulila, S. *et al.* Towards a robust and consistent middle Eocene astronomical timescale. *Earth Planet Sc Lett* **486**, 94-107, doi:10.1016/j.epsl.2018.01.003 (2018).
73 Charbonnier, G. *et al.* Obliquity pacing of the hydrological cycle during the Oceanic Anoxic Event 2. *Earth Planet Sc Lett* **499**, 266-277, doi:10.1016/j.epsl.2018.07.029 (2018).
74 Laskar, J. in *Geologic Time Scale 2020* (eds F. M. Gradstein, J. G. Ogg, M. D. Schmitz, & G. M. Ogg) Ch. 4, 139-158 (Elsevier, 2020).
75 Zeebe, R. E. & Kocken, I. J. Applying astronomical solutions and Milanković forcing in the Earth sciences. *Earth-Sci Rev* **261**, 1-22, doi:10.1016/j.earscirev.2024.104959 (2025).
76 Pas, D., Elrick, M., Da silva, A. C., Hinnov, L., Jamart, V., Thaureau, M. & Arts, M. Millenial-scale climate cycles modulated by Milankovitch forcing in the middle Cambrian (ca. 500 Ma) Marjum Formation, Utah, USA. *Geology* **52**, 605-609, doi:10 .1130 /G52182.1 (2024).
77 Zeeden, C., Laskar, J., De Vleeschouwer, D., Pas, D. & Da Silva, A. C. Earth's rotation and Earth-Moon distance in the Devonian derived from multiple geological records. *Earth Planet Sc Lett* **621**, doi:10.1016/j.epsl.2023.118348 (2023).
78 Zeebe, R. E. & Lantink, M. L. A Secular Solar System Resonance that Disrupts the Dominant Cycle in Earth's Orbital Eccentricity (g2-g5): Implications for Astrochronology. *Astron J* **167**, doi:10.3847/1538-3881/ad32cf (2024).
79 Zhang, T. *et al.* Orbital forcing of tropical climate dynamics in the Early Cambrian. *Global Planet Change* **219**, doi:10.1016/j.gloplacha.2022.103985 (2022).

80 Zhang, T. *et al.* Orbitally-paced climate change in the early Cambrian and its implications for the history of the Solar System. *Earth Planet Sc Lett* **583**, doi:10.1016/j.epsl.2022.117420 (2022).
81 Spiering, B. R. *et al.* Initial cyclostratigraphy of the middle Nama Group (Schwarzrand Subgroup) in southern Namibia. *Precambrian Res* **397**, doi:10.1016/j.precamres.2023.107200 (2023).
82 Fang, J. C. *et al.* Cyclostratigraphy of the global stratotype section and point (GSSP) of the basal Guzhangian Stage of the Cambrian Period. *Palaeogeogr Palaeocl* **540**, doi:10.1016/j.palaeo.2019.109530 (2020).
83 Peng, S. C. *et al.* Global Standard Stratotype-section and Point (GSSP) of the Furongian Series and Paibian Stage (Cambrian). *Lethaia* **37**, 365-379, doi:10.1080/00241160410002081 (2004).
84 Saltzman, M. R. *et al.* A global carbon isotope excursion (SPICE) during the late Cambrian: relation to trilobite extinctions, organic-matter burial and sea level. *Palaeogeogr Palaeocl* **162**, 211-223, doi:10.1016/S0031-0182(00)00128-0 (2000).
85 Nielsen, A. T., Høyberget, M. & Ahlberg, P. The Furongian (upper Cambrian) Alum Shale of Scandinavia: revision of zonation. *Lethaia* **53**, 462-485, doi:10.1111/let.12370 (2020).
86 Cohen, K., Haper, D., Gibbard, P. & Car, N. The ICS international chronostratigraphic chart this decade. *Episodes* **48**, 105-115, doi:10.18814/epiiugs/2025/025001 (2025).
87 Cothren, H. R., Farrell, T. P., Sundberg, F. A., Dehler, C. M. & Schmitz, M. D. Novel age constraints for the onset of the Steptoean Positive Isotopic Carbon Excursion (SPICE) and the late Cambrian time scale using high-precision U-Pb detrital zircon ages. *Geology* **50**, 1415-1420, doi:10.1130/G50434.1 (2022).
88 Farrell, T. P. *et al.* Revising the late Cambrian time scale and the duration of the SPICE event using a novel Bayesian age modeling approach. *GSA Bulletin 2025*, 1-26, doi:10.1130/B37919.1 (2025).
89 Karlstrom, K. E. *et al.* Redefining the Tonto Group of Grand Canyon and recalibrating the Cambrian time scale. *Geology* **48**, 425-430, doi:10.1130/G46755.1 (2020).
90 Sundberg, F. A. *et al.* Asynchronous trilobite extinctions at the early to middle Cambrian transition. *Geology* **48**, 441-445, doi:10.1130/G46913.1 (2020).
91 Landing, E., Schmitz, M. D., Westrop, S. R. & Geyer, G. U-Pb zircon dates from North American and British Avalonia bracket the Lower-Middle Cambrian boundary interval, with evaluation of the Miaolingian Series as a global unit. *Geol Mag* **160**, 1790-1816, doi:10.1017/S0016756823000729 (2023).
92 Landing, E., Geyer, G., Buchwaldt, R. & Bowring, S. A. Geochronology of the Cambrian: a precise Middle Cambrian U-Pb zircon date from the German margin of West Gondwana. *Geol Mag* **152**, 28-40, doi:10.1017/S0016756814000119 (2015).
93 Palacios, T., Jensen, S., Álvaro, J.J., Santos Zaldeugui, J.F., Eguiluz, L., Corfu, F. & Gil Ibarguchi, I. Acritarch-based chronostratigraphic and radiometric calibration of the Cambrian volcanosedimentary Vallehondo and Playón formations in the Cambrian Ossa-Morena Rift, Spain. *Palaeogeogr Palaeocl* **565**, 1-32, doi:10.1016/j.palaeo.2021.110216 (2021).
94 Landing, E., Schmitz, M.D., Westrop, S.R. & Geyer G. U-Pb volcanic zircon dates from New Brunswick constrain the Middle Cambrian Drumian Stage and trans-Avalonian green–black boundary. *Geol Mag* **162**, 1-14, doi: 10.1017/S0016756825100137 (2025).
95 Landing, E., Schmitz, M. D., Westrop, S. R. & Geyer, G. New Middle Cambrian (Drumian) U-Pb age of Cape Breton Island volcanism and Cambrian–lowest Ordovician stratigraphic reevaluation confirm the Bourinot belt (southern "Bras d'Or terrane") is Avalonian. *Can J Earth Sci*, doi:10.1139/cjes-2025-0044 (2025).
96 Andersen, T., Elburg, M. A. & Magwaza, B. N. Sources of bias in detrital zircon geochronology: Discordance, concealed lead loss and common lead correction. *Earth-Sci Rev* **197**, 1-15, doi: 10.1016/j.earscirev.2019.102899 (2019).
97 Rowland, S. M., Korolev, S., Hagadorn, J. W. & Ghosh, K. Frenchman Mountain Dolostone: A new formation of the Cambrian Tonto Group, Grand Canyon and Basin and Range, USA. *Geosphere* **19**, 719-747, doi:10.1130/Ges02514.1 (2023).

***PART II: DETAILED SPECTRAL ANALYSIS' PROTOCOL***

# Astronomical calibration of the middle Cambrian in Baltica: global carbon cycle synchronization and climate dynamics

Authors: Valentin JAMART, Damien PAS, Linda A. HINNOV, Jorge E. SPANGENBERG, Thierry ADATTE, Arne T. NIELSEN, Niels H. SCHOVSBO, Nicolas THIBAULT, Michiel ARTS, Allison C. DALEY

## Work environment, packages and data loading

```
#Setting of the work environment
setwd("C:/Users/vjamart/OneDrive - Université de Lausanne/Bureau/Draft Alum Shale/R code/html"

#Loading of the packages that will be used in this study
library(WaverideR)
library(astrochron)
```

```
Welcome to astrochron v1.5 (2025-04-28)
```

```
library(matrixStats)
```

```
Warning: le package 'matrixStats' a été compilé avec la version R 4.2.3
```

```
#Loading of the dataset file
Alb <- read.csv("Z score.csv", sep=";")
```

## Step 1: Data selection, resampling and detrending

### 1.1. Selecting Titanium data

```
# Selection of the data
alb_Ti <- cbind(Alb$DepthAdj,Alb$Ti)
alb_Ti <- na.omit(alb_Ti)
alb_Ti[!is.finite(alb_Ti)] <- NA
alb_Ti <- na.omit(alb_Ti)

#Isolation of the studied time interval
alb_Ti <- iso(dat=alb_Ti, xmin=73.452, xmax=100)
```

```
----- ISOLATE STRATIGRAPHIC DATA BY LOCATION -----
 * Number of data points= 30577
 * Number of columns= 2
```

```
 * Minimum= 62.111 , Maximum= 93.61
 * Isolating data between 73.452 and 100
 * Number of data points following culling= 19312
```

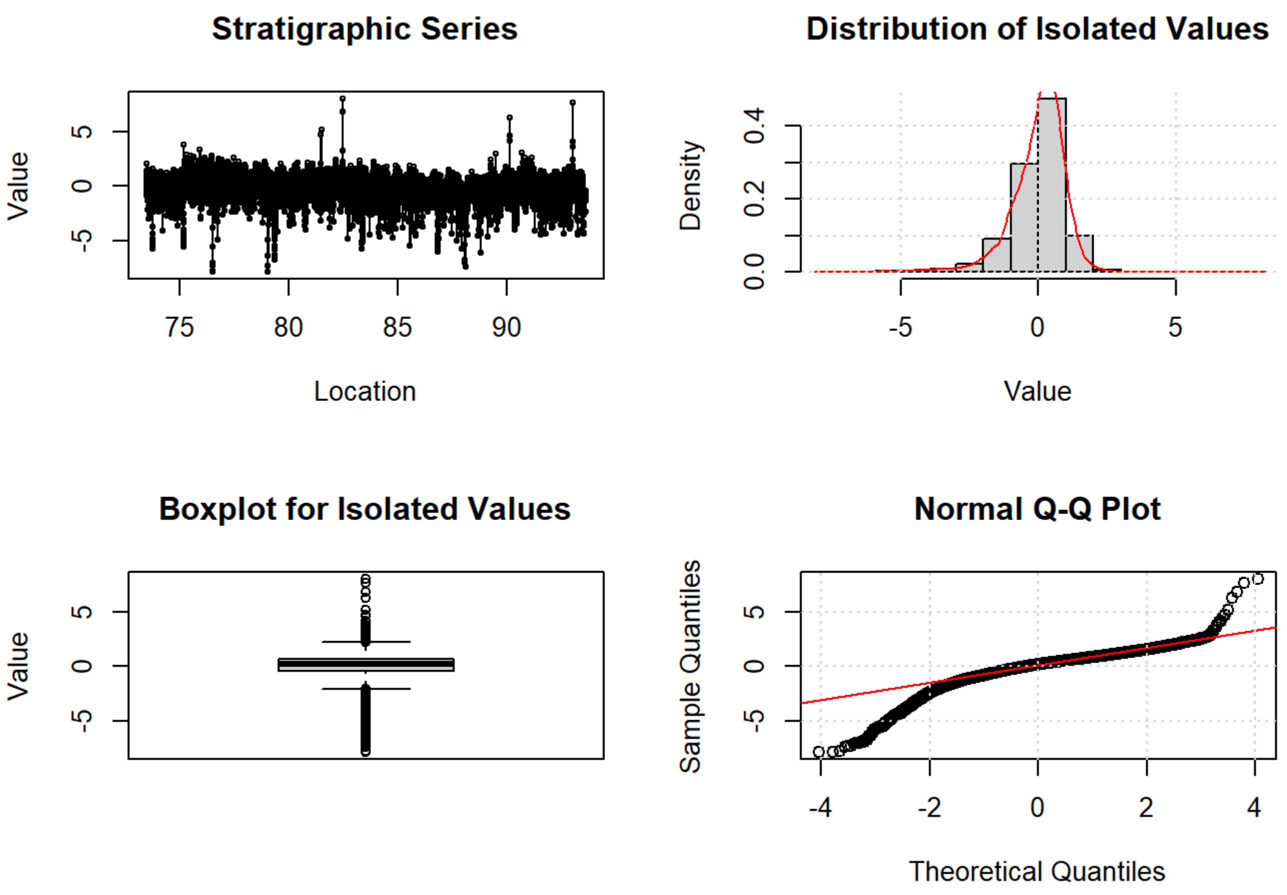


```r
alb_Ti <- linterp(alb_Ti, genplot=T)
```

```
----- APPLYING PIECEWISE-LINEAR INTERPOLATION TO STRATIGRAPHIC SERIES -----

 * Number of samples= 19312
 * Determining median sampling interval for series
 * Will interpolate to median sampling interval of 0.001
 * New number of samples= 20158
```

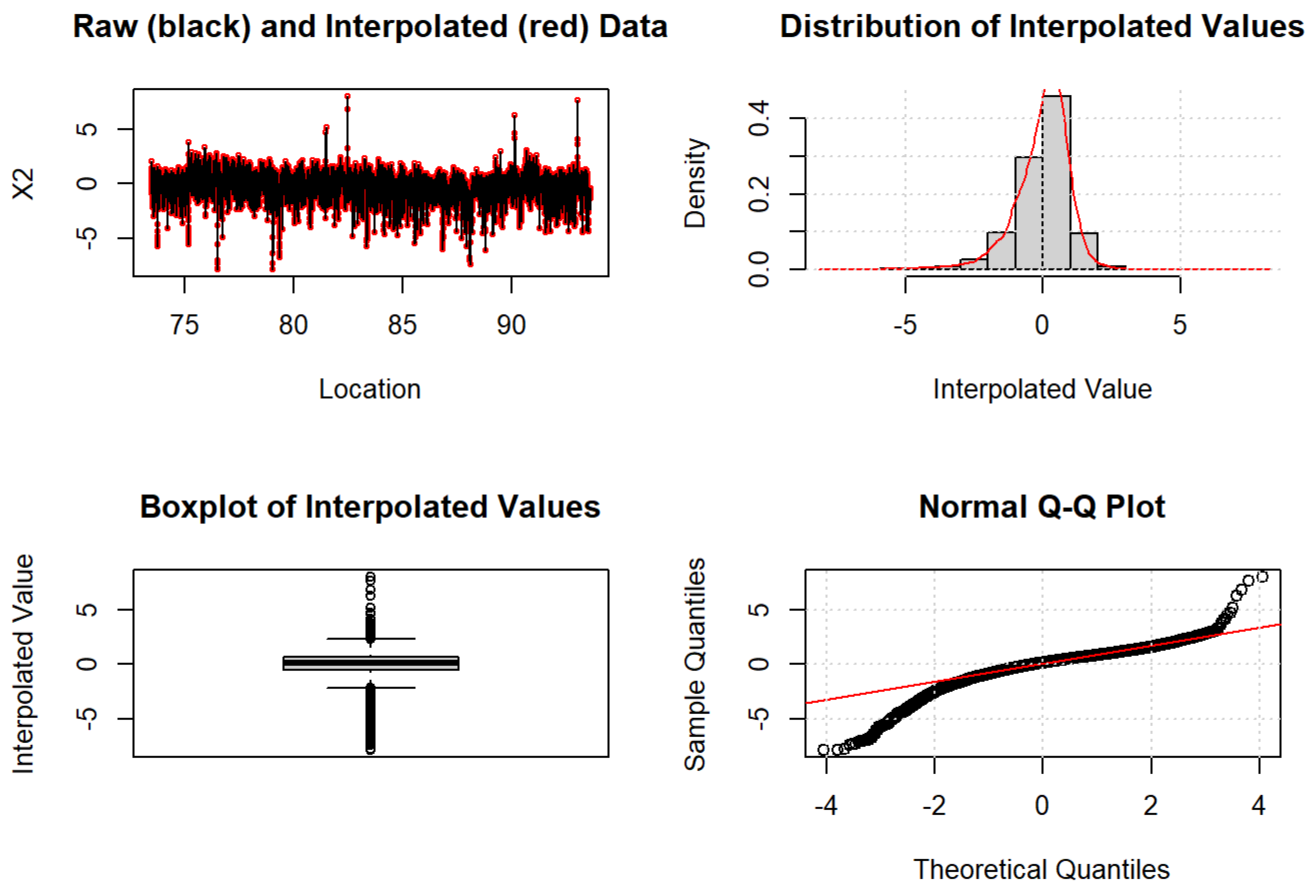


```
#Saving of the 1 mm (non resampled) Ti series
alb_Ti_1mm<-linterp(alb_Ti, genplot=T)
```

```
----- APPLYING PIECEWISE-LINEAR INTERPOLATION TO STRATIGRAPHIC SERIES -----

 * Number of samples= 20158
 * Determining median sampling interval for series
 * Will interpolate to median sampling interval of 0.001
 * New number of samples= 20158
```

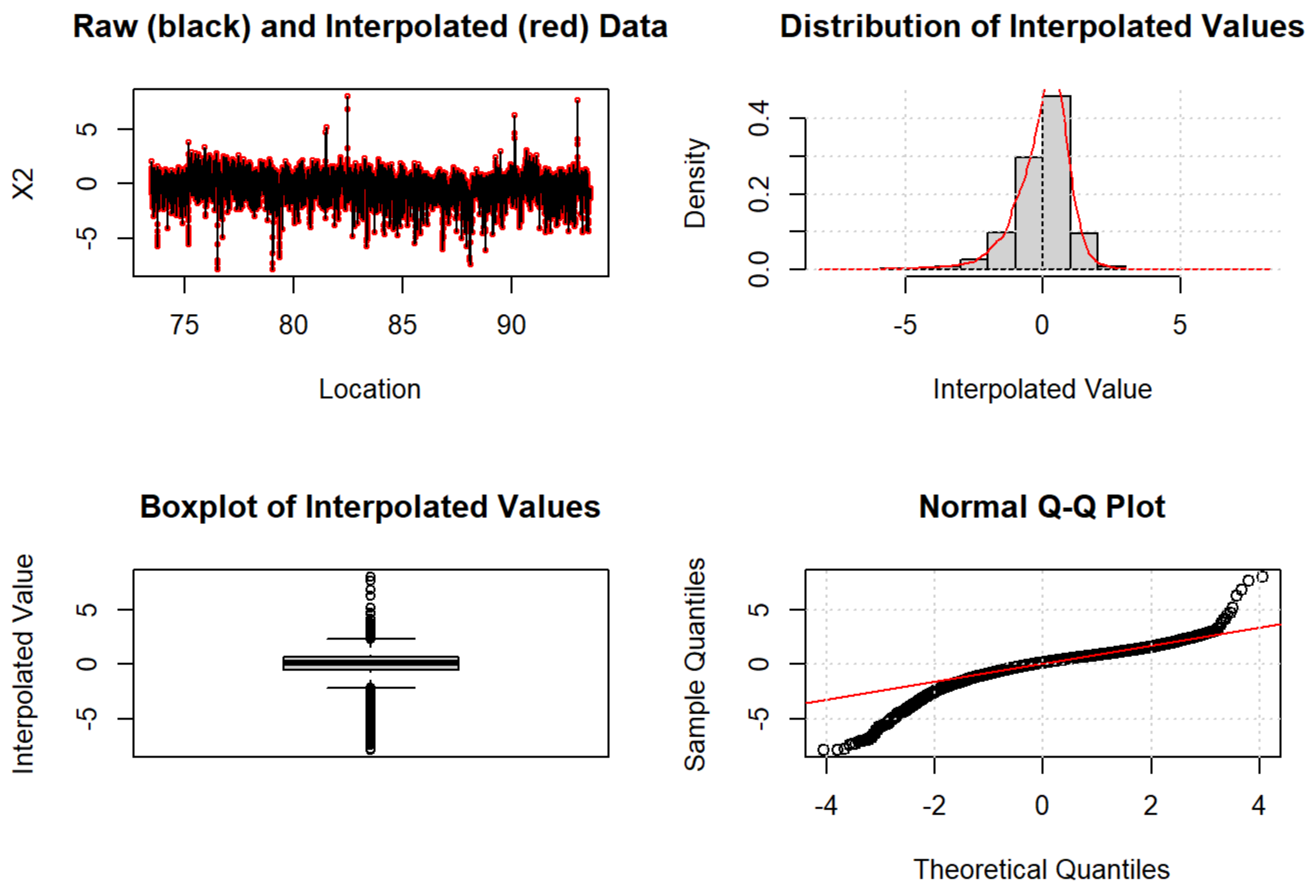


```
#Resampling of the Ti series every 5 mm
alb_Ti <-  linterp(alb_Ti, dt=0.005, genplot=T)
```

```
----- APPLYING PIECEWISE-LINEAR INTERPOLATION TO STRATIGRAPHIC SERIES -----

 * Number of samples= 20158
 * New number of samples= 4032
```

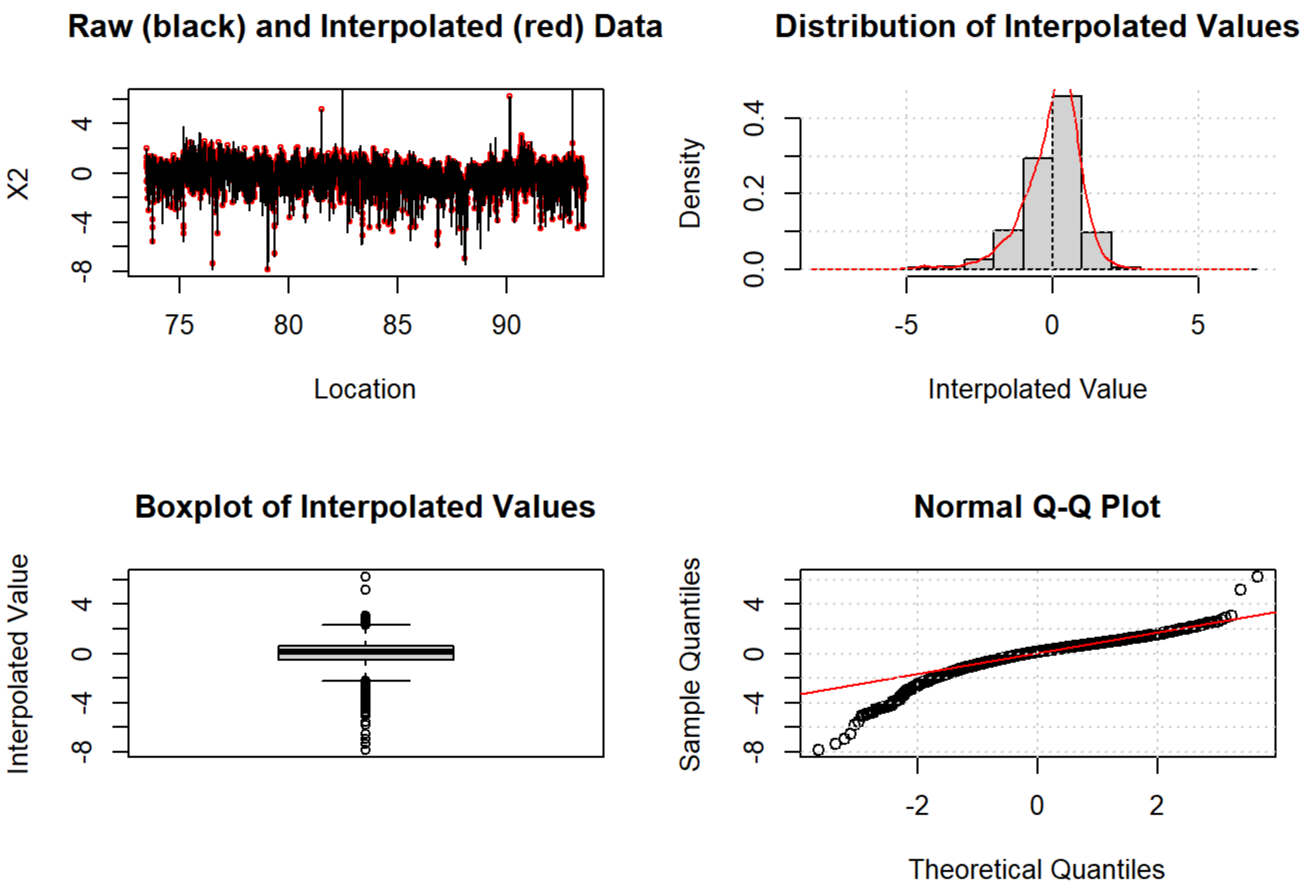


```
#Saving of the non detrended Ti series resampled every 5 mm
alb_Ti_ndet <-  linterp(alb_Ti, genplot=T)
```

```
----- APPLYING PIECEWISE-LINEAR INTERPOLATION TO STRATIGRAPHIC SERIES -----

 * Number of samples= 4032
 * Determining median sampling interval for series
 * Will interpolate to median sampling interval of 0.005
 * New number of samples= 4032
```

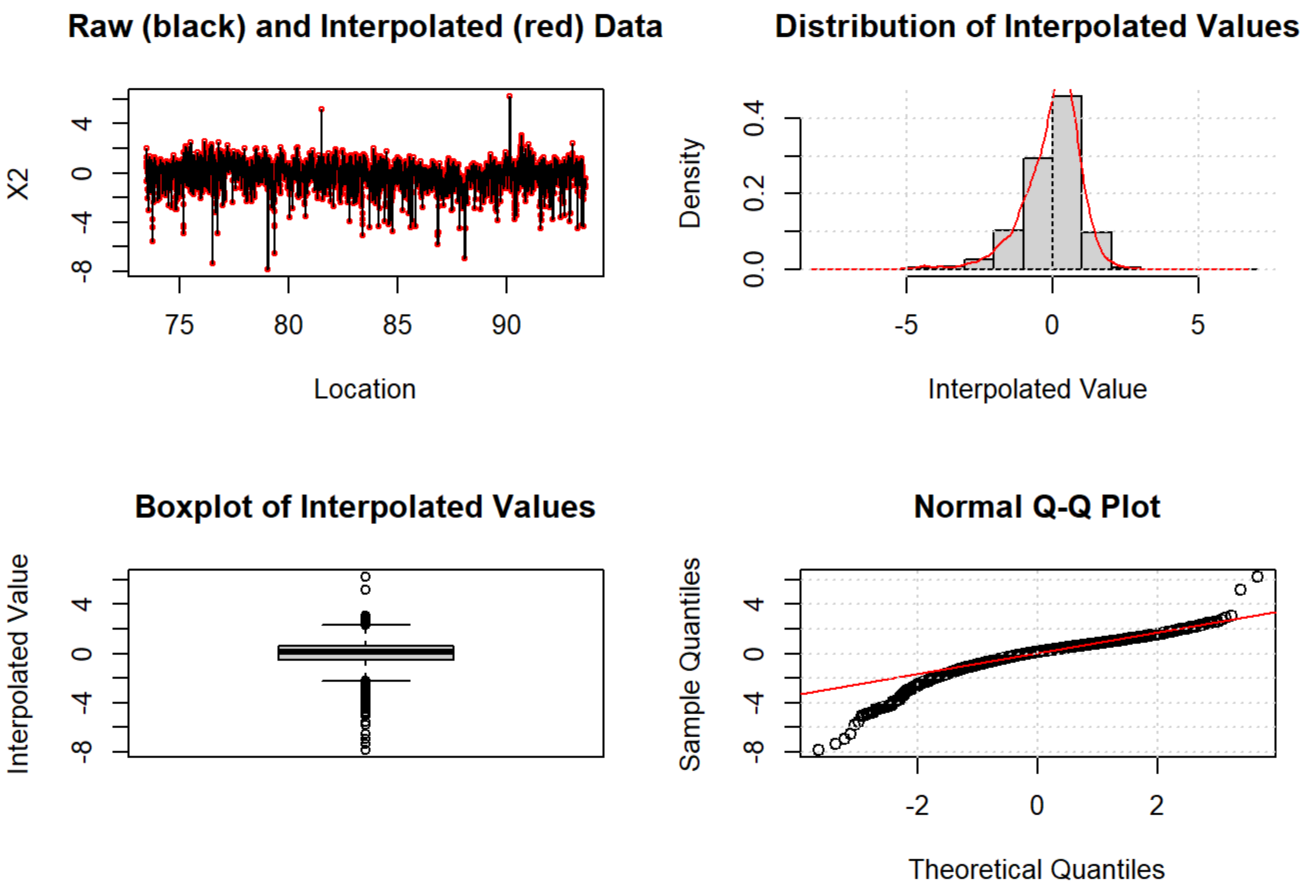


```r
#Plotting of the non detrended 5mm Ti series
plot(alb_Ti_ndet, type = "l", xlab = "Depth Adjusted (m)", ylab = "Ti Z score non-detrended")
```

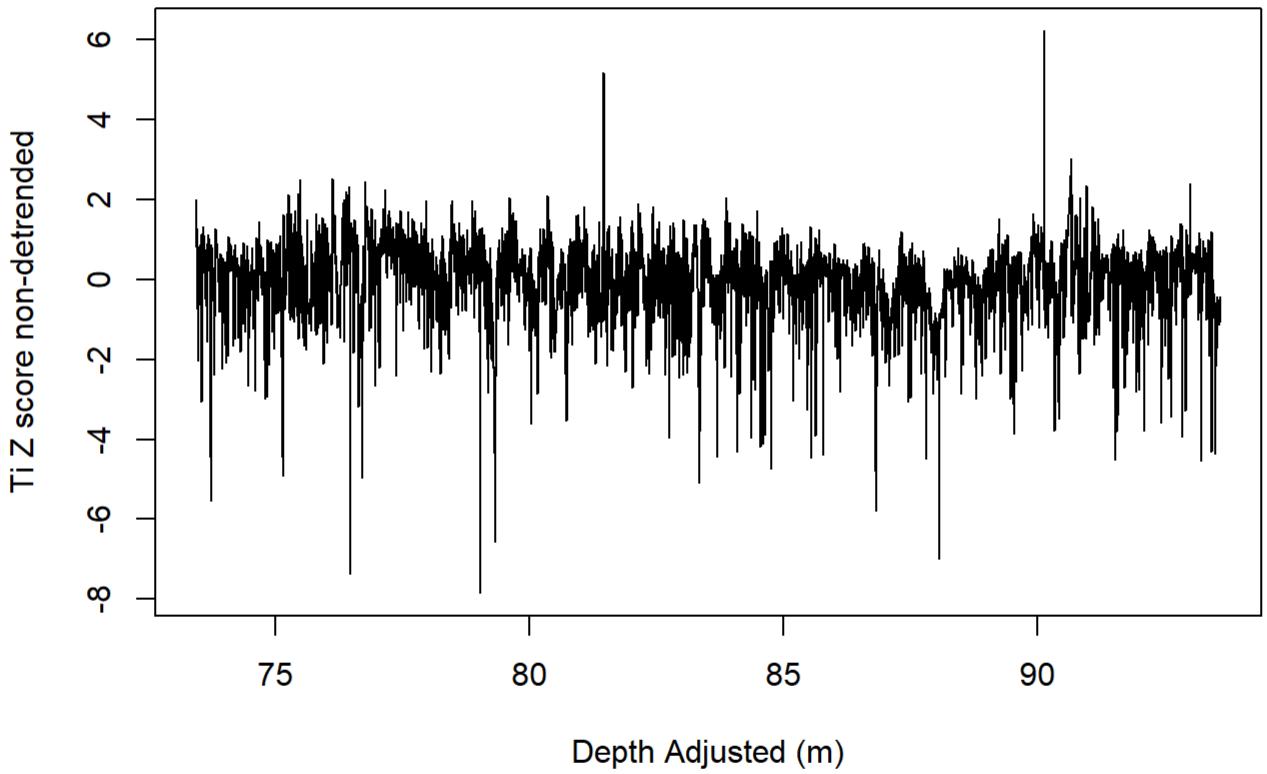


The Ti data have been selected, from the composite core, extending from 73.452 m (= anchoring depth with Zhao et al (2022b) dataset) to 93.609 m (= end of the Alum Shale Formation). Then the data are interpolated to ensure continuity of the Ti record every 5 mm. These data are not detrended and will be used in the Continuous Wavelet analysis (CWT).

## 1.2. Detrending of the Ti data

```
#Detrending (20% LOWESS) of the Ti series resampled every 5 mm
alb_Ti <- noLow(alb_Ti, smooth = 0.2, output = 1, genplot = T)
```

```
----- REMOVING LOWESS SMOOTHER FROM STRATIGRAPHIC SERIES -----
Call:
loess(formula = (dat[, 2]) ~ dat[, 1], span = smooth, degree = 1)

Number of Observations: 4032
Equivalent Number of Parameters: 8.17
Residual Standard Error: 1.015
Trace of smoother matrix: 9.68  (exact)

Control settings:
  span     :  0.2
  degree   :  1
  family   :  gaussian
  surface  :  interpolate      cell = 0.2
  normalize:  TRUE
```

```
 parametric:   FALSE
drop.square:   FALSE
```

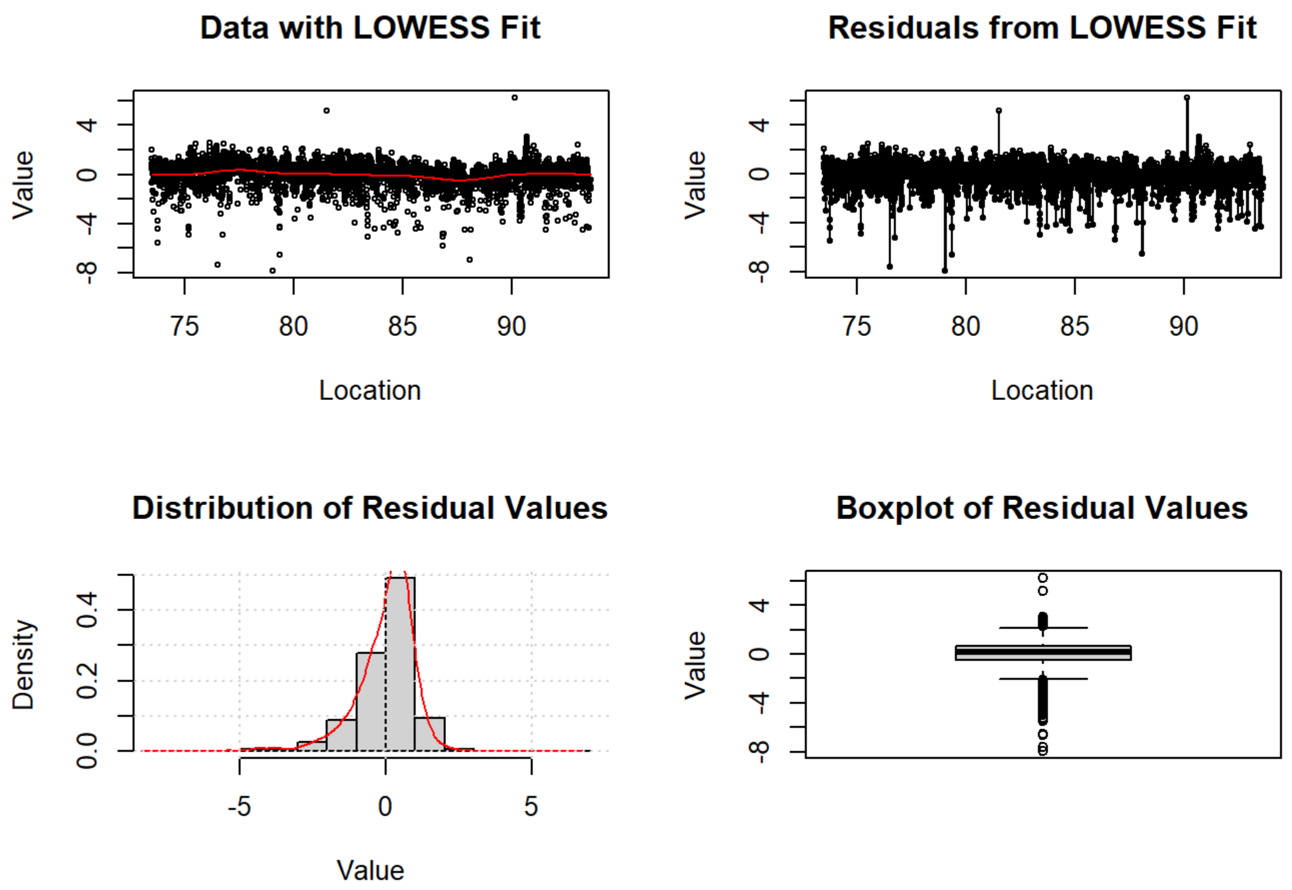


```
alb_Ti <- linterp(alb_Ti, genplot=T)
```

```
----- APPLYING PIECEWISE-LINEAR INTERPOLATION TO STRATIGRAPHIC SERIES -----

 * Number of samples= 4032
 * Determining median sampling interval for series
 * Will interpolate to median sampling interval of 0.005
 * New number of samples= 4032
```

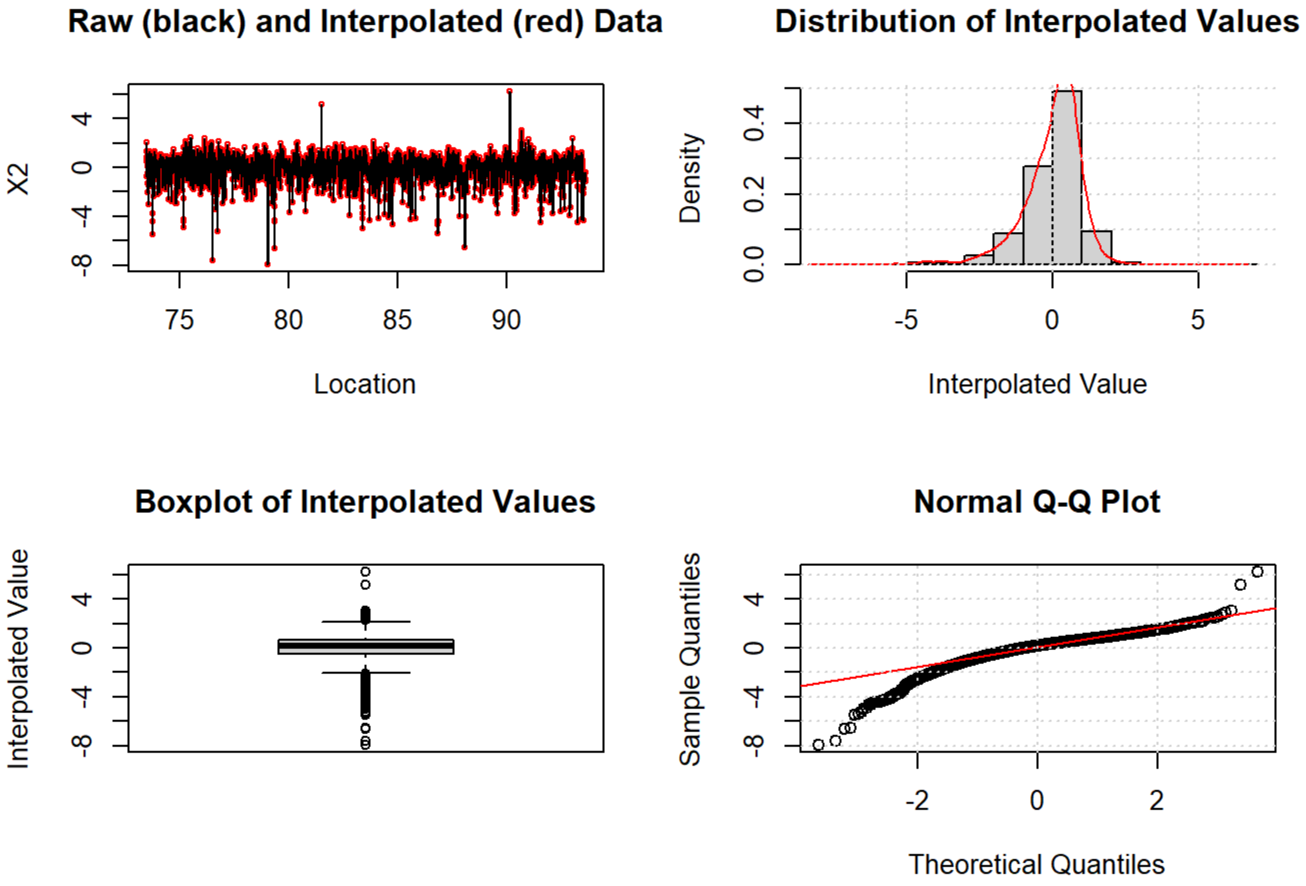


```r
plot(alb_Ti, type = "l", xlab = "Depth Adjusted (m)", ylab = "Ti Z score detrended")
```

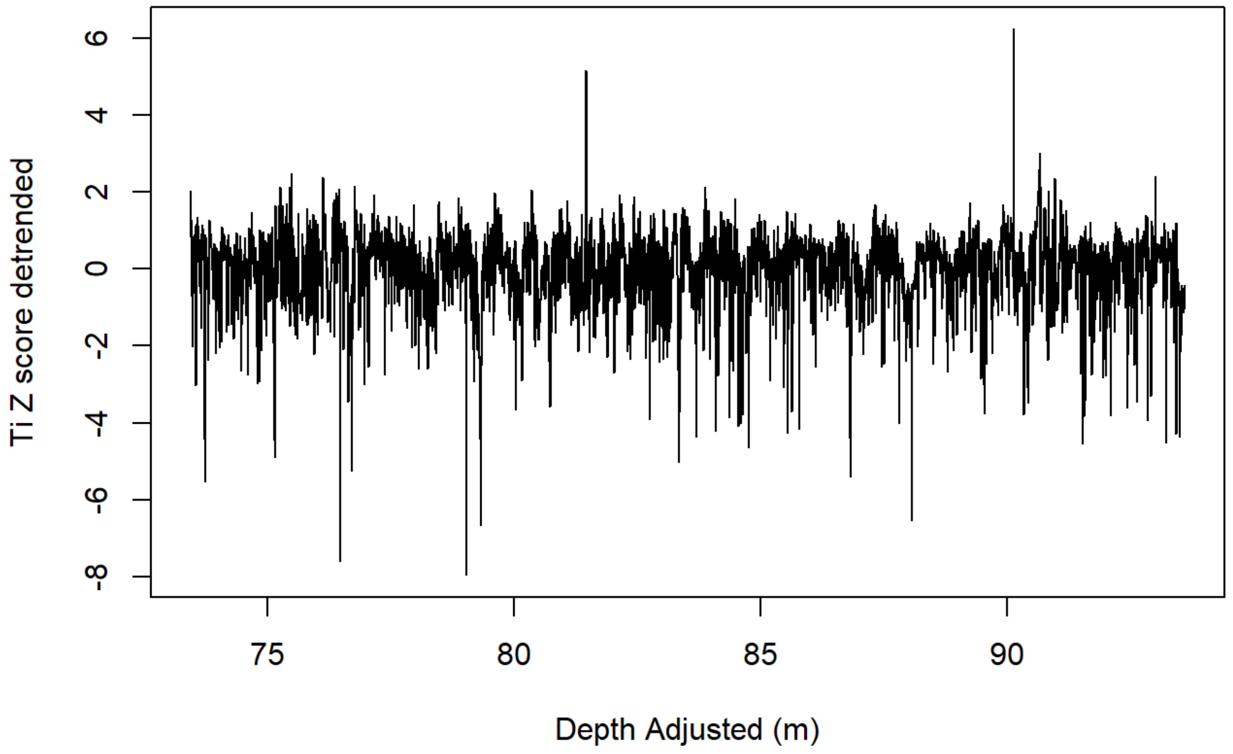

The 5mm spaced Ti series is detrended using a 20% LOWESS regression. These data will be used for step 2 to 10 of the protocol. The step 1 of the protocol was also performed for the other detrital elements (Al, Si, K and Zr)

---------------------------------------------------------------------------------------------------

# Step 2: MTM in depth domain

## 2.1. MTM on the non-detrened dataset

```
alb_Ti_MTM_ndet<-mtm(alb_Ti_ndet, xmax=15, siglevel = 0.9,ar1 = T, tbw= 2, output = 1)
```

```
----- PERFORMING Multitaper Spectral Analysis -----
 * Number of data points in stratigraphic series: 4032
 * Stratigraphic series length (space or time): 20.155
 * Sampling interval (space or time): 0.005
 * Will use default setting of 3 DPSS tapers
 * Mean value subtracted= -0.03819509
 * Linear trend NOT subtracted
 * Nyquist frequency: 100
 * Rayleigh frequency: 0.04960317
 * MTM Power spectrum bandwidth resolution (halfwidth): 0.09920635
 * Padded to 20160 points
 * Estimated AR1 coefficient = 0.4678053

 * Searching for significant spectral peaks that satisfy 90 % CL
    requirements outlined in Meyers (2012):
 * Number of significant F-test peaks identified = 92
ID  / Frequency / Period / Harmonic_CL / Rednoise_CL
1   0.04960317   20.16   99.59413   100
2   0.1488095   6.72   90.52508   99.99988
3   0.2380952   4.2   95.77769   99.63641
4   0.7936508   1.26   99.6483   99.72414
5   0.9126984   1.095652   99.90369   99.76809
6   1.150794   0.8689655   99.02863   99.96726
7   1.25   0.8   98.28226   100
8   1.359127   0.7357664   95.51838   98.69361
9   1.458333   0.6857143   98.10039   97.33287
10   1.547619   0.6461538   93.83247   94.56589
11   1.646825   0.6072289   98.11976   95.44981
12   1.746032   0.5727273   98.2977   98.37743
13   1.964286   0.5090909   96.41317   89.16807
14   2.093254   0.4777251   98.02182   61.40152
15   2.261905   0.4421053   98.61392   94.04487
16   2.678571   0.3733333   90.52717   87.76271
17   2.81746   0.3549296   90.21642   89.00479
18   2.956349   0.338255   99.80688   97.86445
19   3.333333   0.3   95.41258   94.92955
20   3.44246   0.2904899   94.96416   72.0446
21   4.236111   0.2360656   90.59066   92.04306
```

| | | | | |
|---|---|---|---|---|
| 22 | 5.228175 | 0.1912713 | 90.0769 | 95.19748 |
| 23 | 5.704365 | 0.1753043 | 97.37866 | 82.19038 |
| 24 | 5.853175 | 0.1708475 | 99.11178 | 61.88645 |
| 25 | 7.103175 | 0.1407821 | 98.71909 | 58.91435 |
| 26 | 7.202381 | 0.138843 | 93.36762 | 93.5992 |
| 27 | 12.5496 | 0.07968379 | 98.55084 | 64.87143 |
| 28 | 12.65873 | 0.07899687 | 92.19144 | 77.36012 |
| 29 | 25 | 0.04 | 97.3433 | 98.71976 |
| 30 | 28.75 | 0.03478261 | 99.35968 | 81.04829 |
| 31 | 36.37897 | 0.02748841 | 93.34859 | 66.00714 |
| 32 | 36.49802 | 0.02739875 | 96.30149 | 67.03294 |
| 33 | 43.29365 | 0.02309808 | 99.42692 | 86.20213 |
| 34 | 44.6627 | 0.02239005 | 91.44214 | 93.32972 |
| 35 | 50.90278 | 0.01964529 | 94.31977 | 88.83037 |
| 36 | 53.5119 | 0.01868743 | 95.83838 | 91.38573 |
| 37 | 54.4246 | 0.01837404 | 93.35112 | 99.1812 |
| 38 | 56.4881 | 0.01770285 | 97.75266 | 89.76485 |
| 39 | 57.39087 | 0.01742437 | 90.87104 | 57.77427 |
| 40 | 57.60913 | 0.01735836 | 94.16765 | 73.1126 |
| 41 | 57.95635 | 0.01725436 | 98.8449 | 88.00713 |
| 42 | 58.11508 | 0.01720724 | 97.76301 | 96.51954 |
| 43 | 58.22421 | 0.01717499 | 96.76889 | 79.12464 |
| 44 | 59.31548 | 0.01685901 | 92.31114 | 83.25253 |
| 45 | 59.51389 | 0.0168028 | 92.95096 | 83.08796 |
| 46 | 60.66468 | 0.01648406 | 95.20887 | 97.91906 |
| 47 | 61.06151 | 0.01637693 | 97.26079 | 59.79055 |
| 48 | 61.21032 | 0.01633712 | 96.41056 | 70.3925 |
| 49 | 61.33929 | 0.01630277 | 99.90043 | 91.64441 |
| 50 | 61.43849 | 0.01627644 | 97.27363 | 93.0498 |
| 51 | 64.62302 | 0.01547436 | 92.17849 | 96.06461 |
| 52 | 65.87302 | 0.01518072 | 94.95824 | 61.38345 |
| 53 | 67.86706 | 0.01473469 | 91.52643 | 98.95389 |
| 54 | 67.98611 | 0.01470889 | 96.36932 | 91.70161 |
| 55 | 70.82341 | 0.01411962 | 92.4982 | 93.21737 |
| 56 | 71.00198 | 0.01408411 | 95.82177 | 80.20875 |
| 57 | 73.15476 | 0.01366965 | 92.54873 | 73.90308 |
| 58 | 74.89087 | 0.01335276 | 99.1105 | 95.01699 |
| 59 | 75.77381 | 0.01319717 | 98.99207 | 97.37065 |
| 60 | 77.07341 | 0.01297464 | 97.10868 | 73.67353 |
| 61 | 78.65079 | 0.01271443 | 93.08389 | 96.35031 |
| 62 | 79.89087 | 0.01251707 | 98.51305 | 88.55408 |
| 63 | 80.17857 | 0.01247216 | 96.8899 | 84.18897 |
| 64 | 80.60516 | 0.01240615 | 96.57845 | 85.75422 |
| 65 | 80.77381 | 0.01238025 | 94.00894 | 81.35132 |
| 66 | 81.875 | 0.01221374 | 94.37419 | 65.41283 |
| 67 | 81.99405 | 0.01219601 | 97.83034 | 63.22457 |
| 68 | 82.2123 | 0.01216363 | 99.6475 | 74.23975 |
| 69 | 82.33135 | 0.01214604 | 91.42259 | 88.0338 |
| 70 | 82.61905 | 0.01210375 | 94.18016 | 99.50291 |
| 71 | 83.80952 | 0.01193182 | 92.76588 | 98.59736 |
| 72 | 84.59325 | 0.01182127 | 94.44594 | 96.96859 |
| 73 | 85.5754 | 0.0116856 | 96.13289 | 58.66473 |
| 74 | 86.8254 | 0.01151737 | 93.10324 | 99.01141 |
| 75 | 87.14286 | 0.01147541 | 94.39685 | 85.69028 |

```
76   87.29167   0.01145585   95.07262   72.84607
77   87.48016   0.01143116   93.38032   63.57838
78   88.10516   0.01135007   97.96073   99.82218
79   89.33532   0.01119378   91.81732   96.05213
80   91.25992   0.01095771   99.14639   82.01627
81   91.51786   0.01092683   99.20717   98.70252
82   91.72619   0.01090201   99.51691   94.60788
83   93.03571   0.01074856   99.57182   86.93861
84   93.64087   0.0106791   92.99375   96.96892
85   93.95833   0.01064302   94.8374   98.20012
86   94.09722   0.01062731   94.58227   95.31293
87   94.40476   0.01059269   90.78054   79.38122
88   95.13889   0.01051095   93.32935   94.5039
89   95.50595   0.01047055   90.6481   96.25297
90   97.66865   0.0102387   97.14167   72.0247
91   98.90873   0.01011033   90.62833   59.48612
92   99.00794   0.0101002   90.26233   79.78084
```

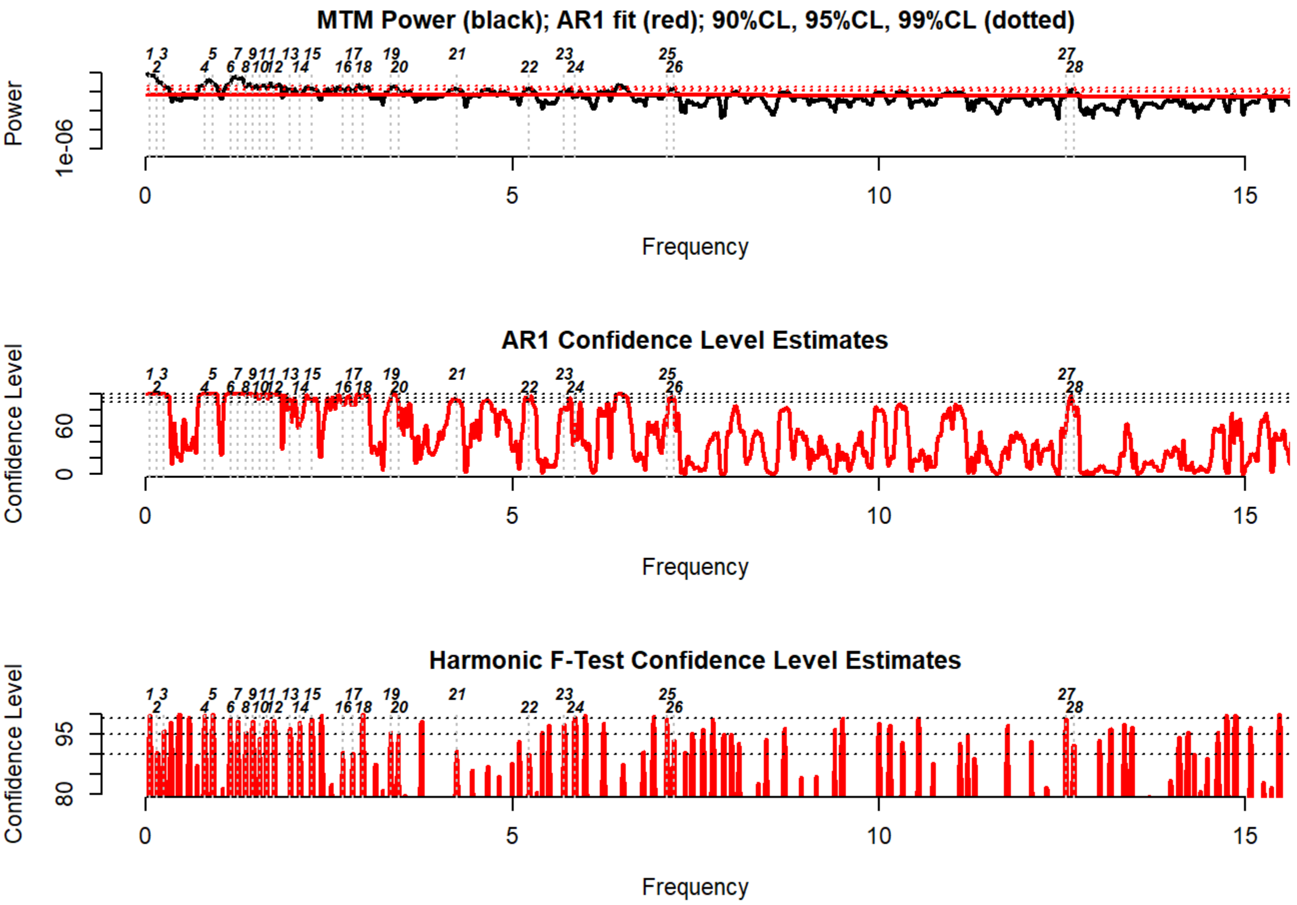


```
alb_Ti_MTM_ndet <- iso(dat=alb_Ti_MTM_ndet,xmin=0,xmax=15, genplot=F)
```

```
----- ISOLATE STRATIGRAPHIC DATA BY LOCATION -----
 * Number of data points= 10079
 * Number of columns= 8
 * Minimum= 0.009920635 , Maximum= 99.99008
 * Isolating data between 0 and 15
 * Number of data points following culling= 1511
```

```
plot(alb_Ti_MTM_ndet$Frequency,
     alb_Ti_MTM_ndet$Power,
     type = 'l',
     xlab = "Frequency (cycles/m)",
     ylab = "Variance"
     )

lines(alb_Ti_MTM_ndet$Frequency,
      alb_Ti_MTM_ndet$AR1_fit,
      type = 'l',
      col = 'black',
      lwd = 2
      )

lines(alb_Ti_MTM_ndet$Frequency,
      alb_Ti_MTM_ndet$AR1_90_power,
      type = 'l',
      col = 'red',
      lwd = 2
      )

lines(alb_Ti_MTM_ndet$Frequency,
      alb_Ti_MTM_ndet$AR1_95_power,
      type = 'l',
      col = 'blue',
      lwd = 2
      )

lines(alb_Ti_MTM_ndet$Frequency,
      alb_Ti_MTM_ndet$AR1_99_power,
      type = 'l',
      col = 'green',
      lwd = 2
      )
```

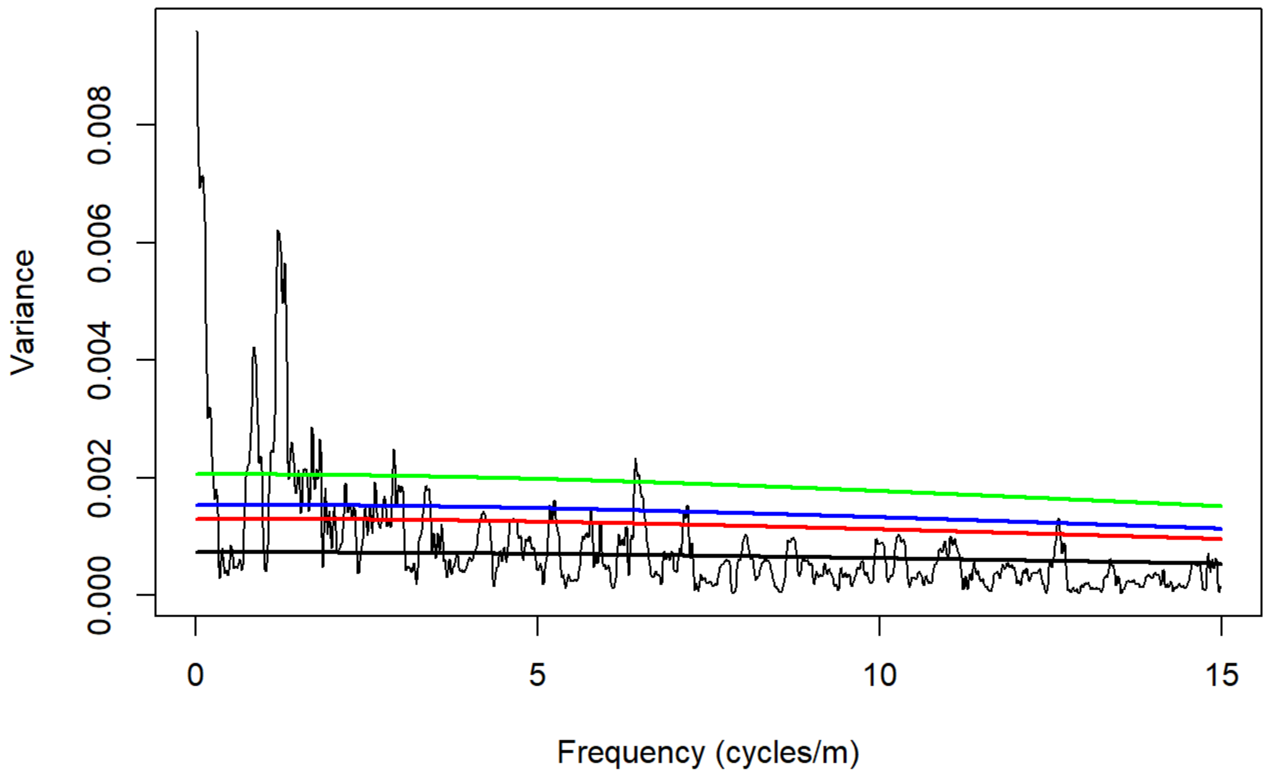


The 2 pi-MTM on the non-detrended data is performed to show the entire range of cyclicities in the signal. This MTM is not used afterward.

## 2.2. MTM on the detrended dataset

```
alb_Ti_MTM <- mtm(alb_Ti, xmax=15, siglevel = 0.9, ar1 = T, tbw= 2, output = 1)
```

```
----- PERFORMING Multitaper Spectral Analysis -----
 * Number of data points in stratigraphic series: 4032
 * Stratigraphic series length (space or time): 20.155
 * Sampling interval (space or time): 0.005
 * Will use default setting of 3 DPSS tapers
 * Mean value subtracted= 4.069401e-05
 * Linear trend NOT subtracted
 * Nyquist frequency: 100
 * Rayleigh frequency: 0.04960317
 * MTM Power spectrum bandwidth resolution (halfwidth): 0.09920635
 * Padded to 20160 points
 * Estimated AR1 coefficient = 0.4427859

 * Searching for significant spectral peaks that satisfy 90 % CL
    requirements outlined in Meyers (2012):
 * Number of significant F-test peaks identified = 94
ID  / Frequency / Period / Harmonic_CL / Rednoise_CL
1   0.7936508   1.26   99.6495   99.9071
```

| | | | | |
|---|---|---|---|---|
| 2 | 0.9126984 | 1.095652 | 99.90678 | 99.90939 |
| 3 | 1.150794 | 0.8689655 | 99.05213 | 99.99092 |
| 4 | 1.25 | 0.8 | 98.30687 | 100 |
| 5 | 1.359127 | 0.7357664 | 95.50549 | 99.41661 |
| 6 | 1.458333 | 0.6857143 | 98.12654 | 98.55564 |
| 7 | 1.547619 | 0.6461538 | 93.79633 | 96.96442 |
| 8 | 1.646825 | 0.6072289 | 98.12533 | 97.42249 |
| 9 | 1.746032 | 0.5727273 | 98.30366 | 99.24096 |
| 10 | 1.964286 | 0.5090909 | 96.34805 | 93.20886 |
| 11 | 2.093254 | 0.4777251 | 97.98995 | 68.89833 |
| 12 | 2.261905 | 0.4421053 | 98.62524 | 96.54462 |
| 13 | 2.678571 | 0.3733333 | 90.55385 | 91.9462 |
| 14 | 2.81746 | 0.3549296 | 90.19723 | 92.8981 |
| 15 | 2.956349 | 0.338255 | 99.80865 | 98.932 |
| 16 | 3.333333 | 0.3 | 95.41094 | 97.09887 |
| 17 | 3.44246 | 0.2904899 | 94.96645 | 78.86869 |
| 18 | 4.236111 | 0.2360656 | 90.58225 | 95.06536 |
| 19 | 5.228175 | 0.1912713 | 90.09819 | 97.21911 |
| 20 | 5.704365 | 0.1753043 | 97.37723 | 87.29627 |
| 21 | 5.853175 | 0.1708475 | 99.11158 | 69.01658 |
| 22 | 6.240079 | 0.1602544 | 97.67422 | 84.33962 |
| 23 | 7.103175 | 0.1407821 | 98.72186 | 65.90099 |
| 24 | 7.202381 | 0.138843 | 93.3723 | 96.0572 |
| 25 | 10.31746 | 0.09692308 | 92.89927 | 86.07821 |
| 26 | 11.10119 | 0.09008043 | 92.65311 | 88.88133 |
| 27 | 12.5496 | 0.07968379 | 98.55134 | 70.54842 |
| 28 | 12.65873 | 0.07899687 | 92.1856 | 82.09713 |
| 29 | 17.09325 | 0.05850261 | 94.11179 | 68.76633 |
| 30 | 17.8373 | 0.05606229 | 90.02154 | 88.05182 |
| 31 | 25 | 0.04 | 97.34224 | 99.05331 |
| 32 | 28.75 | 0.03478261 | 99.3598 | 82.94557 |
| 33 | 36.37897 | 0.02748841 | 93.34558 | 67.44022 |
| 34 | 36.49802 | 0.02739875 | 96.30253 | 68.38105 |
| 35 | 43.29365 | 0.02309808 | 99.42679 | 86.71654 |
| 36 | 44.6627 | 0.02239005 | 91.43392 | 93.59203 |
| 37 | 50.90278 | 0.01964529 | 94.31732 | 88.98035 |
| 38 | 53.5119 | 0.01868743 | 95.83561 | 91.34791 |
| 39 | 54.4246 | 0.01837404 | 93.35017 | 99.19354 |
| 40 | 56.4881 | 0.01770285 | 97.75355 | 89.75403 |
| 41 | 57.39087 | 0.01742437 | 90.87208 | 57.797 |
| 42 | 57.60913 | 0.01735836 | 94.16682 | 73.16123 |
| 43 | 57.95635 | 0.01725436 | 98.8438 | 87.94543 |
| 44 | 58.11508 | 0.01720724 | 97.76266 | 96.49958 |
| 45 | 58.22421 | 0.01717499 | 96.77038 | 79.02898 |
| 46 | 59.31548 | 0.01685901 | 92.31666 | 83.12247 |
| 47 | 59.51389 | 0.0168028 | 92.95351 | 82.93598 |
| 48 | 60.66468 | 0.01648406 | 95.20807 | 97.90035 |
| 49 | 61.06151 | 0.01637693 | 97.25952 | 59.65102 |
| 50 | 61.21032 | 0.01633712 | 96.41152 | 70.29164 |
| 51 | 61.33929 | 0.01630277 | 99.90038 | 91.56283 |
| 52 | 61.43849 | 0.01627644 | 97.2732 | 92.98992 |
| 53 | 64.62302 | 0.01547436 | 92.18034 | 95.99514 |
| 54 | 65.87302 | 0.01518072 | 94.95728 | 60.97955 |
| 55 | 67.86706 | 0.01473469 | 91.5305 | 98.91116 |

| | | | | |
|---|---|---|---|---|
| 56 | 67.98611 | 0.01470889 | 96.37001 | 91.48216 |
| 57 | 70.82341 | 0.01411962 | 92.50066 | 93.04393 |
| 58 | 71.00198 | 0.01408411 | 95.82738 | 79.77178 |
| 59 | 73.15476 | 0.01366965 | 92.5475 | 73.40234 |
| 60 | 74.89087 | 0.01335276 | 99.11115 | 94.81942 |
| 61 | 75.77381 | 0.01319717 | 98.99135 | 97.25962 |
| 62 | 77.07341 | 0.01297464 | 97.1098 | 73.18008 |
| 63 | 78.65079 | 0.01271443 | 93.09069 | 96.18004 |
| 64 | 79.89087 | 0.01251707 | 98.51331 | 88.16902 |
| 65 | 80.17857 | 0.01247216 | 96.89047 | 83.7865 |
| 66 | 80.60516 | 0.01240615 | 96.57814 | 85.30011 |
| 67 | 80.77381 | 0.01238025 | 94.01293 | 80.79683 |
| 68 | 81.875 | 0.01221374 | 94.37741 | 64.65222 |
| 69 | 81.99405 | 0.01219601 | 97.83026 | 62.50613 |
| 70 | 82.2123 | 0.01216363 | 99.64703 | 73.589 |
| 71 | 82.33135 | 0.01214604 | 91.42229 | 87.60278 |
| 72 | 82.61905 | 0.01210375 | 94.18218 | 99.46475 |
| 73 | 83.80952 | 0.01193182 | 92.76434 | 98.51632 |
| 74 | 84.59325 | 0.01182127 | 94.4466 | 96.81177 |
| 75 | 85.5754 | 0.0116856 | 96.13613 | 57.87317 |
| 76 | 86.8254 | 0.01151737 | 93.10284 | 98.94794 |
| 77 | 87.14286 | 0.01147541 | 94.39533 | 85.17613 |
| 78 | 87.29167 | 0.01145585 | 95.06977 | 72.27986 |
| 79 | 87.48016 | 0.01143116 | 93.37054 | 62.7484 |
| 80 | 88.10516 | 0.01135007 | 97.95976 | 99.80612 |
| 81 | 89.33532 | 0.01119378 | 91.8167 | 95.82547 |
| 82 | 91.25992 | 0.01095771 | 99.14693 | 81.4381 |
| 83 | 91.51786 | 0.01092683 | 99.20682 | 98.60804 |
| 84 | 91.72619 | 0.01090201 | 99.51717 | 94.34934 |
| 85 | 93.03571 | 0.01074856 | 99.57157 | 86.42848 |
| 86 | 93.64087 | 0.0106791 | 92.99197 | 96.78454 |
| 87 | 93.95833 | 0.01064302 | 94.83938 | 98.07256 |
| 88 | 94.09722 | 0.01062731 | 94.58366 | 95.04817 |
| 89 | 94.40476 | 0.01059269 | 90.77981 | 78.9516 |
| 90 | 95.13889 | 0.01051095 | 93.32958 | 94.27396 |
| 91 | 95.50595 | 0.01047055 | 90.64873 | 96.05761 |
| 92 | 97.66865 | 0.0102387 | 97.14153 | 71.41045 |
| 93 | 98.90873 | 0.01011033 | 90.6295 | 58.61966 |
| 94 | 99.00794 | 0.0101002 | 90.26207 | 79.29464 |

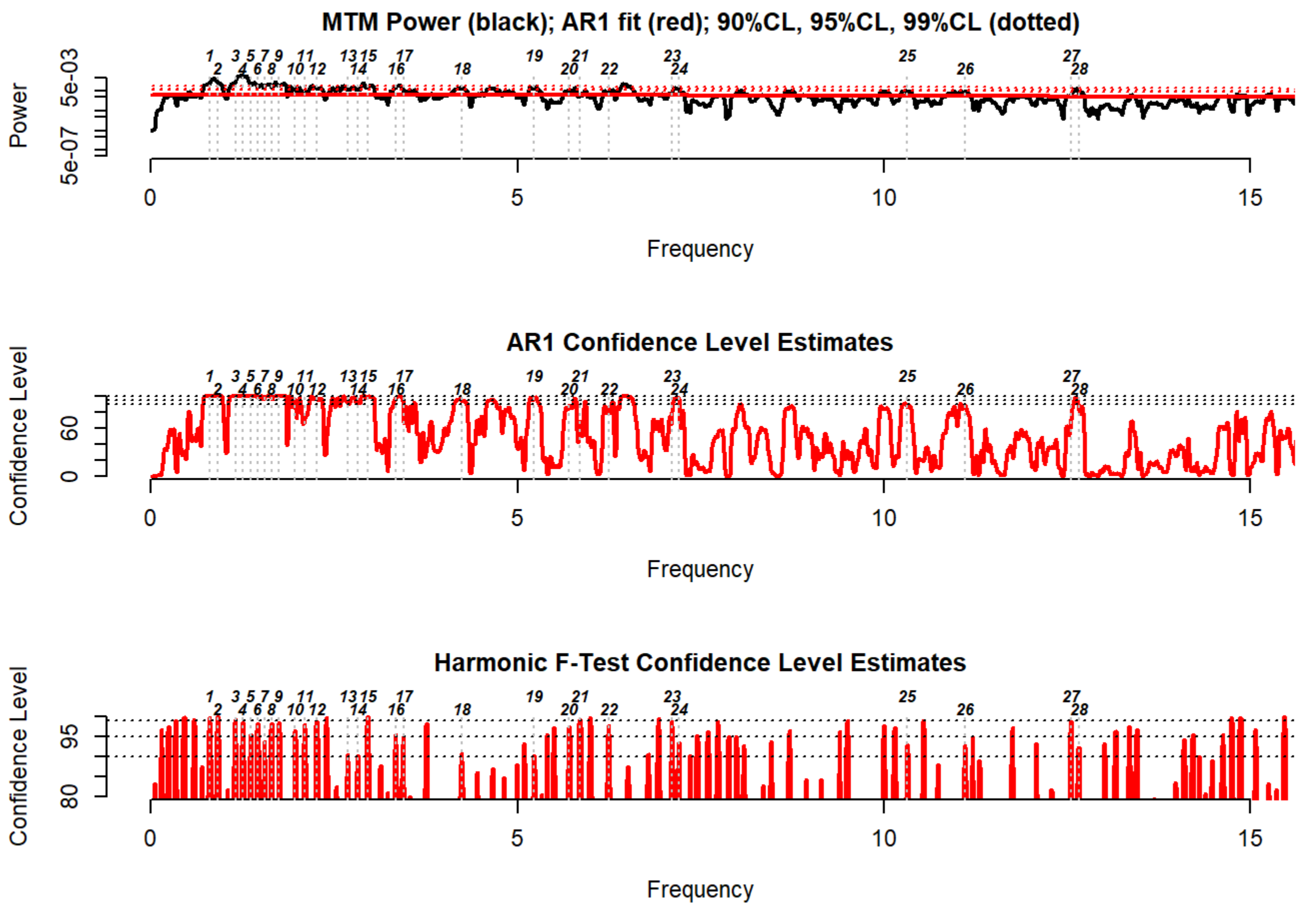


```
alb_Ti_MTM <- iso(dat=alb_Ti_MTM, xmin=0, xmax=15, genplot=F)
```

```
----- ISOLATE STRATIGRAPHIC DATA BY LOCATION -----
 * Number of data points= 10079
 * Number of columns= 8
 * Minimum= 0.009920635 , Maximum= 99.99008
 * Isolating data between 0 and 15
 * Number of data points following culling= 1511
```

```
plot(alb_Ti_MTM$Frequency,
     alb_Ti_MTM$Power,
     type = 'l',
     xlab = "Frequency (cycles/m)",
     ylab = "Variance",
     )

lines(alb_Ti_MTM$Frequency,
      alb_Ti_MTM$AR1_fit,
      type = 'l',
      col = 'black',
      lwd = 2
      )

lines(alb_Ti_MTM$Frequency,
      alb_Ti_MTM$AR1_90_power,
      type = 'l',
```

```
       col = 'red',
       lwd = 2
       )

lines(alb_Ti_MTM$Frequency,
      alb_Ti_MTM$AR1_95_power,
       type = 'l',
       col = 'blue',
       lwd = 2
       )

lines(alb_Ti_MTM$Frequency,
      alb_Ti_MTM$AR1_99_power,
       type = 'l',
       col = 'green',
       lwd = 2
       )
```

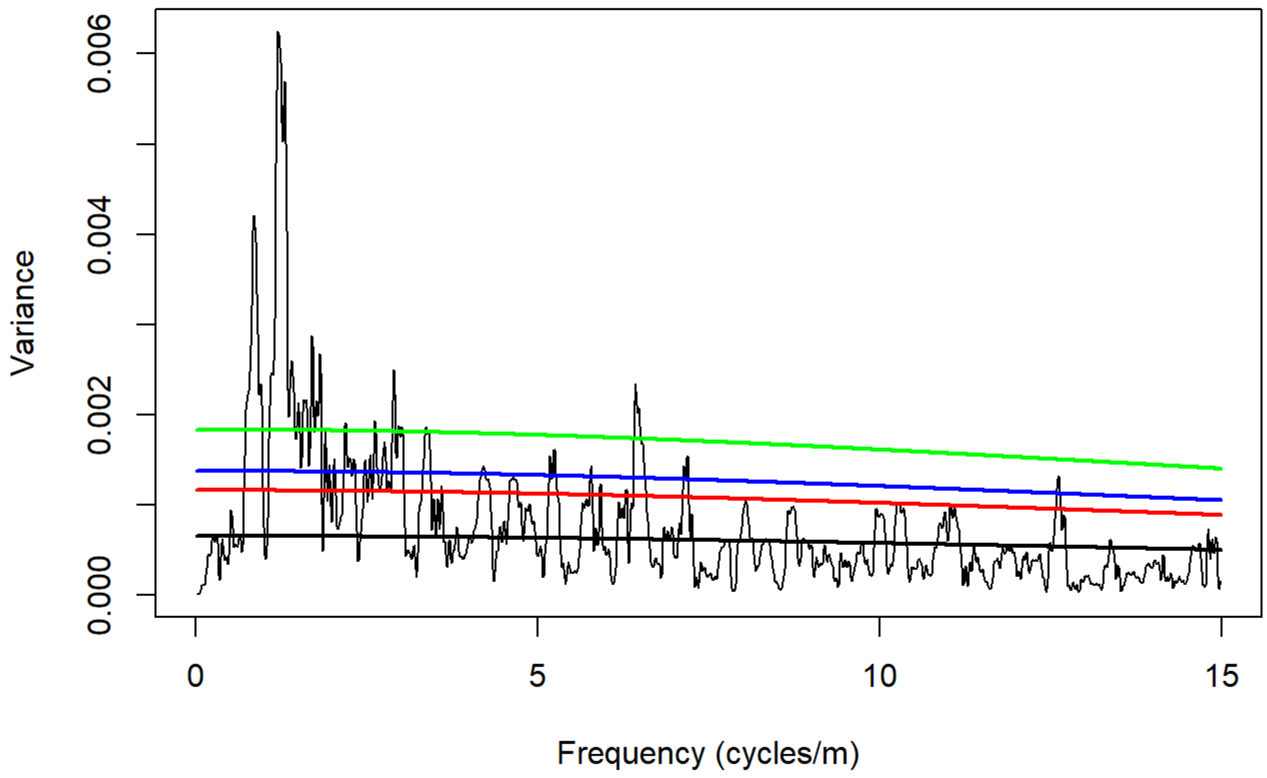


The 2 pi-MTM on the detrended data is performed to show the range of cyclicities in the detrended signal. Allowing to visually identify > 90 % CL periodicities.

## 2.3. Identification and extraction of the >90% CL periodicities

```
alb_Ti_MTM<-mtm(alb_Ti, xmax=15, siglevel = 0.9, ar1 = T, tbw= 2, output = 2)
```

```
----- PERFORMING Multitaper Spectral Analysis -----
 * Number of data points in stratigraphic series: 4032
 * Stratigraphic series length (space or time): 20.155
 * Sampling interval (space or time): 0.005
 * Will use default setting of 3 DPSS tapers
 * Mean value subtracted= 4.069401e-05
 * Linear trend NOT subtracted
 * Nyquist frequency: 100
 * Rayleigh frequency: 0.04960317
 * MTM Power spectrum bandwidth resolution (halfwidth): 0.09920635
 * Padded to 20160 points
 * Estimated AR1 coefficient = 0.4427859

 * Searching for significant spectral peaks that satisfy 90 % CL
     requirements outlined in Meyers (2012):
 * Number of significant F-test peaks identified = 94
ID  / Frequency / Period / Harmonic_CL / Rednoise_CL
1   0.7936508   1.26   99.6495   99.9071
2   0.9126984   1.095652   99.90678   99.90939
3   1.150794   0.8689655   99.05213   99.99092
4   1.25   0.8   98.30687   100
5   1.359127   0.7357664   95.50549   99.41661
6   1.458333   0.6857143   98.12654   98.55564
7   1.547619   0.6461538   93.79633   96.96442
8   1.646825   0.6072289   98.12533   97.42249
9   1.746032   0.5727273   98.30366   99.24096
10   1.964286   0.5090909   96.34805   93.20886
11   2.093254   0.4777251   97.98995   68.89833
12   2.261905   0.4421053   98.62524   96.54462
13   2.678571   0.3733333   90.55385   91.9462
14   2.81746   0.3549296   90.19723   92.8981
15   2.956349   0.338255   99.80865   98.932
16   3.333333   0.3   95.41094   97.09887
17   3.44246   0.2904899   94.96645   78.86869
18   4.236111   0.2360656   90.58225   95.06536
19   5.228175   0.1912713   90.09819   97.21911
20   5.704365   0.1753043   97.37723   87.29627
21   5.853175   0.1708475   99.11158   69.01658
22   6.240079   0.1602544   97.67422   84.33962
23   7.103175   0.1407821   98.72186   65.90099
24   7.202381   0.138843   93.3723   96.0572
25   10.31746   0.09692308   92.89927   86.07821
26   11.10119   0.09008043   92.65311   88.88133
27   12.5496   0.07968379   98.55134   70.54842
28   12.65873   0.07899687   92.1856   82.09713
29   17.09325   0.05850261   94.11179   68.76633
30   17.8373   0.05606229   90.02154   88.05182
31   25   0.04   97.34224   99.05331
32   28.75   0.03478261   99.3598   82.94557
33   36.37897   0.02748841   93.34558   67.44022
34   36.49802   0.02739875   96.30253   68.38105
35   43.29365   0.02309808   99.42679   86.71654
36   44.6627   0.02239005   91.43392   93.59203
```

| | | | | |
|---|---|---|---|---|
| 37 | 50.90278 | 0.01964529 | 94.31732 | 88.98035 |
| 38 | 53.5119 | 0.01868743 | 95.83561 | 91.34791 |
| 39 | 54.4246 | 0.01837404 | 93.35017 | 99.19354 |
| 40 | 56.4881 | 0.01770285 | 97.75355 | 89.75403 |
| 41 | 57.39087 | 0.01742437 | 90.87208 | 57.797 |
| 42 | 57.60913 | 0.01735836 | 94.16682 | 73.16123 |
| 43 | 57.95635 | 0.01725436 | 98.8438 | 87.94543 |
| 44 | 58.11508 | 0.01720724 | 97.76266 | 96.49958 |
| 45 | 58.22421 | 0.01717499 | 96.77038 | 79.02898 |
| 46 | 59.31548 | 0.01685901 | 92.31666 | 83.12247 |
| 47 | 59.51389 | 0.0168028 | 92.95351 | 82.93598 |
| 48 | 60.66468 | 0.01648406 | 95.20807 | 97.90035 |
| 49 | 61.06151 | 0.01637693 | 97.25952 | 59.65102 |
| 50 | 61.21032 | 0.01633712 | 96.41152 | 70.29164 |
| 51 | 61.33929 | 0.01630277 | 99.90038 | 91.56283 |
| 52 | 61.43849 | 0.01627644 | 97.2732 | 92.98992 |
| 53 | 64.62302 | 0.01547436 | 92.18034 | 95.99514 |
| 54 | 65.87302 | 0.01518072 | 94.95728 | 60.97955 |
| 55 | 67.86706 | 0.01473469 | 91.5305 | 98.91116 |
| 56 | 67.98611 | 0.01470889 | 96.37001 | 91.48216 |
| 57 | 70.82341 | 0.01411962 | 92.50066 | 93.04393 |
| 58 | 71.00198 | 0.01408411 | 95.82738 | 79.77178 |
| 59 | 73.15476 | 0.01366965 | 92.5475 | 73.40234 |
| 60 | 74.89087 | 0.01335276 | 99.11115 | 94.81942 |
| 61 | 75.77381 | 0.01319717 | 98.99135 | 97.25962 |
| 62 | 77.07341 | 0.01297464 | 97.1098 | 73.18008 |
| 63 | 78.65079 | 0.01271443 | 93.09069 | 96.18004 |
| 64 | 79.89087 | 0.01251707 | 98.51331 | 88.16902 |
| 65 | 80.17857 | 0.01247216 | 96.89047 | 83.7865 |
| 66 | 80.60516 | 0.01240615 | 96.57814 | 85.30011 |
| 67 | 80.77381 | 0.01238025 | 94.01293 | 80.79683 |
| 68 | 81.875 | 0.01221374 | 94.37741 | 64.65222 |
| 69 | 81.99405 | 0.01219601 | 97.83026 | 62.50613 |
| 70 | 82.2123 | 0.01216363 | 99.64703 | 73.589 |
| 71 | 82.33135 | 0.01214604 | 91.42229 | 87.60278 |
| 72 | 82.61905 | 0.01210375 | 94.18218 | 99.46475 |
| 73 | 83.80952 | 0.01193182 | 92.76434 | 98.51632 |
| 74 | 84.59325 | 0.01182127 | 94.4466 | 96.81177 |
| 75 | 85.5754 | 0.0116856 | 96.13613 | 57.87317 |
| 76 | 86.8254 | 0.01151737 | 93.10284 | 98.94794 |
| 77 | 87.14286 | 0.01147541 | 94.39533 | 85.17613 |
| 78 | 87.29167 | 0.01145585 | 95.06977 | 72.27986 |
| 79 | 87.48016 | 0.01143116 | 93.37054 | 62.7484 |
| 80 | 88.10516 | 0.01135007 | 97.95976 | 99.80612 |
| 81 | 89.33532 | 0.01119378 | 91.8167 | 95.82547 |
| 82 | 91.25992 | 0.01095771 | 99.14693 | 81.4381 |
| 83 | 91.51786 | 0.01092683 | 99.20682 | 98.60804 |
| 84 | 91.72619 | 0.01090201 | 99.51717 | 94.34934 |
| 85 | 93.03571 | 0.01074856 | 99.57157 | 86.42848 |
| 86 | 93.64087 | 0.0106791 | 92.99197 | 96.78454 |
| 87 | 93.95833 | 0.01064302 | 94.83938 | 98.07256 |
| 88 | 94.09722 | 0.01062731 | 94.58366 | 95.04817 |
| 89 | 94.40476 | 0.01059269 | 90.77981 | 78.9516 |
| 90 | 95.13889 | 0.01051095 | 93.32958 | 94.27396 |

```
91    95.50595    0.01047055    90.64873    96.05761
92    97.66865    0.0102387    97.14153    71.41045
93    98.90873    0.01011033    90.6295    58.61966
94    99.00794    0.0101002    90.26207    79.29464
```

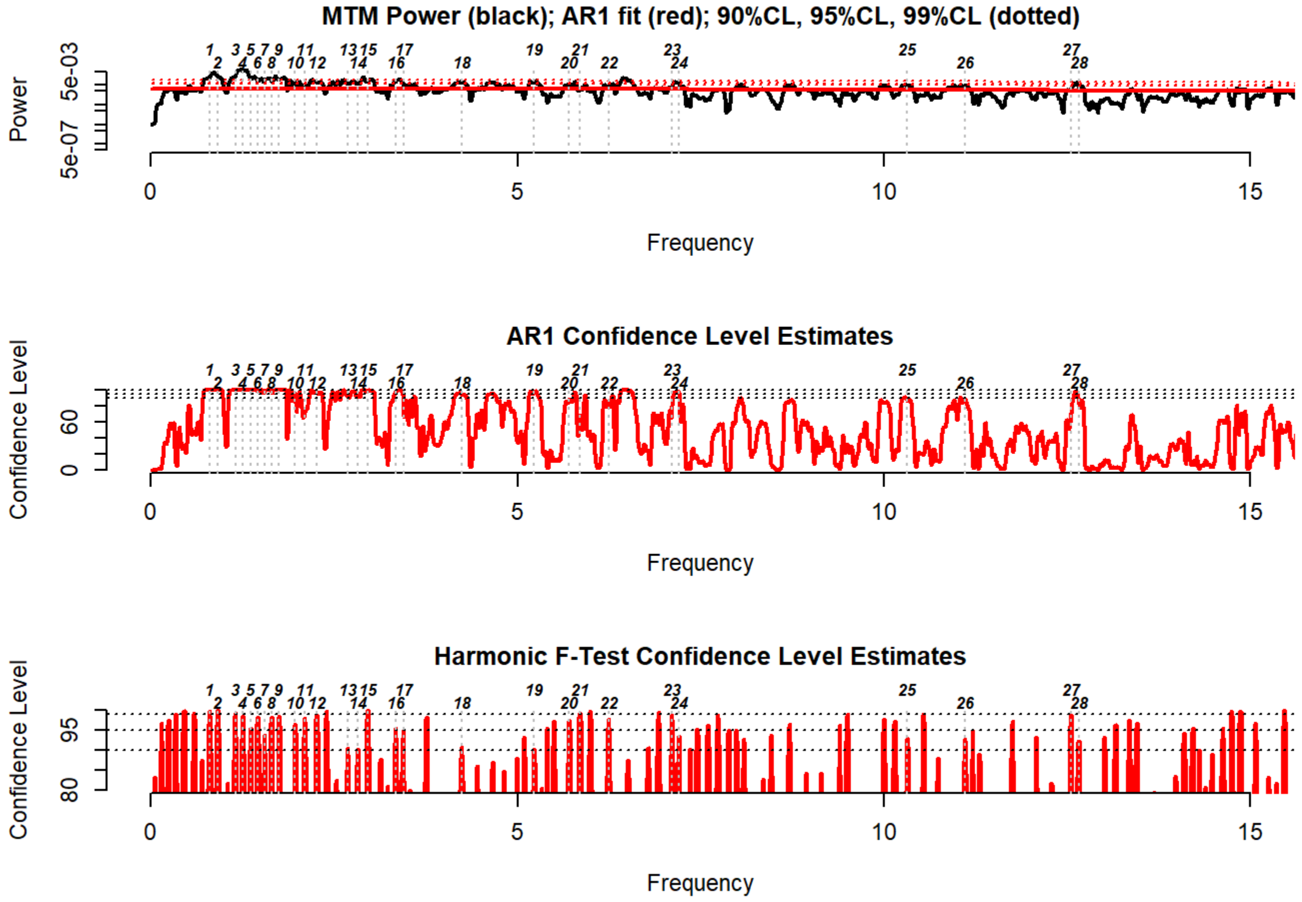


```r
alb_Ti_MTM <- iso(dat=alb_Ti_MTM, xmin=0, xmax=15, genplot=F)
```

```
----- ISOLATE STRATIGRAPHIC DATA BY LOCATION -----
 * Number of data points= 94
 * Number of columns= 1
 * Minimum= 0.7936508 , Maximum= 99.00794
 * Isolating data between 0 and 15
 * Number of data points following culling= 28
```

```r
write.csv(alb_Ti_MTM,"alb_Ti_sigfreq_MTM_rsp0.005_det0.20_Zscore_ACL.csv")
```

---

# Step 3: Ratio of frequencies

## 3.1. Ratio of the significant frequencies extracted from the MTM (in Excel)

The comparison between the ratios of the observed >90% CL and the theoretical ratio of the Milankovitch cycles is conducted in a separate Excel file. See Supplementary Materials 6 for more details.

Based on Zhao et al (2022b) paper, the 405 kyr cycle should be recorded between 1.75 and 2.25 m but nothing is visible in the Ti data (but is is observable within the other detrital elements). However, all the other frequency bands observed by Zhao et al (2022b), and corresponding to Milankovitch periodicities are identify in the Ti data.

In addition, a significant peak is located around 0.85 m where the 173-kyr long obliquity (Inclination metronome) should be identified and this explains why we focus our study on the 173 kyr metronome.

The duration of target cycles are recalculated from the literature (Laskar et al (2004); Laskar (2020); Wu et al (2024)).

-------------------------------------------------------------------------------------------------------

# Step 4: EHA and CWT

## 4.1. Evolutive Harmonic Analysis (EHA)

```
eha(alb_Ti,
    tbw=2,
    fmin=0.01,
    fmax=15,
    step=0.005,
    win=6,
    demean=T,
    detrend=T,
    siglevel=0.90,
    sigID=F,
    ydir=1,
    output=0,
    pl=1,
    palette=1,
    centerZero=T,
    ncolors=100,
    genplot=4,
    verbose=T
    )
```

```
----- PERFORMING EVOLUTIVE HARMONIC ANALYSIS -----
* Number of data points in stratigraphic series: 4032
* Stratigraphic series length (space or time): 20.155
* Sampling interval (space or time): 0.005
* Number of data points per window: 1201
* Moving window size (space or time): 6
* Window step points: 1
* Window step (space or time): 0.005
* Number of windows: 2832
```

```
 * Mean value for each window will be subtracted
 * Linear trend for each window will be subtracted
 * Nyquist frequency: 100
 * Rayleigh frequency: 0.1665279
 * MTM Power spectrum bandwidth resolution (halfwidth): 0.3330558
 * Will use 3 DPSS tapers
 * Padded to 4096 points
```

The EHA is performed on the Ti detrended series and confirms the presence of recurrent periodicities along the ACL as suggested by the > 90 % CL frequencies observed in the MTM

## 4.2. WaverideR (non-detrended data)

### 4.2.1. Continuous Wavelet Transform (CWT)

```
alb_Ti_wt <- analyze_wavelet(alb_Ti_ndet, dj = 1/100,
                             lowerPeriod = 0.05,
                             upperPeriod = 25,
                             verbose = FALSE,
                             omega_nr = 10
                             )

plot_wavelet(wavelet = alb_Ti_wt,
             lowerPeriod = NULL,
             upperPeriod = NULL,
             n.levels = 100,
             palette_name = "rainbow",
             color_brewer = "grDevices",
             useRaster = TRUE,
             periodlab = "Period (metres)",
             x_lab = "Depth (metres)",
             keep_editable = FALSE,
             dev_new = F,
             add_lines = NULL,
             add_points = NULL,
             add_abline_h = NULL,
             add_abline_v = NULL,
             add_MTM_peaks = FALSE,
             add_data = TRUE,
             add_avg = TRUE,
             add_MTM = FALSE,
             demean_mtm = TRUE,
             detrend_mtm = TRUE,
             padfac_mtm = 5,
             tbw_mtm = 3,
             plot_horizontal = TRUE
             )
```

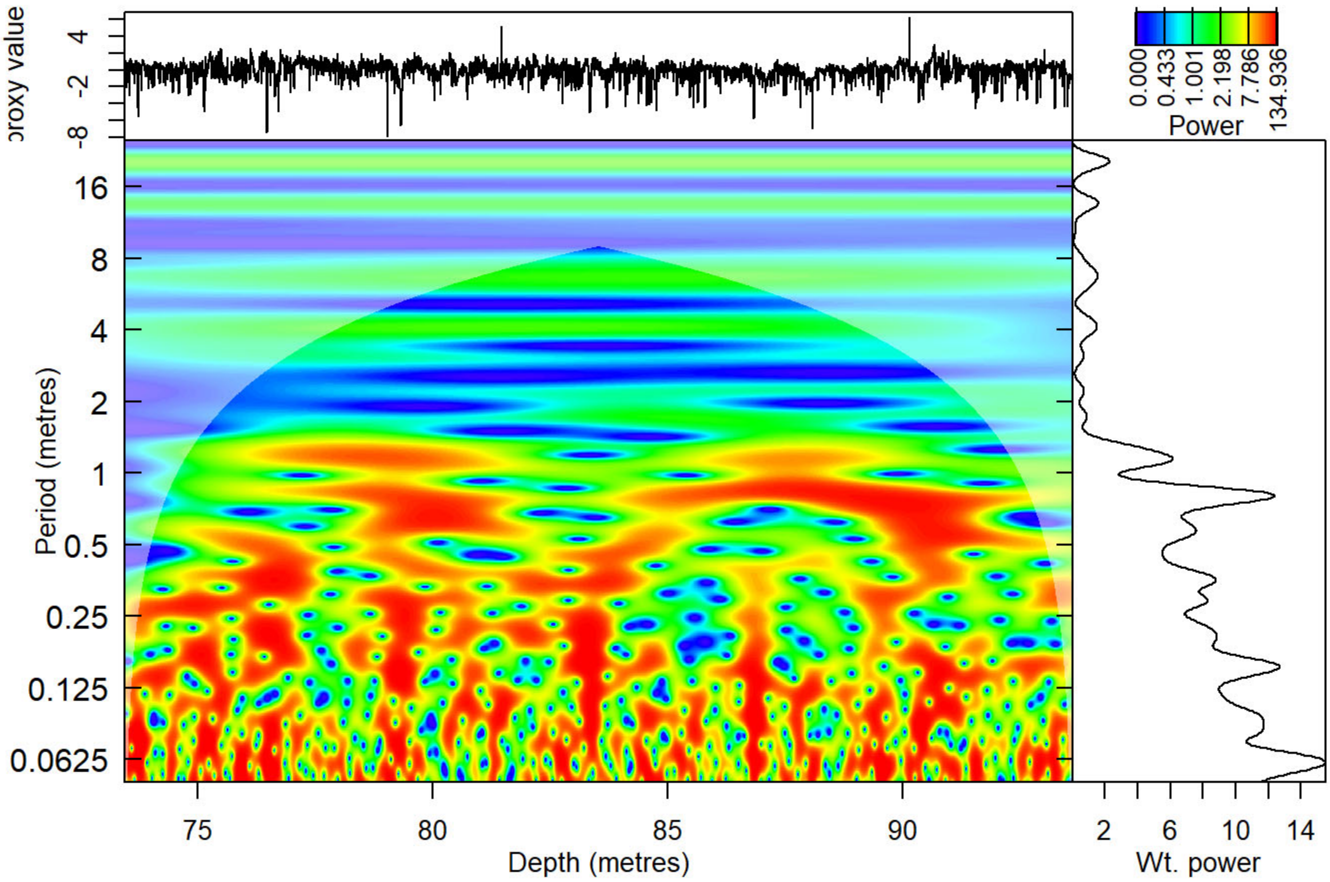


The Continuous Wavelet Transfrom (CWT) is performed on the Ti non-detrended series and exhibits similar results as the EHA analysis with a lot of power between 0.7-1m

## 4.2.2. Tracking (in depth domain) of the 173 kyr cycle

The powerful 0.7-1m (supposed to correspond to the 173 kyr cycle) is tracked along the ACL

```
#Tracking the wavelet (remove the "#" in front of the 3 next command lines)

#alb_Ti_track <- track_period_wavelet(astro_cycle = 173,
#                                    wavelet = alb_Ti_wt,
#                                     n.levels = 100,
#                                     periodlab = "Period (metres)",
#                                     x_lab = "depth (metres)",
#                                     palette_name = "rainbow",
#                                     color_brewer = "grDevices",
#                                     plot_horizontal = TRUE
#                                     )

#alb_Ti_track_comp <- completed_series(wavelet = alb_Ti_wt,
#                                      tracked_curve = alb_Ti_track,
#                                      period_up = 1,
#                                      period_down = 0.75,
#                                      extrapolate = TRUE,
#                                      genplot = FALSE,
#                                      keep_editable = FALSE
#                                      )
```

```
#alb_Ti_track_comp <- loess_auto(alb_Ti_track_comp)

#write.csv(alb_Ti_track_comp,"alb_Ti_track.csv")


# Loading the tracked curve
alb_Ti_track_comp <- read.csv("alb_Ti_track.csv")
alb_Ti_track_comp <- alb_Ti_track_comp[,c(2,3)]


plot_wavelet(wavelet = alb_Ti_wt,
             lowerPeriod = NULL,
             upperPeriod = NULL,
             n.levels = 100,
             palette_name = "rainbow",
             color_brewer = "grDevices",
             useRaster = TRUE,
             periodlab = "Period (metres)",
             x_lab = "Depth (metres)",
             keep_editable = FALSE,
             dev_new = F,
             add_lines = cbind(alb_Ti_track_comp[,1],alb_Ti_track_comp[,2]),
             add_points = NULL,
             add_abline_h = NULL,
             add_abline_v = NULL,
             add_MTM_peaks = FALSE,
             add_data = TRUE,
             add_avg = TRUE,
             add_MTM = FALSE,
             demean_mtm = TRUE,
             detrend_mtm = TRUE,
             padfac_mtm = 5,
             tbw_mtm = 3,
             plot_horizontal = TRUE
             )
```

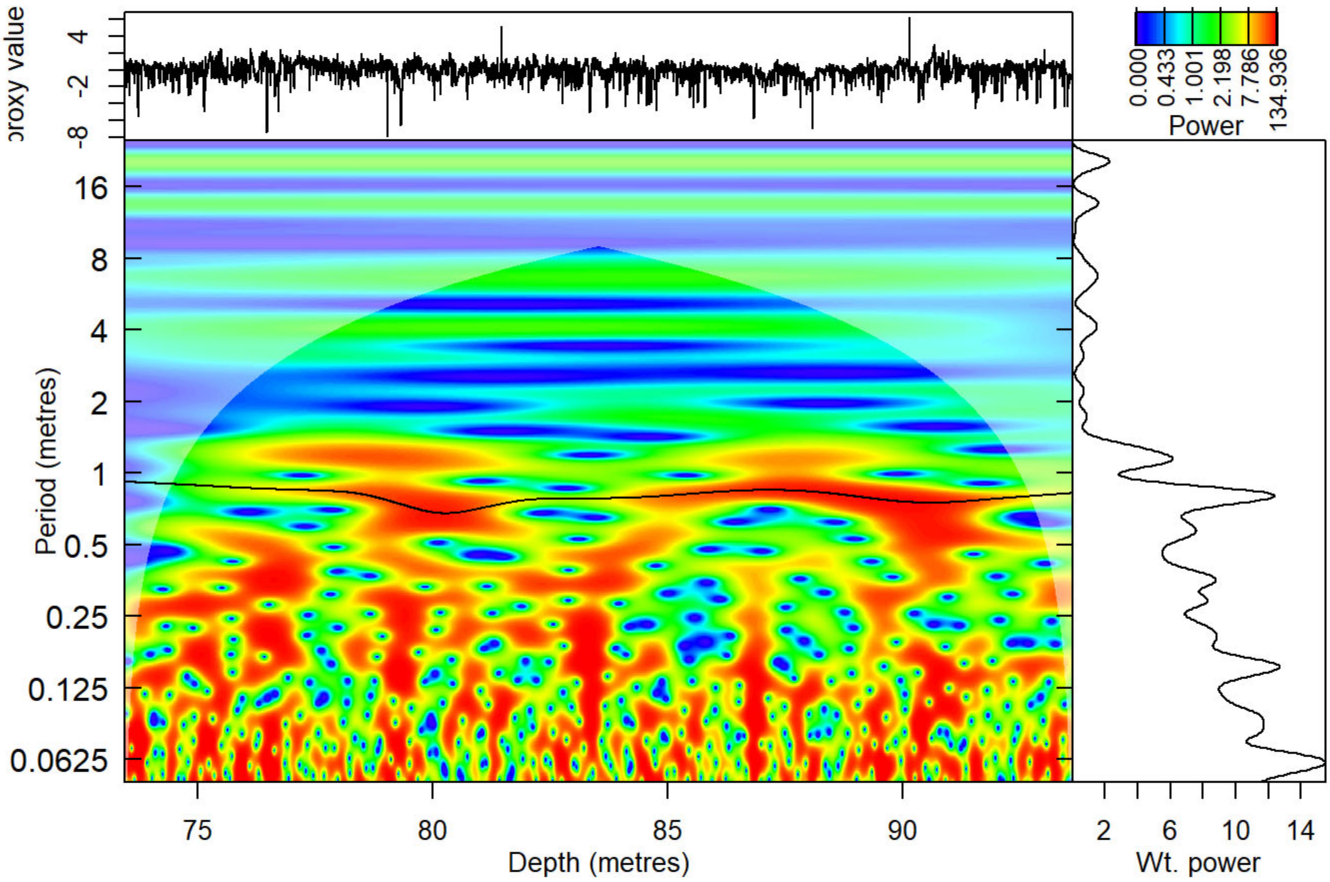


### 4.2.3. Depth to time domain convertion using the tracked cycle

```
alb_Ti_time <- curve2tune(data = alb_Ti_ndet,
                          tracked_cycle_curve = alb_Ti_track_comp,
                          tracked_cycle_period = 173,
                          genplot = FALSE,
                          keep_editable = FALSE
                          )

alb_Ti_time <- linterp(alb_Ti_time)
```

```
----- APPLYING PIECEWISE-LINEAR INTERPOLATION TO STRATIGRAPHIC SERIES -----

 * Number of samples= 4032
 * Determining median sampling interval for series
 * Will interpolate to median sampling interval of 1.082284
 * New number of samples= 4038
```

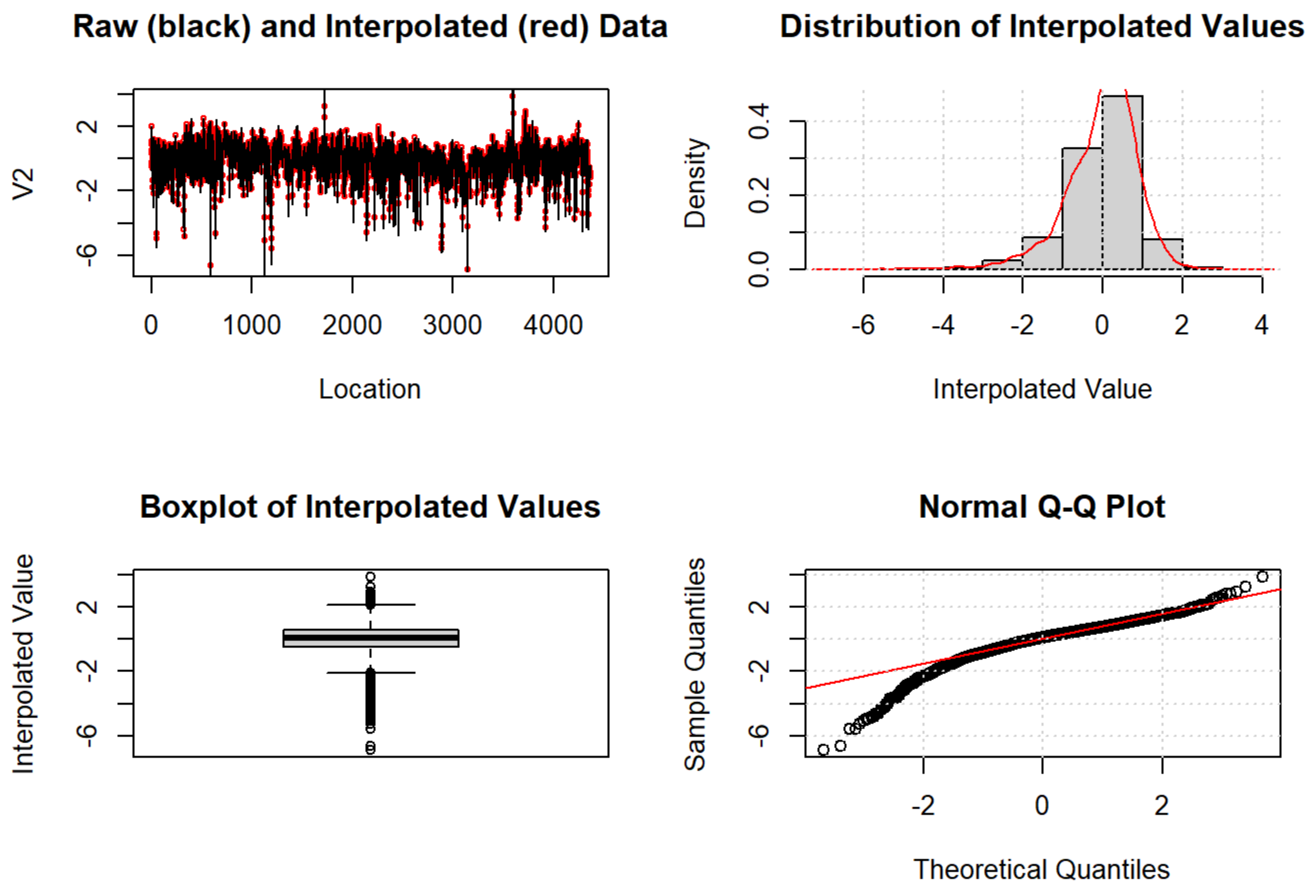


```
alb_Ti_time_wt <- analyze_wavelet(alb_Ti_time,
                                  dj = 1/100,
                                  lowerPeriod = 1,
                                  upperPeriod = 3000,
                                  verbose = FALSE,
                                  omega_nr = 10
                                  )

plot_wavelet(wavelet = alb_Ti_time_wt,
             lowerPeriod = NULL,
             upperPeriod = NULL,
             n.levels = 100,
             palette_name = "rainbow",
             color_brewer = "grDevices",
             useRaster = TRUE,
             periodlab = "Period (kyr)",
             x_lab = "Time (kyr)",
             keep_editable = FALSE,
             dev_new = F,
             add_lines = NULL,
             add_points = NULL,
             add_abline_h = c(20,32,100,110,125,173,405),
             add_abline_v = NULL,
             add_MTM_peaks = FALSE,
             add_data = TRUE,
             add_avg = TRUE,
             add_MTM = FALSE,
```

```
            demean_mtm = TRUE,
            detrend_mtm = TRUE,
            padfac_mtm = 5,
            tbw_mtm = 3,
            plot_horizontal = TRUE
            )
```

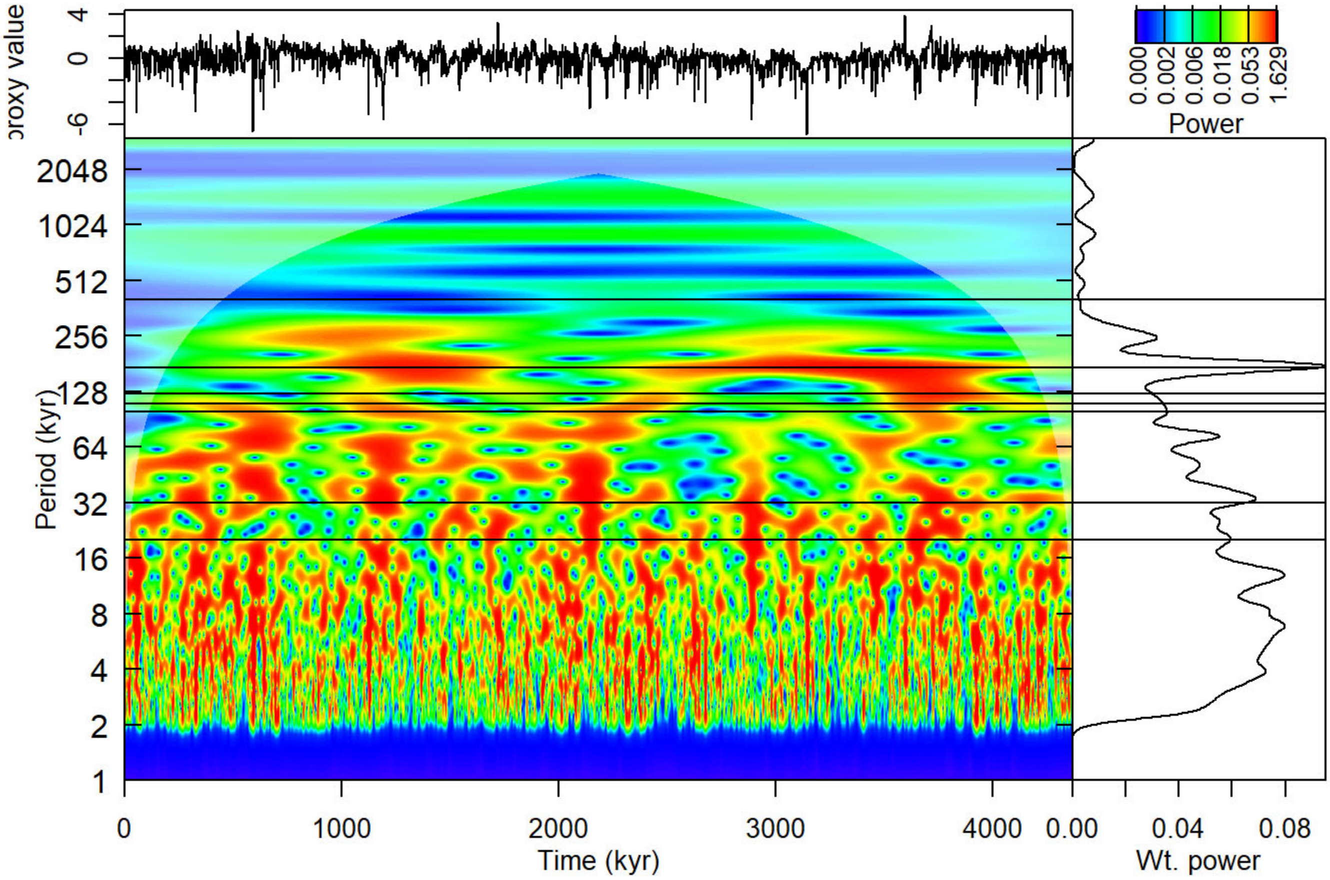


The CWT performed in time domain shows a lot of power and/or prominent peaks in the Milankovitch bands (precession: ~20 kyr, obliquity: ~32 kyr, short eccentricity: ~100-125 kyr and 173 kyr) in line with the expectations from the literature.

---------------------------------------------------------------------------------------------------

## Step 5: Average Spectral Misfit (ASM)

```
freq <- c(1.151,1.458,1.548,1.647,1.746,1.964,2.093,5.228,5.704,5.853,
          6.240,7.103,7.202,10.317,11.101,12.55,12.659)

target <- c(1/405,1/173,1/131.3,1/123.9,1/99.2,1/94.9,1/38.8,1/32.1,
            1/31.6,1/31.3,1/30.8, 1/24.4,1/20.2,1/19.8,1/19.3,1/16.8,1/16.7)

rayleigh <- 0.1

nyquist <- 15
```

```
asm(freq,
    target,
    fper=NULL,
    rayleigh,
    nyquist,
    sedmin=0.05,
    sedmax=5,
    numsed=500,
    linLog=1,
    iter=100000,
    output=F,
    genplot=T
    )
```

```
----- PERFORMING AVERAGE SPECTRAL MISFIT ANALYSIS -----

**** WARNING: No uncertainty assigned to astronomical target frequencies.


 * Analysis complete:
    Optimal Sedimentation Rate (cm/ka) at = 0.4931261
    Ho-SL (%) = 9.561
      or p-value = 0.09561
    ASM (cycles/ka) = 0.001008439
    Number of Astronomical Terms Fit = 17
```

The ASM analysis is performed confronting the > 90 % CL frequencies from the MTM analysis with the theroretical duration of Milankovitch cycles.

The ASM results in a sedimentation rate of 0.493 cm/ka, consistent with the 0.4-0.6 cm/kyr expectation for Blatica during the Cambrian.

---

# Step 6: Milankovitch bands filters in depth domain

```
#405 kyr cycle (1.75-2.25 m)
alb_Ti_405 <- taner(alb_Ti, xmax=5, fhigh=1/1.75, flow=1/2.25, demean = T)
```

```
----- TANER BANDPASS FILTERING STRATIGRAPHIC SERIES-----
 * Number of data points= 4032
 * Sample interval= 0.005
 * Mean value removed= 4.069401e-05
```

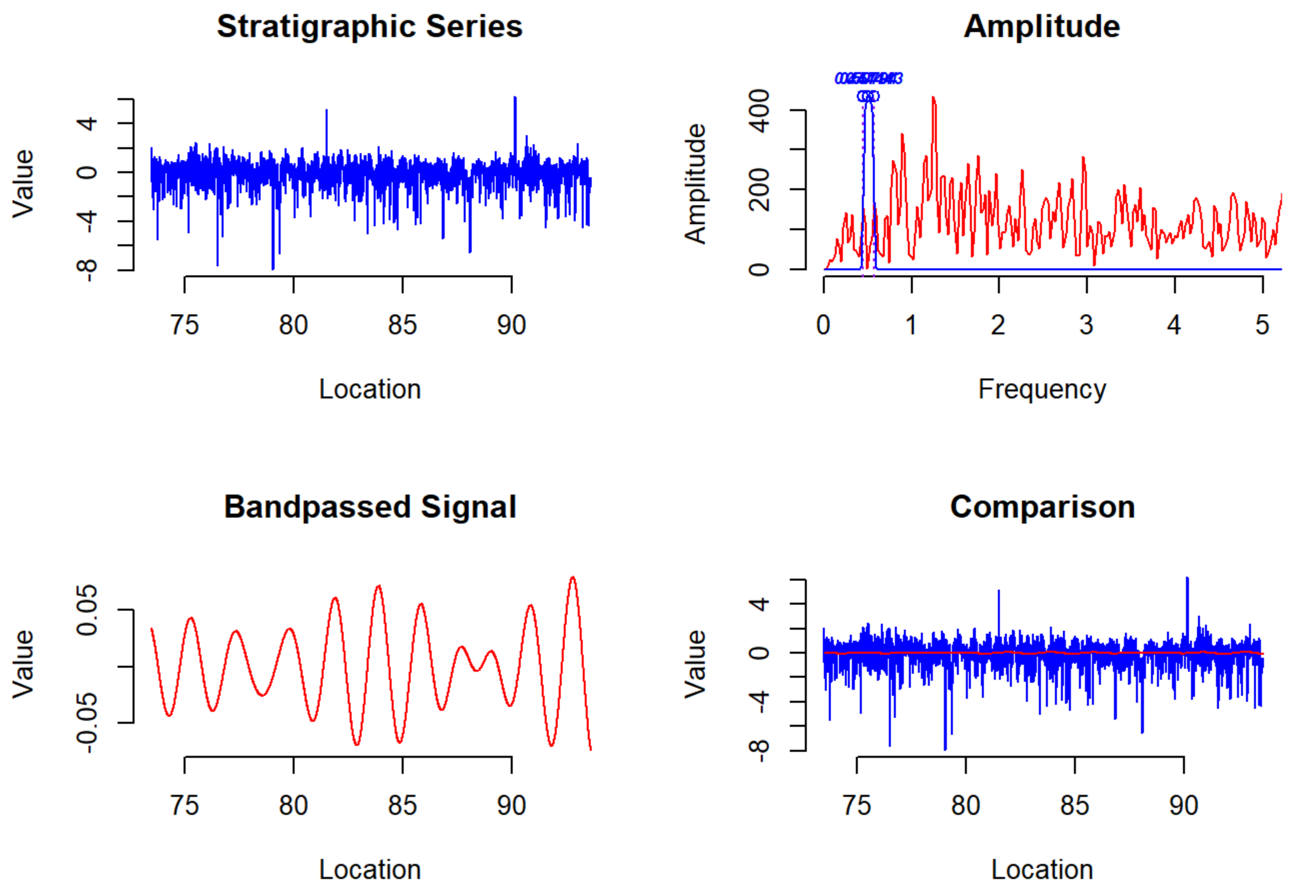


```
#173 kyr cycle (0.76-1 m)
alb_Ti_173 <- taner(alb_Ti,xmax=5, fhigh=1/0.76, flow=1, demean = T)
```

```
----- TANER BANDPASS FILTERING STRATIGRAPHIC SERIES-----
 * Number of data points= 4032
 * Sample interval= 0.005
 * Mean value removed= 4.069401e-05
```

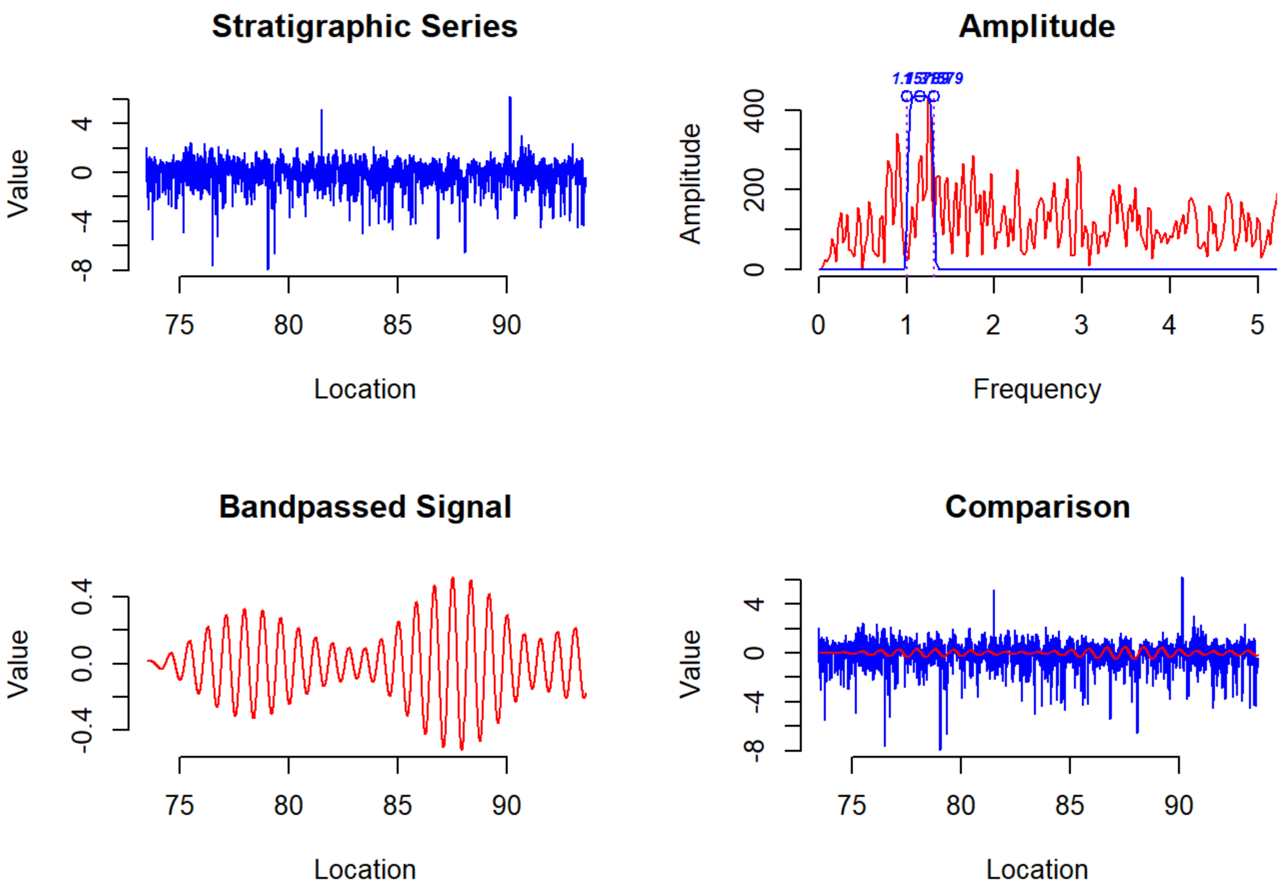


```
#short eccentricity band (0.42-0.72 m)
alb_Ti_100 <- taner(alb_Ti, xmax=5, fhigh=1/0.42, flow=1/0.72, demean = T)
```

```
----- TANER BANDPASS FILTERING STRATIGRAPHIC SERIES-----
 * Number of data points= 4032
 * Sample interval= 0.005
 * Mean value removed= 4.069401e-05
```

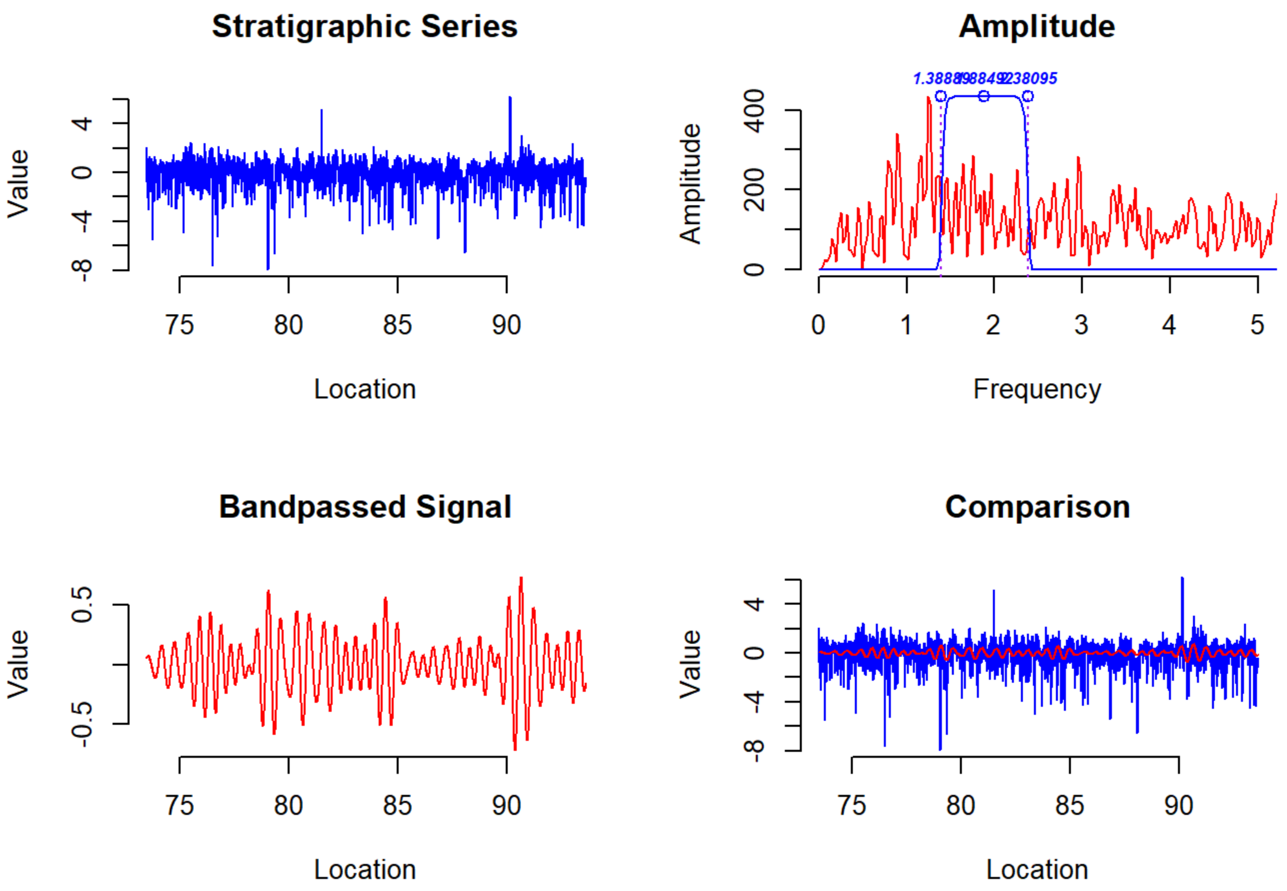


```
#obliquity band (0.12-0.2 m)
alb_Ti_31 <- taner(alb_Ti, xmax=10, fhigh=1/0.12, flow=1/0.2, demean=T)
```

```
----- TANER BANDPASS FILTERING STRATIGRAPHIC SERIES-----
 * Number of data points= 4032
 * Sample interval= 0.005
 * Mean value removed= 4.069401e-05
```

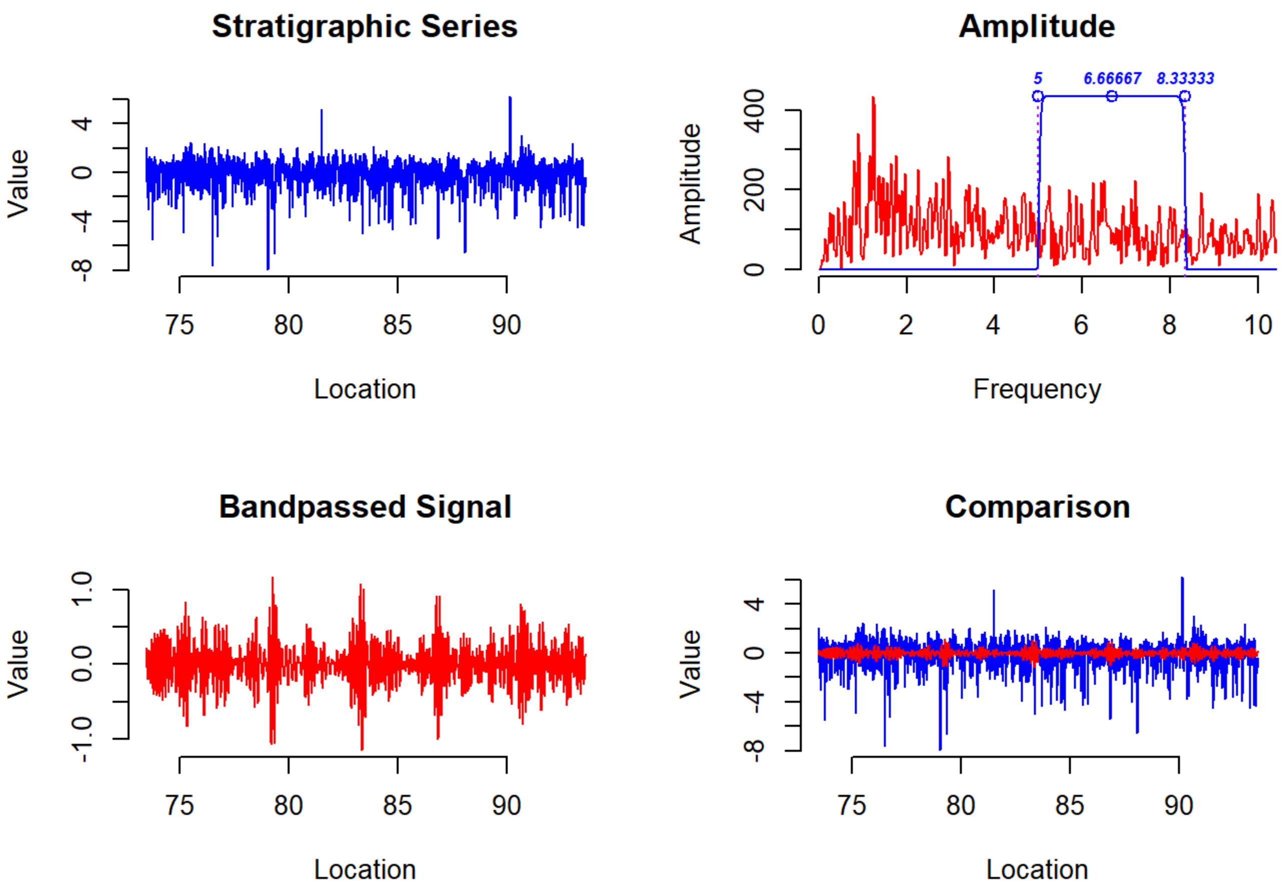


```
#precession band (0.08-0.1 m)
alb_Ti_20 <- taner(alb_Ti, xmax=15, fhigh=1/0.08, flow=1/0.1, demean=T)
```

```
----- TANER BANDPASS FILTERING STRATIGRAPHIC SERIES-----
 * Number of data points= 4032
 * Sample interval= 0.005
 * Mean value removed= 4.069401e-05
```

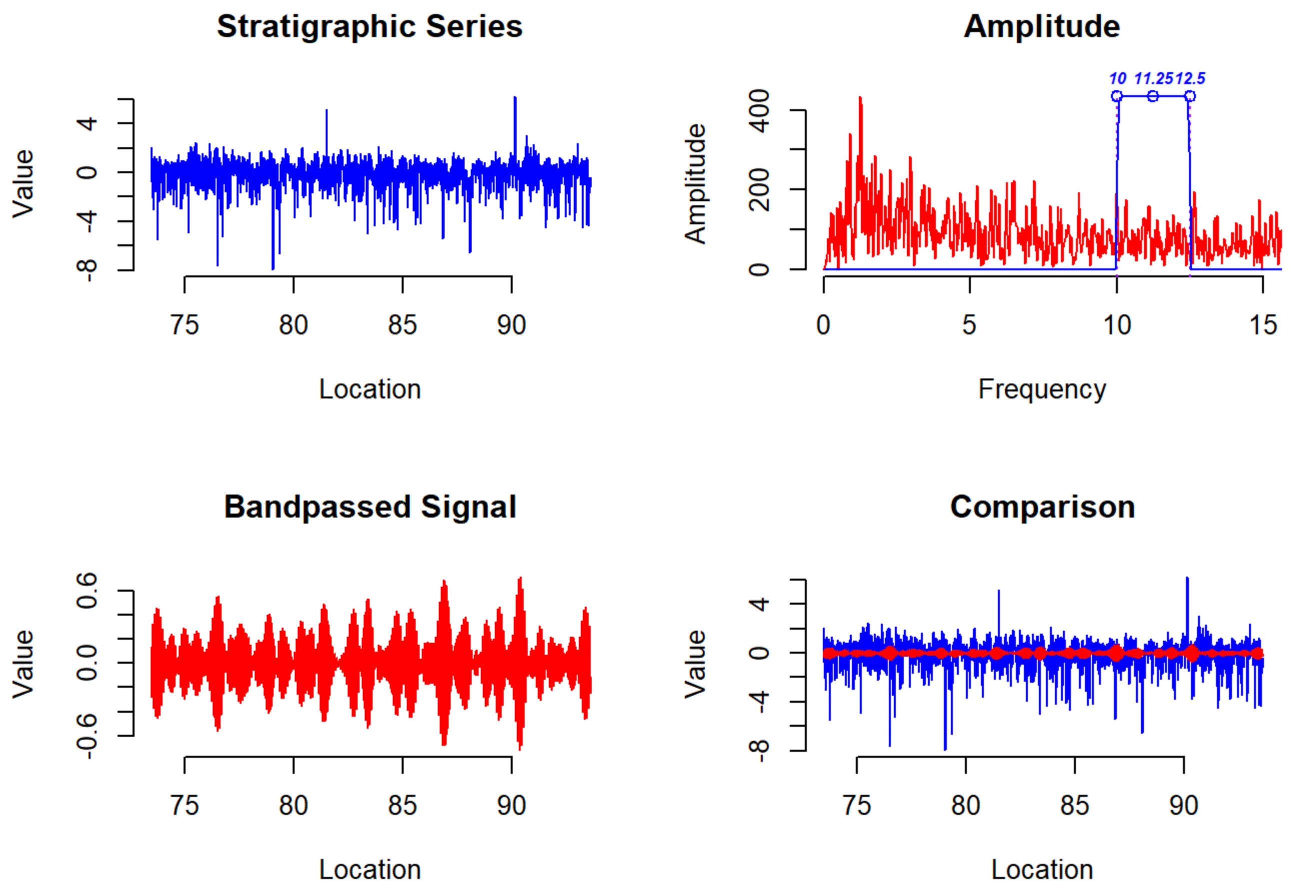


The filtering of the Milankovitch bands in depth domain allows to see the imprint of the astronomical cycles on the Ti sedimentary record.

---------------------------------------------------------------------------------------------------

# Step 7: Tuning of the Ti series

## 7.1. Filtering of the 0.76-0.97 m band

```
Ti_173R <- taner(alb_Ti, xmax=2, fhigh=1/0.97, flow=1/0.76, demean=T)
```

```
----- TANER BANDPASS FILTERING STRATIGRAPHIC SERIES-----
 * Number of data points= 4032
 * Sample interval= 0.005
 * Mean value removed= 4.069401e-05
```

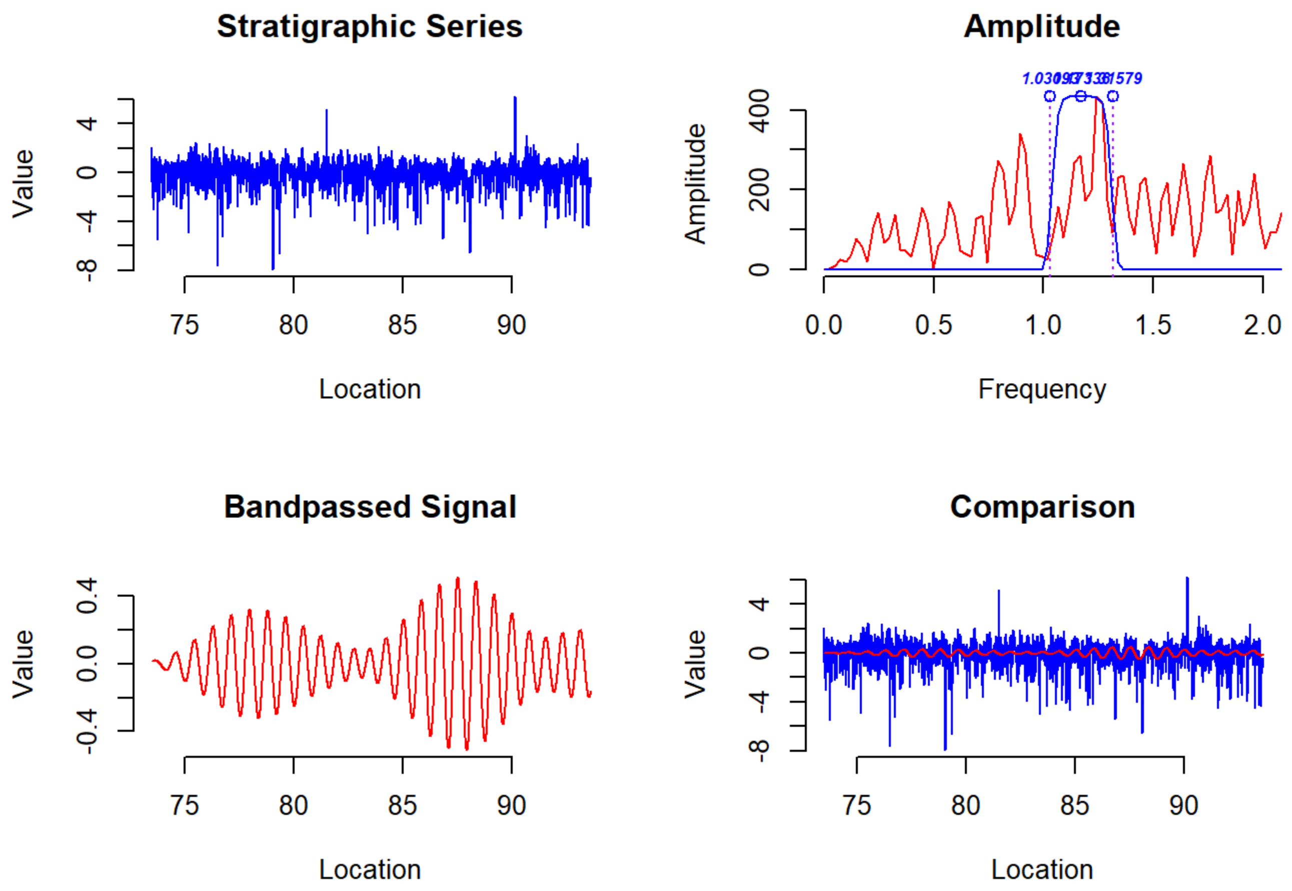


## 7.2. Identification of the trough of the filtered signal

```
Ti_173_min <- trough(Ti_173R, level = 200, genplot = T)
```

```
----- FINDING MINIMA OF TROUGHS, FILTERING AT THRESHOLD VALUE -----
 * Number of data points= 4032
 * Number of columns= 2
 * Identifying minima of troughs
 * Number of troughs identified= 25
 * Filtering troughs at threshold of 200
 * Number of troughs <= 200 : 25
```

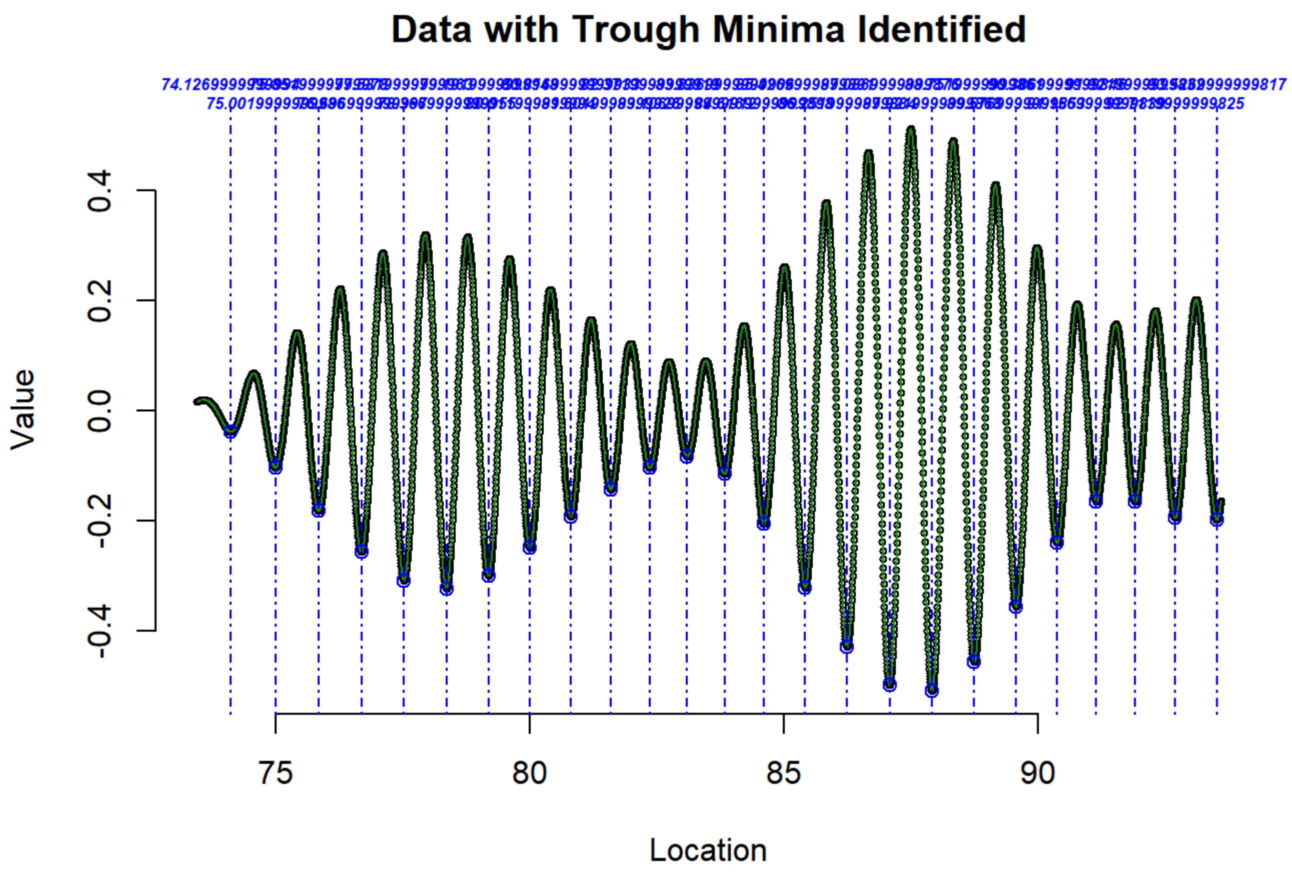


```
Ti_173_min <- Ti_173_min[,c(2,3)]

#write.csv(Ti_173_min,"Ti_173_rsp0.005_minTan_0.76-0.97m.csv")
```

## 7.3. Trough = multiple of 173 kyr cycle

Once the ".csv" file is created, go into it and for each location, add a multiple of 173 for the trough value as follows:

Location Trough_Value

74.127; 173

75.002; 346

75.852; 519

76.697; 692

…; …

93.527; 4325

## 7.4. Tuning of the 5mm spaced Ti detrended dataset

```
Ti_173_min <- read.csv("Ti_173_rsp0.005_minTan_0.76-0.97m.csv", sep=";")

Ti_173_tuned <- tune(alb_Ti, Ti_173_min, extrapolate=T, genplot=T, check=T, verbose=T)
```

```
----- TUNING STRATIGRAPHIC SERIES -----
 * Number of data points= 4032
 * Number of time control points= 25
 * Sorting datasets into ensure increasing order, removing empty entries

 * Mean sampling interval= 1.067338
 * Median sampling interval= 1.054878
 * Maximum sampling interval= 1.184932
 * Minimum sampling interval= 0.9885714
```

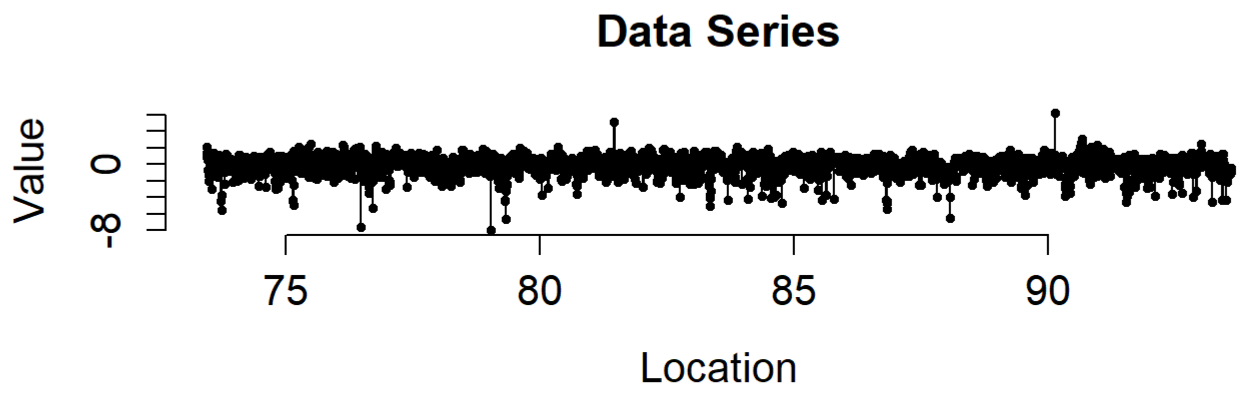


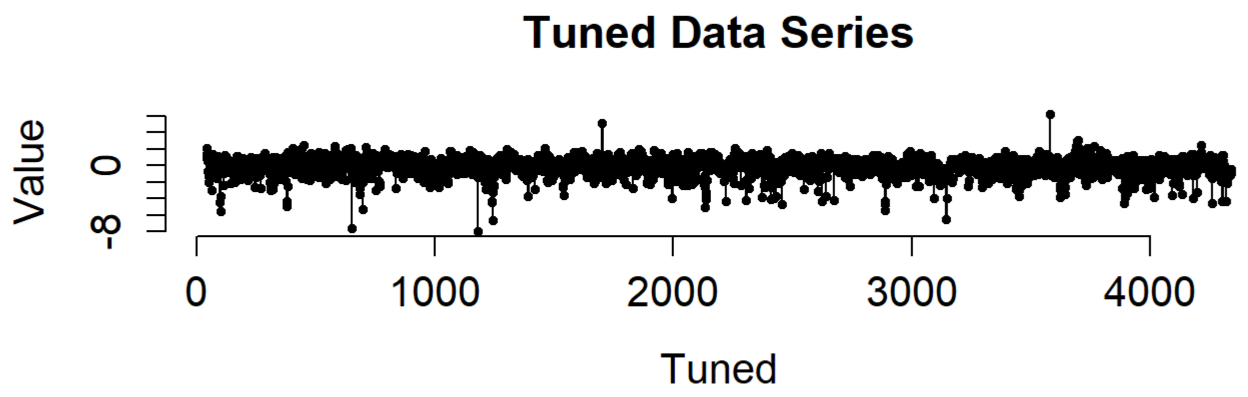


## 7.5. Evenly spaced dataset in time domain

Interpolation of the tuned dataset every 1.067338 kyr (mean sampling interval) to have an evenly space tune dataset

```
alb_Ti_time <- linterp(Ti_173_tuned, dt=1.067338, genplot=T)
```

```
----- APPLYING PIECEWISE-LINEAR INTERPOLATION TO STRATIGRAPHIC SERIES -----
```

```
 * Number of samples= 4032
 * New number of samples= 4031
```

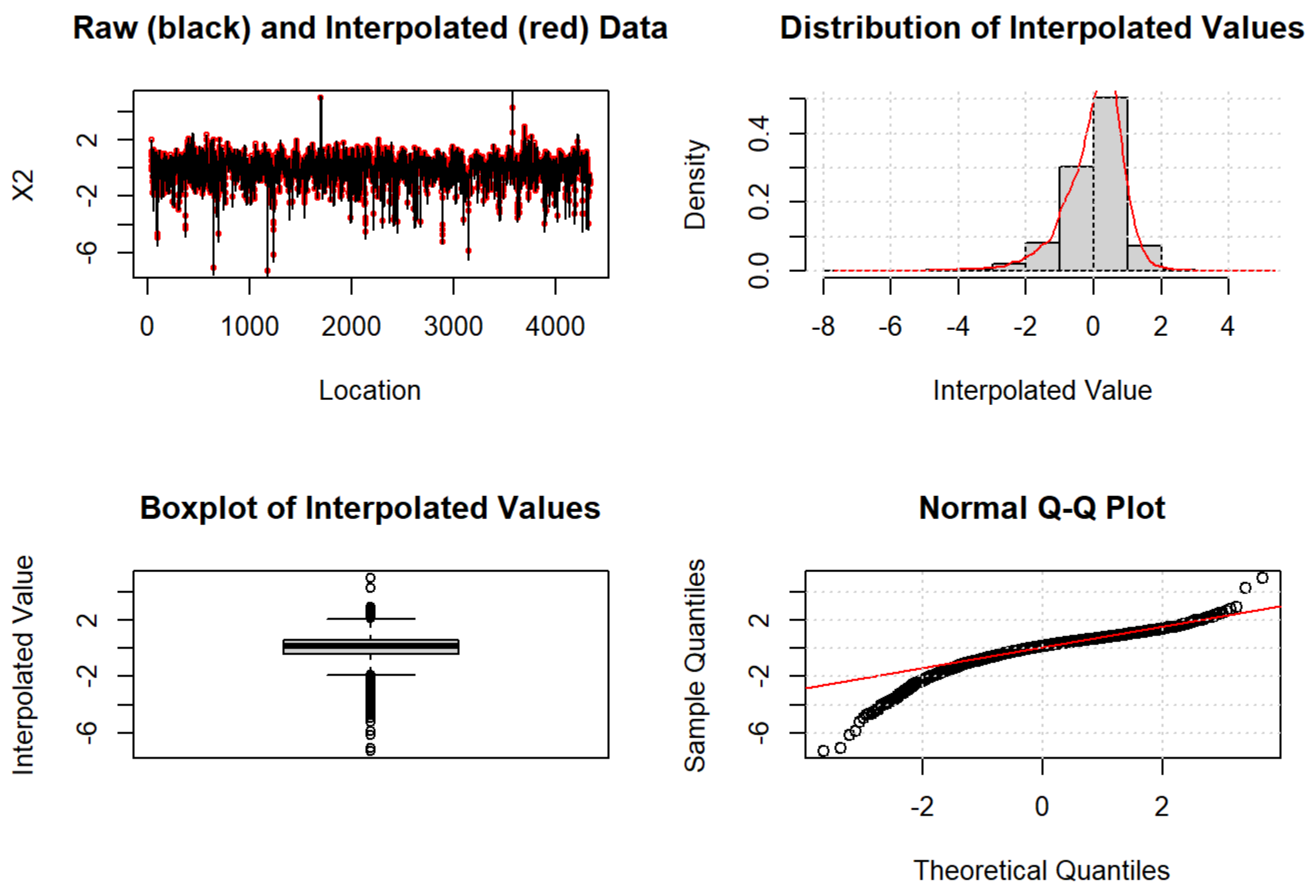


---

# Step 8: MTM and EHA in time domain

## 8.1. MTM in time domain

```
alb_Ti_time_MTM<-mtm(alb_Ti_time, xmax=0.07, siglevel = 0.9, ar1 = T, tbw= 2, output = 1)
```

```
----- PERFORMING Multitaper Spectral Analysis -----
 * Number of data points in stratigraphic series: 4031
 * Stratigraphic series length (space or time): 4301.372
 * Sampling interval (space or time): 1.067338
 * Will use default setting of 3 DPSS tapers
 * Mean value subtracted= 0.0002517748
 * Linear trend NOT subtracted
 * Nyquist frequency: 0.4684552
 * Rayleigh frequency: 0.0002324263
 * MTM Power spectrum bandwidth resolution (halfwidth): 0.0004648526
 * Padded to 20156 points
 * Estimated AR1 coefficient = 0.6212844

 * Searching for significant spectral peaks that satisfy 90 % CL
```

requirements outlined in Meyers (2012):

* Number of significant F-test peaks identified = 92

| ID | Frequency | Period | Harmonic_CL | Rednoise_CL |
|---|---|---|---|---|
| 1 | 0.003672153 | 272.3198 | 98.11969 | 94.30495 |
| 2 | 0.004183465 | 239.0363 | 99.9596 | 99.94852 |
| 3 | 0.005763886 | 173.4941 | 99.59287 | 100 |
| 4 | 0.006693545 | 149.3977 | 99.93526 | 94.85659 |
| 5 | 0.007576721 | 131.9832 | 99.38519 | 98.51764 |
| 6 | 0.00850638 | 117.5588 | 97.05777 | 84.83089 |
| 7 | 0.009110658 | 109.7616 | 99.83016 | 88.81513 |
| 8 | 0.01148129 | 87.09824 | 91.49029 | 60.62901 |
| 9 | 0.01366599 | 73.17437 | 93.73957 | 69.35043 |
| 10 | 0.0158042 | 63.27431 | 96.79027 | 70.85455 |
| 11 | 0.02003415 | 49.91477 | 91.8715 | 99.14432 |
| 12 | 0.02500783 | 39.98748 | 92.75957 | 81.17411 |
| 13 | 0.02551914 | 39.18627 | 93.30452 | 82.46565 |
| 14 | 0.03091116 | 32.35077 | 94.76663 | 81.53078 |
| 15 | 0.03146896 | 31.77735 | 92.44902 | 89.46365 |
| 16 | 0.07888157 | 12.67723 | 98.24804 | 81.27618 |
| 17 | 0.08311151 | 12.03203 | 99.41217 | 93.76132 |
| 18 | 0.08520325 | 11.73664 | 99.78533 | 78.37017 |
| 19 | 0.0924081 | 10.82156 | 98.55063 | 97.42502 |
| 20 | 0.1046331 | 9.557203 | 99.35061 | 72.60485 |
| 21 | 0.1083518 | 9.2292 | 98.04082 | 59.37082 |
| 22 | 0.1189499 | 8.406903 | 93.34178 | 95.41128 |
| 23 | 0.1204373 | 8.303074 | 90.56264 | 88.05812 |
| 24 | 0.1385657 | 7.216795 | 98.05109 | 97.67991 |
| 25 | 0.1396348 | 7.16154 | 90.55456 | 86.19492 |
| 26 | 0.1450733 | 6.893068 | 92.72452 | 92.61329 |
| 27 | 0.1464678 | 6.82744 | 93.29638 | 82.1956 |
| 28 | 0.1504653 | 6.64605 | 94.22877 | 72.70561 |
| 29 | 0.1521852 | 6.570942 | 96.01006 | 87.28064 |
| 30 | 0.1688726 | 5.921625 | 94.73928 | 59.53296 |
| 31 | 0.1694304 | 5.90213 | 98.06945 | 75.66421 |
| 32 | 0.1751478 | 5.709465 | 99.53258 | 73.8443 |
| 33 | 0.1756126 | 5.694353 | 92.28933 | 86.49587 |
| 34 | 0.1777043 | 5.627325 | 95.84424 | 91.07312 |
| 35 | 0.1787734 | 5.593673 | 98.74848 | 96.1525 |
| 36 | 0.1841654 | 5.4299 | 95.13835 | 93.33122 |
| 37 | 0.1899758 | 5.263828 | 92.77685 | 94.60851 |
| 38 | 0.1930437 | 5.180175 | 98.45542 | 77.28544 |
| 39 | 0.1984357 | 5.039415 | 92.93684 | 76.75734 |
| 40 | 0.199133 | 5.02177 | 96.27157 | 79.9269 |
| 41 | 0.2035488 | 4.912826 | 97.12894 | 91.63699 |
| 42 | 0.2126595 | 4.702353 | 95.2339 | 97.48242 |
| 43 | 0.2133567 | 4.686986 | 99.82262 | 79.82971 |
| 44 | 0.2140075 | 4.672733 | 98.66001 | 95.50713 |
| 45 | 0.2169824 | 4.608669 | 99.91905 | 83.88459 |
| 46 | 0.218191 | 4.583141 | 98.57328 | 96.36083 |
| 47 | 0.2366447 | 4.225744 | 95.4402 | 83.09314 |
| 48 | 0.2404563 | 4.15876 | 97.94883 | 89.73545 |
| 49 | 0.2438496 | 4.100889 | 90.44932 | 89.0816 |
| 50 | 0.2458483 | 4.067549 | 98.96937 | 65.27153 |
| 51 | 0.2491021 | 4.014418 | 97.02854 | 88.59115 |

| | | | | |
|---|---|---|---|---|
| 52 | 0.2545871 | 3.927929 | 97.9532 | 96.12543 |
| 53 | 0.2637443 | 3.791552 | 91.03357 | 83.59602 |
| 54 | 0.2665332 | 3.751877 | 95.18007 | 61.42055 |
| 55 | 0.2670445 | 3.744694 | 94.39814 | 81.11712 |
| 56 | 0.2676953 | 3.73559 | 98.92259 | 69.35863 |
| 57 | 0.2682066 | 3.728469 | 91.48261 | 90.09225 |
| 58 | 0.2702984 | 3.699616 | 94.62618 | 94.21628 |
| 59 | 0.2719717 | 3.676853 | 97.87828 | 82.45936 |
| 60 | 0.2763876 | 3.618107 | 99.5166 | 97.56497 |
| 61 | 0.2775032 | 3.603562 | 99.72265 | 80.50463 |
| 62 | 0.2790371 | 3.583752 | 97.50418 | 83.856 |
| 63 | 0.2865209 | 3.490147 | 98.47766 | 87.8011 |
| 64 | 0.2881943 | 3.469881 | 90.22236 | 90.43317 |
| 65 | 0.2933539 | 3.408852 | 99.06799 | 84.75965 |
| 66 | 0.2948878 | 3.39112 | 90.61869 | 90.75301 |
| 67 | 0.295771 | 3.380994 | 99.87185 | 88.54789 |
| 68 | 0.29856 | 3.349411 | 96.87521 | 79.55894 |
| 69 | 0.301256 | 3.319436 | 93.67934 | 98.21133 |
| 70 | 0.3028829 | 3.301606 | 93.53422 | 82.58865 |
| 71 | 0.3036266 | 3.293519 | 95.67298 | 82.83303 |
| 72 | 0.3043239 | 3.285973 | 99.44436 | 97.6364 |
| 73 | 0.3049281 | 3.279461 | 96.0226 | 79.69787 |
| 74 | 0.3101807 | 3.223927 | 97.95119 | 96.23019 |
| 75 | 0.3262173 | 3.065441 | 97.45783 | 81.97655 |
| 76 | 0.3330503 | 3.002549 | 92.79158 | 99.85386 |
| 77 | 0.3377451 | 2.960813 | 99.22818 | 75.69901 |
| 78 | 0.3433231 | 2.912708 | 93.21386 | 89.50133 |
| 79 | 0.3459726 | 2.890402 | 94.27931 | 88.59534 |
| 80 | 0.3467628 | 2.883816 | 91.75708 | 77.67877 |
| 81 | 0.3510857 | 2.848307 | 98.04238 | 91.04519 |
| 82 | 0.3755357 | 2.662862 | 91.87629 | 57.88583 |
| 83 | 0.4113276 | 2.431152 | 99.71812 | 89.88467 |
| 84 | 0.4196481 | 2.382949 | 94.69839 | 85.34974 |
| 85 | 0.4240639 | 2.358135 | 98.08403 | 97.02254 |
| 86 | 0.4422853 | 2.260984 | 98.02701 | 77.99548 |
| 87 | 0.4428895 | 2.257899 | 96.66744 | 82.09898 |
| 88 | 0.4442375 | 2.251048 | 94.98735 | 77.30791 |
| 89 | 0.4534877 | 2.205132 | 92.15221 | 64.84901 |
| 90 | 0.453999 | 2.202648 | 95.81803 | 85.82515 |
| 91 | 0.460832 | 2.169988 | 91.3433 | 86.07989 |
| 92 | 0.4617151 | 2.165838 | 93.16843 | 87.15591 |

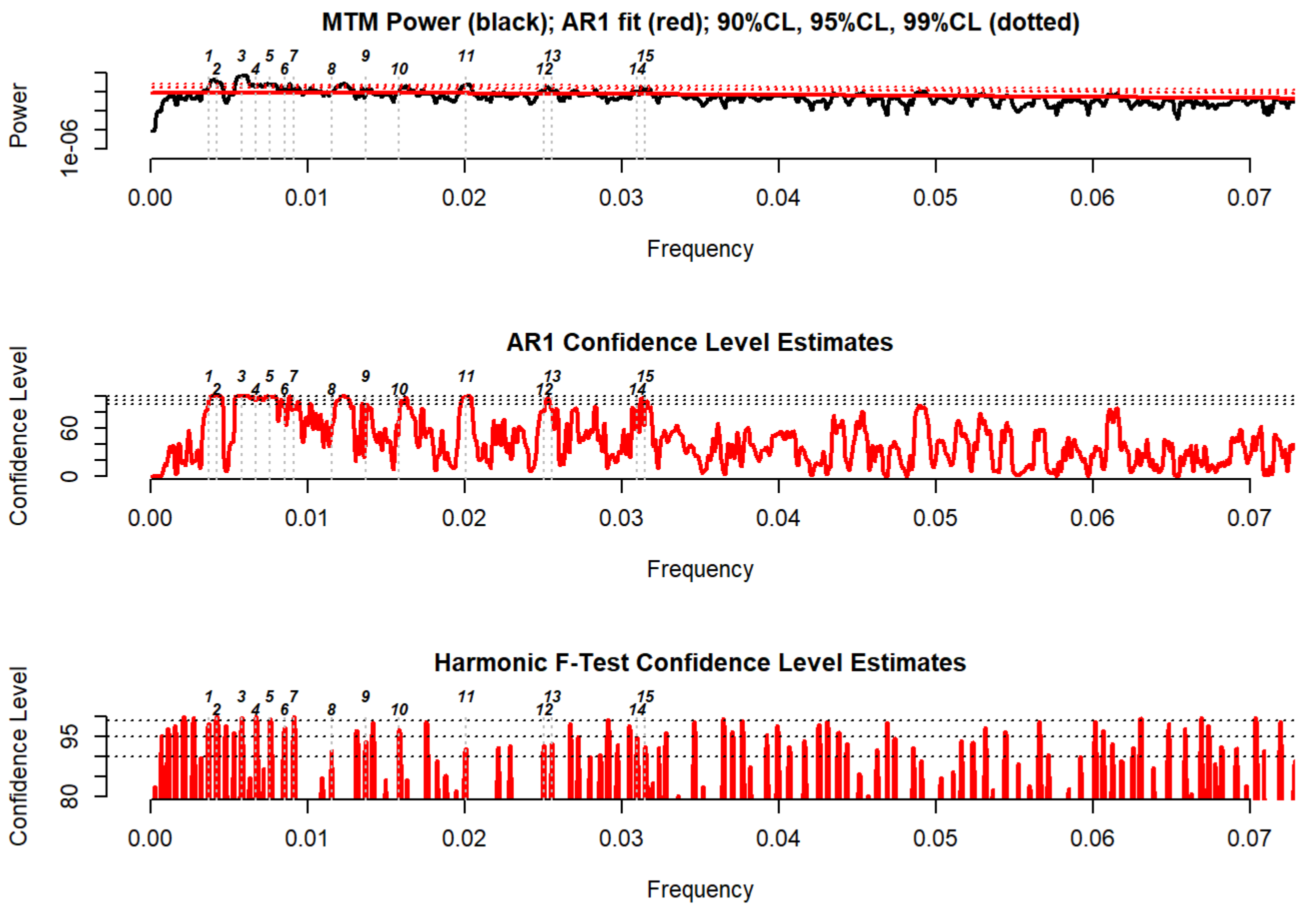


```
alb_Ti_time_MTM <- iso(dat=alb_Ti_time_MTM,xmin=0,xmax=0.07, genplot=F)
```

```
----- ISOLATE STRATIGRAPHIC DATA BY LOCATION -----
 * Number of data points= 10077
 * Number of columns= 8
 * Minimum= 4.648295e-05 , Maximum= 0.4684087
 * Isolating data between 0 and 0.07
 * Number of data points following culling= 1505
```

```
plot(alb_Ti_time_MTM$Frequency,
     alb_Ti_time_MTM$Power,
     type = 'l',
     xlab = "Frequency (cycles/kyr)",
     ylab = "Variance"
     )

lines(alb_Ti_time_MTM$Frequency,
      alb_Ti_time_MTM$AR1_fit,
      type = 'l',
      col = 'black',
      lwd = 2
      )

lines(alb_Ti_time_MTM$Frequency,
      alb_Ti_time_MTM$AR1_90_power,
      type = 'l',
```

```
        col = 'red',
        lwd = 2
        )

lines(alb_Ti_time_MTM$Frequency,
        alb_Ti_time_MTM$AR1_95_power,
        type = 'l',
        col = 'blue',
        lwd = 2
        )

lines(alb_Ti_time_MTM$Frequency,
        alb_Ti_time_MTM$AR1_99_power,
        type = 'l',
        col = 'green',
        lwd = 2
        )
```

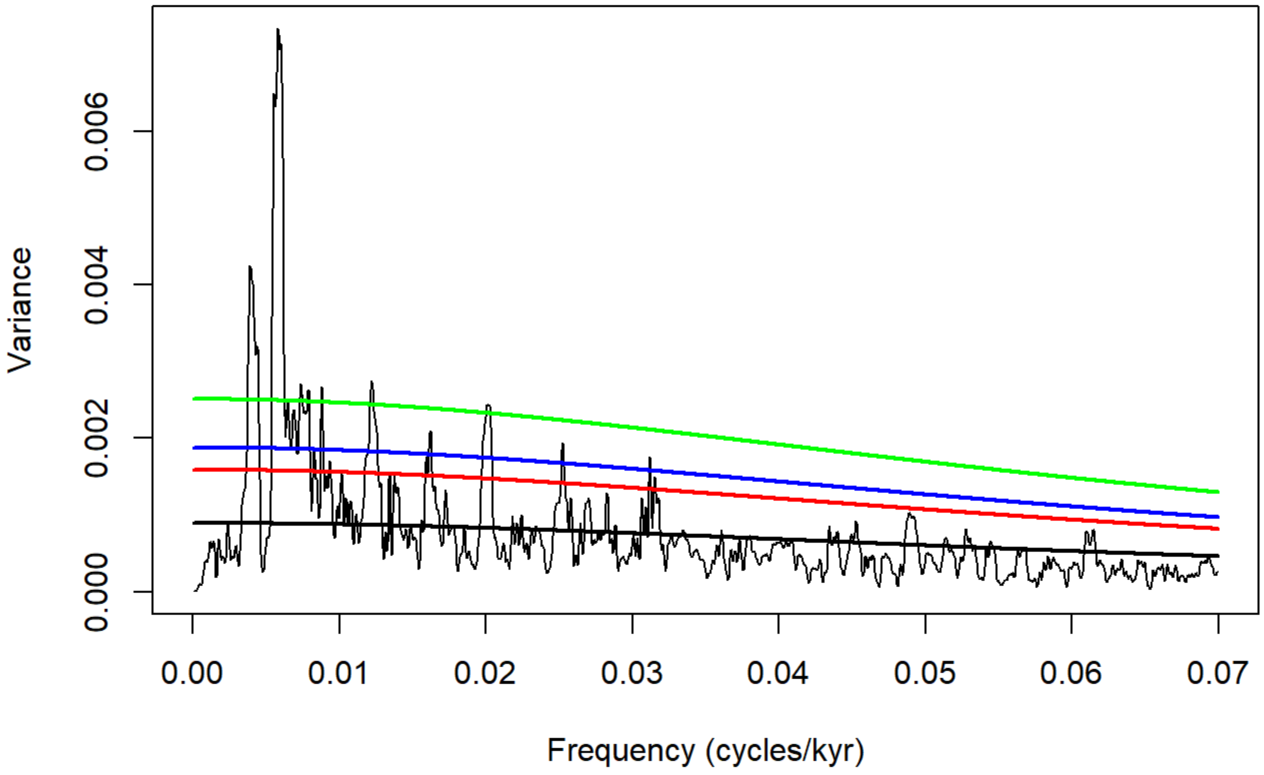


The 2 pi-MTM on the tuned dataset shows the range of cyclicities in the signal in time domain. Allowing to visually identify > 90 % CL periodicities.

## 8.2. EHA in time domain

```
alb_Ti_time_EHA<- eha(alb_Ti_time,
                      tbw=2,
                      fmin=0.001,
```

```
                        fmax=0.07,
                        step=1,
                        win=1100,
                        demean=T,
                        detrend=T,
                        siglevel=0.90,
                        sigID=F,
                        ydir=1,
                        output=1,
                        pl=1,
                        palette=1,
                        centerZero=T,
                        ncolors=200,
                        genplot=4,
                        verbose=T
                        )
```

```
 ----- PERFORMING EVOLUTIVE HARMONIC ANALYSIS -----
 * Number of data points in stratigraphic series: 4031
 * Stratigraphic series length (space or time): 4301.372
 * Sampling interval (space or time): 1.067338
 * Number of data points per window: 1031
 * Moving window size (space or time): 1099.358
 * Window step points: 1
 * Window step (space or time): 1.067338
 * Number of windows: 3001
 * Mean value for each window will be subtracted
 * Linear trend for each window will be subtracted
 * Nyquist frequency: 0.4684552
 * Rayleigh frequency: 0.0009087394
 * MTM Power spectrum bandwidth resolution (halfwidth): 0.001817479
 * Will use 3 DPSS tapers
 * Padded to 4096 points
```

The EHA is performed on the Ti tuned series and confirms the presence of recurrent periodicities in the Milankovitch bands along the ACL as suggested by the > 90 % CL frequencies observed in the 2 pi-MTM

---

# Step 9: Hilbert transform analysis between obliquity and the 173 kyr cycle

```
#Filter out the obliquity cycle
alb_Ti_time_obl <- taner(alb_Ti_time, xmax=1/20, fhigh=1/27, flow=1/40)
```

```
----- TANER BANDPASS FILTERING STRATIGRAPHIC SERIES-----
 * Number of data points= 4031
 * Sample interval= 1.067338
 * Mean value removed= 0.0002517748
```

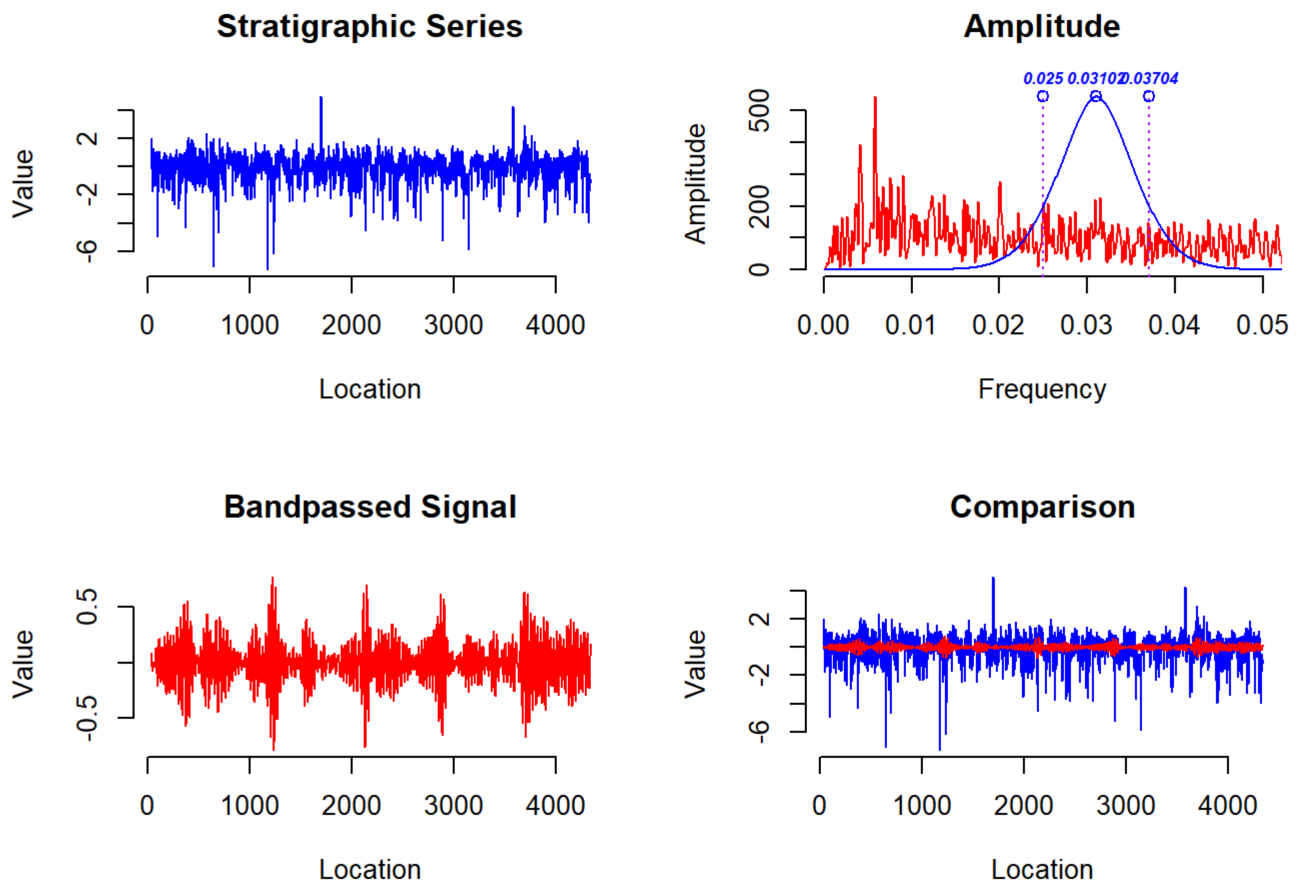


```r
#Do the Hilbert transform to extract the amplitude
alb_Ti_time_obl_hilbert <- hilbert(alb_Ti_time_obl)
```

```
----- PERFORMING HILBERT TRANSFORM ON STRATIGRAPHIC SERIES -----
 * Number of data points= 4031
 * Sample interval= 1.067338
 * Mean value removed= 0.0001213115
```

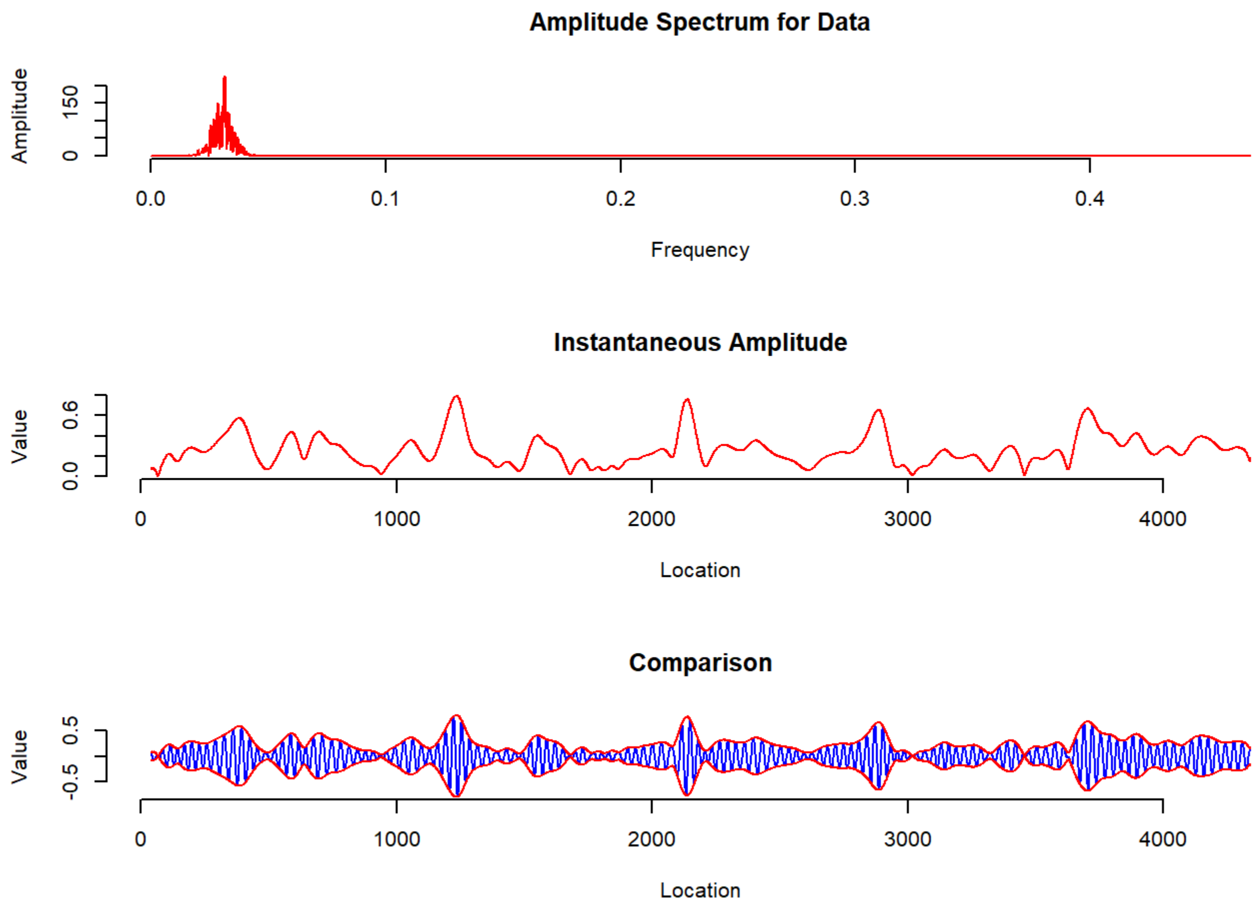


```
#Filter out the 173 kyr cycle from the amplitude modulation of the obliquity cycle
alb_Ti_time_obl_hilbert_173 <-taner(alb_Ti_time_obl_hilbert,
                                    xmax=1/100,
                                    fhigh=1/195,
                                    flow=1/155,
                                    detrend=TRUE
                                    )
```

```
----- TANER BANDPASS FILTERING STRATIGRAPHIC SERIES-----
 * Number of data points= 4031
 * Sample interval= 1.067338
 * Mean value removed= 0.2578766
 * Linear trend removed. m= 5.15436e-06 b= -0.01128923
```

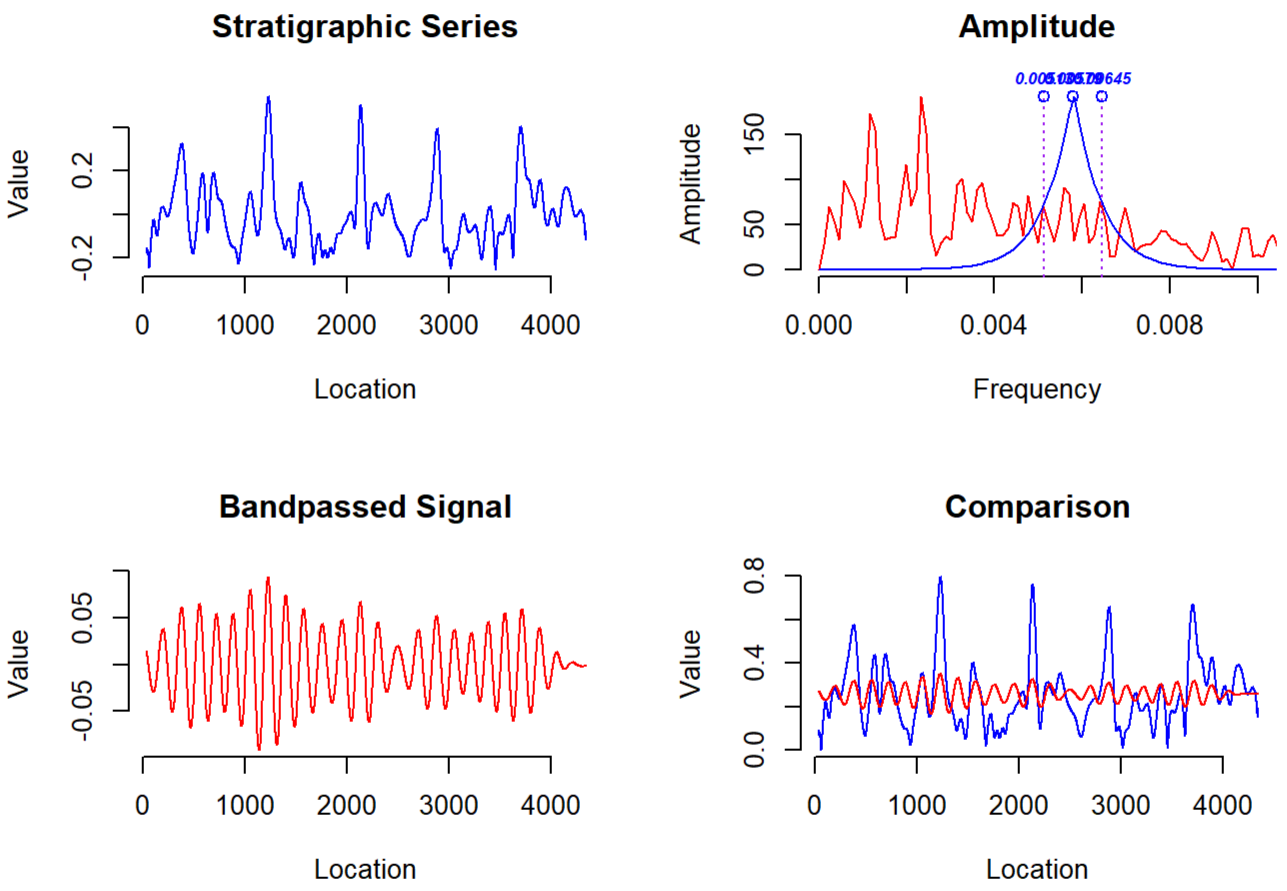


```
#Filter out the 173 kyr obliquity cycle directly from the proxy record
alb_Ti_time_173 <- taner(alb_Ti_time,
                         xmax=1/50,
                         fhigh=1/195,
                         flow=1/155,
                         demean=T
                         )
```

```
----- TANER BANDPASS FILTERING STRATIGRAPHIC SERIES-----
 * Number of data points= 4031
 * Sample interval= 1.067338
 * Mean value removed= 0.0002517748
```

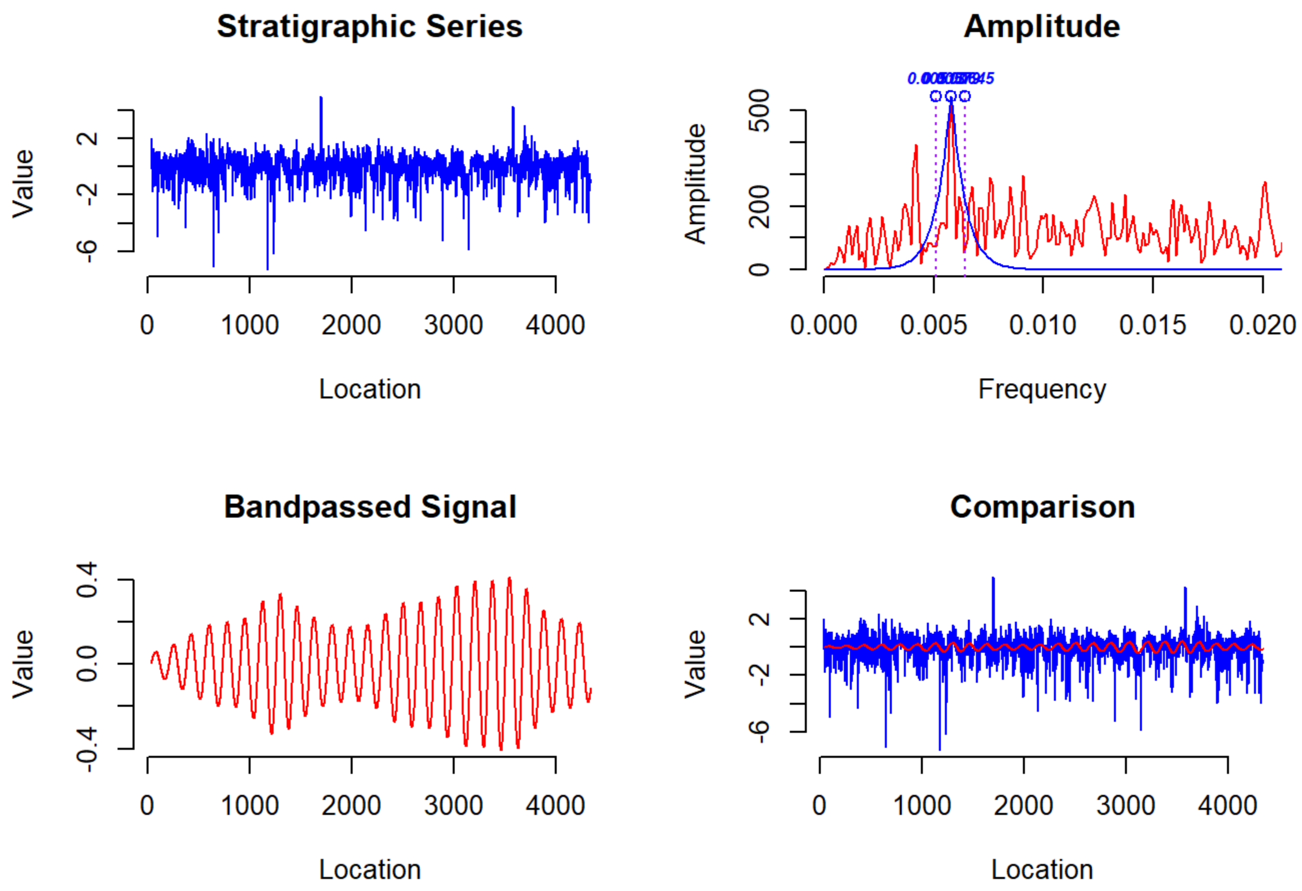


```
#Plot the 173 kyr obliquity cycle extracted from the amplitude modulation of the
#obliquity cycle vs the 173 kyr obliquity cycle directly extracted from the proxy
#record.

plot(alb_Ti_time_173[,1],
     alb_Ti_time_173[,2]-mean(alb_Ti_time_173[,2]),
     type="l",
     xlab ="Time (kyr)",
     ylab = "Power"
     )

lines(alb_Ti_time_obl_hilbert_173[,1],
      alb_Ti_time_obl_hilbert_173[,2]-mean(alb_Ti_time_obl_hilbert_173[,2]),
      col="red",
      lwd=2
      )
```

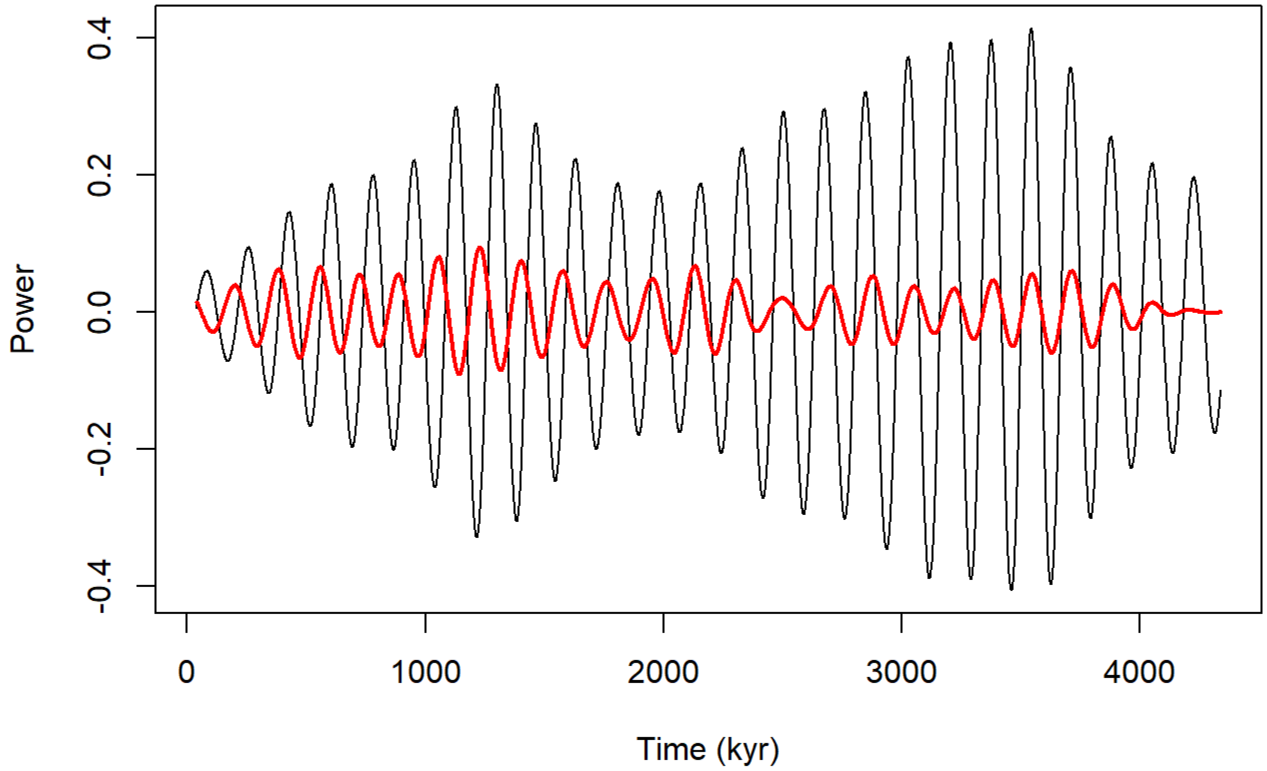


---

# Step 10: Hilbert transform analysis between the 173 kyr and the 1.2 Myr cycles

## 10.1. Tuning of the 1mm non-detrended Ti series

Same tuning procedure as for step 7 but on the non resampled and non detrended Ti dataset

```
#tuning of the non detrended 1mm Ti series
Ti_1mm_173R <- taner(alb_Ti_1mm, xmax=2, fhigh=1/0.97, flow=1/0.76, demean=T)
```

```
----- TANER BANDPASS FILTERING STRATIGRAPHIC SERIES-----
 * Number of data points= 20158
 * Sample interval= 0.001
 * Mean value removed= -0.03216273
```

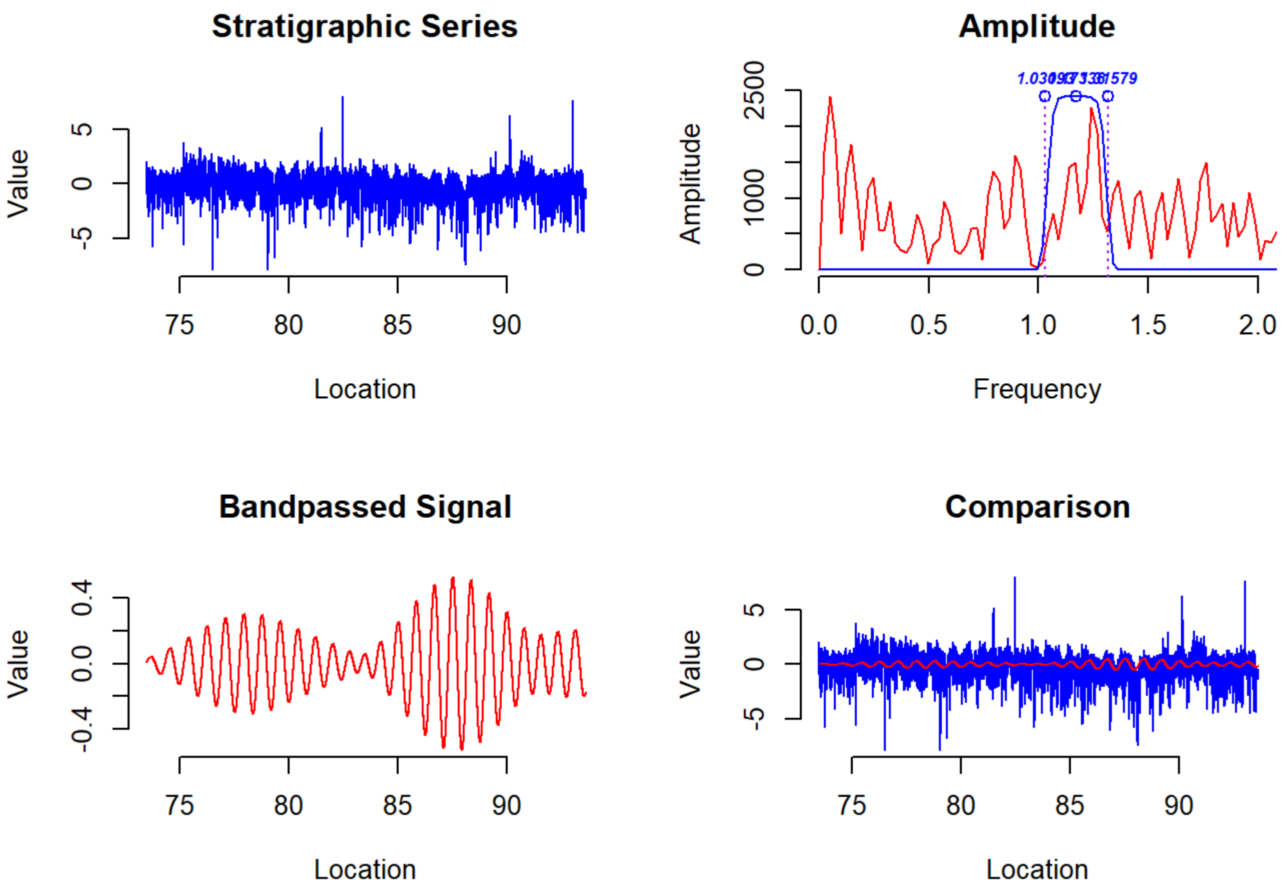


```
Ti_1mm_173_min <- trough(Ti_1mm_173R, level = 200, genplot = T)
```

```
----- FINDING MINIMA OF TROUGHS, FILTERING AT THRESHOLD VALUE -----
 * Number of data points= 20158
 * Number of columns= 2
 * Identifying minima of troughs
 * Number of troughs identified= 25
 * Filtering troughs at threshold of 200
 * Number of troughs <= 200 : 25
```

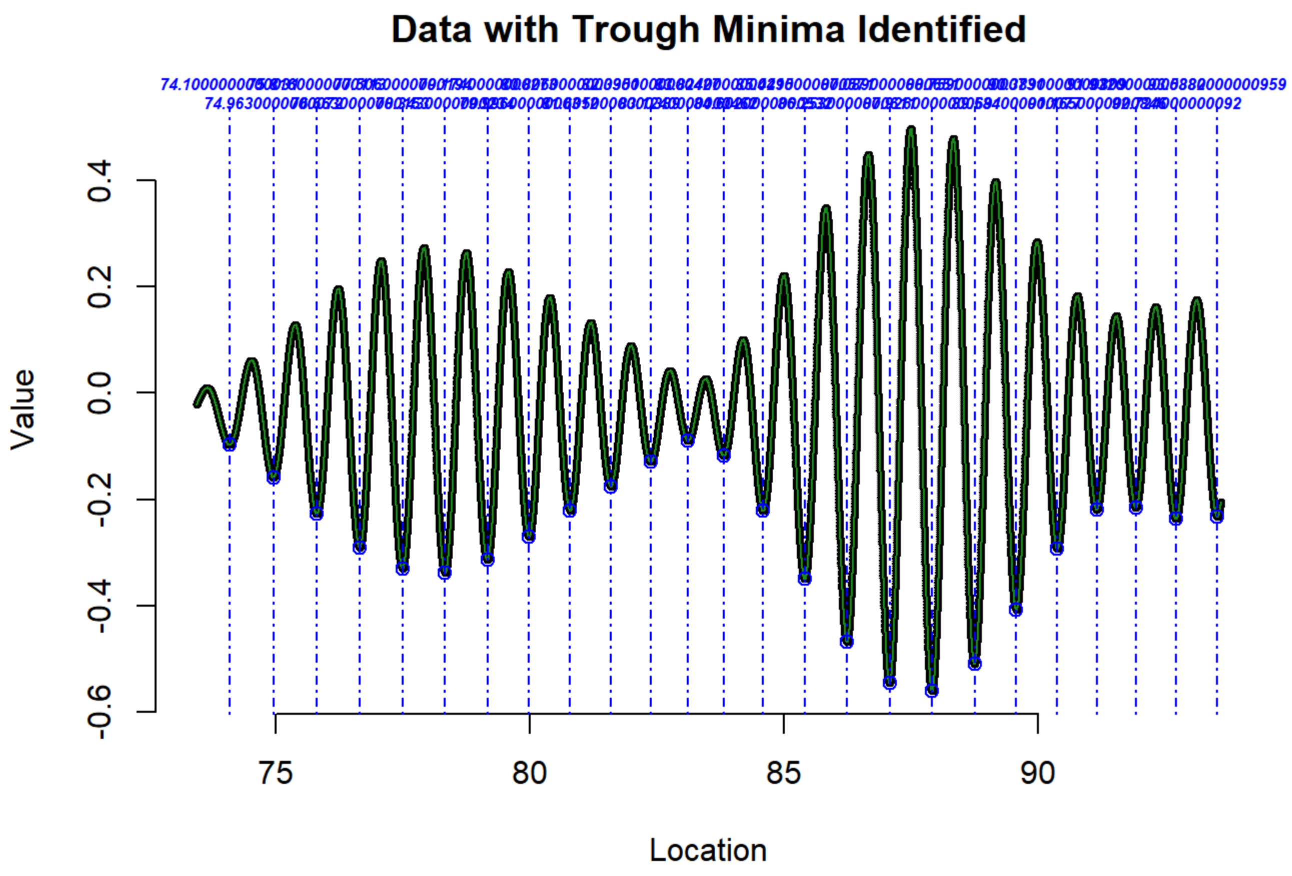


```
Ti_1mm_173_min <- Ti_1mm_173_min[,c(2,3)]

#write.csv(Ti_1mm_173_min,"Ti_1mm_173_minTan_0.76-0.97m.csv")

#Before continuing, go to the .csv file and for each location, add a multiple of
#173 for the trough value as explain in step 7.3.

Ti_1mm_173_min <- read.csv("Ti_1mm_173_minTan_0.76-0.97m.csv", sep=";")

Ti_1mm_173_tuned <- tune(alb_Ti_1mm,
                         Ti_1mm_173_min,
                         extrapolate=T,
                         genplot=T,
                         check=T,
                         verbose=T
                         )
```

```
----- TUNING STRATIGRAPHIC SERIES -----
 * Number of data points= 20158
 * Number of time control points= 25
 * Sorting datasets into ensure increasing order, removing empty entries

 * Mean sampling interval= 0.2131761
 * Median sampling interval= 0.2104623
 * Maximum sampling interval= 0.2471429
 * Minimum sampling interval= 0.2004635
```

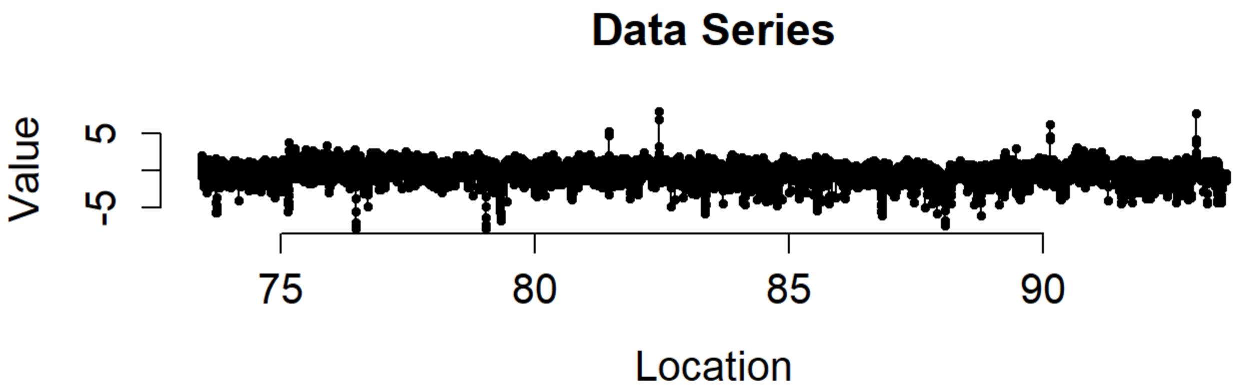


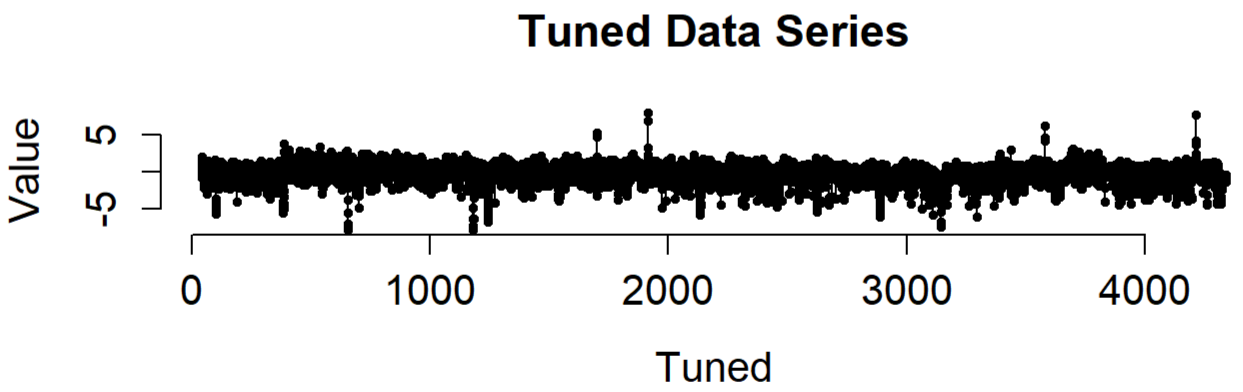


```
#Interpolation every 0.2131761 kyr (= mean sampling interval)
alb_Ti_1mm_time <- linterp(Ti_1mm_173_tuned, dt=0.2131761, genplot=T)
```

```
----- APPLYING PIECEWISE-LINEAR INTERPOLATION TO STRATIGRAPHIC SERIES -----

 * Number of samples= 20158
 * New number of samples= 20157
```

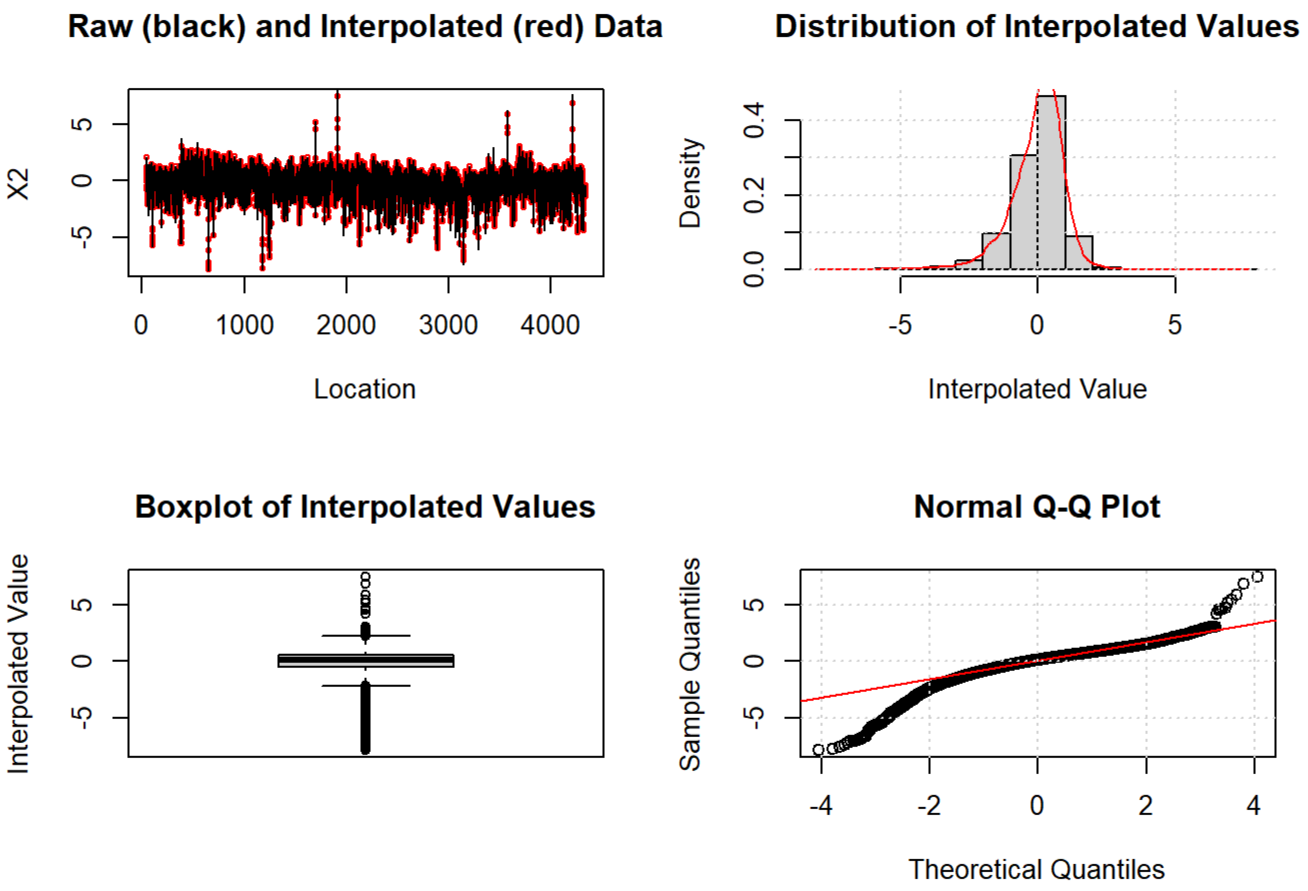


## 10.2. Hilbert analysis

```
#Filter out the 173 kyr long obliquity cycle
alb_Ti_1mm_time_173 <- taner(alb_Ti_1mm_time, xmax=1/100, fhigh=1/155, flow=1/195)
```

```
----- TANER BANDPASS FILTERING STRATIGRAPHIC SERIES-----
 * Number of data points= 20157
 * Sample interval= 0.2131761
 * Mean value removed= -0.03228278
```

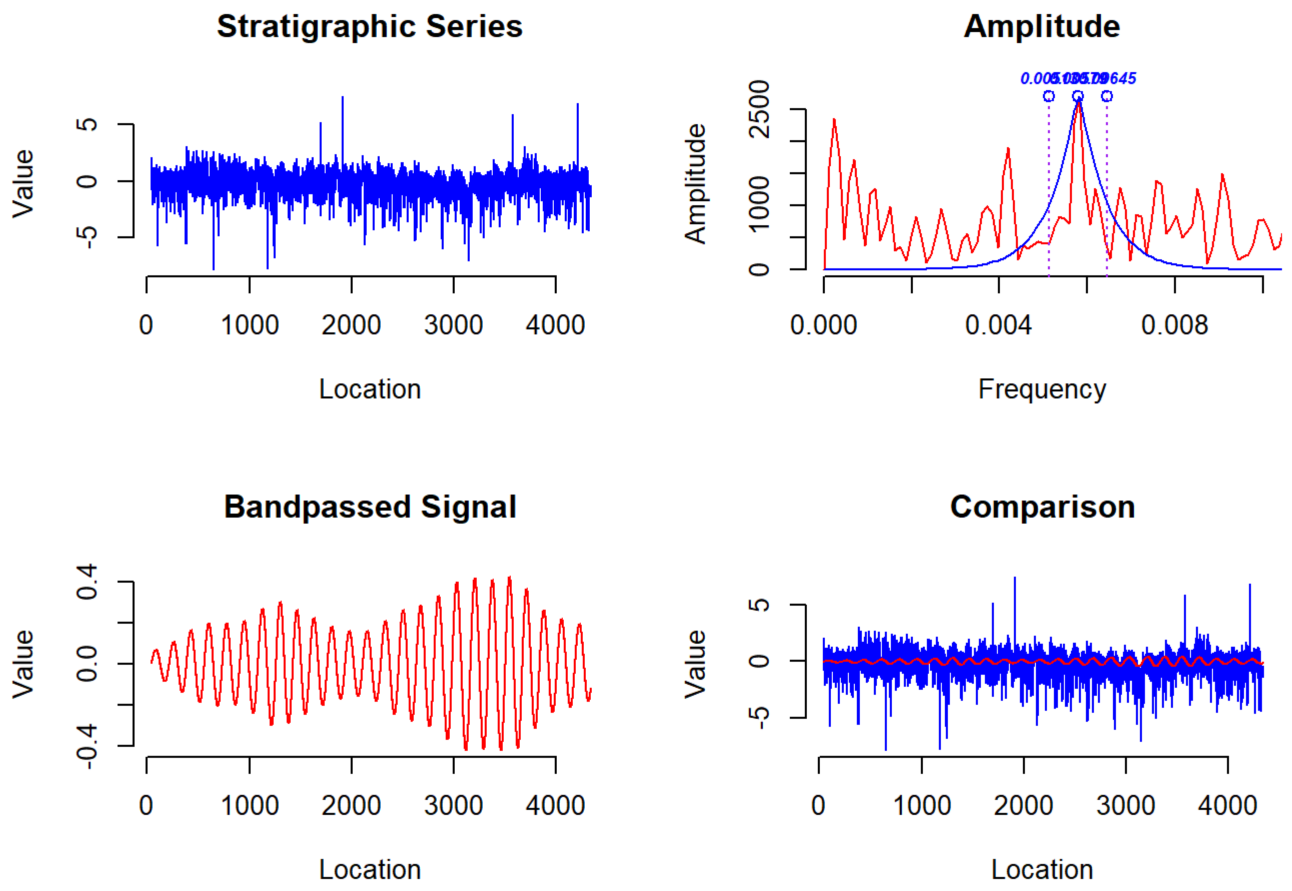


```r
#Do the Hilbert transform to extract the amplitude
alb_Ti_1mm_time_173_hilbert <- hilbert(alb_Ti_1mm_time_173)
```

```
----- PERFORMING HILBERT TRANSFORM ON STRATIGRAPHIC SERIES -----
 * Number of data points= 20157
 * Sample interval= 0.2131761
 * Mean value removed= -0.03265143
```

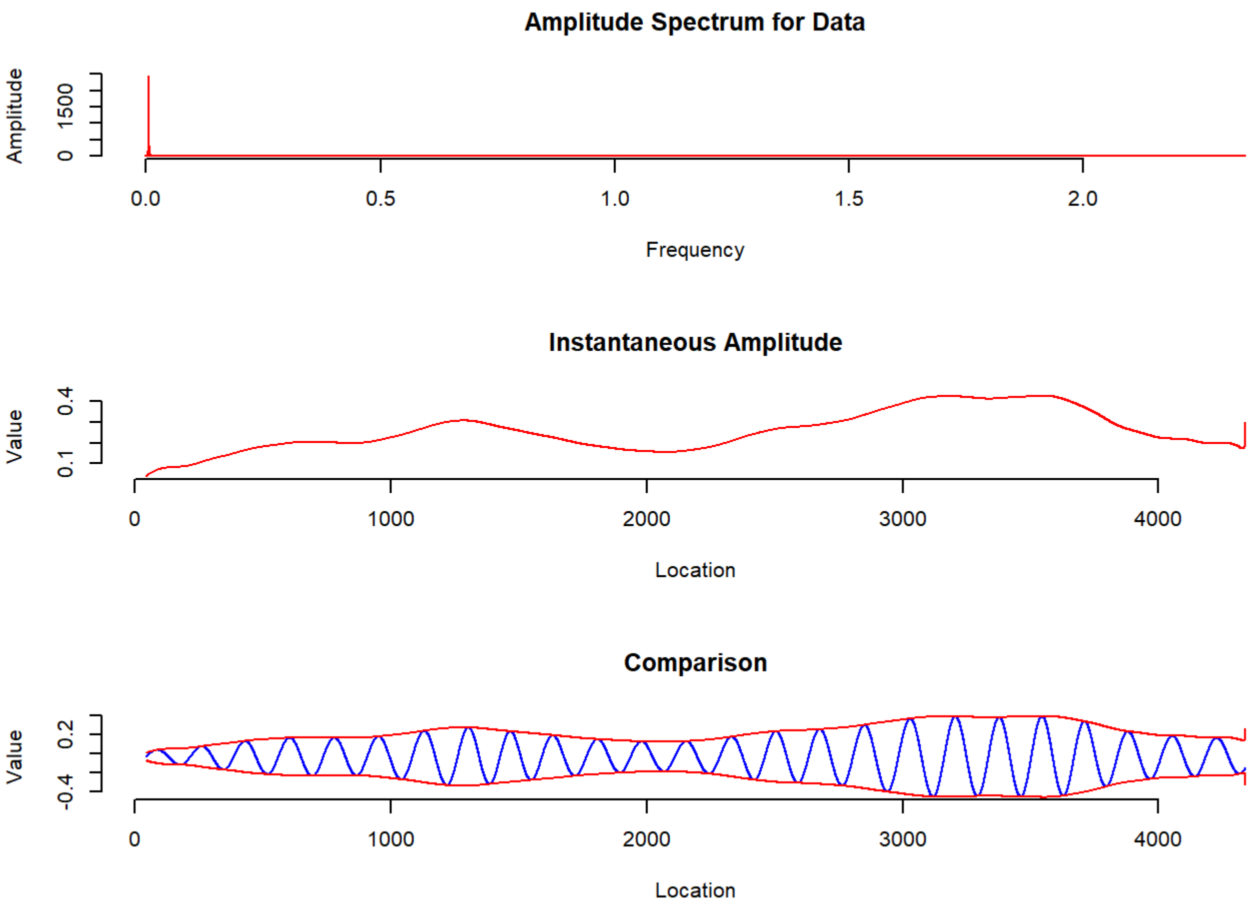


```
#Filter out the 1.2 Myr cycle from the amplitude modulation of the 173 kyr cycle
alb_Ti_1mm_time_173_hilbert_1300 <- taner(alb_Ti_1mm_time_173_hilbert,
                                          xmax=1/500,
                                          fhigh=1/1300,
                                          flow=1/1150,
                                          detrend=T
                                          )
```

```
----- TANER BANDPASS FILTERING STRATIGRAPHIC SERIES-----
 * Number of data points= 20157
 * Sample interval= 0.2131761
 * Mean value removed= 0.2545402
 * Linear trend removed. m= 4.437838e-05 b= -0.09725471
```

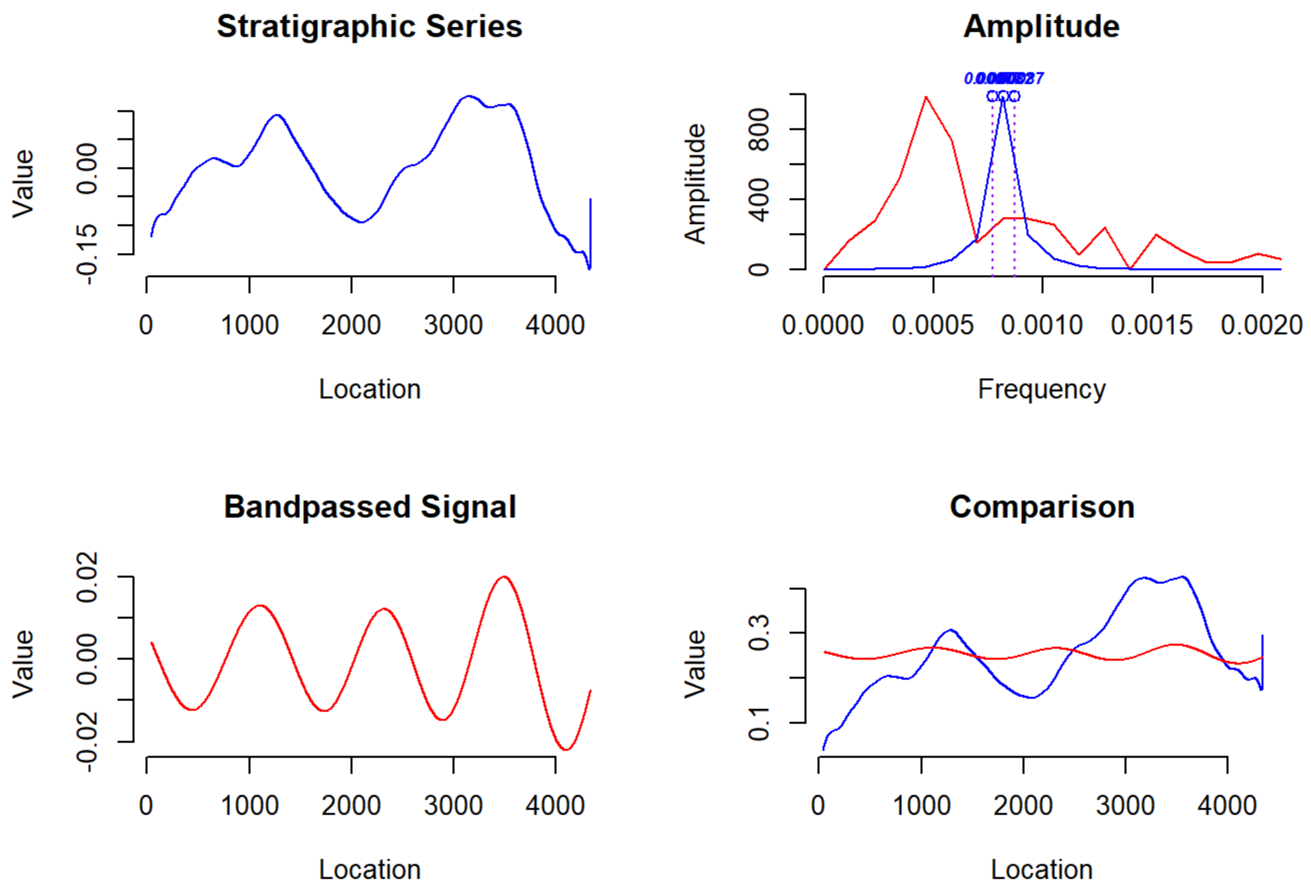


```
#Filter out the 1.2 Myr obliquity cycle directly from the proxy record
alb_Ti_1mm_time_1300 <- taner(alb_Ti_1mm_time,
                              xmax=1/500,
                              fhigh=1/1300,
                              flow=1/1150,
                              demean=T
                              )
```

```
----- TANER BANDPASS FILTERING STRATIGRAPHIC SERIES-----
 * Number of data points= 20157
 * Sample interval= 0.2131761
 * Mean value removed= -0.03228278
```

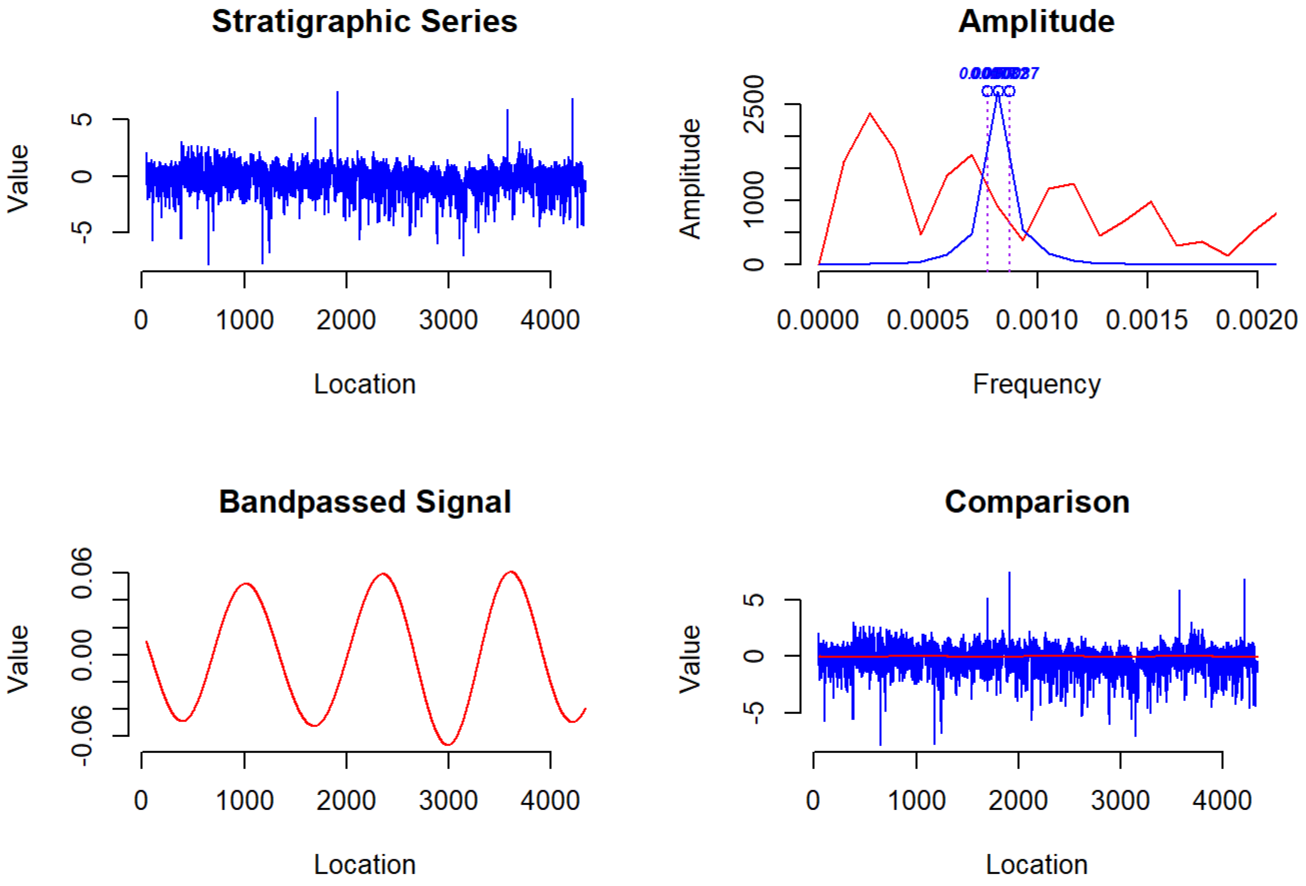


```
#Plot the 1.2 Myr obliquity cycle extracted from the amplitude modulation of the
#173 kyr long obliquity cycle vs the 1.2 Myr obliquity cycle directly extracted
#from the proxy record.

plot(alb_Ti_1mm_time_1300[,1],
     alb_Ti_1mm_time_1300[,2]-mean(alb_Ti_1mm_time_1300[,2]),
     type="l",
     xlab = "Time (kyr)",
     ylab = "Power")

lines(alb_Ti_1mm_time_173_hilbert_1300[,1],
      alb_Ti_1mm_time_173_hilbert_1300[,2]-mean(alb_Ti_1mm_time_173_hilbert_1300[,2]),
      col="red",
      lwd=2
      )
```

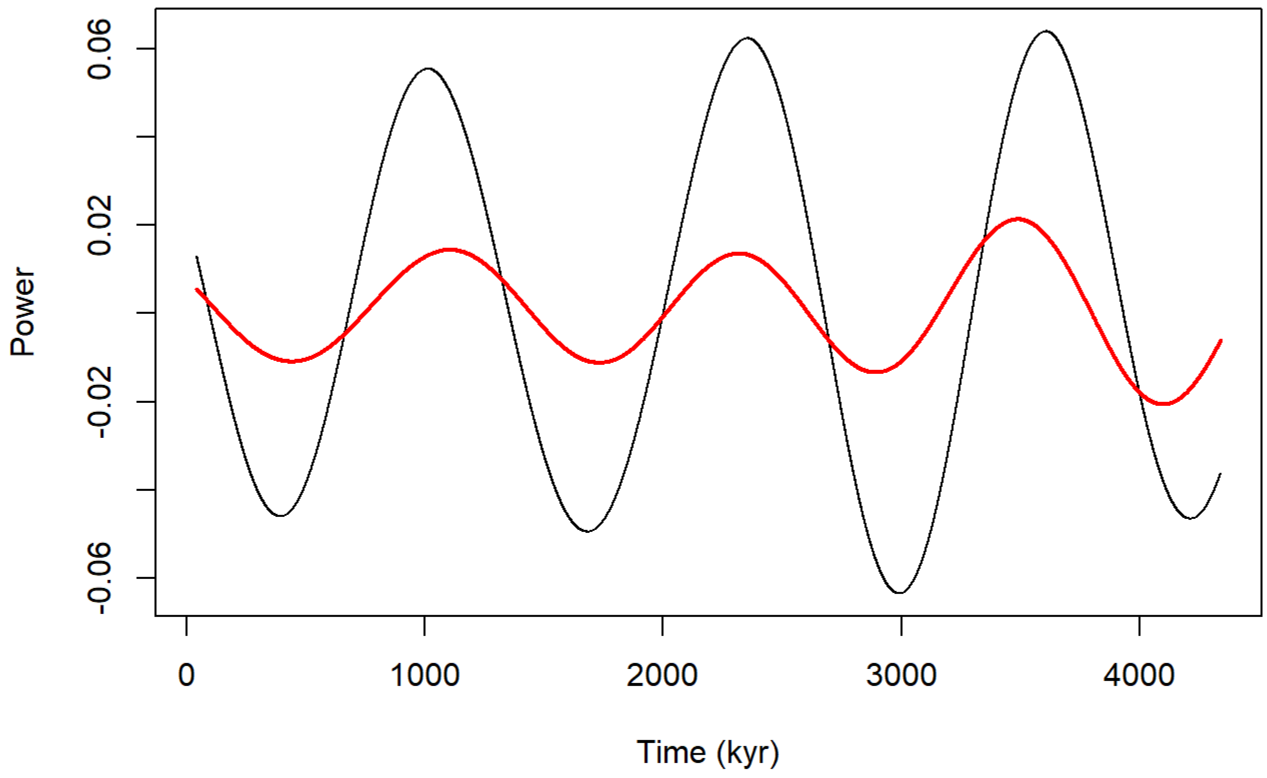


---

# Step 11: Age-depth model and sedimentation rate based on 4 proxy comparison

## 11.1. Age-Depth model (bandpass filtering)

### 11.1.1. Minimal_tuning function

```
minimal_tuning <- function (data = NULL, pts = 5, cycle = 173, tune_opt = "max",
                            output = 0, genplot = FALSE, keep_editable = FALSE)
{
  astro_mindetect <- as.data.frame(data)
  astro_mindetect$min <- 0
  for (i in pts:(nrow(data) - pts)) {
    if ((data[i, 2] - data[(i + pts), 2] < 0) & (data[i,
                                                       2] - data[(i - (pts - 1)), 2] < 0)) {
      astro_mindetect[i, 3] <- 1
    }
  }
  astro_mindetect_error_corr <- astro_mindetect
  astro_mindetect_error_corr <- astro_mindetect_error_corr[astro_mindetect_error_corr$min ==
                                                              1, ]
  astro_maxdetect <- as.data.frame(data)
```

```
  astro_maxdetect$max <- 0
  for (i in pts:(nrow(data) - pts)) {
    if ((data[i, 2] - data[(i + pts), 2] > 0) & (data[i,
                                                       2] - data[(i - (pts - 1)), 2] > 0)) {
      astro_maxdetect[i, 3] <- 1
    }
  }
  astro_maxdetect_error_corr <- astro_maxdetect
  astro_maxdetect_error_corr <- astro_maxdetect_error_corr[astro_maxdetect_error_corr$max ==
                                                             1, ]
  max <- astro_maxdetect_error_corr
  colnames(max) <- c("A", "B", "C")
  min <- astro_mindetect_error_corr
  colnames(min) <- c("A", "B", "C")
  min[, 3] <- -1
  peaks <- rbind(max, min)
  peaks <- peaks[order(peaks[, 1]), ]
  i <- 1
  res_rownr <- nrow(peaks)
  while (i < res_rownr) {
    if ((i < res_rownr) & (peaks[i, 3] == peaks[(i + 1),
                                                3])) {
      if ((i < res_rownr) & (peaks[i, 3] == 1 & peaks[(i +
                                                       1), 3] == 1) & (peaks[i, 2] > peaks[(
                                                                                          2
        peaks[(i + 1), ] <- NA
        peaks <- na.omit(peaks)
        res_rownr <- res_rownr - 1
      }
      if ((i < res_rownr) & (peaks[i, 3] == 1 & peaks[(i +
                                                       1), 3] == 1) & (peaks[i, 2] < peaks[(
                                                                                          2
        peaks[i, ] <- NA
        peaks <- na.omit(peaks)
        res_rownr <- res_rownr - 1
      }
      if ((i < res_rownr) & (peaks[i, 3] == -1 & peaks[(i +
                                                        1), 3] == -1) & (peaks[i, 2] < peaks

        peaks[(i + 1), ] <- NA
        peaks <- na.omit(peaks)
        res_rownr <- res_rownr - 1
      }
      if ((i < res_rownr) & (peaks[i, 3] == -1 & peaks[(i +
                                                        1), 3] == -1) & (peaks[i, 2] > peaks

        peaks[i, ] <- NA
        peaks <- na.omit(peaks)
        res_rownr <- res_rownr - 1
      }
    }
    if ((peaks[i, 3] != peaks[(i + 1), 3]) | is.na(peaks[i,
                                                         3] != peaks[(i + 1), 3])) {
      i <- i + 1
```

```
    }
  }
  if (tune_opt == "min") {
    peaks_min <- peaks[peaks[, 3] < 0, ]
    dist <- peaks_min[2:(nrow(peaks_min)), ] - peaks_min[1:(nrow(peaks_min) -
                                                          1), ]
    sed_rate <- (dist[, 1] * 100)/cycle
    sed_rate <- cbind(sed_rate, peaks_min[1:(nrow(peaks_min) -
                                              1), 1], peaks_min[2:(nrow(peaks_min)), 1])
  }
  if (tune_opt == "max") {
    peaks_min <- peaks[peaks[, 3] > 0, ]
    dist <- peaks_min[2:(nrow(peaks_min)), ] - peaks_min[1:(nrow(peaks_min) -
                                                          1), ]
    sed_rate <- (dist[, 1] * 100)/cycle
    sed_rate <- cbind(sed_rate, peaks_min[1:(nrow(peaks_min) -
                                              1), 1], peaks_min[2:(nrow(peaks_min)), 1])
  }
  if (tune_opt == "minmax") {
    peaks_min <- peaks
    dist <- peaks_min[2:(nrow(peaks_min)), ] - peaks_min[1:(nrow(peaks_min) -
                                                          1), ]
    sed_rate <- (dist[, 1] * 100)/(cycle/2)
    sed_rate <- cbind(sed_rate, peaks_min[1:(nrow(peaks_min) -
                                              1), 1], peaks_min[2:(nrow(peaks_min)), 1])
  }
  top <- c(sed_rate[1, 1], data[1, 1], sed_rate[1, 2])
  bot <- c(sed_rate[nrow(sed_rate), 1], sed_rate[nrow(sed_rate),
                                                  3], data[nrow(data), 1])
  sed_rate <- rbind(top, sed_rate, bot)
  data[, 3] <- NA
  p <- 1
  for (i in 1:nrow(data)) {
    if (data[i, 1] < sed_rate[p, 2]) {
      data[i, 3] <- sed_rate[p, 1]
    }
    if (data[i, 1] == sed_rate[p, 2] & p + 1 <= nrow(sed_rate)) {
      data[i, 3] <- (sed_rate[p, 1] + sed_rate[(p + 1),
                                              1])/2
    }
    if (p > nrow(sed_rate)) {
      p <- nrow(sed_rate)
    }
    if (p == nrow(sed_rate)) {
      data[i, 3] <- sed_rate[nrow(sed_rate), 1]
    }
    if (data[i, 1] > sed_rate[p, 2]) {
      data[i, 3] <- sed_rate[p, 1]
    }
    if (data[i, 1] == sed_rate[p, 3] & p + 1 <= nrow(sed_rate)) {
      p <- p + 1
    }
  }
  tracked_cycle_curve <- data[, c(1, 3)]
```

```r
sedrates <- data.frame(tracked_cycle_curve)
dat <- as.matrix(tracked_cycle_curve)
dat <- na.omit(dat)
dat <- dat[order(dat[, 1], na.last = NA, decreasing = F),
]
npts <- length(dat[, 1])
start <- dat[1, 1]
end <- dat[length(dat[, 1]), 1]
x1 <- dat[1:(npts - 1), 1]
x2 <- dat[2:(npts), 1]
dx = x2 - x1
dt = median(dx)
xout <- seq(start, end, by = dt)
npts <- length(xout)
interp <- approx(dat[, 1], dat[, 2], xout, method = "linear",
                 n = npts)
sedrates <- as.data.frame(interp)
npts <- length(sedrates[, 1])
sedrates[1] = sedrates[1] * 100
sedrates[2] = 1/sedrates[2]
dx = sedrates[2, 1] - sedrates[1, 1]
midptx = (sedrates[2:npts, 1] + sedrates[1:(npts - 1), 1])/2
slope = (sedrates[2:npts, 2] - sedrates[1:(npts - 1), 2])/dx
yint = sedrates[2:npts, 2] - (slope * sedrates[2:npts, 1])
midpty = (slope * midptx) + yint
hsum = cumsum(midpty * dx)
hsum = append(0, hsum)
out = data.frame(cbind(sedrates[, 1]/100, hsum))
data[, 4] <- out[, 2]
colnames(data) <- c("depth", "proxy", "cm/kyr", "time")
if (output == 0) {
  data <- data
  if (genplot == TRUE) {
    if (keep_editable == FALSE) {
      oldpar <- par(no.readonly = TRUE)
      on.exit(par(oldpar))
    }
    layout.matrix <- matrix(c(1, 2, 3, 4), nrow = 4,
                            ncol = 1)
    graphics::layout(mat = layout.matrix, heights = c(1),
                     widths = c(1))
    par(mar = c(4, 4, 1, 1))
    plot(x = data[, 1], y = data[, 2], type = "l", main = "Data depth domain",
         xlab = "meters", ylab = "proxy")
    plot(x = data[, 1], y = data[, 3], type = "l", xlab = "meters",
         ylab = "cm/kyr (ka)", main = "sedimentation rate plot")
    plot(data[, 1], data[, 4], type = "l", xlab = "meters",
         ylab = "Time (ka)", main = "Data time domain")
    plot(data[, 4], data[, 2], type = "l", xlab = "time (ka)",
         ylab = "proxy", main = "Data time domain")
  }
}
if (output == 1) {
  data <- data[, c(1, 3)]
```

```
    if (genplot == TRUE) {
      if (keep_editable == FALSE) {
        oldpar <- par(no.readonly = TRUE)
        on.exit(par(oldpar))
      }
      layout.matrix <- matrix(c(1), nrow = 1, ncol = 1)
      graphics::layout(mat = layout.matrix, heights = c(1),
                       widths = c(1))
      par(mar = c(4, 4, 1, 1))
      plot(x = data[, 1], y = data[, 2], type = "l", xlab = "meters",
           ylab = "cm/kyr (ka)", main = "sedimentation rate plot")
    }
  }
  if (output == 2) {
    data <- data[, c(1, 4)]
    if (genplot == TRUE) {
      if (keep_editable == FALSE) {
        oldpar <- par(no.readonly = TRUE)
        on.exit(par(oldpar))
      }
      layout.matrix <- matrix(c(1), nrow = 1, ncol = 1)
      graphics::layout(mat = layout.matrix, heights = c(1),
                       widths = c(1))
      par(mar = c(4, 4, 1, 1))
      plot(data[, 1], data[, 2], type = "l", xlab = "meters",
           ylab = "Time (ka)", main = "Data time domain")
    }
  }
  return(data)
}
```

## 11.1.2. Taner filtering of the detrital elements in depth domain

### 11.1.2.1. Titanium

```
alb_Ti <- cbind(Alb$DepthAdj,Alb$Ti)
alb_Ti <- na.omit(alb_Ti)
alb_Ti[!is.finite(alb_Ti)] <- NA
alb_Ti <- na.omit(alb_Ti)
alb_Ti <- iso(dat=alb_Ti, xmin=73.453, xmax=93.609)
```

```
----- ISOLATE STRATIGRAPHIC DATA BY LOCATION -----
 * Number of data points= 30577
 * Number of columns= 2
 * Minimum= 62.111 , Maximum= 93.61
 * Isolating data between 73.453 and 93.609
 * Number of data points following culling= 19310
```

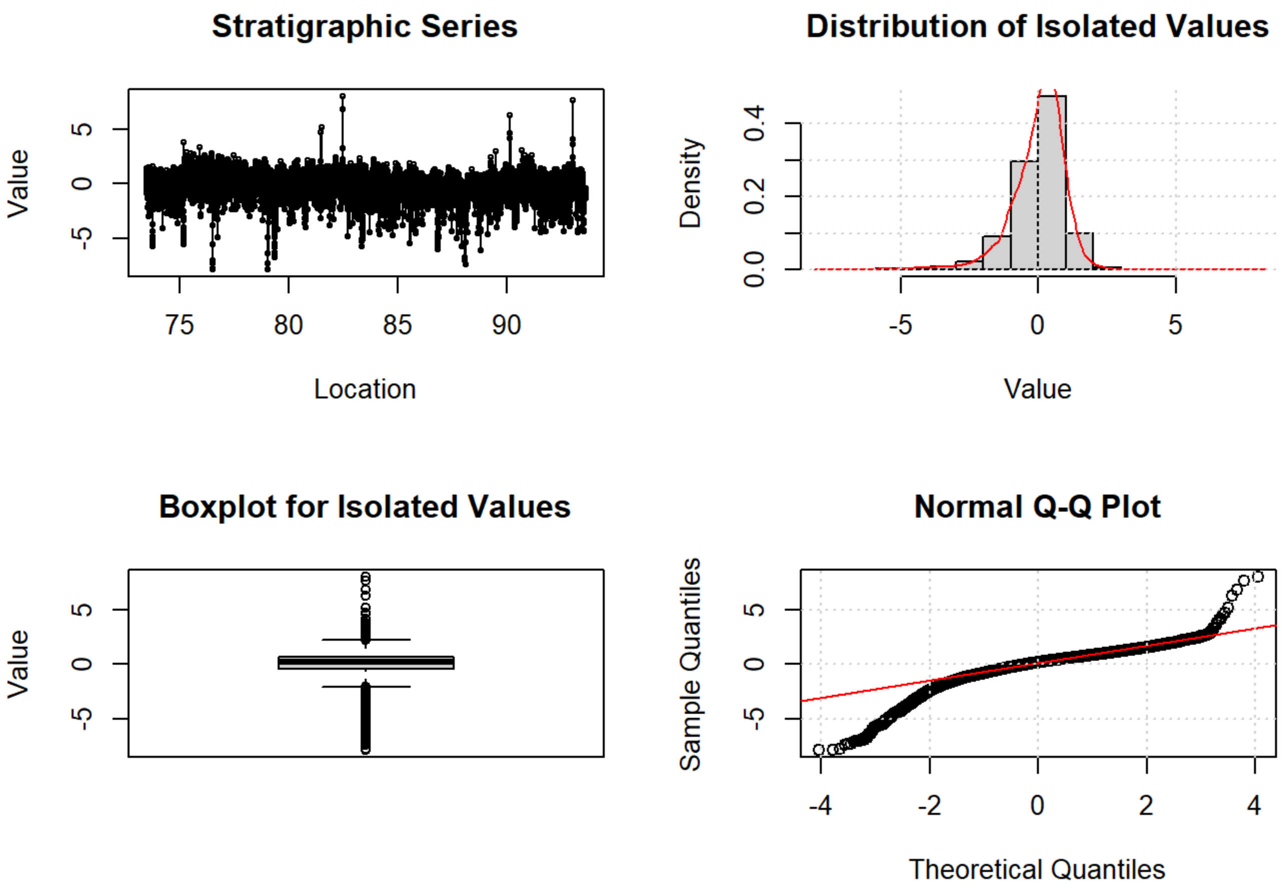


```r
alb_Ti <- linterp(alb_Ti, dt=0.005, genplot=T)
```

```
----- APPLYING PIECEWISE-LINEAR INTERPOLATION TO STRATIGRAPHIC SERIES -----

 * Number of samples= 19310
 * New number of samples= 4032
```

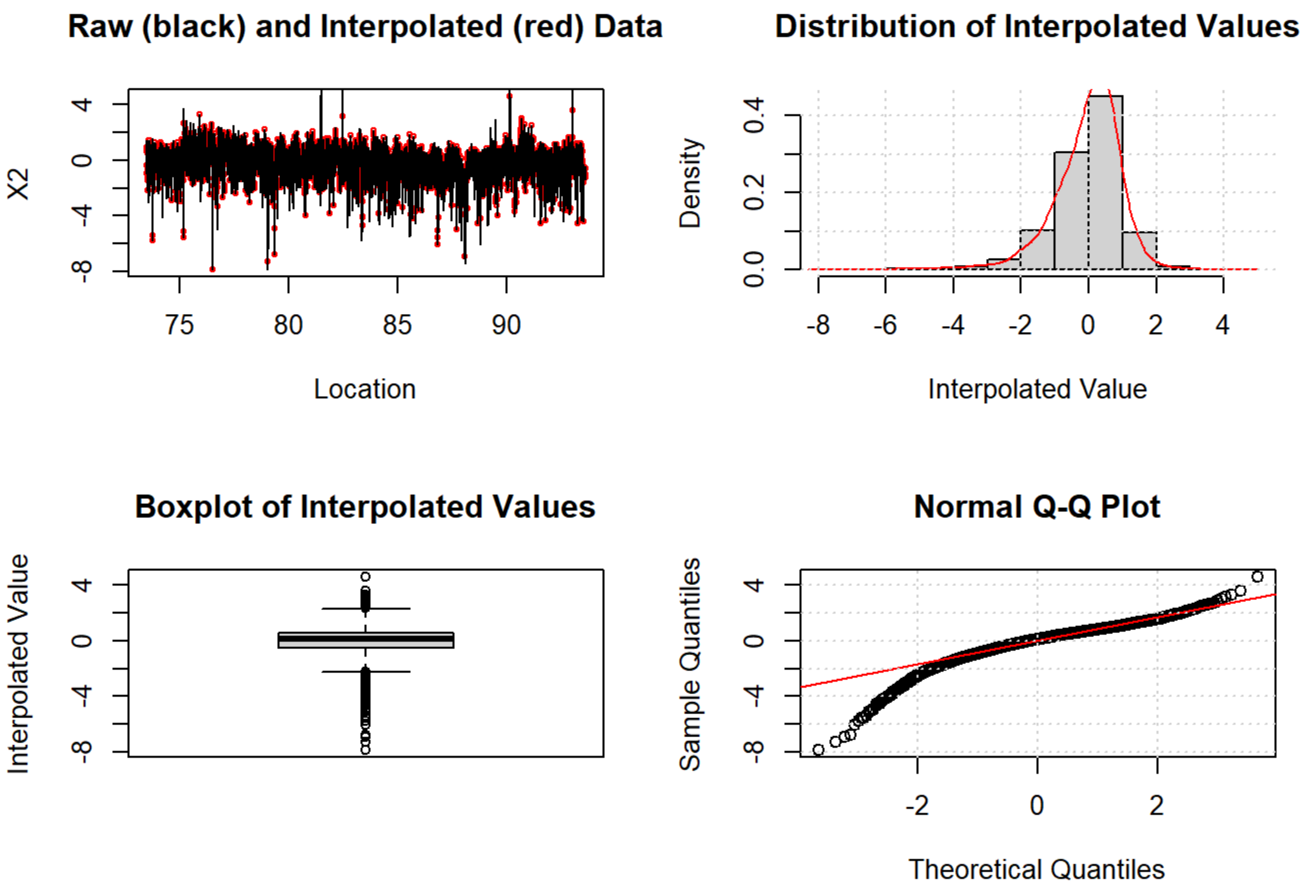


```r
Ti_173R <- taner(alb_Ti,xmax=2,fhigh=1/0.97,flow=1/0.75,demean=TRUE)
```

```
----- TANER BANDPASS FILTERING STRATIGRAPHIC SERIES-----
 * Number of data points= 4032
 * Sample interval= 0.005
 * Mean value removed= -0.04021631
```

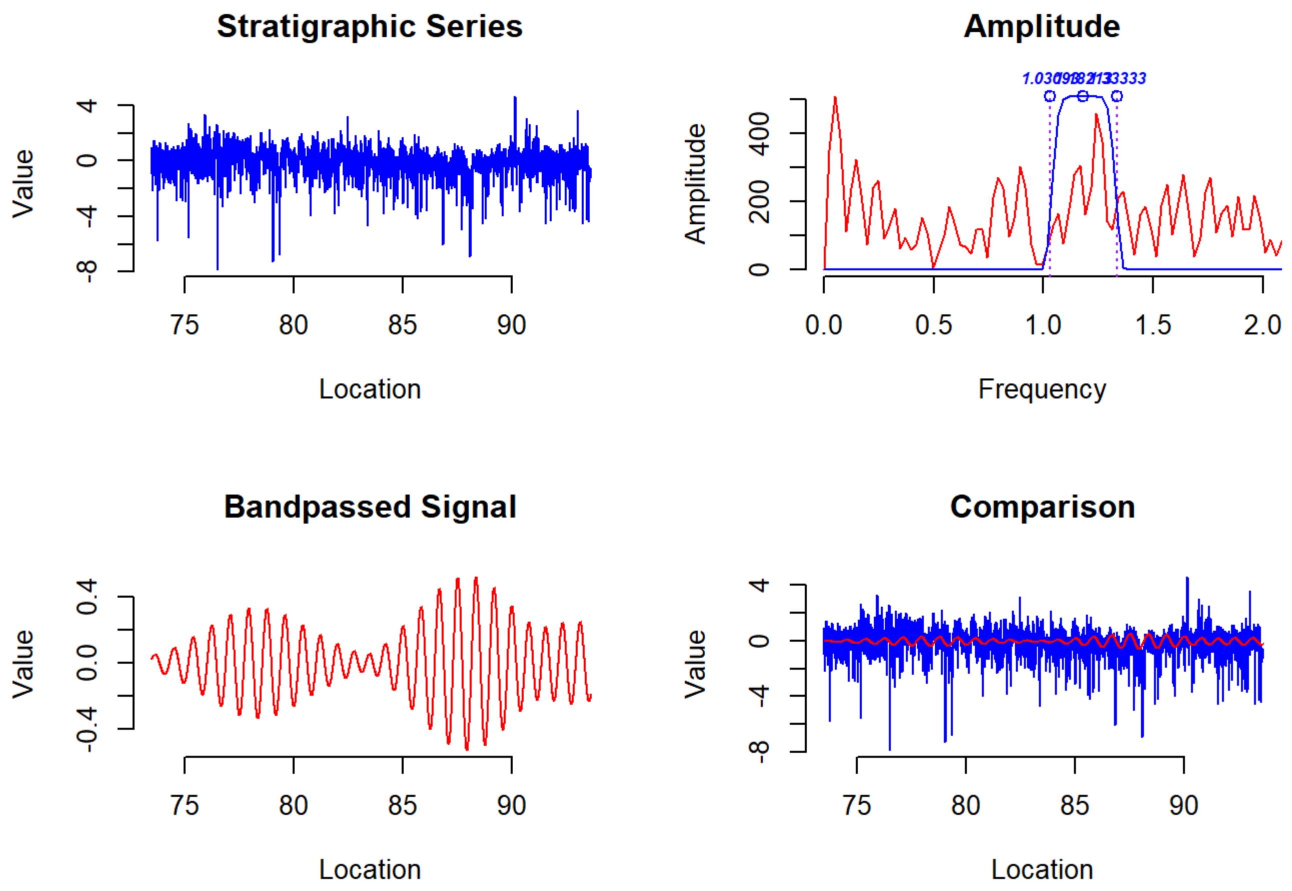


```r
Ti_173R_min_tun <-    minimal_tuning(data = Ti_173R,
                          pts = 20,
                          cycle = 173,
                          tune_opt = "min",
                          output = 0,
                          genplot = TRUE,
                          keep_editable = FALSE
                          )
```

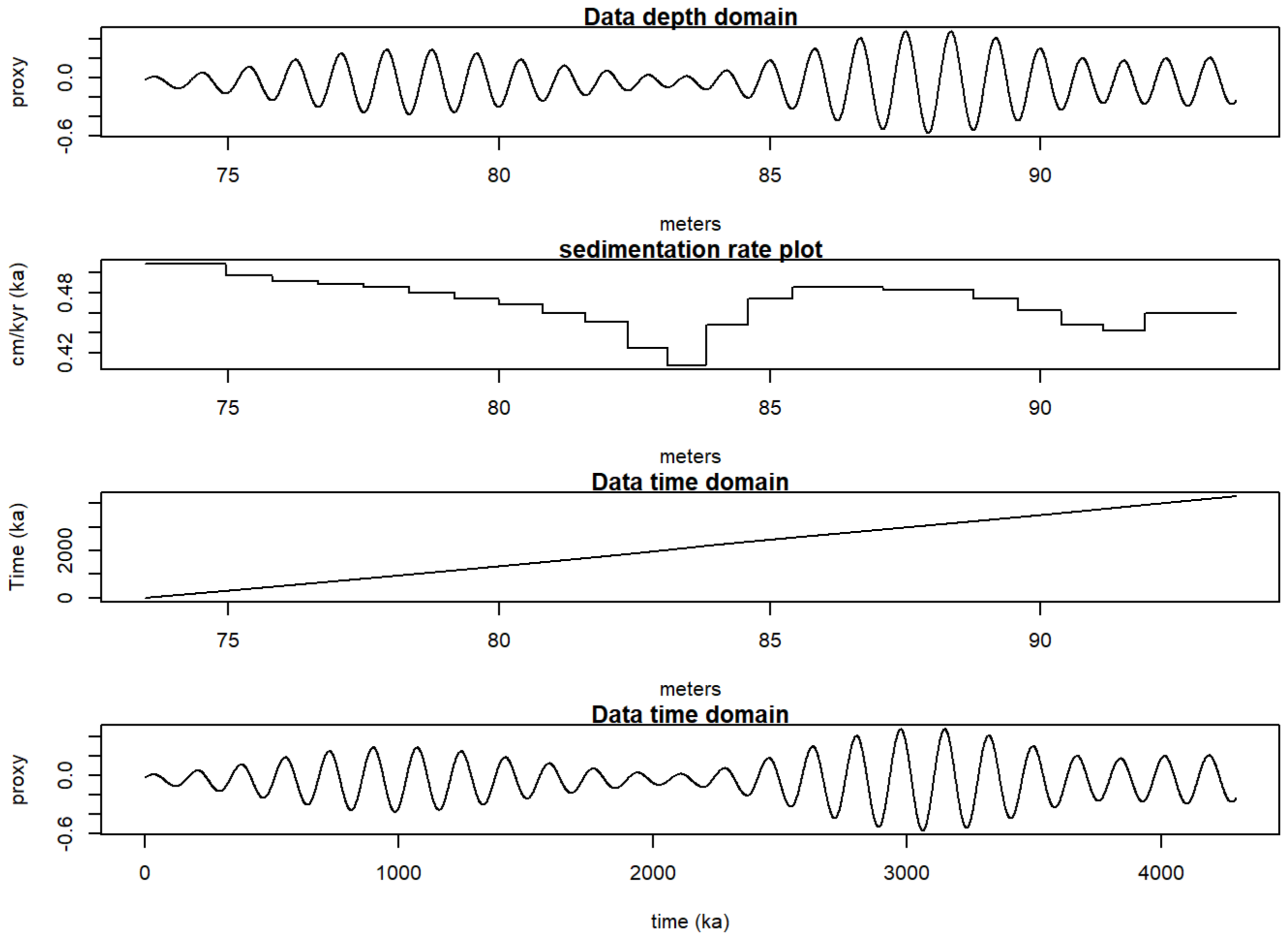


```
colnames(Ti_173R_min_tun)<-c("Ti Depth","Ti","Ti SR","Ti time")
```

### 11.1.2.2. Silicon

```
alb_Si <- cbind(Alb$DepthAdj,Alb$Si)
alb_Si <- na.omit(alb_Si)
alb_Si[!is.finite(alb_Si)] <- NA
alb_Si <- na.omit(alb_Si)
alb_Si <- iso(dat=alb_Si, xmin=73.453, xmax=93.609)
```

```
----- ISOLATE STRATIGRAPHIC DATA BY LOCATION -----
 * Number of data points= 30577
 * Number of columns= 2
 * Minimum= 62.111 , Maximum= 93.61
 * Isolating data between 73.453 and 93.609
 * Number of data points following culling= 19310
```

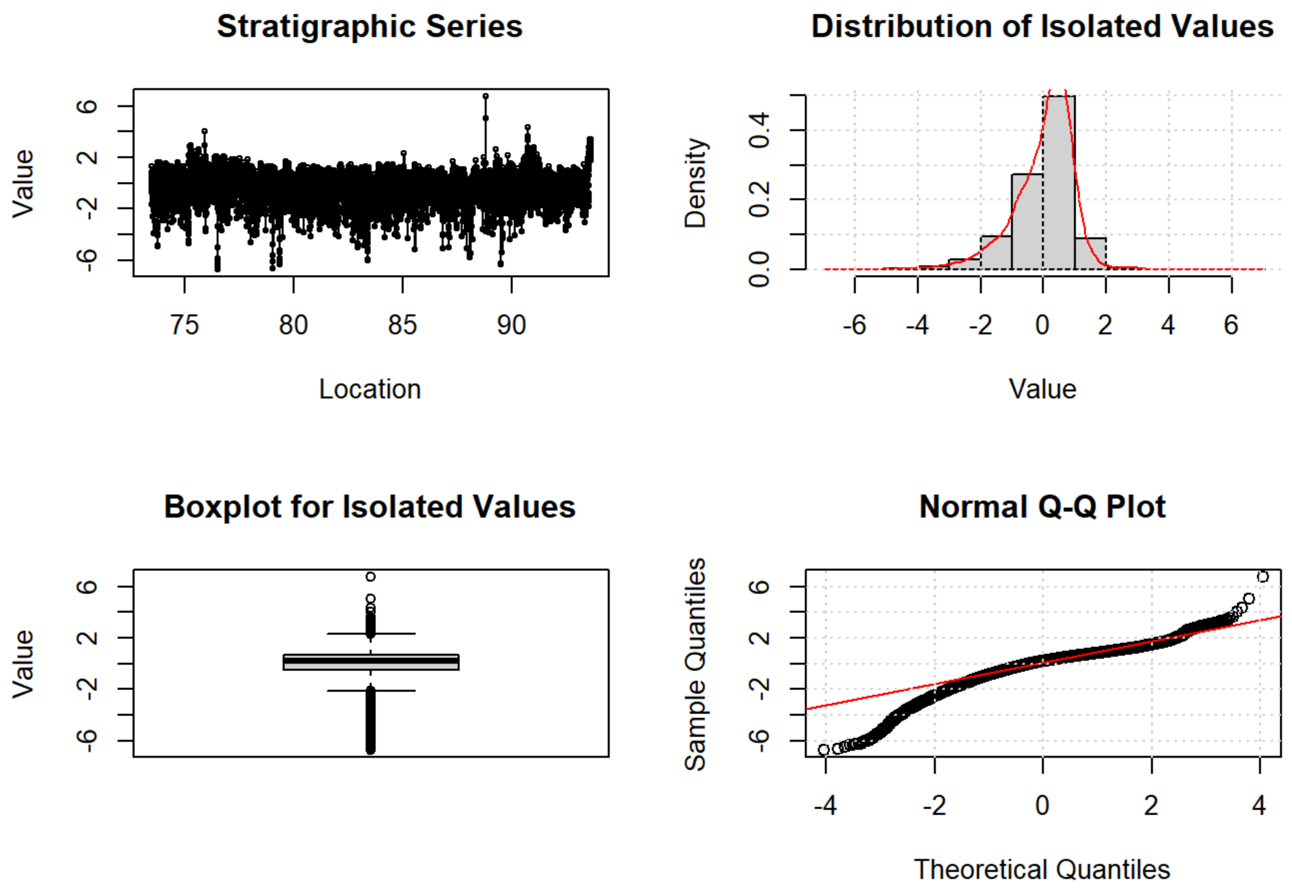


```
alb_Si <- linterp(alb_Si, dt=0.005, genplot=T)
```

```
----- APPLYING PIECEWISE-LINEAR INTERPOLATION TO STRATIGRAPHIC SERIES -----

 * Number of samples= 19310
 * New number of samples= 4032
```

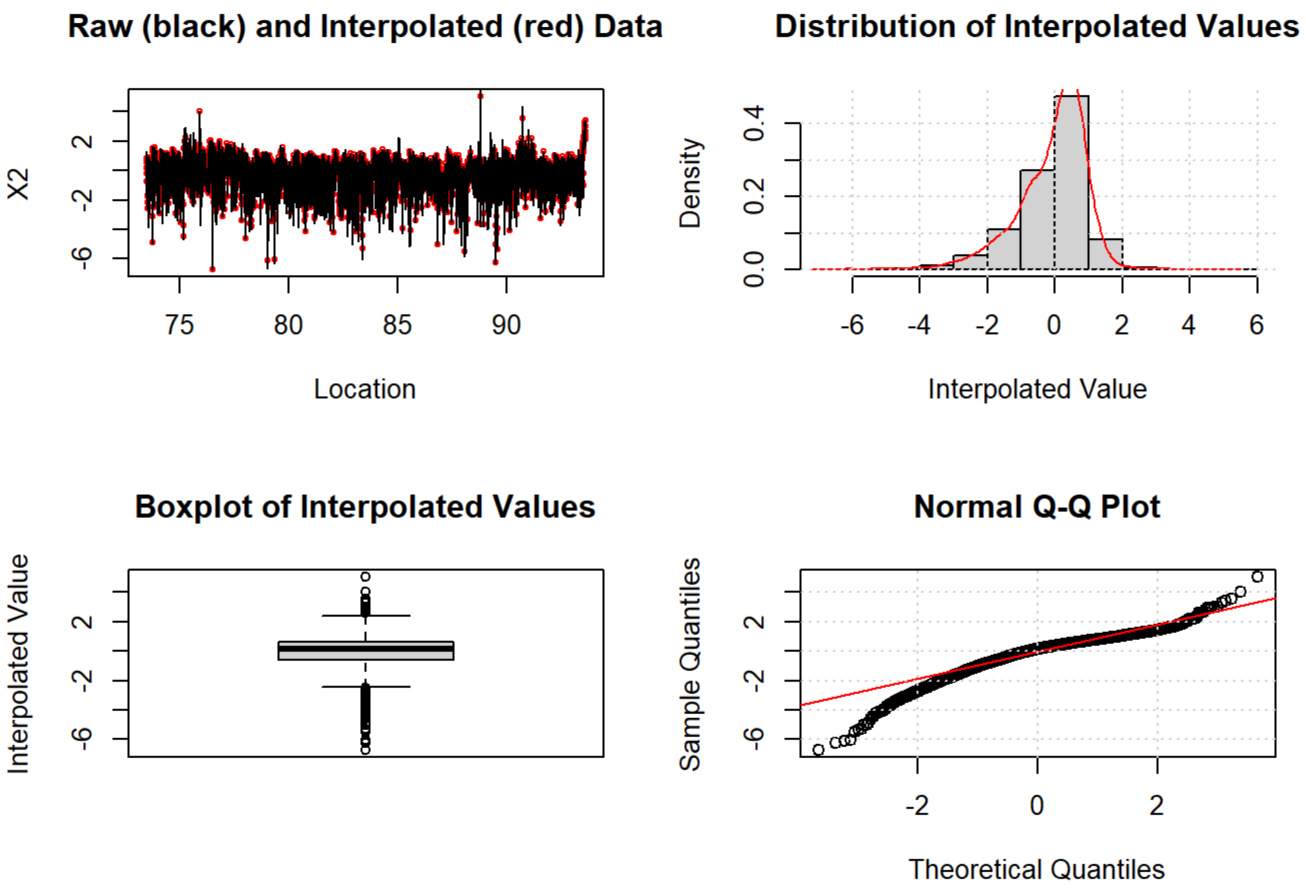


```r
Si_173R <- taner(alb_Si,xmax=2,fhigh=1/0.97,flow=1/0.75,demean=TRUE)
```

```
----- TANER BANDPASS FILTERING STRATIGRAPHIC SERIES-----
 * Number of data points= 4032
 * Sample interval= 0.005
 * Mean value removed= -0.0791156
```

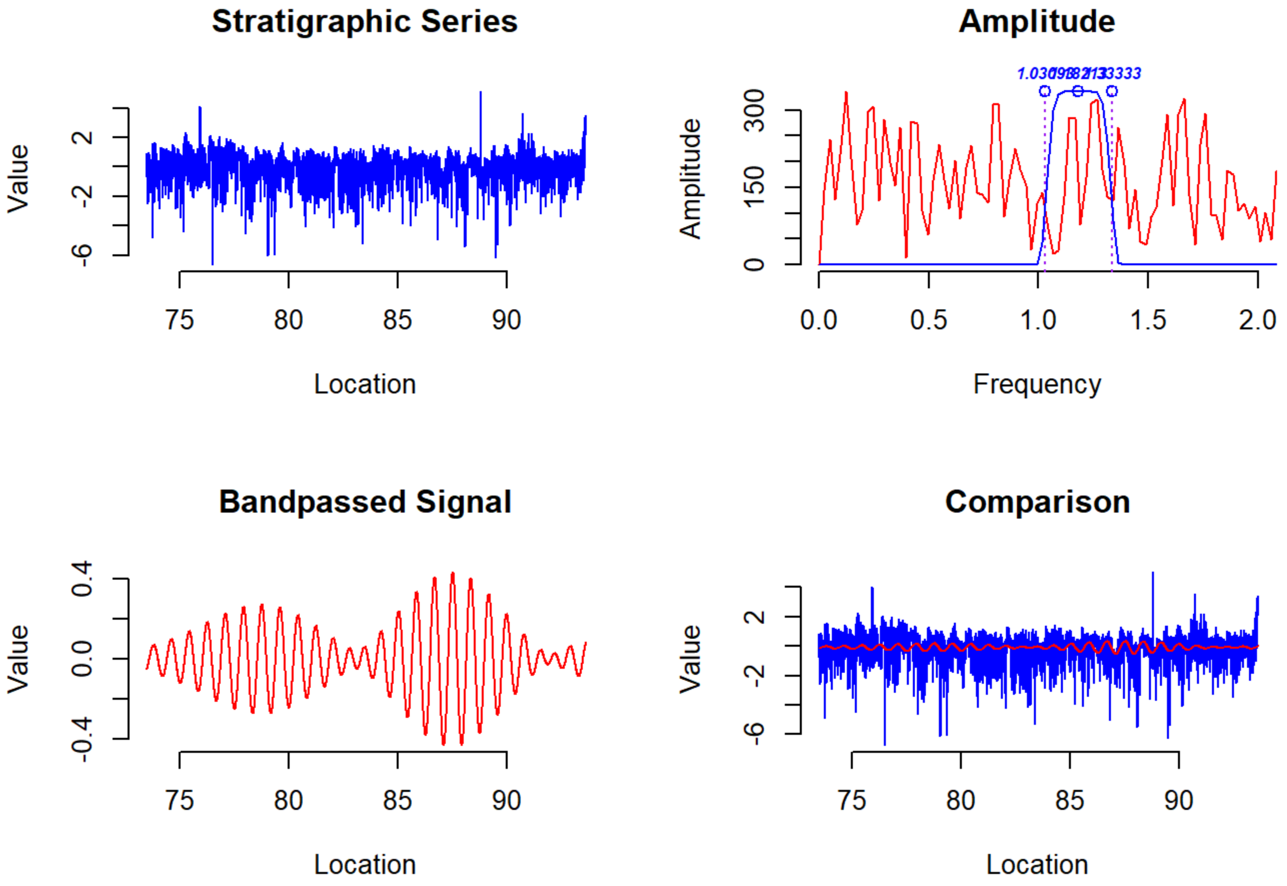


```r
Si_173R_min_tun <-    minimal_tuning(data = Si_173R,
                                     pts = 20,
                                     cycle = 173,
                                     tune_opt = "min",
                                     output = 0,
                                     genplot = TRUE,
                                     keep_editable = FALSE
                                     )
```

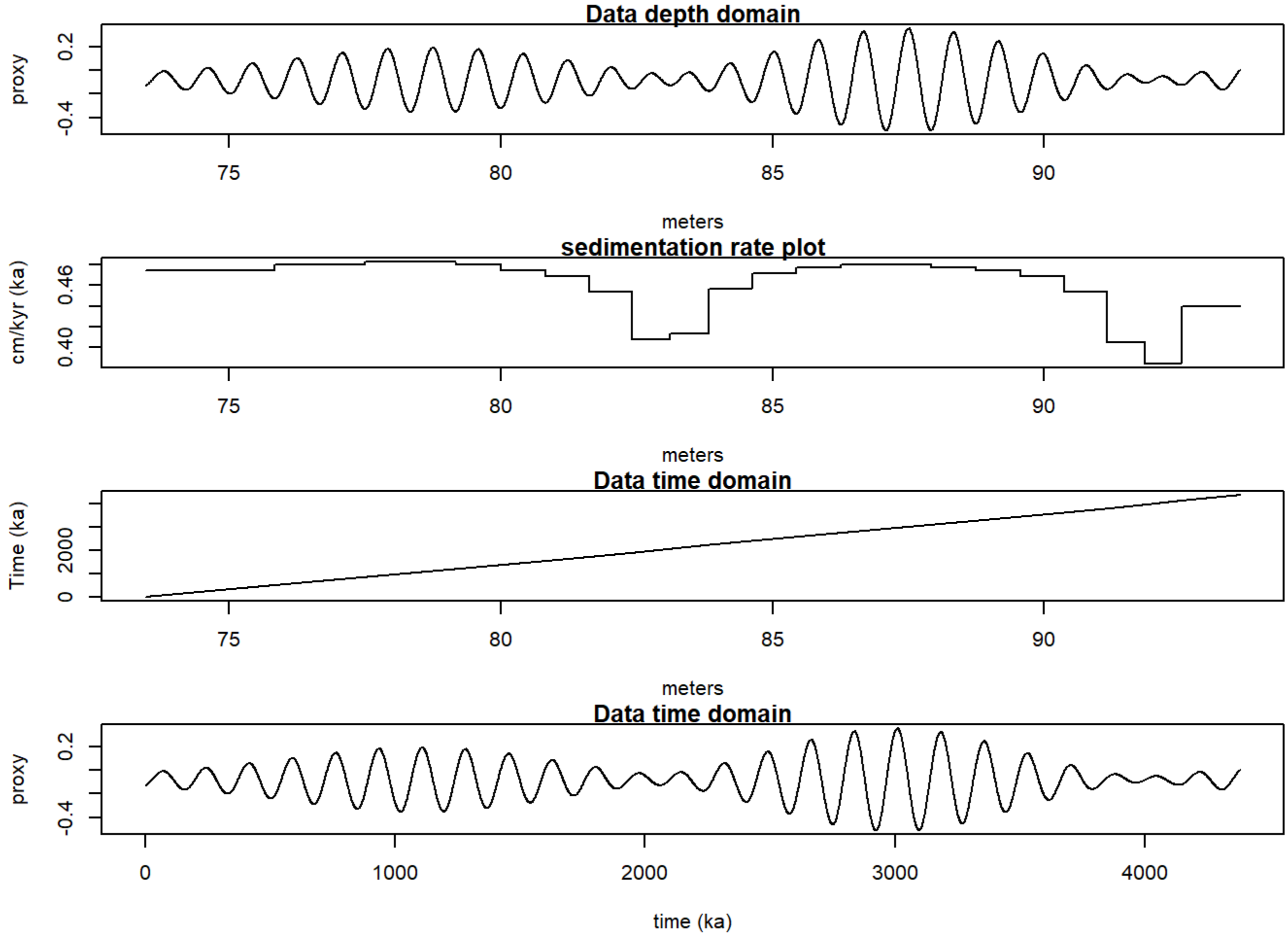


```
colnames(Si_173R_min_tun)<-c("Si Depth","Si","Si SR","Si time")
```

### 11.1.2.3. Aluminium

```
alb_Al <- cbind(Alb$DepthAdj,Alb$Al)
alb_Al <- na.omit(alb_Al)
alb_Al[!is.finite(alb_Al)] <- NA
alb_Al <- na.omit(alb_Al)
alb_Al <- iso(dat=alb_Al, xmin=73.453, xmax=93.609)
```

```
----- ISOLATE STRATIGRAPHIC DATA BY LOCATION -----
 * Number of data points= 30062
 * Number of columns= 2
 * Minimum= 62.111 , Maximum= 93.609
 * Isolating data between 73.453 and 93.609
 * Number of data points following culling= 18796
```

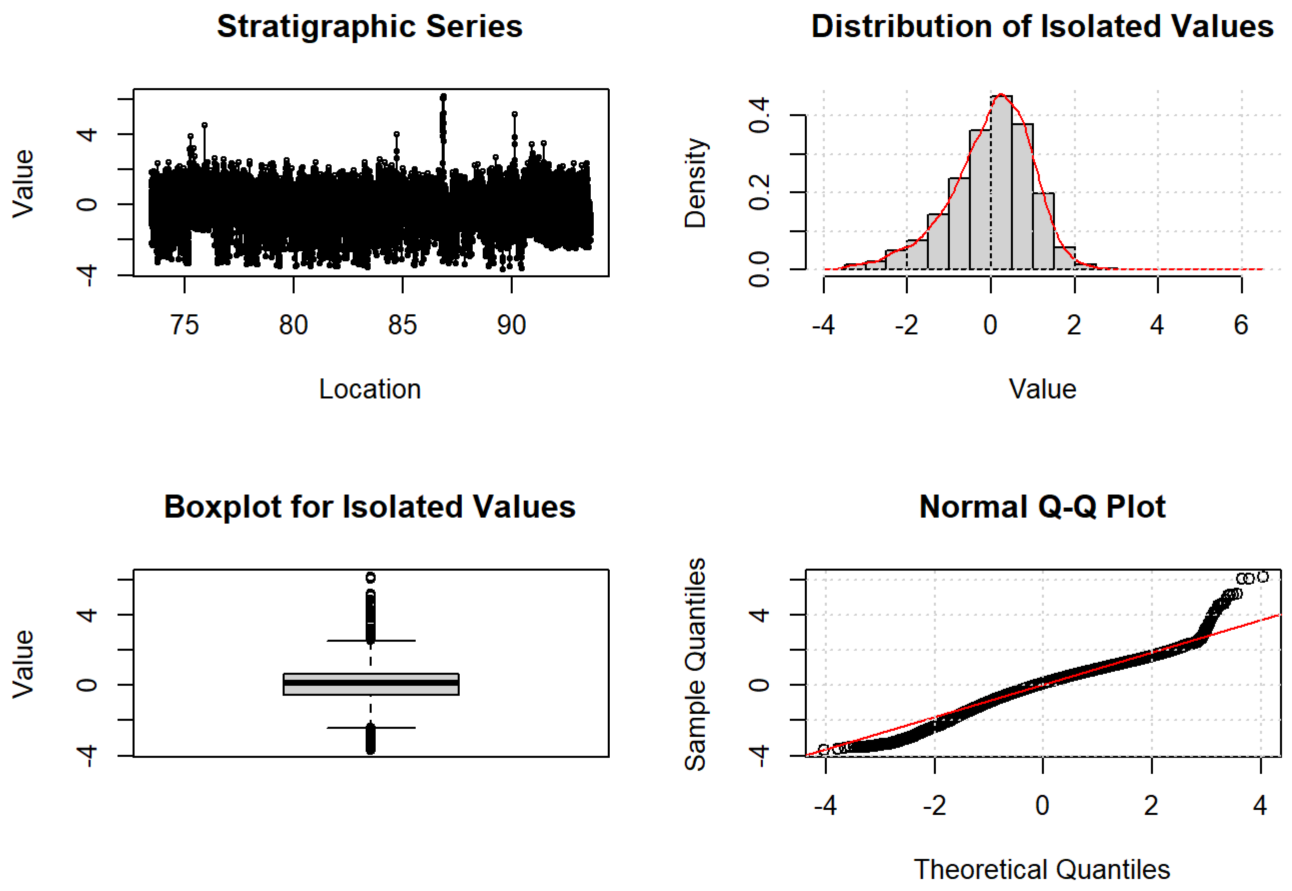


```
alb_Al <- linterp(alb_Al, dt=0.005, genplot=T)
```

```
----- APPLYING PIECEWISE-LINEAR INTERPOLATION TO STRATIGRAPHIC SERIES -----

 * Number of samples= 18796
 * New number of samples= 4032
```

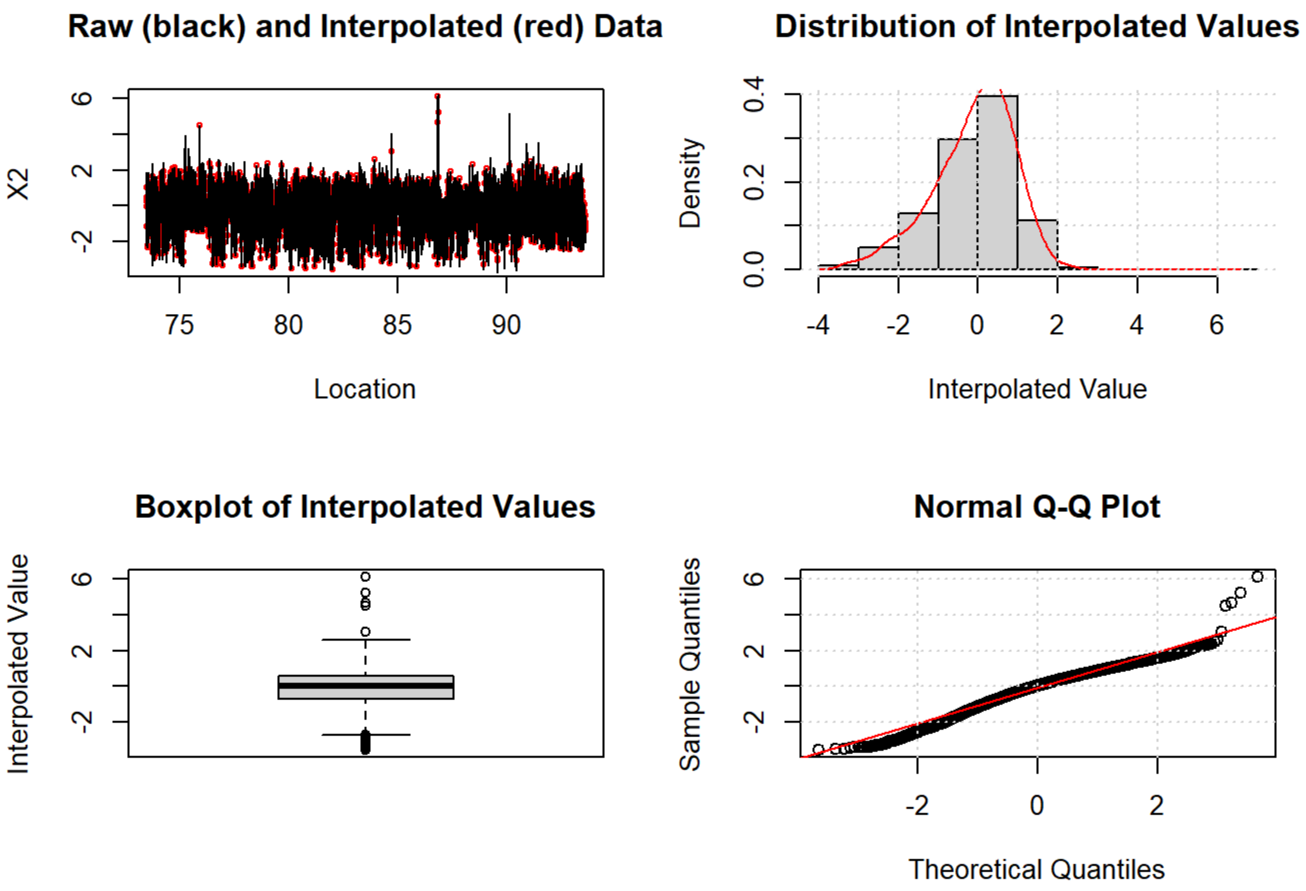


```
Al_173R <- taner(alb_Al,xmax=2,fhigh=1/0.97,flow=1/0.75,demean=TRUE)
```

```
----- TANER BANDPASS FILTERING STRATIGRAPHIC SERIES-----
 * Number of data points= 4032
 * Sample interval= 0.005
 * Mean value removed= -0.1067612
```

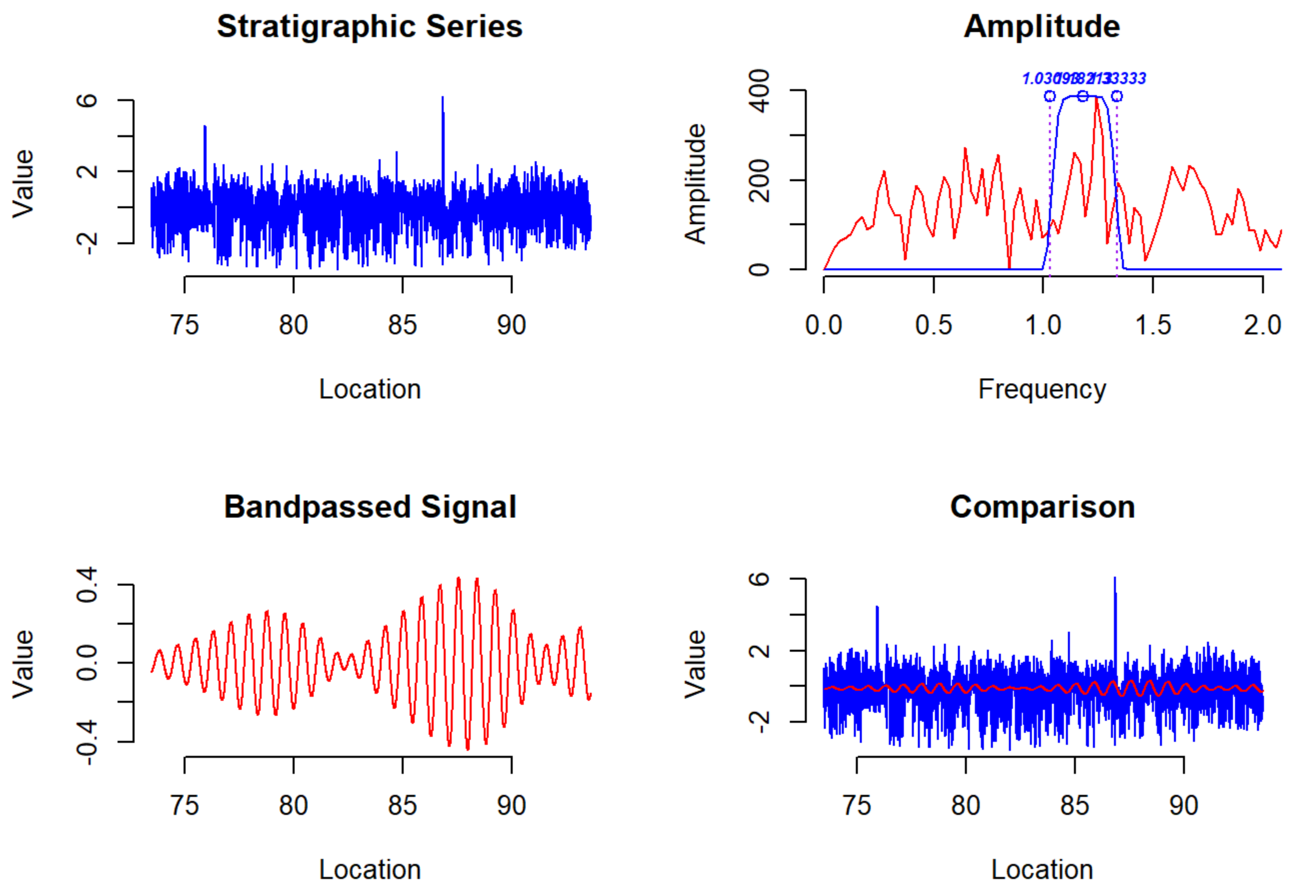


```
Al_173R_min_tun <-    minimal_tuning(data = Al_173R,
                              pts = 20,
                              cycle = 173,
                              tune_opt = "min",
                              output = 0,
                              genplot = TRUE,
                              keep_editable = FALSE
                              )
```

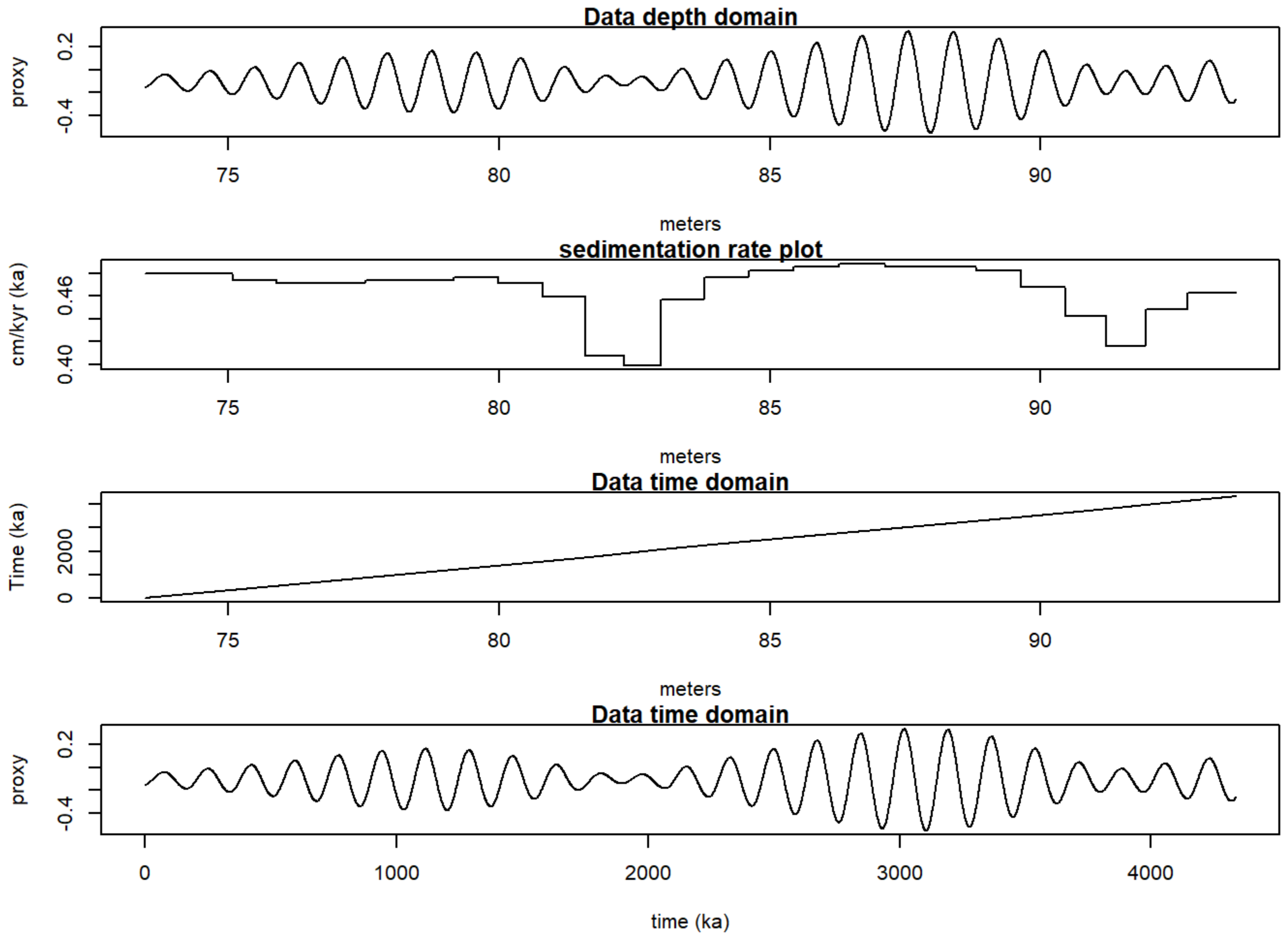


```
colnames(Al_173R_min_tun)<-c("Al Depth","Al","Al SR","Al time")
```

### 11.1.2.4. Potassium

```
alb_K <- cbind(Alb$DepthAdj,Alb$K)
alb_K <- na.omit(alb_K)
alb_K[!is.finite(alb_K)] <- NA
alb_K <- na.omit(alb_K)
alb_K <- iso(dat=alb_K, xmin=73.453, xmax=93.609)
```

```
----- ISOLATE STRATIGRAPHIC DATA BY LOCATION -----
 * Number of data points= 19311
 * Number of columns= 2
 * Minimum= 73.453 , Maximum= 93.61
 * Isolating data between 73.453 and 93.609
 * Number of data points following culling= 19310
```

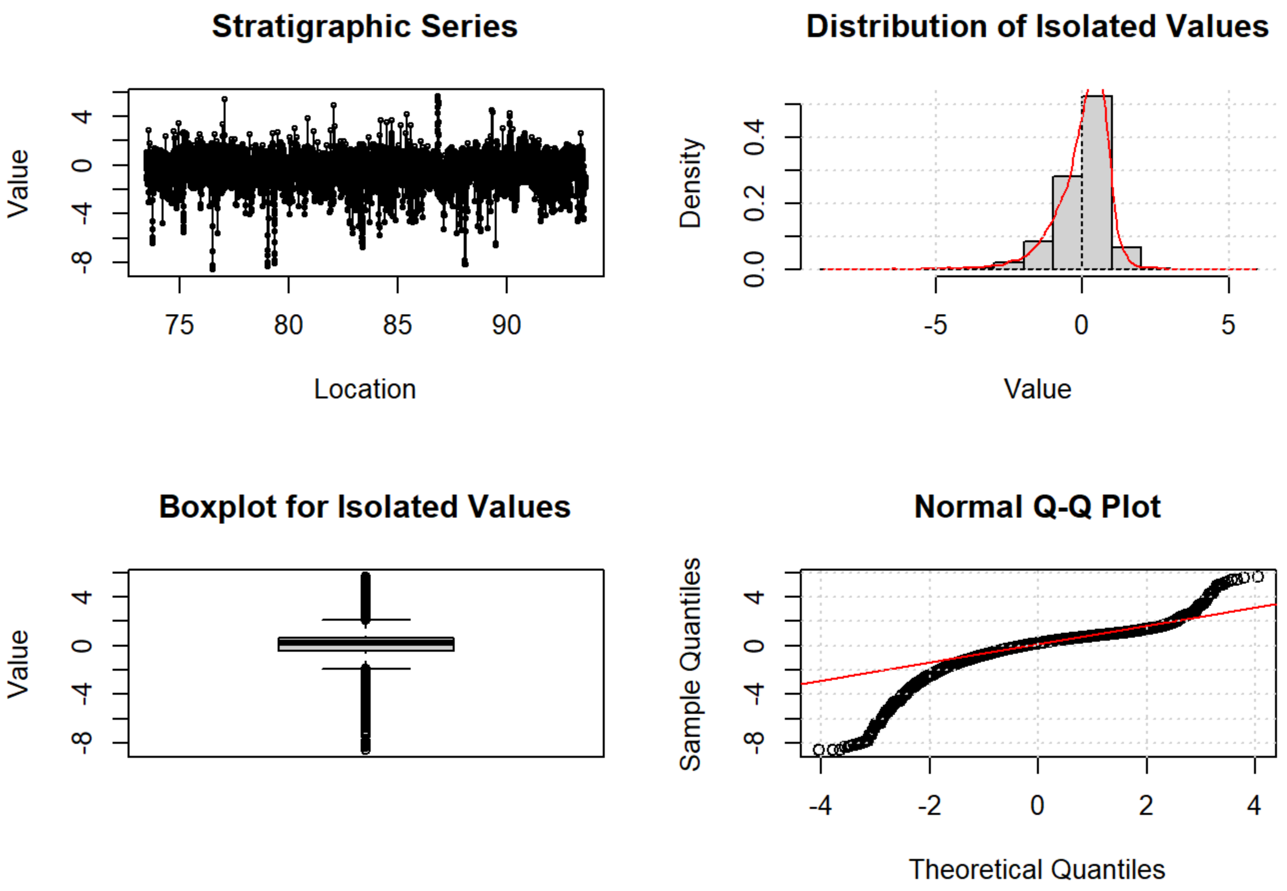


```r
alb_K <- linterp(alb_K, dt=0.005, genplot=T)
```

```
----- APPLYING PIECEWISE-LINEAR INTERPOLATION TO STRATIGRAPHIC SERIES -----

 * Number of samples= 19310
 * New number of samples= 4032
```

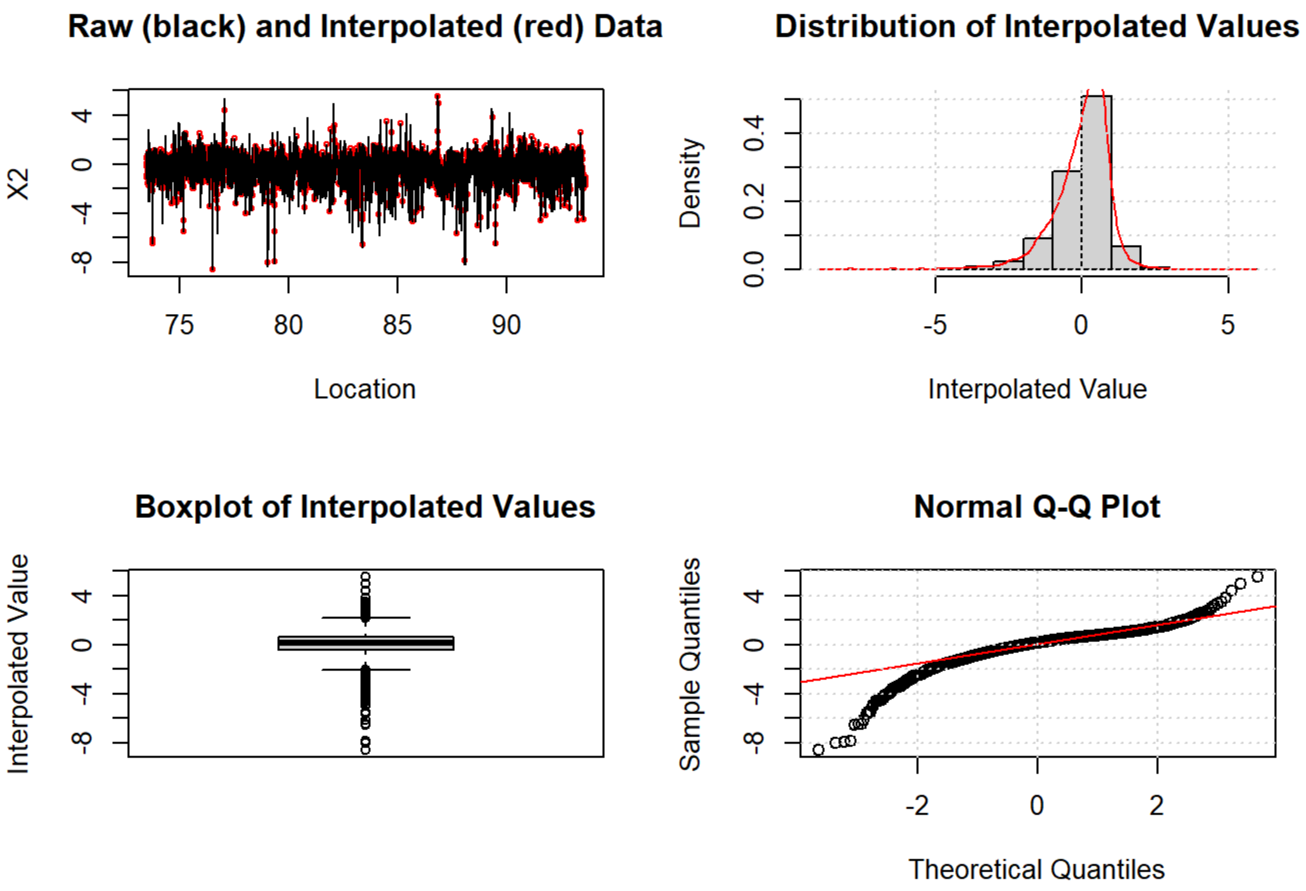


```
K_173R <- taner(alb_K,xmax=2,fhigh=1/0.97,flow=1/0.75,demean=TRUE)
```

```
----- TANER BANDPASS FILTERING STRATIGRAPHIC SERIES-----
 * Number of data points= 4032
 * Sample interval= 0.005
 * Mean value removed= -0.01677853
```

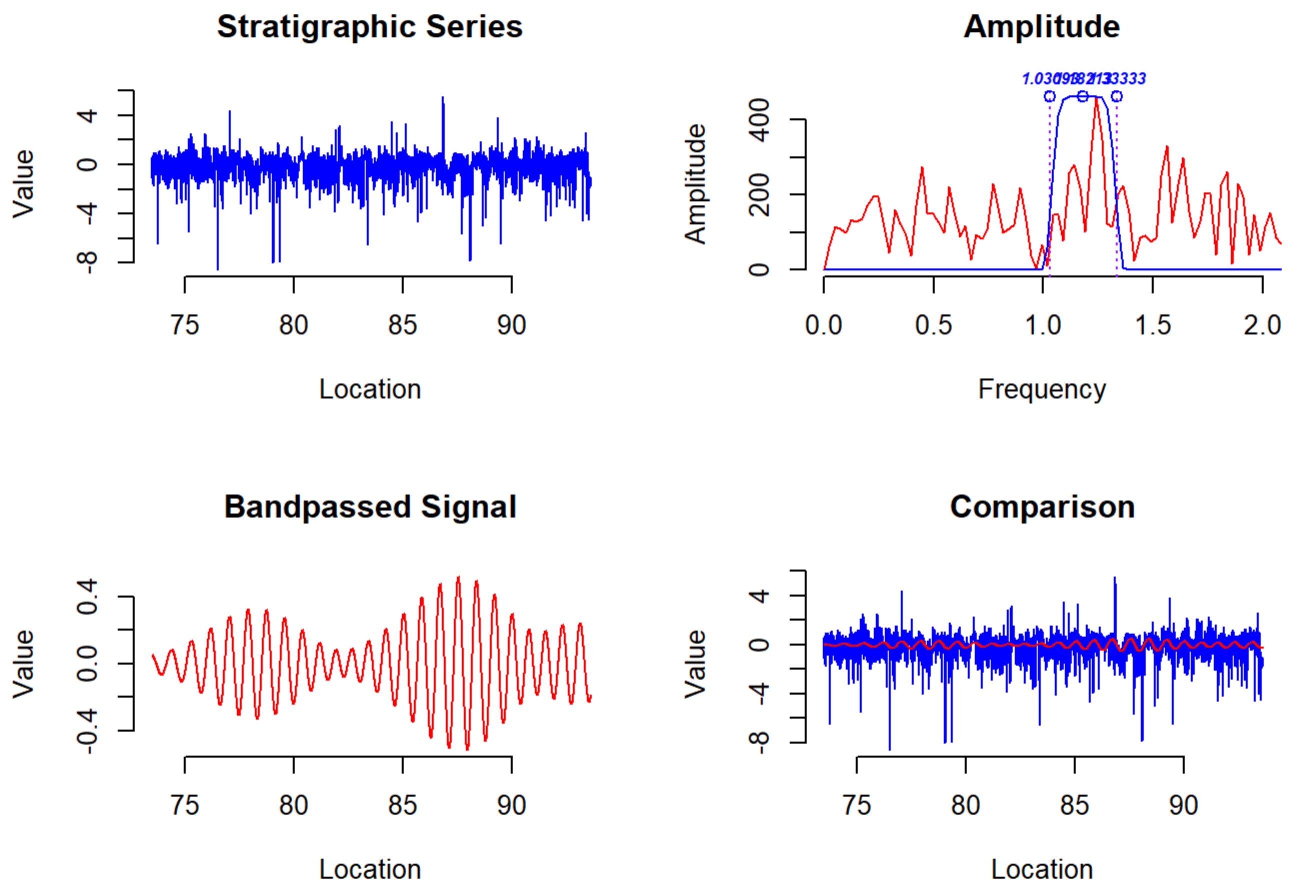


```
K_173R_min_tun <-    minimal_tuning(data = K_173R,
                                    pts = 20,
                                    cycle = 173,
                                    tune_opt = "min",
                                    output = 0,
                                    genplot = TRUE,
                                    keep_editable = FALSE
                                    )
```

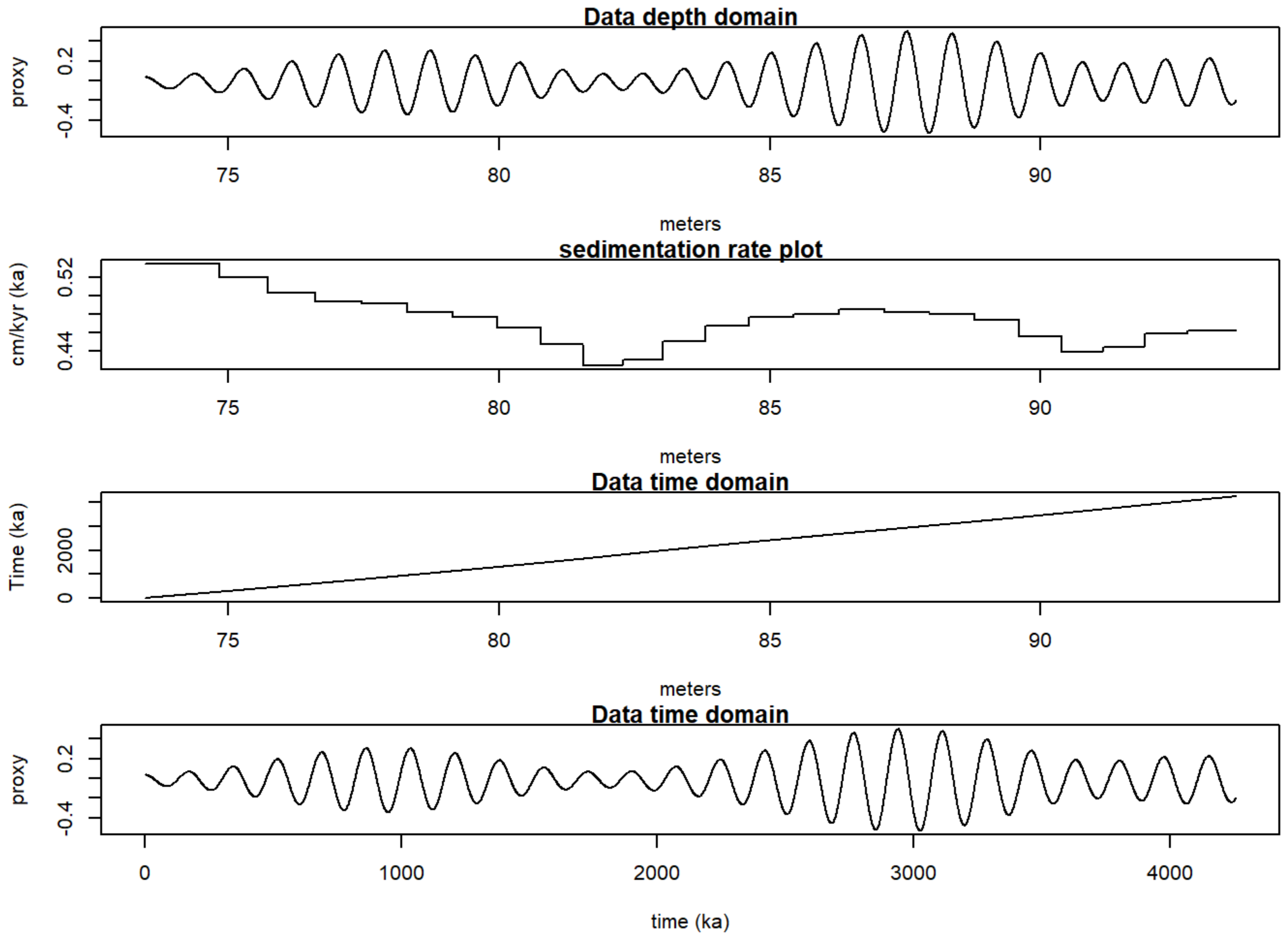


```
colnames(K_173R_min_tun)<-c("K Depth","K","K SR","K time")
```

### 11.1.2.5. Zirconium

```
alb_Zr <- cbind(Alb$DepthAdj,Alb$Zr)
alb_Zr <- na.omit(alb_Zr)
alb_Zr[!is.finite(alb_Zr)] <- NA
alb_Zr <- na.omit(alb_Zr)
alb_Zr <- iso(dat=alb_Zr, xmin=73.453, xmax=93.609)
```

```
----- ISOLATE STRATIGRAPHIC DATA BY LOCATION -----
 * Number of data points= 18653
 * Number of columns= 2
 * Minimum= 73.453 , Maximum= 93.609
 * Isolating data between 73.453 and 93.609
 * Number of data points following culling= 18653
```

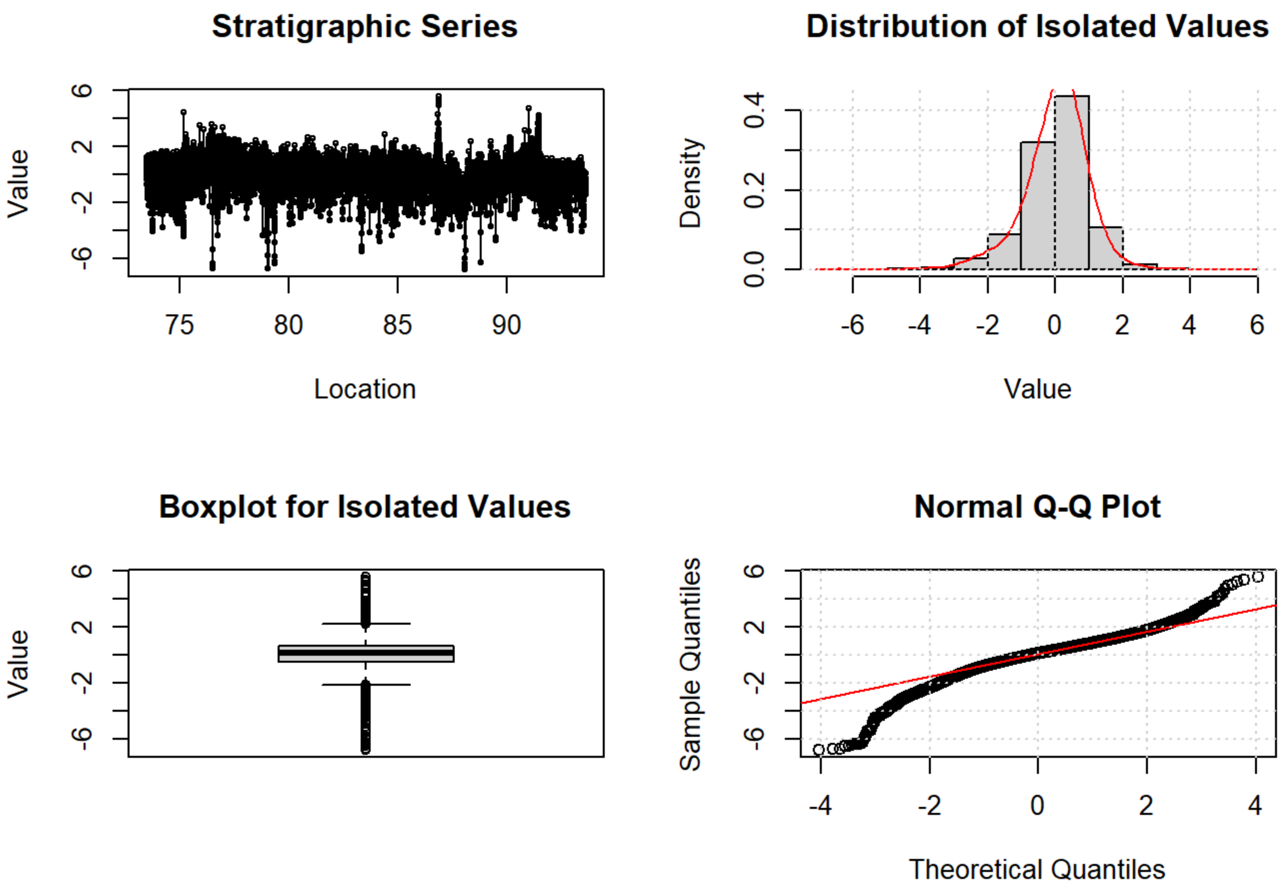


```
alb_Zr <- linterp(alb_Zr, dt=0.005, genplot=T)
```

```
----- APPLYING PIECEWISE-LINEAR INTERPOLATION TO STRATIGRAPHIC SERIES -----

 * Number of samples= 18653
 * New number of samples= 4032
```

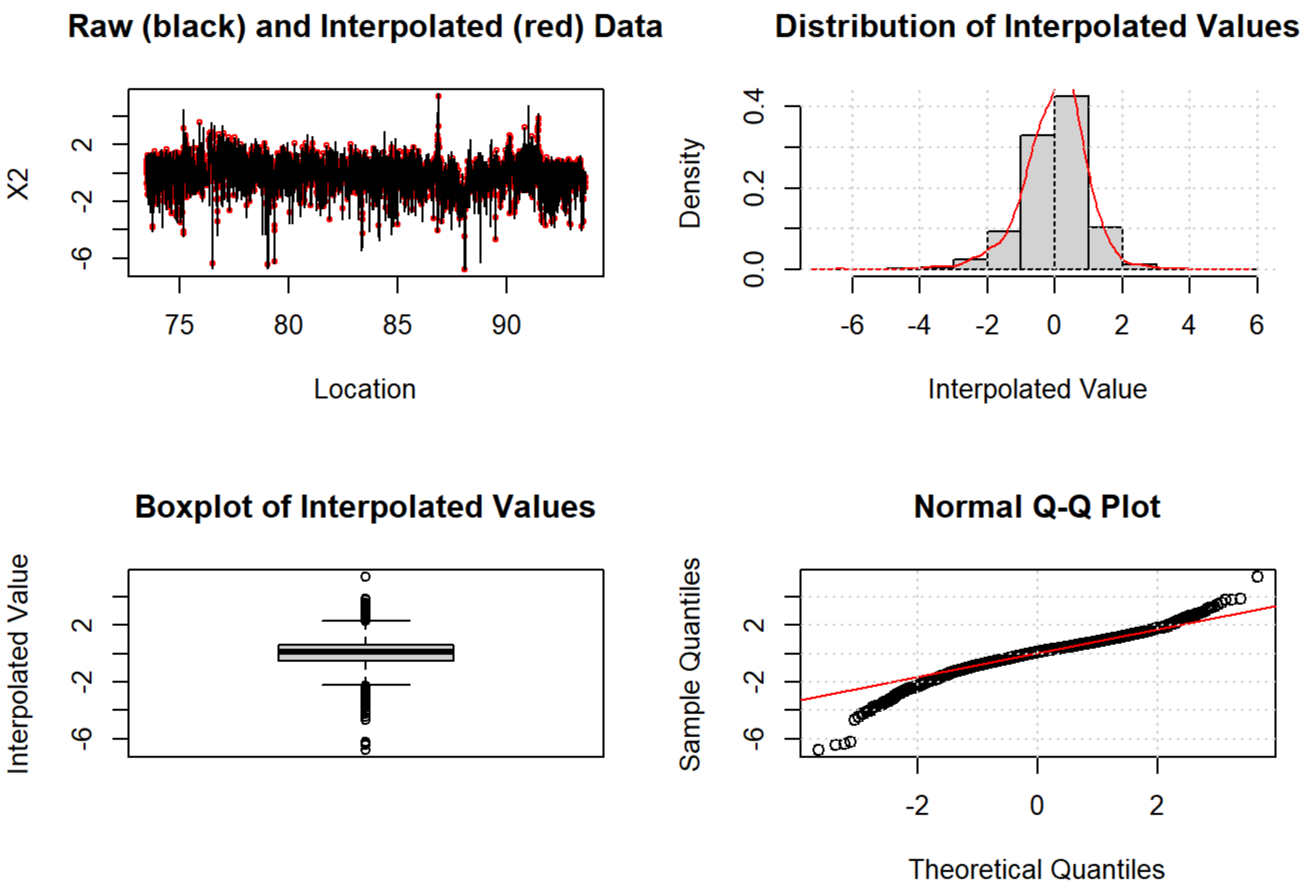


```
Zr_173R <- taner(alb_Zr,xmax=2,fhigh=1/0.97,flow=1/0.75,demean=TRUE)
```

```
----- TANER BANDPASS FILTERING STRATIGRAPHIC SERIES-----
 * Number of data points= 4032
 * Sample interval= 0.005
 * Mean value removed= -0.004319494
```

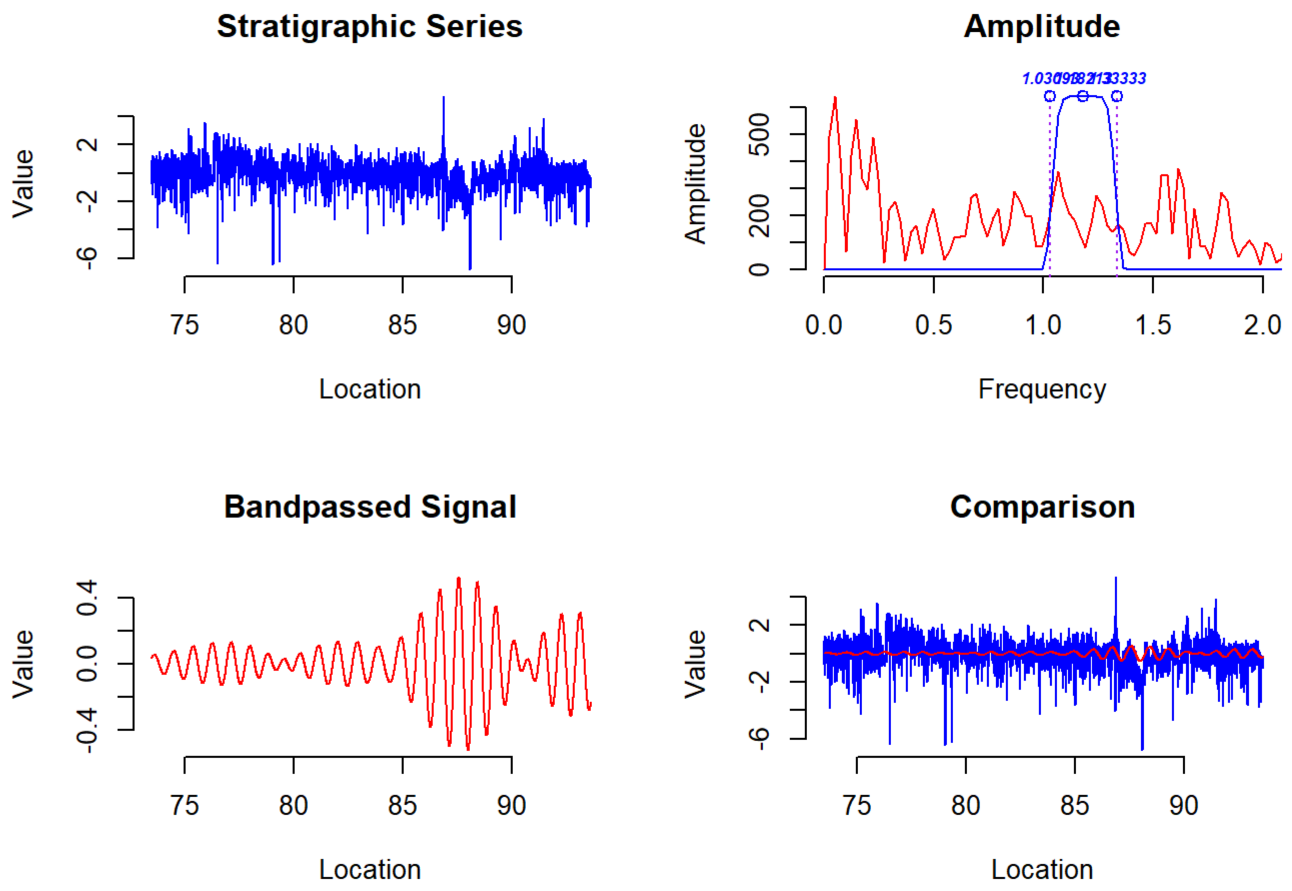


```
Zr_173R_min_tun <-    minimal_tuning(data = Zr_173R,pts = 20,cycle = 173,
tune_opt = "min",output = 0,genplot = TRUE,keep_editable = FALSE)
```

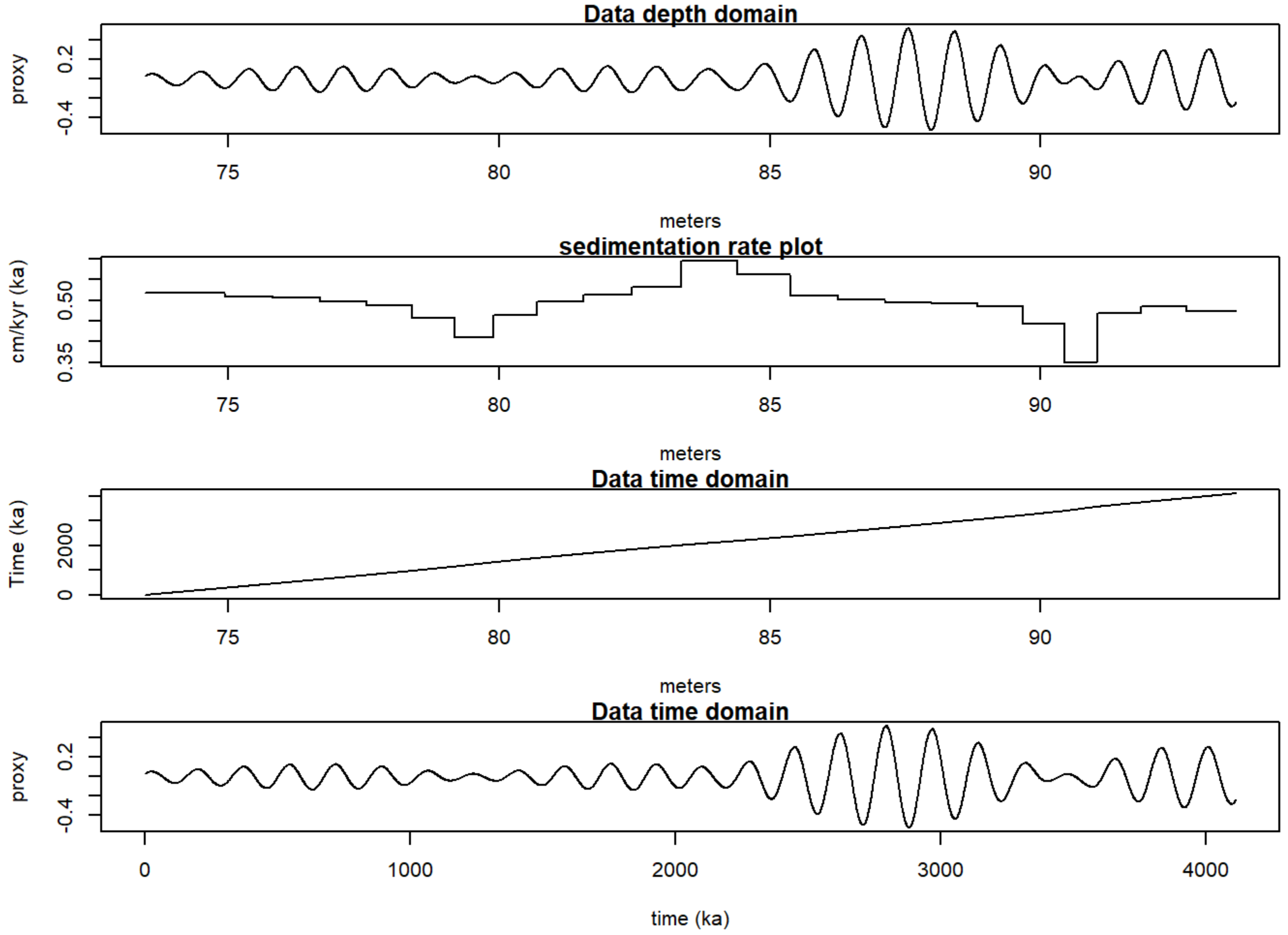


```
colnames(Zr_173R_min_tun)<-c("Zr Depth","Zr","Zr SR","Zr time")
```

### 11.1.3. File containing the filtering of all the detrital element

```
#All detrital
Det_173_min_tun <- cbind(Ti_173R_min_tun,
                         Si_173R_min_tun,
                         Al_173R_min_tun,
                         K_173R_min_tun,
                         Zr_173R_min_tun
                         )

head((Det_173_min_tun))
```

```
  Ti Depth          Ti     Ti SR   Ti time Si Depth         Si     Si SR
1   73.453 -0.01918895 0.5086705 0.0000000   73.453 -0.1302751 0.4739884
2   73.458 -0.01773397 0.5086705 0.9829545   73.458 -0.1289772 0.4739884
3   73.463 -0.01629626 0.5086705 1.9659091   73.463 -0.1275983 0.4739884
4   73.468 -0.01487777 0.5086705 2.9488636   73.468 -0.1261400 0.4739884
5   73.473 -0.01348043 0.5086705 3.9318182   73.473 -0.1246042 0.4739884
6   73.478 -0.01210616 0.5086705 4.9147727   73.478 -0.1229929 0.4739884
   Si time Al Depth         Al     Al SR  Al time K Depth          K      K SR
1 0.000000   73.453 -0.1533187 0.4797688 0.000000  73.453 0.03580243 0.5346821
2 1.054878   73.458 -0.1524303 0.4797688 1.042169  73.458 0.03562798 0.5346821
3 2.109756   73.463 -0.1514751 0.4797688 2.084337  73.463 0.03539204 0.5346821
4 3.164634   73.468 -0.1504541 0.4797688 3.126506  73.468 0.03509460 0.5346821
```

```
5 4.219512   73.473 -0.1493681 0.4797688 4.168675  73.473 0.03473567 0.5346821
6 5.274390   73.478 -0.1482183 0.4797688 5.210843  73.478 0.03431536 0.5346821
     K time  Zr Depth         Zr     Zr SR    Zr time
1 0.0000000   73.453 0.02894888 0.517341 0.0000000
2 0.9351351   73.458 0.03032727 0.517341 0.9664804
3 1.8702703   73.463 0.03166989 0.517341 1.9329609
4 2.8054054   73.468 0.03297492 0.517341 2.8994413
5 3.7405405   73.473 0.03424055 0.517341 3.8659218
6 4.6756757   73.478 0.03546501 0.517341 4.8324022
```

## 11.1.4. Sedimentation rate

```
plot(Det_173_min_tun[,1],
     Det_173_min_tun[,3],
     type="l",
     xlab = "Depth adjusted (m)",
     ylab = "Sed rate (cm/kyr)",
     ylim=c(0.38,0.55),
     lwd = 2
     )

lines(Det_173_min_tun[,1],
      Det_173_min_tun[,7],
      col="red",
      lwd=2
      )

lines(Det_173_min_tun[,1],
      Det_173_min_tun[,11],
      col="green",
      lwd=2
      )

lines(Det_173_min_tun[,1],
      Det_173_min_tun[,15],
      col="blue",
      lwd=2
      )

lines(Det_173_min_tun[,1],
      Det_173_min_tun[,19],
      col="grey",
      lwd=2
      )

lines(Det_173_min_tun[,1],
      Det_173_min_tun[,19]-Det_173_min_tun[,19]+0.493,
      col="red",
      lwd=2,
      lty=3
      )
```

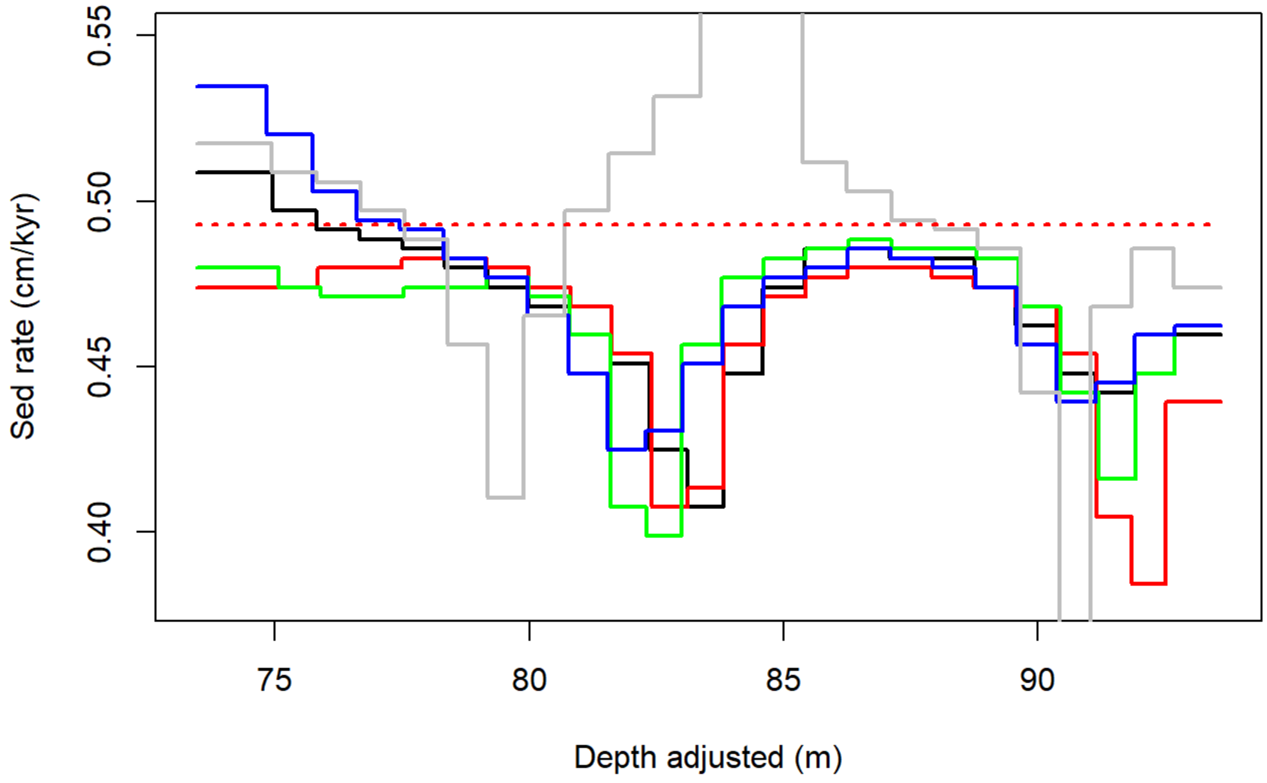


```
#Zr sedimentation rate is significantly different compared to the other detrital
#element and will not be used.

  plot(Det_173_min_tun[,1],
       Det_173_min_tun[,3],
       type="l",
       xlab = "Depth adjusted (m)",
       ylab = "Sed rate (cm/kyr)",
       ylim=c(0.38,0.55),
       lwd = 2
       )

  lines(Det_173_min_tun[,1],
        Det_173_min_tun[,7],
        col="red",
        lwd=2
        )

  lines(Det_173_min_tun[,1],
        Det_173_min_tun[,11],
        col="green",
        lwd=2
        )

  lines(Det_173_min_tun[,1],
        Det_173_min_tun[,15],
        col="blue",
```

```
       lwd=2
       )

lines(Det_173_min_tun[,1],
       Det_173_min_tun[,19]-Det_173_min_tun[,19]+0.493,
       col="red",
       lwd=2,
       lty=3
       )

legend("bottomleft",
        legend = c("Ti (0.75-0.97 m filter)",
                   "Si (0.75-0.97 m filter)",
                   "Al (0.75-0.97 m filter)",
                   "K (0.75-0.97 m filter)",
                   "ASM = 0.493 cm/kyr (theoretical)"),
        col = c("black", "red", "green", "blue", "red"),
        lty = c(1, 1, 1, 1,3),
        lwd = c(2, 2, 2, 2,2),
        pch = c(NA, NA, NA, NA,NA),
        pt.cex = 2,
        bty = "n",          # No box around legend
        cex = 0.7)
```

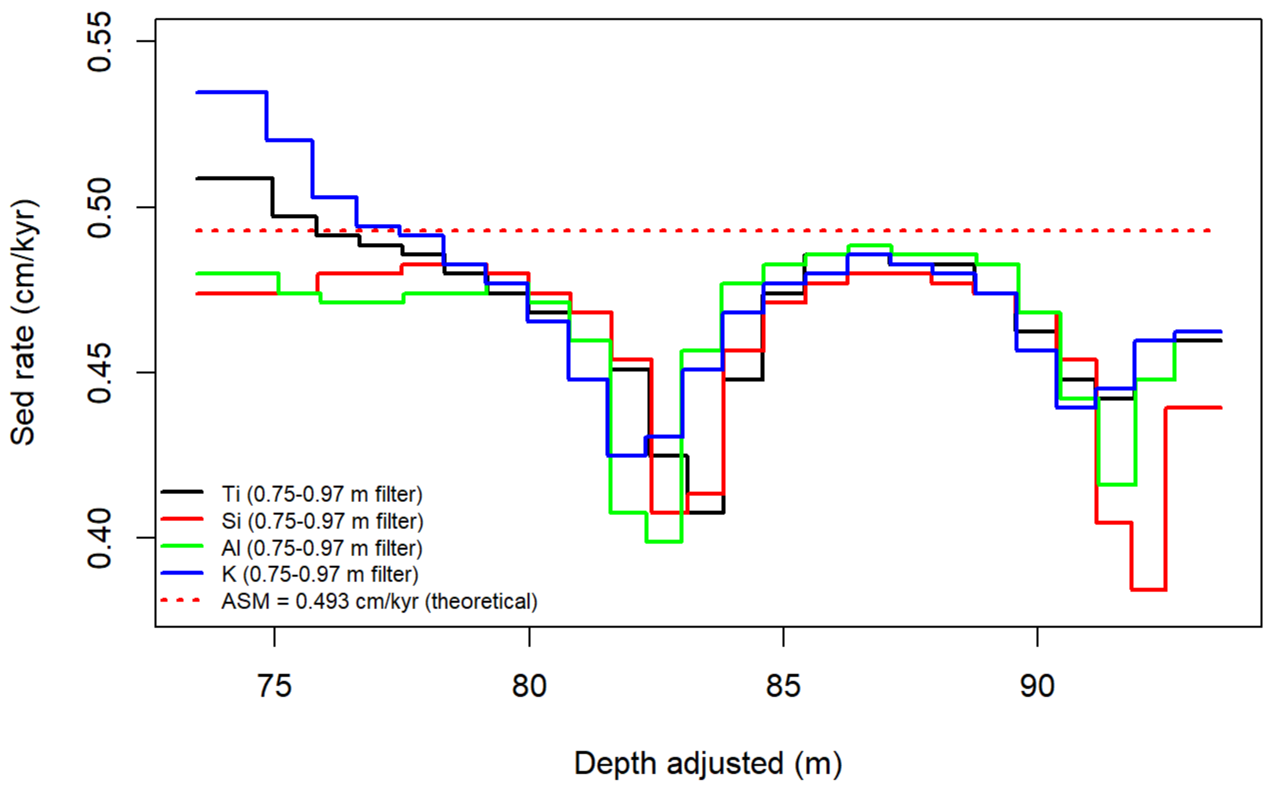


## 11.1.5. Mean and standard deviation calculation

```
#Smoothing the sed rate and turning it into frequency to calculate the mean and the
#standard deviation of the age model
  Ti_173_freq<-noLow(cbind(Det_173_min_tun[,c(1)],1/(Det_173_min_tun[,c(3)]*173)),
                      0.05,
                      output = 2
                      )
```

```
----- REMOVING LOWESS SMOOTHER FROM STRATIGRAPHIC SERIES -----
Call:
loess(formula = (dat[, 2]) ~ dat[, 1], span = smooth, degree = 1)

Number of Observations: 4032
Equivalent Number of Parameters: 31.09
Residual Standard Error: 9.956e-05
Trace of smoother matrix: 36.89  (exact)

Control settings:
  span     :  0.05
  degree   :  1
  family   :  gaussian
  surface  :  interpolate      cell = 0.2
  normalize:  TRUE
 parametric:  FALSE
drop.square:  FALSE
```

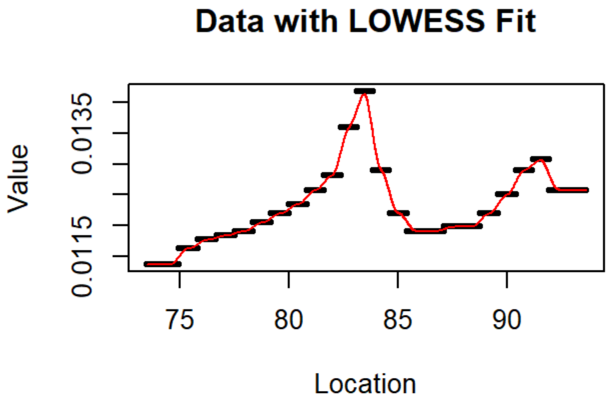


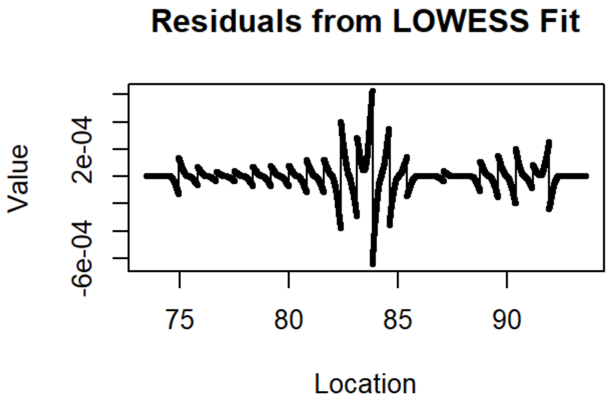


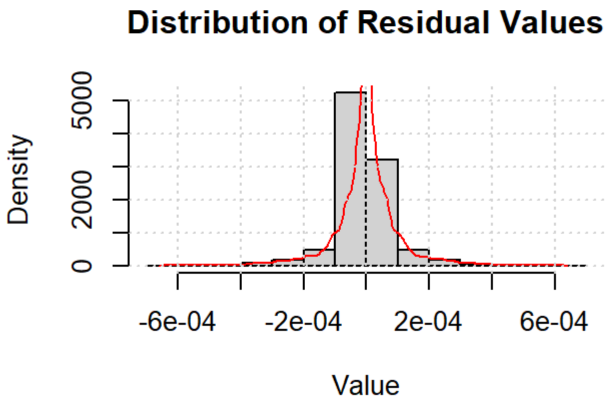


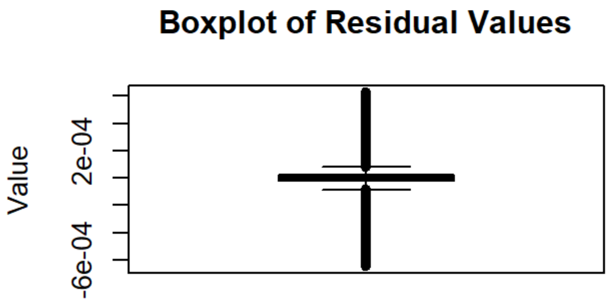

```r
Si_173_freq<-noLow(cbind(Det_173_min_tun[,c(1)],1/(Det_173_min_tun[,c(7)]*173)),
                   0.05,
                   output = 2
                   )
```

```
----- REMOVING LOWESS SMOOTHER FROM STRATIGRAPHIC SERIES -----
Call:
loess(formula = (dat[, 2]) ~ dat[, 1], span = smooth, degree = 1)

Number of Observations: 4032
Equivalent Number of Parameters: 31.09
Residual Standard Error: 0.0001639
Trace of smoother matrix: 36.89  (exact)

Control settings:
  span     :  0.05
  degree   :  1
  family   :  gaussian
  surface  :  interpolate      cell = 0.2
  normalize:  TRUE
 parametric:  FALSE
drop.square:  FALSE
```

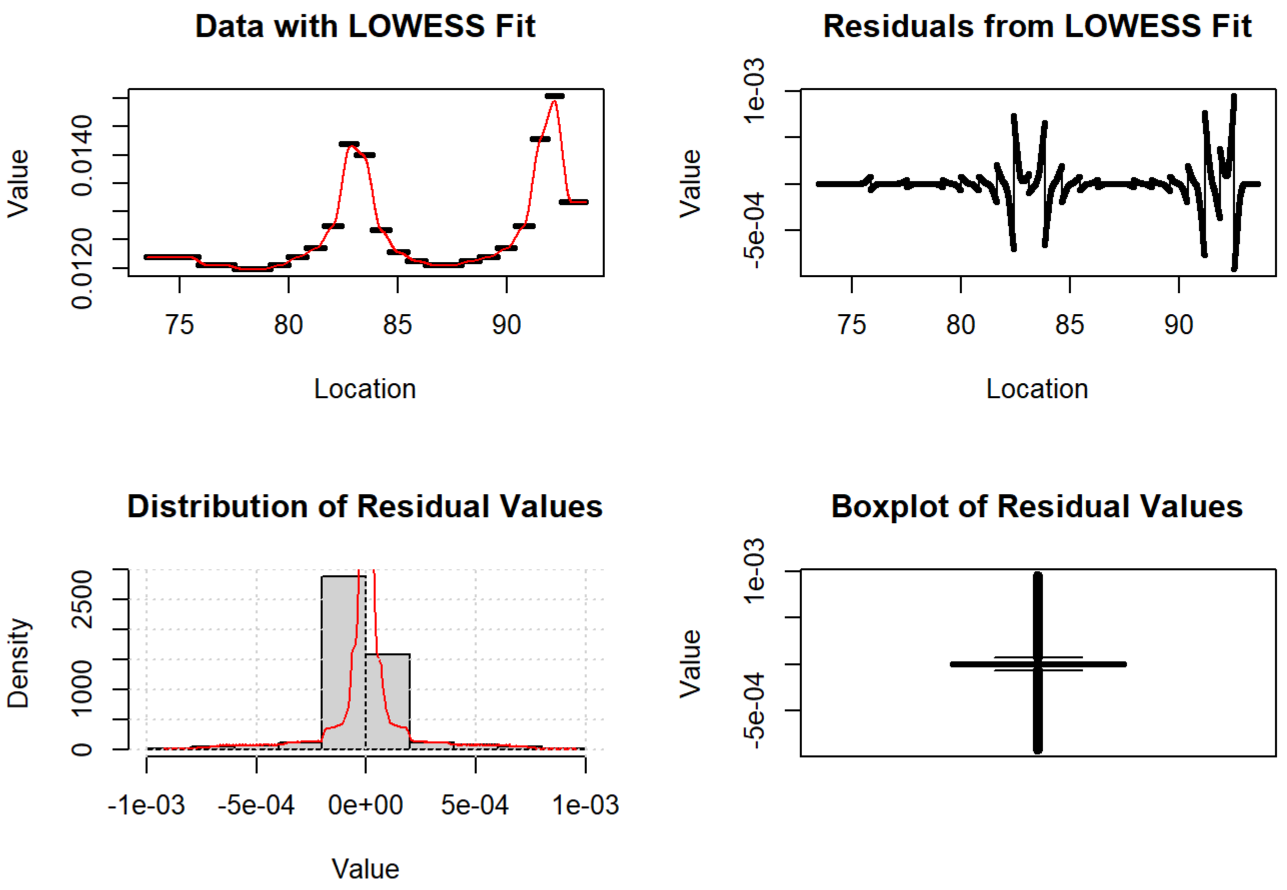


```r
Al_173_freq<-noLow(cbind(Det_173_min_tun[,c(1)],1/(Det_173_min_tun[,c(11)]*173)),
                   0.05,
```

```
                  output = 2
                  )
```

```
----- REMOVING LOWESS SMOOTHER FROM STRATIGRAPHIC SERIES -----
Call:
loess(formula = (dat[, 2]) ~ dat[, 1], span = smooth, degree = 1)

Number of Observations: 4032
Equivalent Number of Parameters: 31.09
Residual Standard Error: 0.0001489
Trace of smoother matrix: 36.89  (exact)

Control settings:
  span     :  0.05
  degree   :  1
  family   :  gaussian
  surface  :  interpolate      cell = 0.2
  normalize:  TRUE
 parametric:  FALSE
drop.square:  FALSE
```

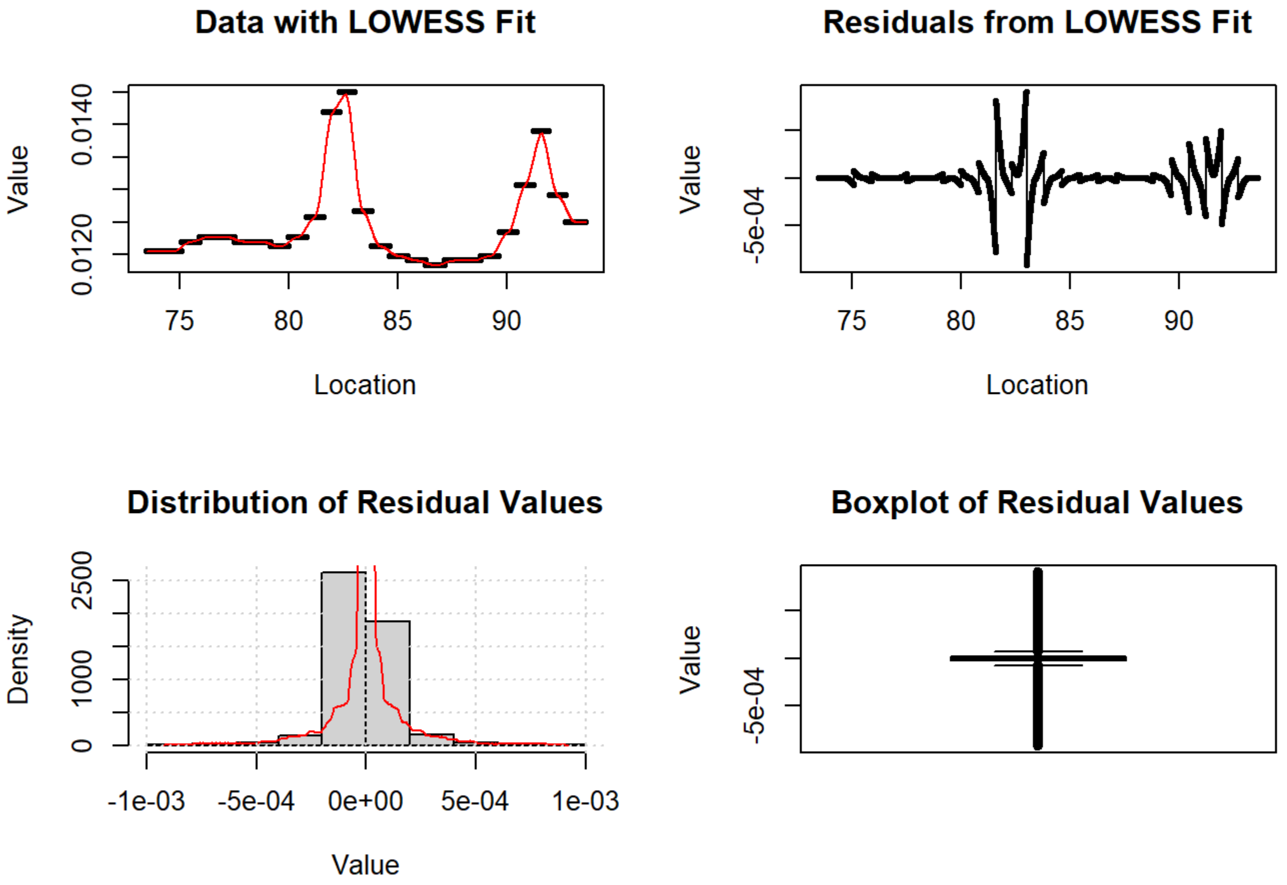


```
K_173_freq<-noLow(cbind(Det_173_min_tun[,c(1)],1/(Det_173_min_tun[,c(15)]*173)),
                 0.05,
                 output = 2
                 )
```

```
----- REMOVING LOWESS SMOOTHER FROM STRATIGRAPHIC SERIES -----
Call:
loess(formula = (dat[, 2]) ~ dat[, 1], span = smooth, degree = 1)

Number of Observations: 4032
Equivalent Number of Parameters: 31.09
Residual Standard Error: 7.944e-05
Trace of smoother matrix: 36.89  (exact)

Control settings:
  span     :  0.05
  degree   :  1
  family   :  gaussian
  surface  :  interpolate      cell = 0.2
  normalize:  TRUE
 parametric:  FALSE
drop.square:  FALSE
```

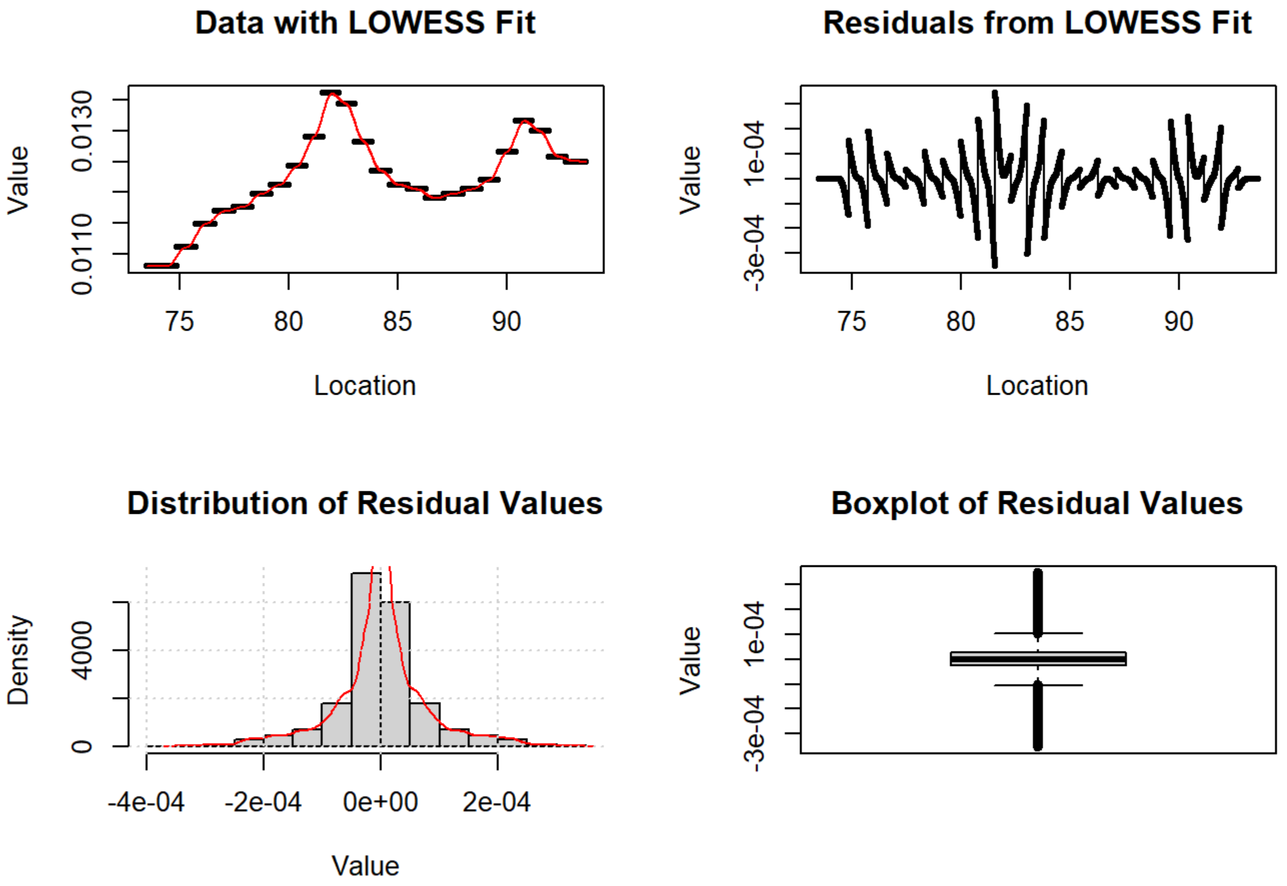


```
Det_173_freq <- cbind(Det_173_min_tun[,1],
                      Ti_173_freq[,2],
                      Si_173_freq[,2],
                      Al_173_freq[,2],
                      K_173_freq[,2]
                      )

head(Det_173_freq)
```

```
      [,1]       [,2]       [,3]       [,4]       [,5]
[1,] 73.453 0.01136364 0.01219512 0.01204819 0.01081081
[2,] 73.458 0.01136364 0.01219512 0.01204819 0.01081081
[3,] 73.463 0.01136364 0.01219512 0.01204819 0.01081081
[4,] 73.468 0.01136364 0.01219512 0.01204819 0.01081081
[5,] 73.473 0.01136364 0.01219512 0.01204819 0.01081081
[6,] 73.478 0.01136364 0.01219512 0.01204819 0.01081081
```

```
Det_173_freq<-as.data.frame(Det_173_freq)
Det_173_freq$mean<-rowMeans(Det_173_freq[,c(2:5)], na.rm = FALSE)

library(matrixStats)
Det_173_freq$sds<-rowSds(as.matrix(Det_173_freq[,c(2:5)]), na.rm = FALSE)
colnames(Det_173_freq)<- cbind("Depth",
                               "Ti freq",
                               "Si freq",
                               "Al freq",
                               "K freq",
                               "mean",
                               "sds"
                               )
```

```
plot(Det_173_freq[,1],
     1/(Det_173_freq[,2]),
     type="l",
     col="black",
     lwd= 2,
     xlab = "Depth adjusted (m)",
     ylab = "Period (cm)",
     ylim = c(65,95)
     )

lines(Det_173_freq[,1],
      1/(Det_173_freq[,3]),
      col="red",
      lwd=2
      )

lines(Det_173_freq[,1],
      1/(Det_173_freq[,4]),
      col="green",
      lwd=2
      )

lines(Det_173_freq[,1],
      1/(Det_173_freq[,5]),
      col="blue",
      lwd=2
      )

#Graphical visualisation of the uncertaineties
depth<-Det_173_freq[,1]
curve_mean <- Det_173_freq[,6]
```

```
curve_sd <- Det_173_freq[,7]

# Add polygon for mean ± SD
polygon(c(depth, rev(depth)),
        1/c(curve_mean + curve_sd, rev(curve_mean - curve_sd)),
        col = rgb(0.7, 0.7, 0.7, 0.4), border = NA)

# Optionally overlay mean line
lines(depth, 1/curve_mean, col = "yellow", lwd = 2)
legend("bottomleft",
       legend = c("Ti", "Si", "K", "Al", "Mean", "Mean ± SD"),
       col = c("black", "red", "blue", "green","yellow", rgb(0.7, 0.7, 0.7, 0.8)),
       lty = c(1, 1, 1, 1, 1,NA),
       lwd = c(1, 1, 1, 1, 1,NA),
       pch = c(NA, NA, NA, NA,NA, 15),
       pt.cex = 2,
       bty = "n",          # No box around legend
       cex = 1)          # Larger legend text
```

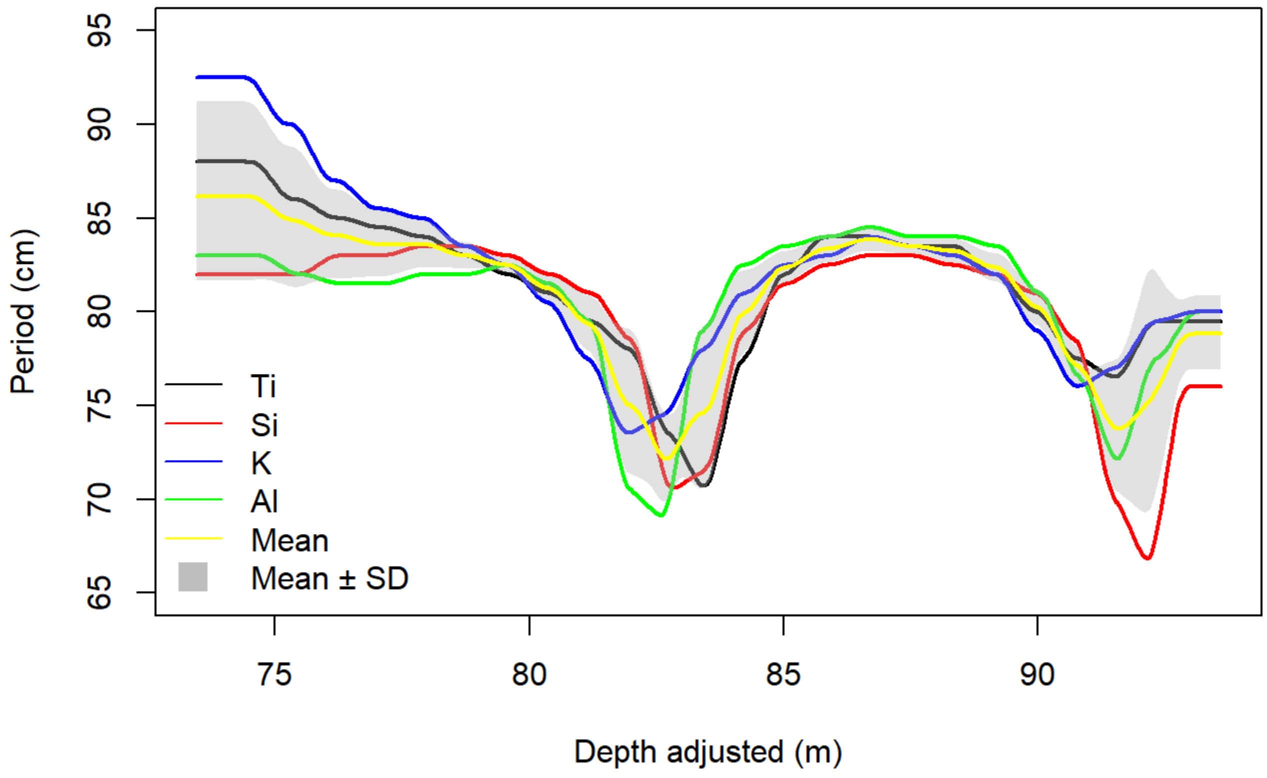


## 11.1.6. Building of the Age model

```
#Calculating the minimal, mean and maximal sed rates
mean_sed<-freq2sedrate(cbind(Det_173_freq[,1],
                             (Det_173_freq[,6])),
                       period=17300
                       )
```

```
----- CONVERTING RECORD OF SPATIAL FREQUENCY TO SEDIMENTATION RATE CURVE-----

 * Sorting into increasing depth/height order.
     Will remove empty entries.
 * Number of control points = 4032
```

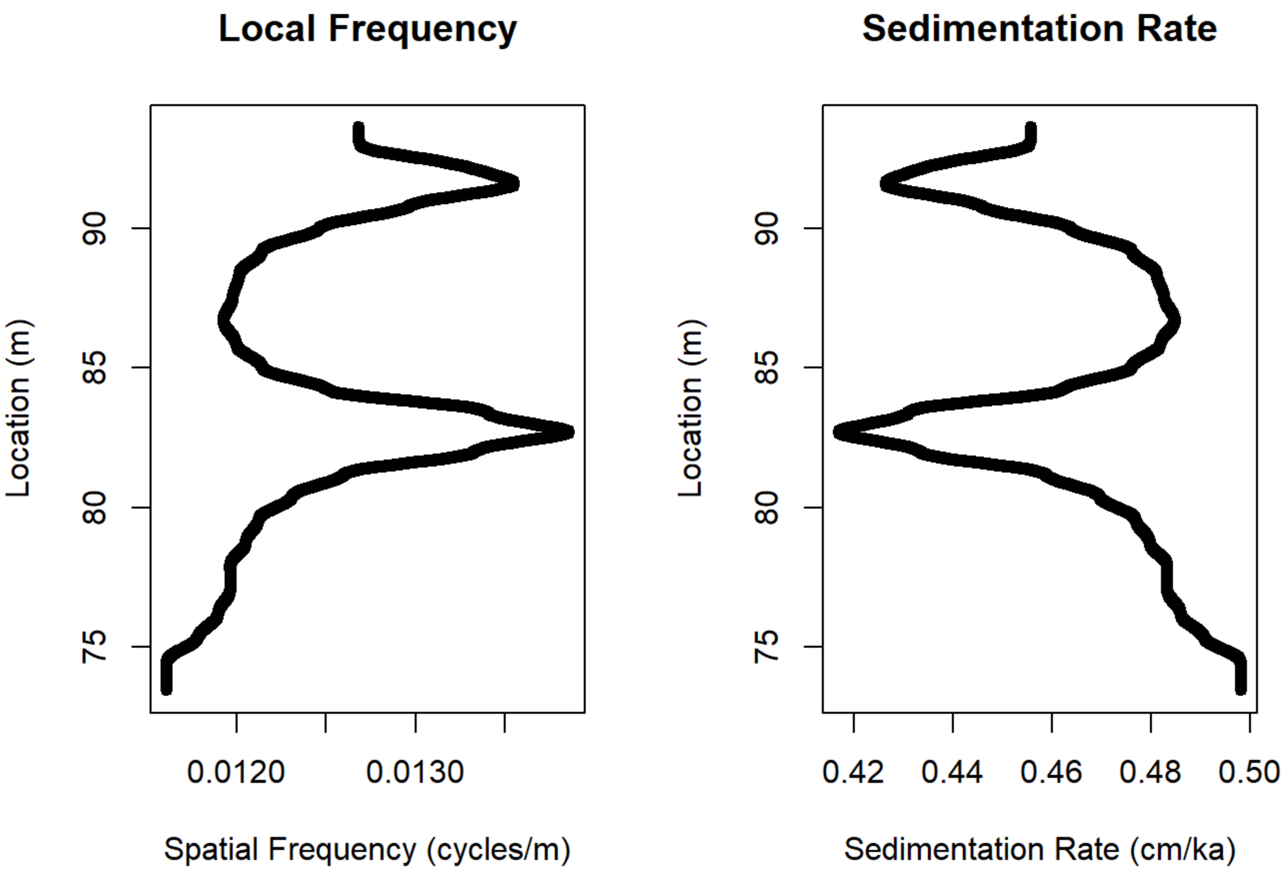


```
max_sed<-freq2sedrate(cbind(Det_173_freq[,1],
                            (Det_173_freq[,6]+2*Det_173_freq[,7])),
                      period=17300
                      )
```


```
----- CONVERTING RECORD OF SPATIAL FREQUENCY TO SEDIMENTATION RATE CURVE-----

 * Sorting into increasing depth/height order.
     Will remove empty entries.
 * Number of control points = 4032
```

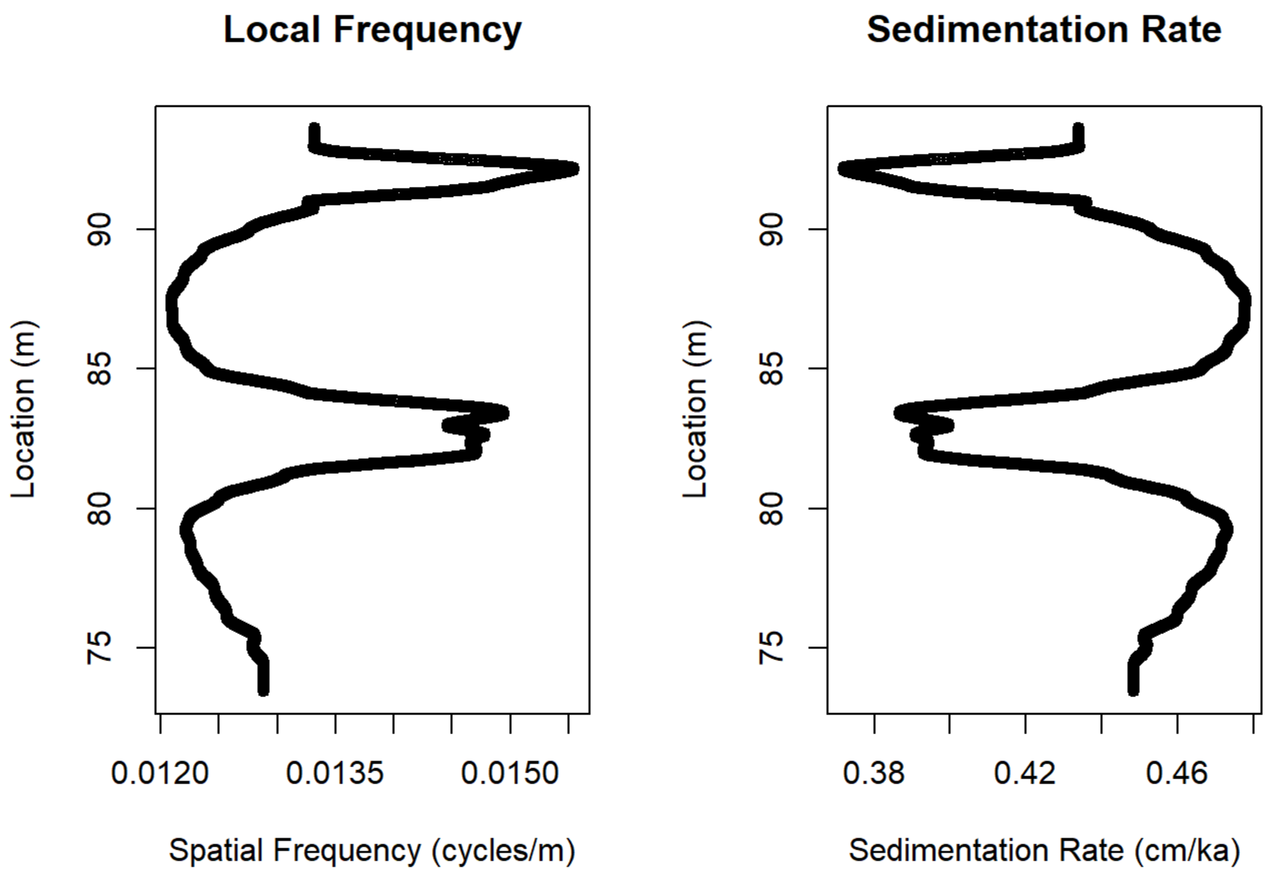


```
min_sed<-freq2sedrate(cbind(Det_173_freq[,1],
                            (Det_173_freq[,6]-2*Det_173_freq[,7])),
                      period=17300
                      )
```

```
----- CONVERTING RECORD OF SPATIAL FREQUENCY TO SEDIMENTATION RATE CURVE-----

 * Sorting into increasing depth/height order.
     Will remove empty entries.
 * Number of control points = 4032
```

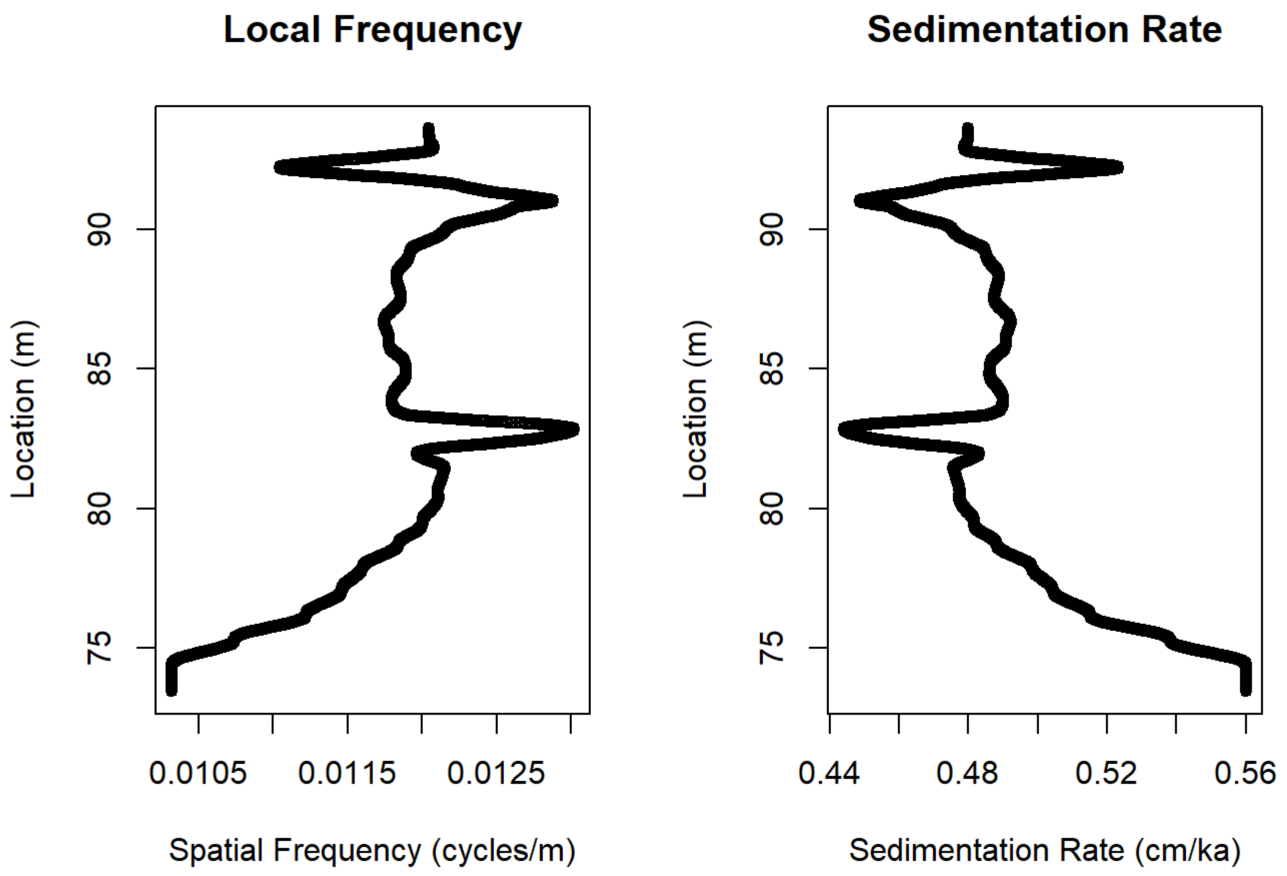


```
#Sed rate to time and creation fo the Age Model
Mean_AgeModel<-sedrate2time(mean_sed)
```

```
----- INTEGRATING SEDIMENTATION RATE CURVE-----

 * Sorting sedrates into increasing depth/height order.
     Will remove empty entries.
 * Number of sedimentation rates= 4032
```

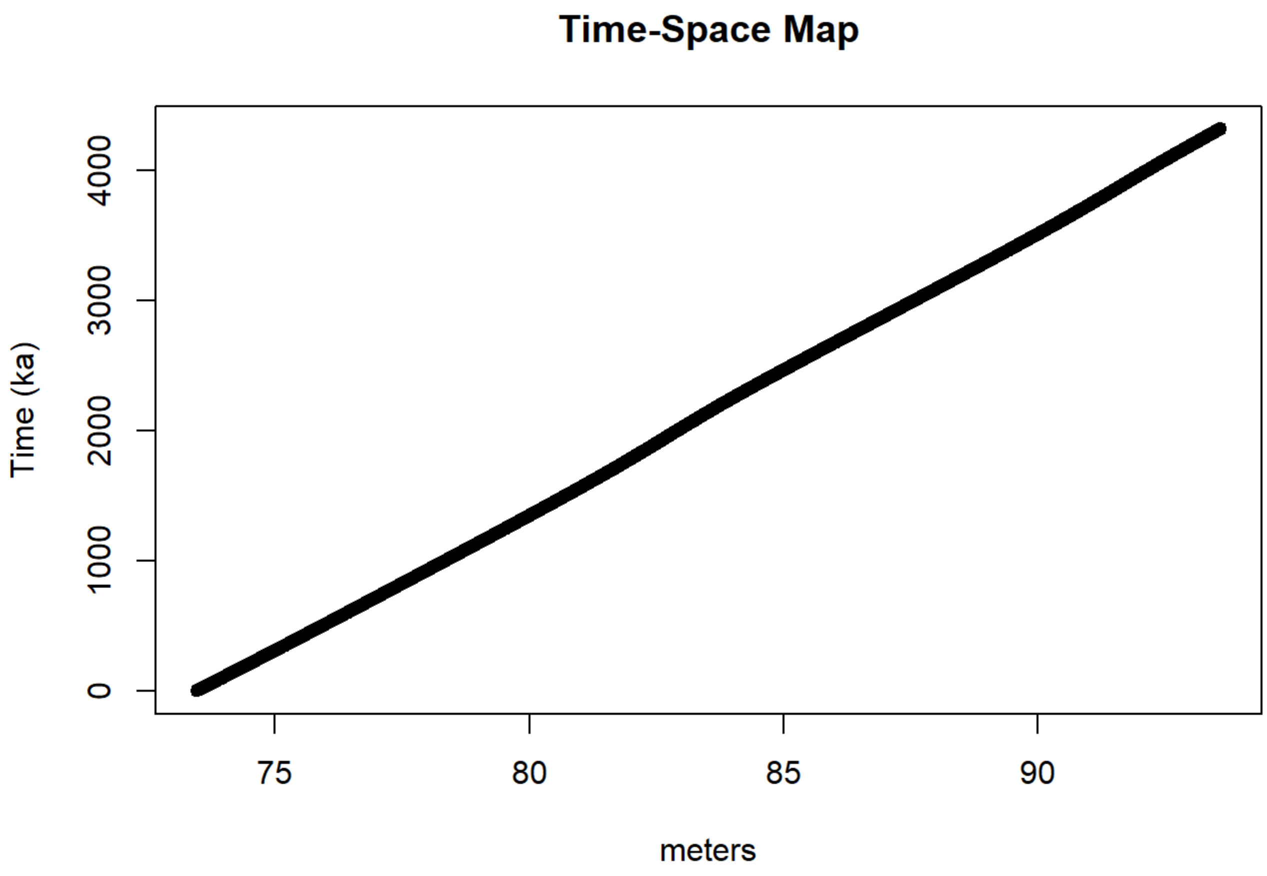


```
Max_AgeModel<-sedrate2time(max_sed)
```

```
----- INTEGRATING SEDIMENTATION RATE CURVE-----

 * Sorting sedrates into increasing depth/height order.
     Will remove empty entries.
 * Number of sedimentation rates= 4032
```

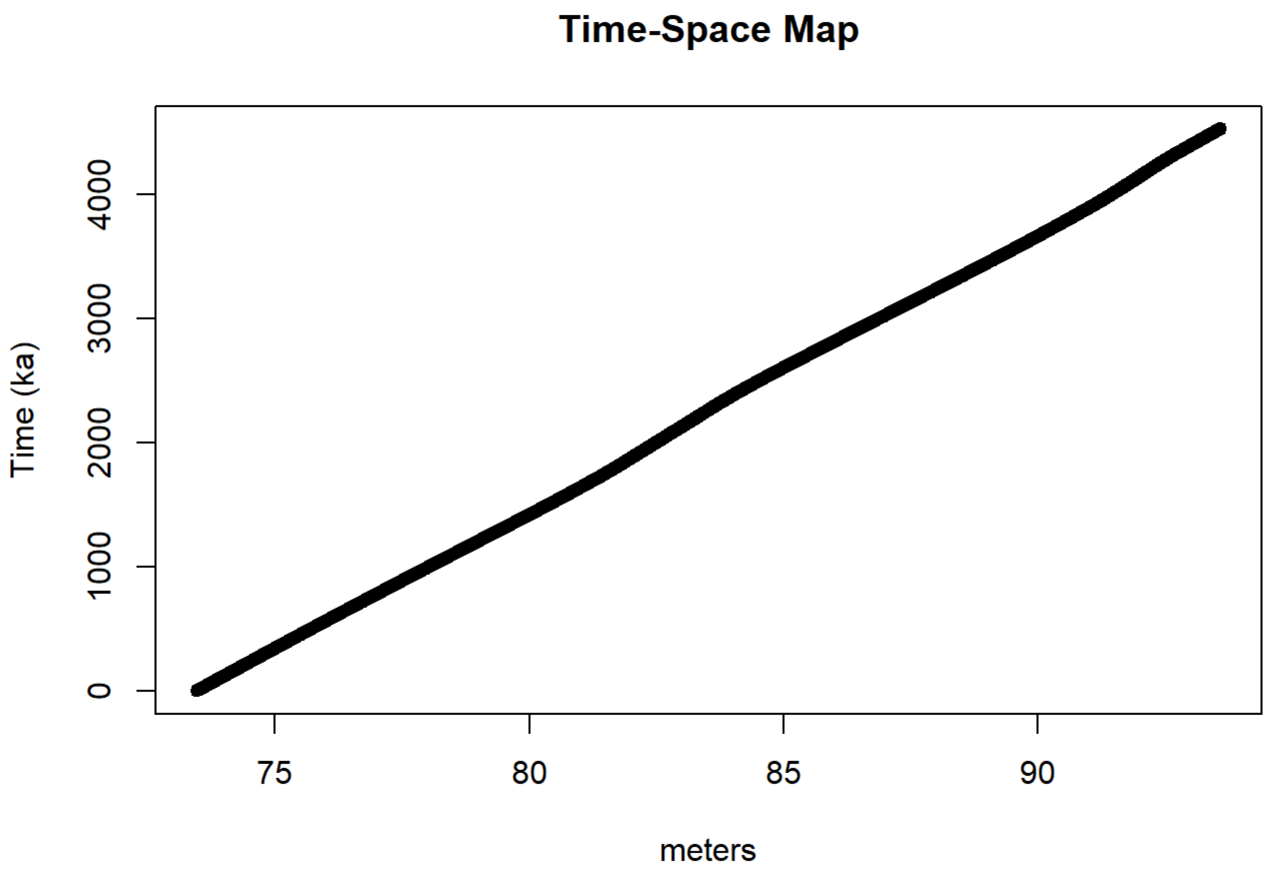


```
Min_AgeModel<-sedrate2time(min_sed)
```

```
----- INTEGRATING SEDIMENTATION RATE CURVE-----

 * Sorting sedrates into increasing depth/height order.
     Will remove empty entries.
 * Number of sedimentation rates= 4032
```

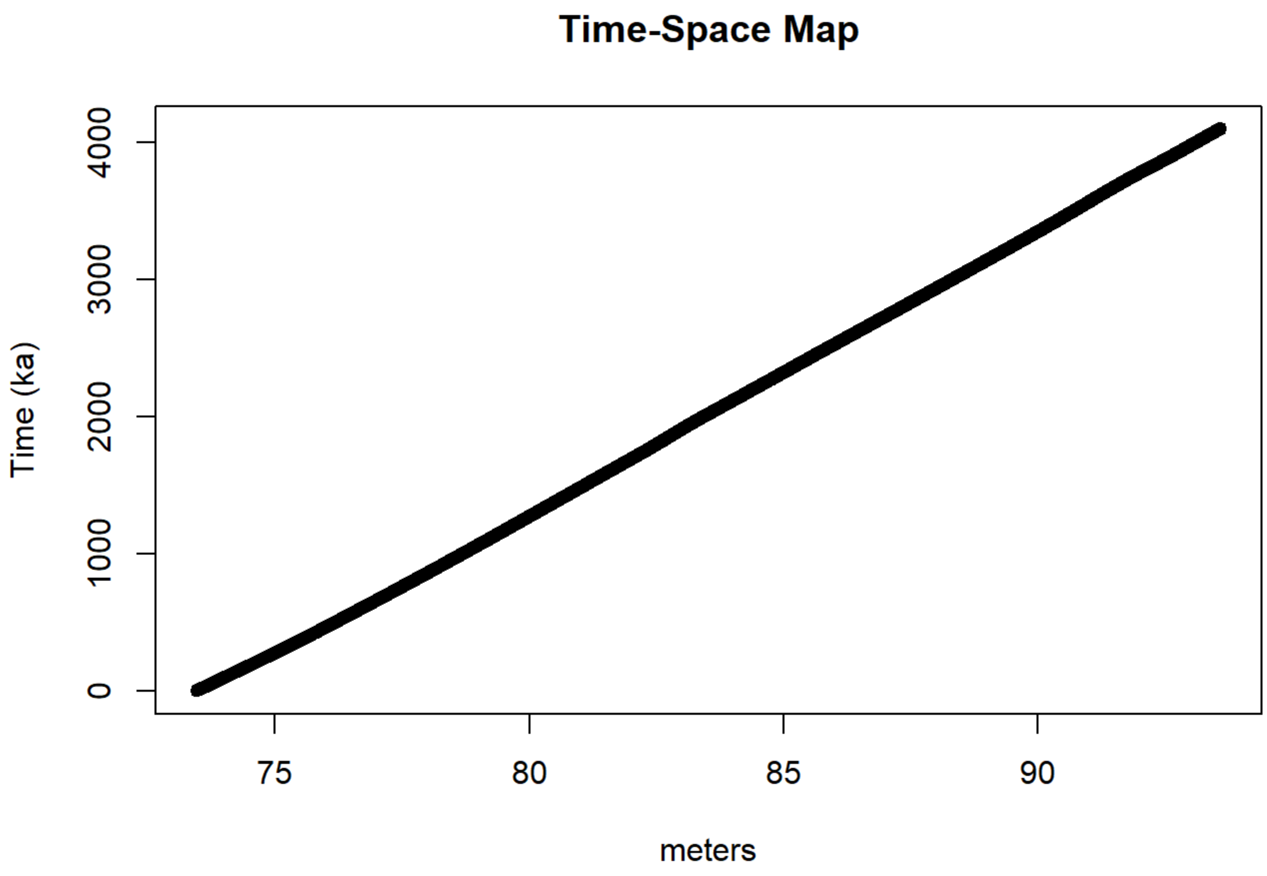


```
plot(Mean_AgeModel,type="l", lwd=2)
lines(Max_AgeModel,col="green",lwd=2)
lines(Min_AgeModel,col="blue",lwd=2)
```

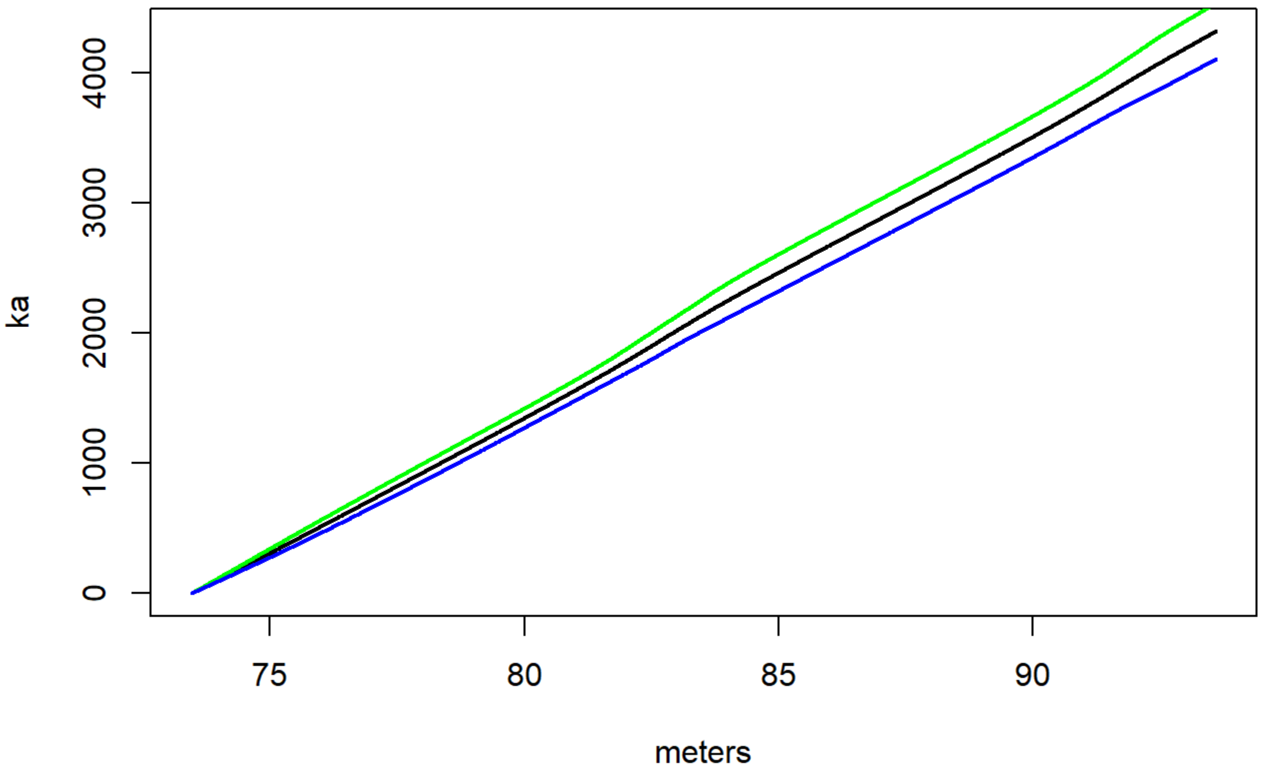


```
Age_Model <- cbind(Mean_AgeModel,Max_AgeModel[,2],Min_AgeModel[,2])
colnames(Age_Model)<- cbind("Adjusted depth (m)",
                            "Mean age (ka)",
                            "Max age (ka)",
                            "Min age (ka)"
                            )
#Interpolation of the Age-Model every 1 mm to have an age-depth correlation for
#the entire Ti dataset with the original sampling step of 1mm
Mean_Age_Model_1mm <- linterp(Mean_AgeModel, dt=0.001, genplot=T)
```

```
----- APPLYING PIECEWISE-LINEAR INTERPOLATION TO STRATIGRAPHIC SERIES -----

 * Number of samples= 4032
 * New number of samples= 20155
```

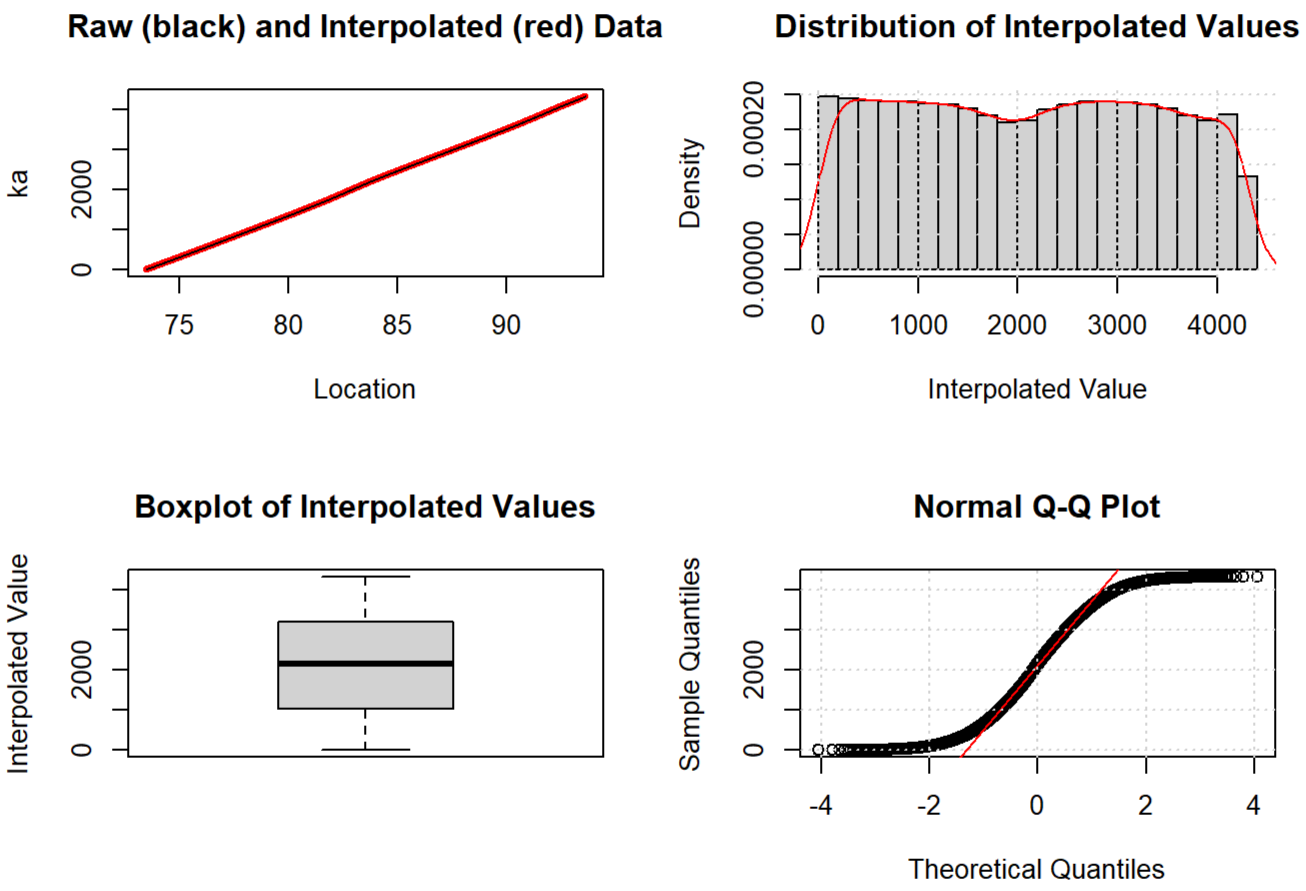


```r
Max_Age_Model_1mm <- linterp(Max_AgeModel, dt=0.001, genplot=T)
```

```
----- APPLYING PIECEWISE-LINEAR INTERPOLATION TO STRATIGRAPHIC SERIES -----

 * Number of samples= 4032
 * New number of samples= 20155
```

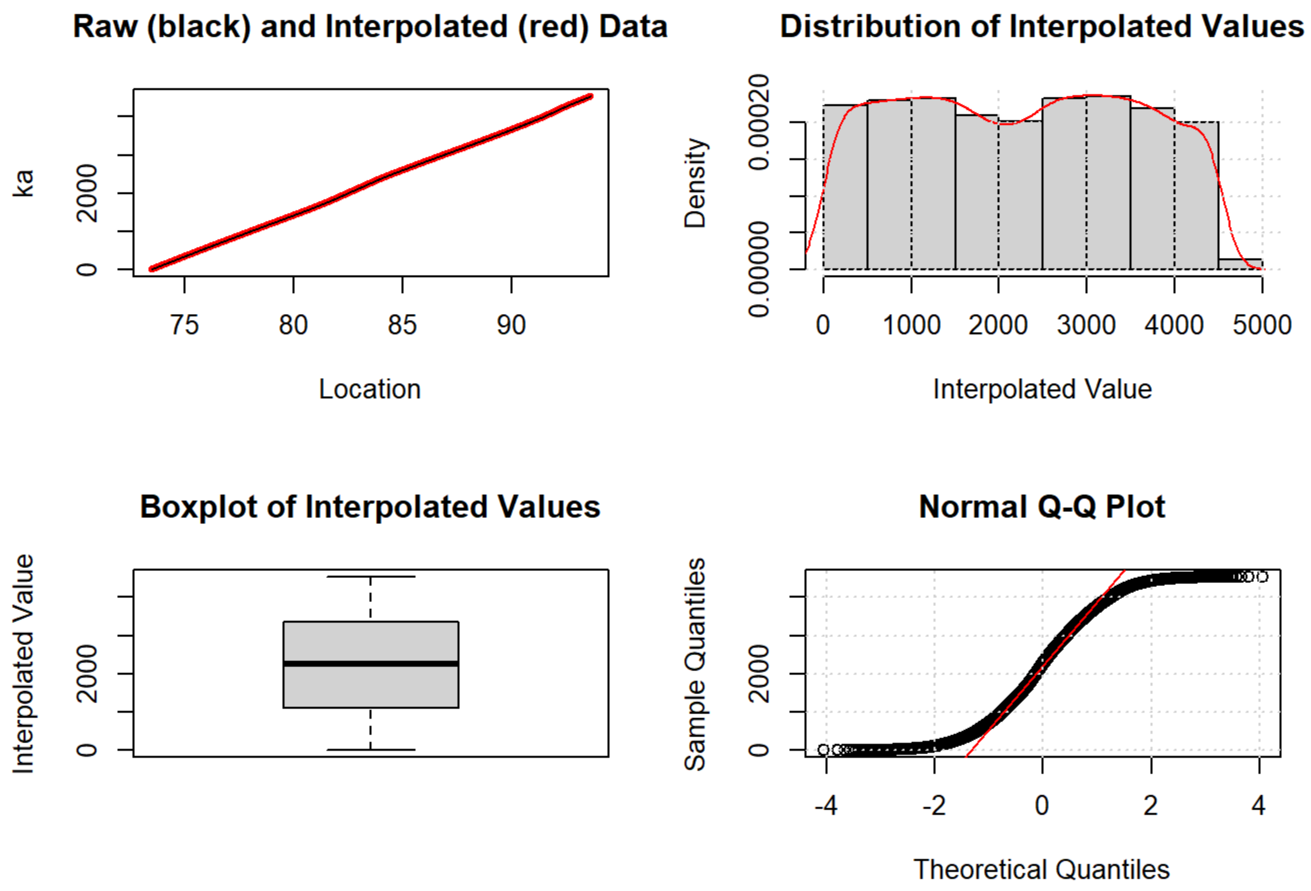


```
Min_Age_Model_1mm <- linterp(Min_AgeModel, dt=0.001, genplot=T)
```

```
----- APPLYING PIECEWISE-LINEAR INTERPOLATION TO STRATIGRAPHIC SERIES -----

 * Number of samples= 4032
 * New number of samples= 20155
```

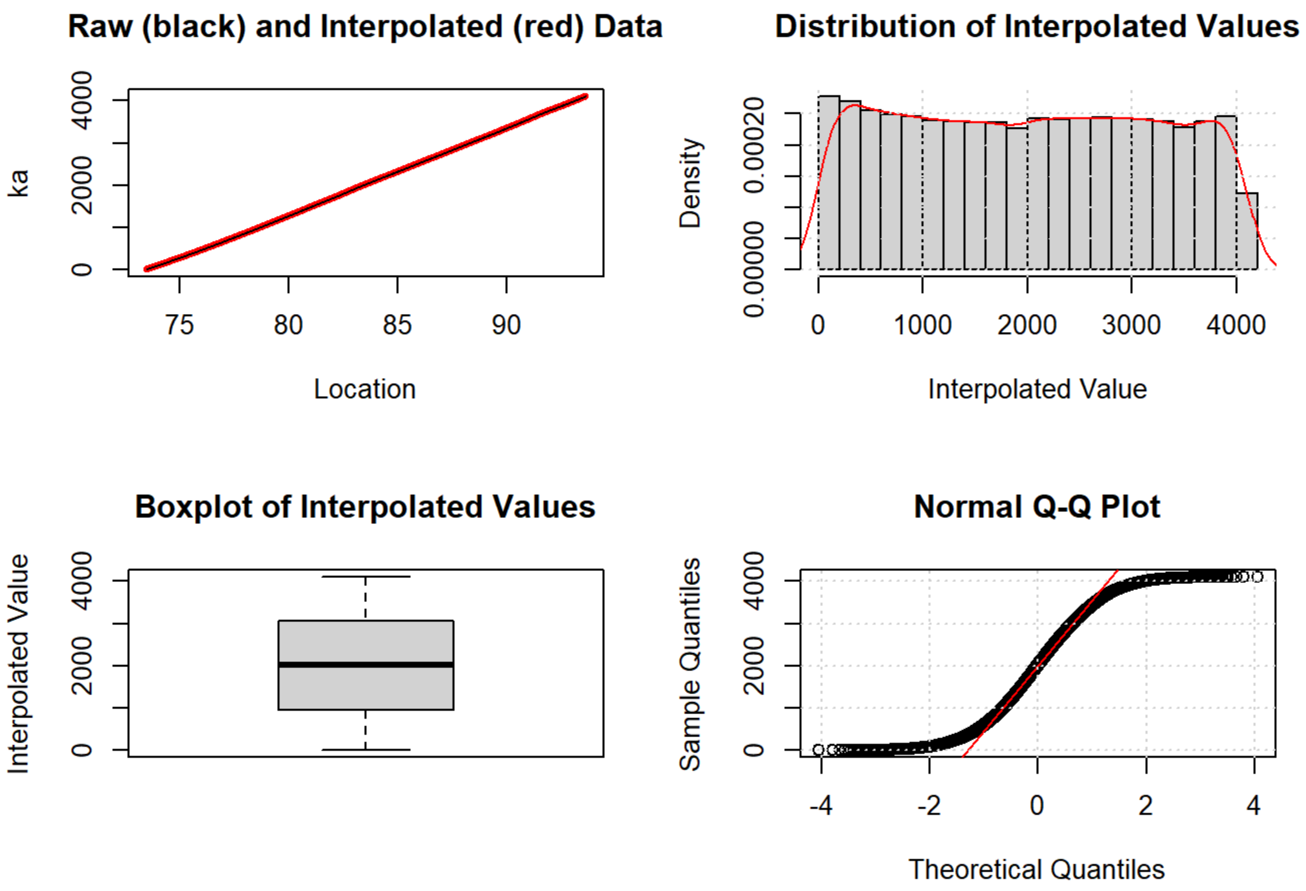


```
  Age_Model_1mm <- cbind(Mean_Age_Model_1mm,Max_Age_Model_1mm[,2],Min_Age_Model_1mm[,2])
  colnames(Age_Model)<- cbind("Adjusted depth (m)",
                              "Mean age (ka)",
                              "Max age (ka)",
                              "Min age (ka)"
                              )
  #Saving of the age models as .csv files
#  write.csv(Age_Model_1mm,"AgeModel_rsp1mm_filter0.75-0.97m.csv")
```

## 11.2. Age-Depth model (Wavelet tracking; preferred)

### 11.2.1. Tracking of the 173 kyr for Ti, Al, Si and K

As shown in the time series analysis (MTM, EHA and CWT), the 173 kyr cycle is comprise between 0.75 and 0.9 m. To avoid overpass this range when tracking the Wavelet, especially with periodicities lower than 0.74 m, that could be linked to the short eccentricity (100-135 kyr) band, we used a lowpass filtering of the tracked Wavelet in accordance with the general trend observed in the CWT.

#### 11.2.1.1. Titanium

```
    alb_Ti <- cbind(Alb$DepthAdj,Alb$Ti)
    alb_Ti <- na.omit(alb_Ti)
    alb_Ti[!is.finite(alb_Ti)] <- NA
```

```
alb_Ti <- na.omit(alb_Ti)
alb_Ti <- iso(dat=alb_Ti, xmin=73.453, xmax=100)
```

```
----- ISOLATE STRATIGRAPHIC DATA BY LOCATION -----
 * Number of data points= 30577
 * Number of columns= 2
 * Minimum= 62.111 , Maximum= 93.61
 * Isolating data between 73.453 and 100
 * Number of data points following culling= 19311
```

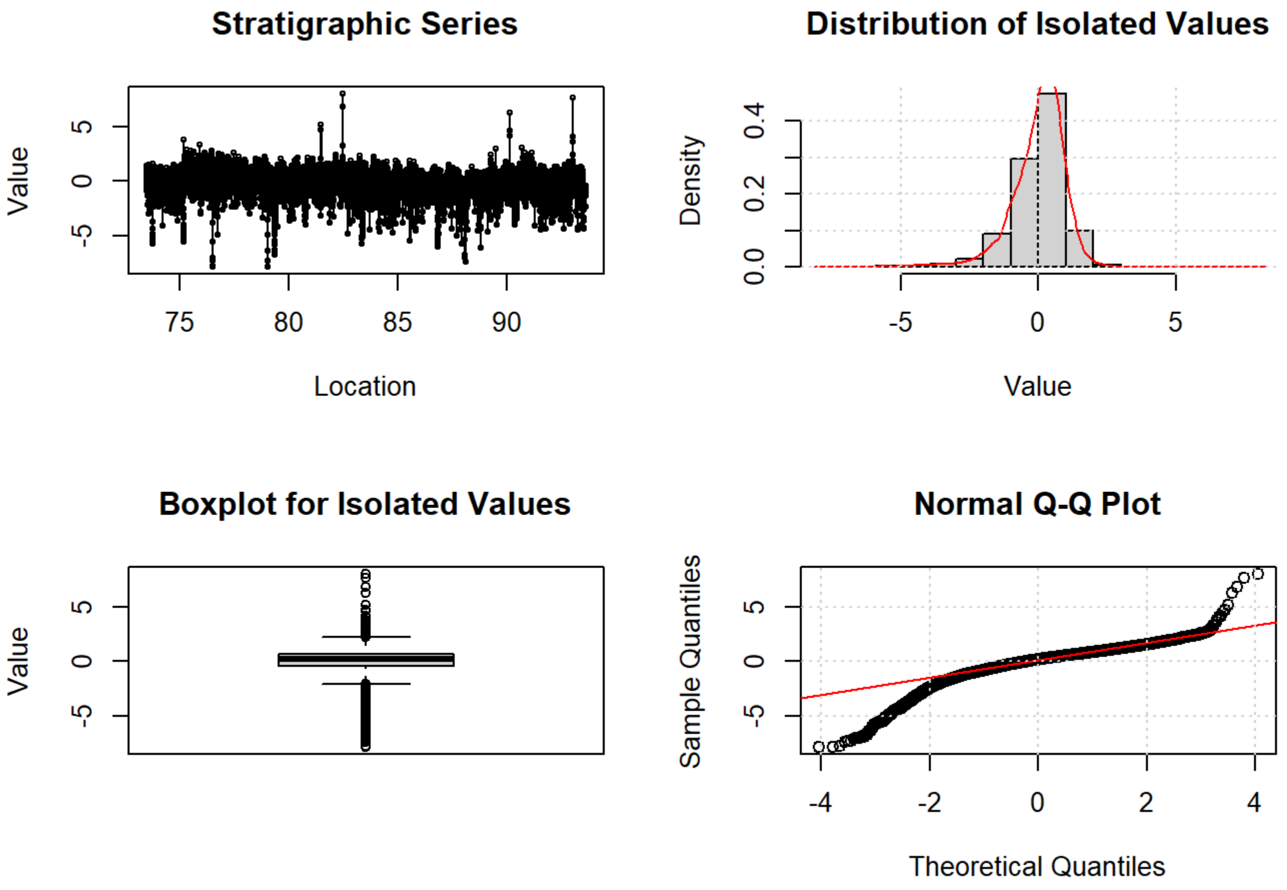


```
alb_Ti_ndet <-  linterp(alb_Ti, dt=0.005, genplot=T)
```

```
----- APPLYING PIECEWISE-LINEAR INTERPOLATION TO STRATIGRAPHIC SERIES -----

 * Number of samples= 19311
 * New number of samples= 4032
```

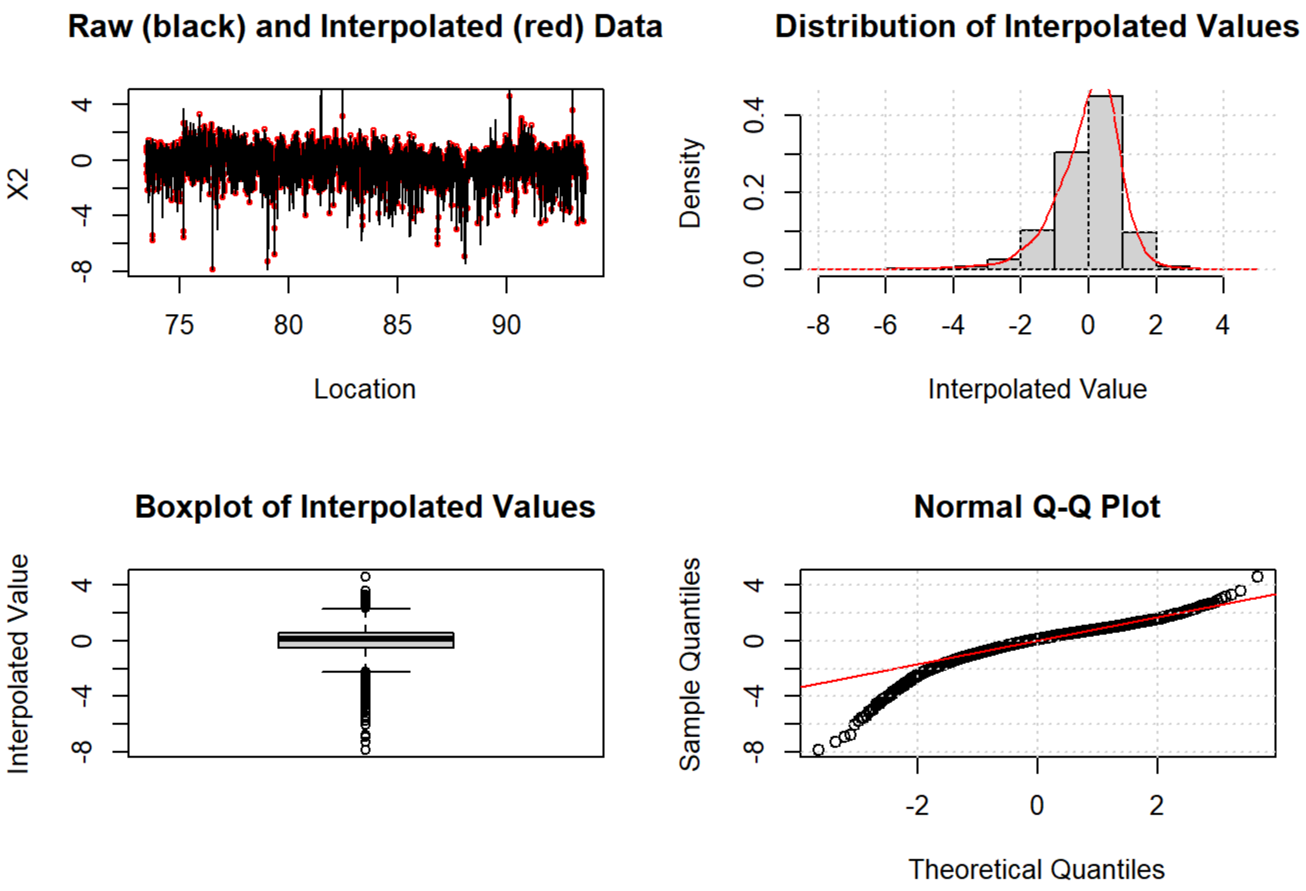


```r
#Waverider
alb_Ti_wt <- analyze_wavelet(alb_Ti_ndet,
                             dj = 1/100,
                             lowerPeriod = 0.5,
                             upperPeriod = 5,
                             verbose = FALSE,
                             omega_nr = 10
                             )

plot_wavelet(wavelet = alb_Ti_wt,
             lowerPeriod = NULL,
             upperPeriod = NULL,
             n.levels = 100,
             palette_name = "rainbow",
             color_brewer = "grDevices",
             useRaster = TRUE,
             periodlab = "Period (metres)",
             x_lab = "Depth (metres)",
             keep_editable = FALSE,
             dev_new = F,
             add_lines = NULL,
             add_points = NULL,
             add_abline_h = NULL,
             add_abline_v = NULL,
             add_MTM_peaks = FALSE,
             add_data = TRUE,
             add_avg = TRUE,
```

```
                 add_MTM = FALSE,
                 demean_mtm = TRUE,
                 detrend_mtm = TRUE,
                 padfac_mtm = 5,
                 tbw_mtm = 3,
                 plot_horizontal = TRUE
                 )
```

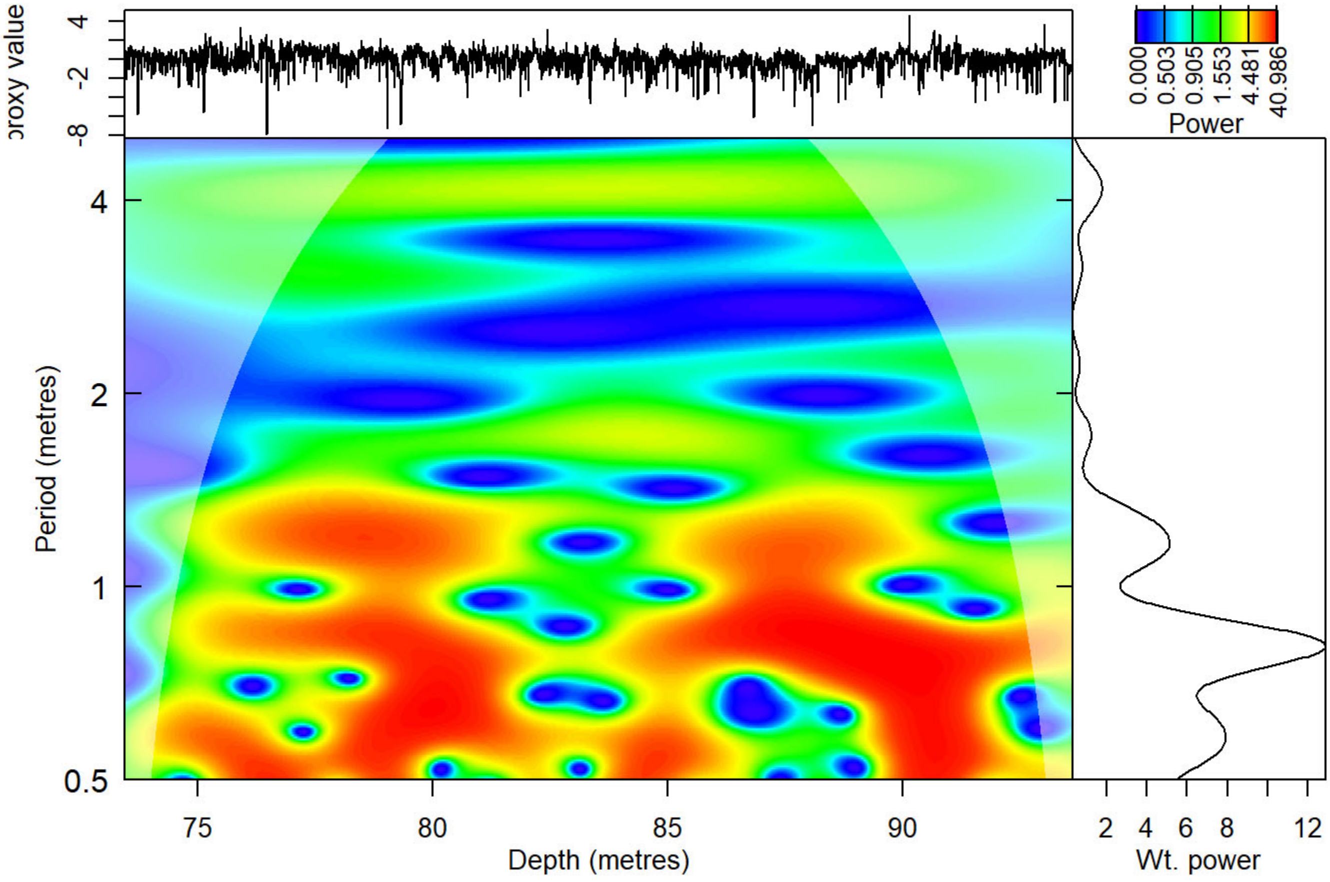


```
    #track the period (m) of the 173 kyr cycle
#    alb_Ti_track_WR <- track_period_wavelet(astro_cycle = 173,
#                                        wavelet = alb_Ti_wt,
#                                        n.levels = 100,
#                                        periodlab = "Period (metres)",
#                                        x_lab = "depth (metres)",
#                                        palette_name = "rainbow",
#                                        color_brewer = "grDevices",
#                                        plot_horizontal = TRUE
#                                        )

#   alb_Ti_track_WR_comp <- completed_series(wavelet = alb_Ti_wt,
#                                        tracked_curve = alb_Ti_track_WR,
#                                        period_up = 1,
#                                        period_down = 0.70,
#                                        extrapolate = TRUE,
#                                        genplot = FALSE,
#                                        keep_editable = FALSE
#                                        )
```

```r
#    alb_Ti_track_WR_comp[alb_Ti_track_WR_comp[,2]<0.74,2]<-0.74

#    alb_Ti_track_WR_comp <- loess_auto(alb_Ti_track_WR_comp)

#    write.csv(alb_Ti_track_WR_comp,"alb_Ti_track_WR.csv")

# Loading the tracked curve
    alb_Ti_track_WR_comp <- read.csv("alb_Ti_track_WR.csv")
    alb_Ti_track_WR_comp <- alb_Ti_track_WR_comp[,c(2,3)]


    plot_wavelet(wavelet = alb_Ti_wt,
                 lowerPeriod = NULL,
                 upperPeriod = NULL,
                 n.levels = 100,
                 palette_name = "rainbow",
                 color_brewer = "grDevices",
                 useRaster = TRUE,
                 periodlab = "Period (metres)",
                 x_lab = "Depth (metres)",
                 keep_editable = FALSE,
                 dev_new = F,
                 add_lines = cbind(alb_Ti_track_WR_comp[,1],
                                   alb_Ti_track_WR_comp[,2]),
                 add_points = NULL,
                 add_abline_h = NULL,
                 add_abline_v = NULL,
                 add_MTM_peaks = FALSE,
                 add_data = TRUE,
                 add_avg = TRUE,
                 add_MTM = FALSE,
                 demean_mtm = TRUE,
                 detrend_mtm = TRUE,
                 padfac_mtm = 5,
                 tbw_mtm = 3,
                 plot_horizontal = TRUE
                 )
```

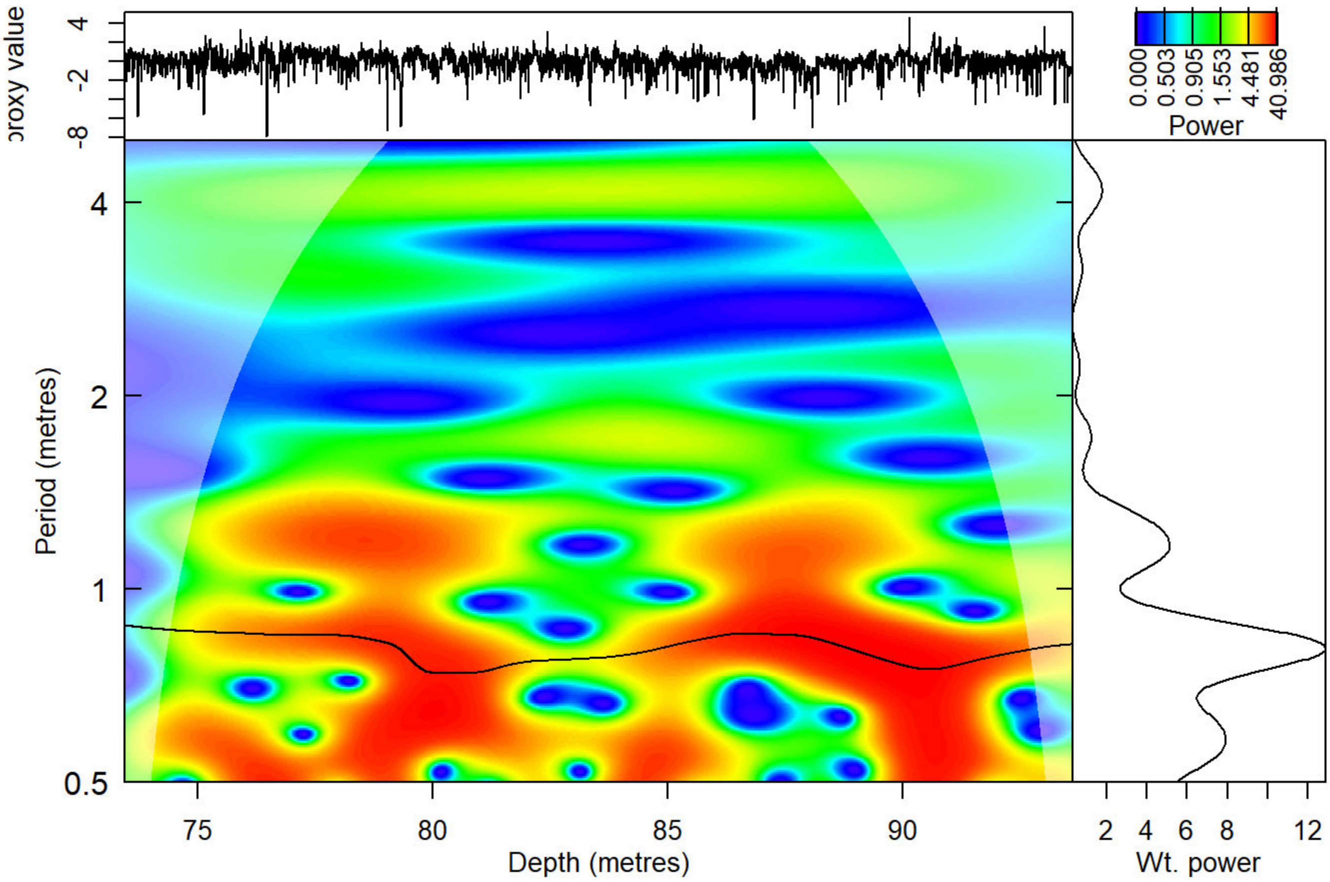


### 11.2.1.2. Silicon

```
alb_Si <- cbind(Alb$DepthAdj,Alb$Si)
  alb_Si <- na.omit(alb_Si)
  alb_Si[!is.finite(alb_Si)] <- NA
  alb_Si <- na.omit(alb_Si)
  alb_Si <- iso(dat=alb_Si, xmin=73.453, xmax=100)
```

```
----- ISOLATE STRATIGRAPHIC DATA BY LOCATION -----
 * Number of data points= 30577
 * Number of columns= 2
 * Minimum= 62.111 , Maximum= 93.61
 * Isolating data between 73.453 and 100
 * Number of data points following culling= 19311
```

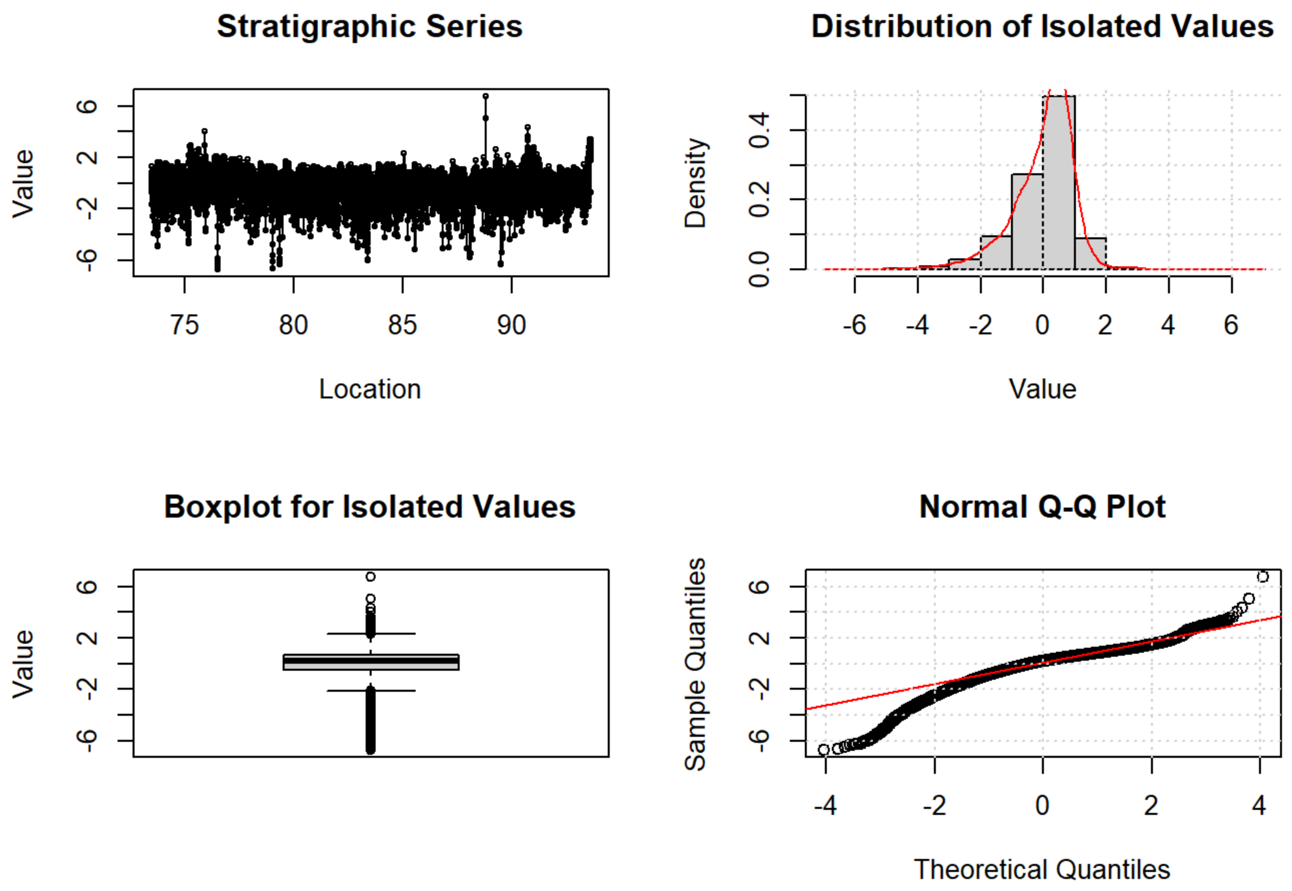


```
alb_Si_ndet <-  linterp(alb_Si, dt=0.005, genplot=T)
```

```
----- APPLYING PIECEWISE-LINEAR INTERPOLATION TO STRATIGRAPHIC SERIES -----

 * Number of samples= 19311
 * New number of samples= 4032
```

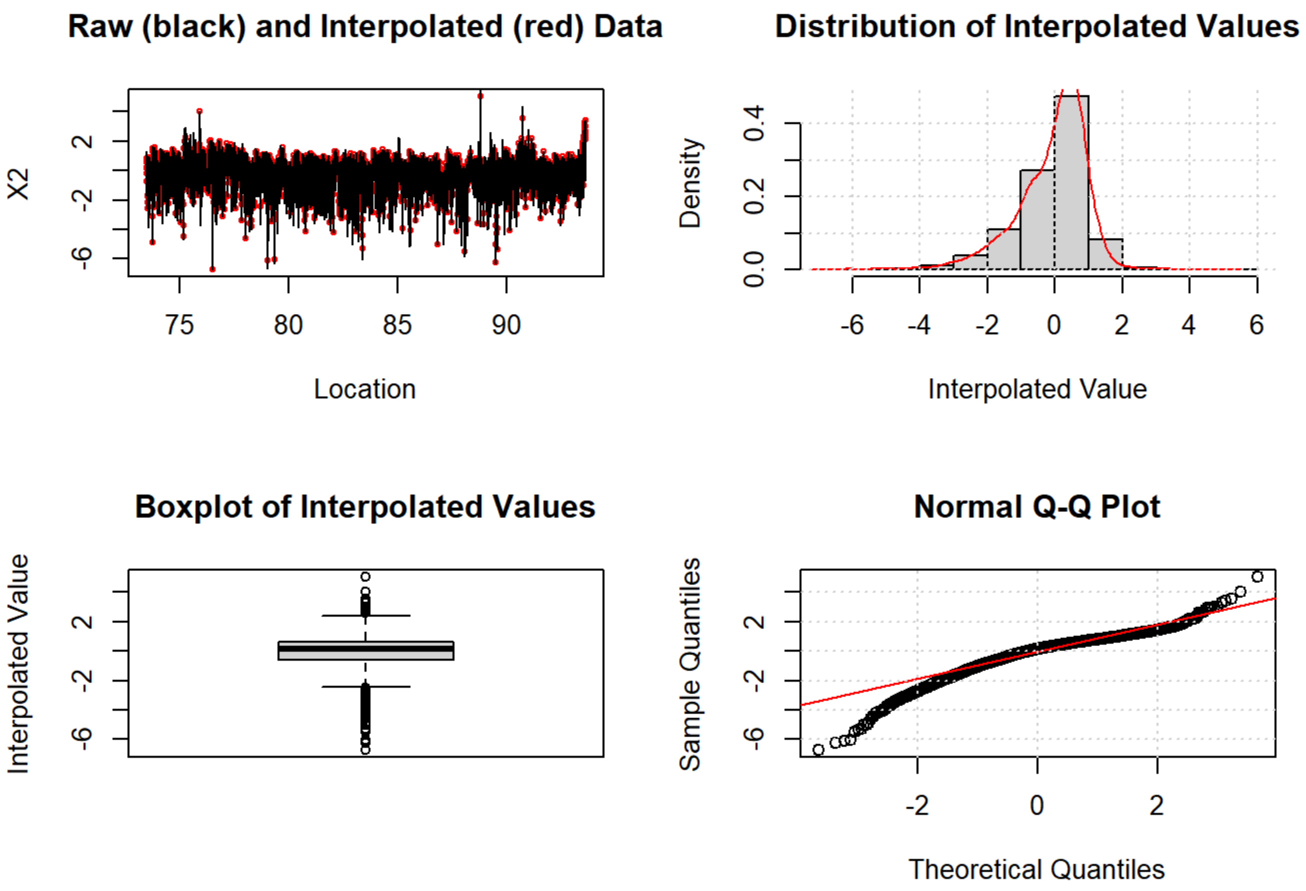


```r
#Waverider
alb_Si_wt <- analyze_wavelet(alb_Si_ndet,
                             dj = 1/100,
                             lowerPeriod = 0.5,
                             upperPeriod = 5,
                             verbose = FALSE,
                             omega_nr = 10
                             )

plot_wavelet(wavelet = alb_Si_wt,
             lowerPeriod = NULL,
             upperPeriod = NULL,
             n.levels = 100,
             palette_name = "rainbow",
             color_brewer = "grDevices",
             useRaster = TRUE,
             periodlab = "Period (metres)",
             x_lab = "Depth (metres)",
             keep_editable = FALSE,
             dev_new = F,
             add_lines = NULL,
             add_points = NULL,
             add_abline_h = NULL,
             add_abline_v = NULL,
             add_MTM_peaks = FALSE,
             add_data = TRUE,
             add_avg = TRUE,
```

```
add_MTM = FALSE,
demean_mtm = TRUE,
detrend_mtm = TRUE,
padfac_mtm = 5,
tbw_mtm = 3,
plot_horizontal = TRUE
)
```

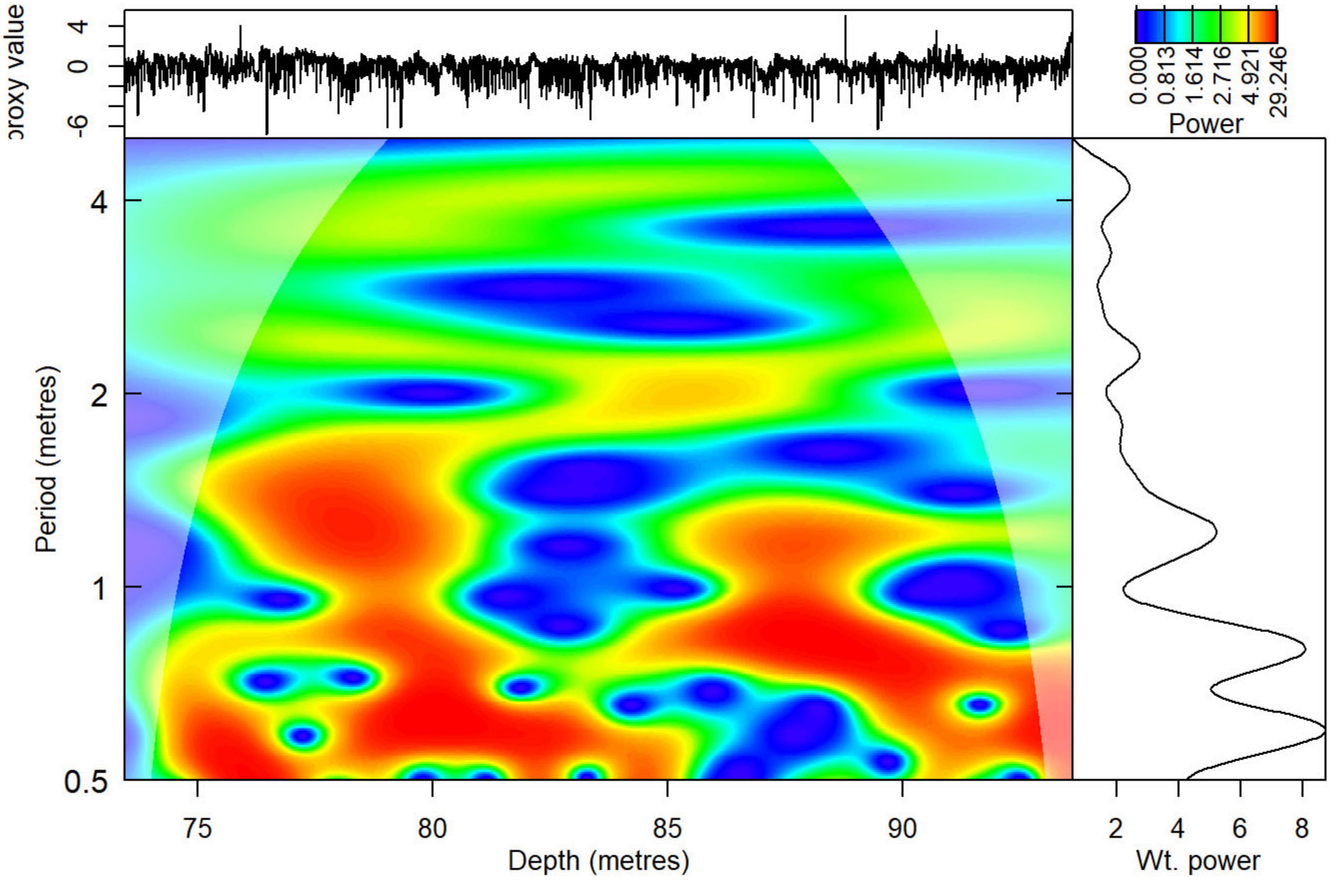


```
    #track the period (m) of the 173 kyr cycle
#    alb_Si_track_WR <- track_period_wavelet(astro_cycle = 173,
#                                         wavelet = alb_Si_wt,
#                                         n.levels = 100,
#                                         periodlab = "Period (metres)",
#                                         x_lab = "depth (metres)",
#                                         palette_name = "rainbow",
#                                         color_brewer = "grDevices",
#                                         plot_horizontal = TRUE
#                                         )

#   alb_Si_track_WR_comp <- completed_series(wavelet = alb_Si_wt,
#                                         tracked_curve = alb_Si_track_WR,
#                                         period_up = 1,
#                                         period_down = 0.70,
#                                         extrapolate = TRUE,
#                                         genplot = FALSE,
#                                         keep_editable = FALSE
#                                         )
```

```r
#    alb_Si_track_WR_comp[alb_Si_track_WR_comp[,2]<0.74,2]<-0.74

  #To keep a similar trend in the Si data in the 73-80 m interval, when tracking,
  #we deliberately went for a higher band (above 0.9 m) and we implemented a
  #highpass filter for all the values above 0.86m to keep a similar trend between
  #Si and the other detrital elements

#     alb_Si_track_WR_comp[alb_Si_track_WR_comp[,2]>0.86,2]<-0.81

#     alb_Si_track_WR_comp <- loess_auto(alb_Si_track_WR_comp)

#     write.csv(alb_Si_track_WR_comp,"alb_Si_track_WR.csv")


# Loading the tracked curve
    alb_Si_track_WR_comp <- read.csv("alb_Si_track_WR.csv")
    alb_Si_track_WR_comp <- alb_Si_track_WR_comp[,c(2,3)]

    plot_wavelet(wavelet = alb_Si_wt,
                 lowerPeriod = NULL,
                 upperPeriod = NULL,
                 n.levels = 100,
                 palette_name = "rainbow",
                 color_brewer = "grDevices",
                 useRaster = TRUE,
                 periodlab = "Period (metres)",
                 x_lab = "Depth (metres)",
                 keep_editable = FALSE,
                 dev_new = F,
                 add_lines = cbind(alb_Si_track_WR_comp[,1],
                                   alb_Si_track_WR_comp[,2]),
                 add_points = NULL,
                 add_abline_h = NULL,
                 add_abline_v = NULL,
                 add_MTM_peaks = FALSE,
                 add_data = TRUE,
                 add_avg = TRUE,
                 add_MTM = FALSE,
                 demean_mtm = TRUE,
                 detrend_mtm = TRUE,
                 padfac_mtm = 5,
                 tbw_mtm = 3,
                 plot_horizontal = TRUE
                 )
```

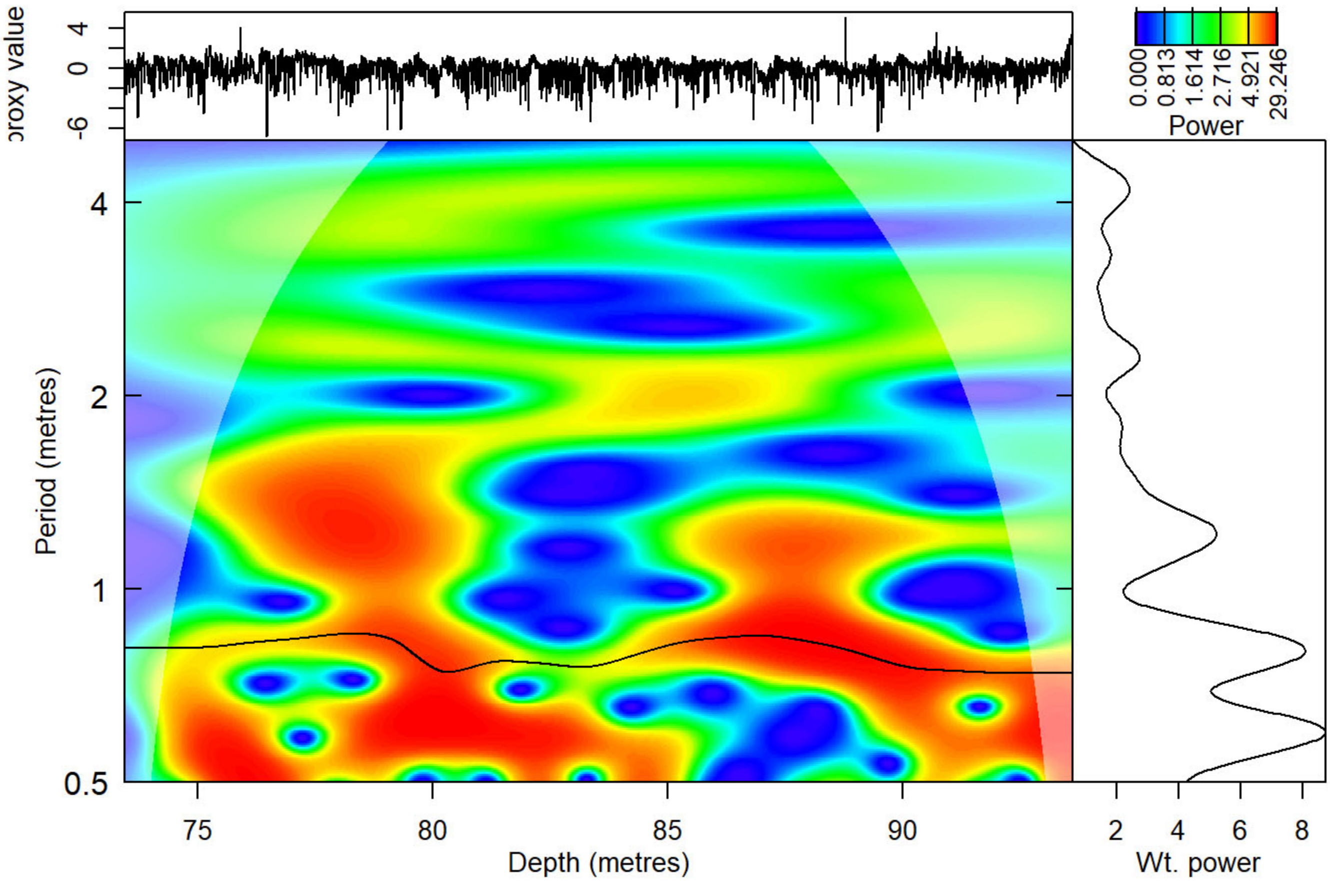


### 11.2.1.3. Aluminium

```
alb_Al <- cbind(Alb$DepthAdj,Alb$Al)
alb_Al <- na.omit(alb_Al)
alb_Al[!is.finite(alb_Al)] <- NA
alb_Al <- na.omit(alb_Al)
alb_Al <- iso(dat=alb_Al, xmin=73.453, xmax=100)
```

```
----- ISOLATE STRATIGRAPHIC DATA BY LOCATION -----
 * Number of data points= 30062
 * Number of columns= 2
 * Minimum= 62.111 , Maximum= 93.609
 * Isolating data between 73.453 and 100
 * Number of data points following culling= 18796
```

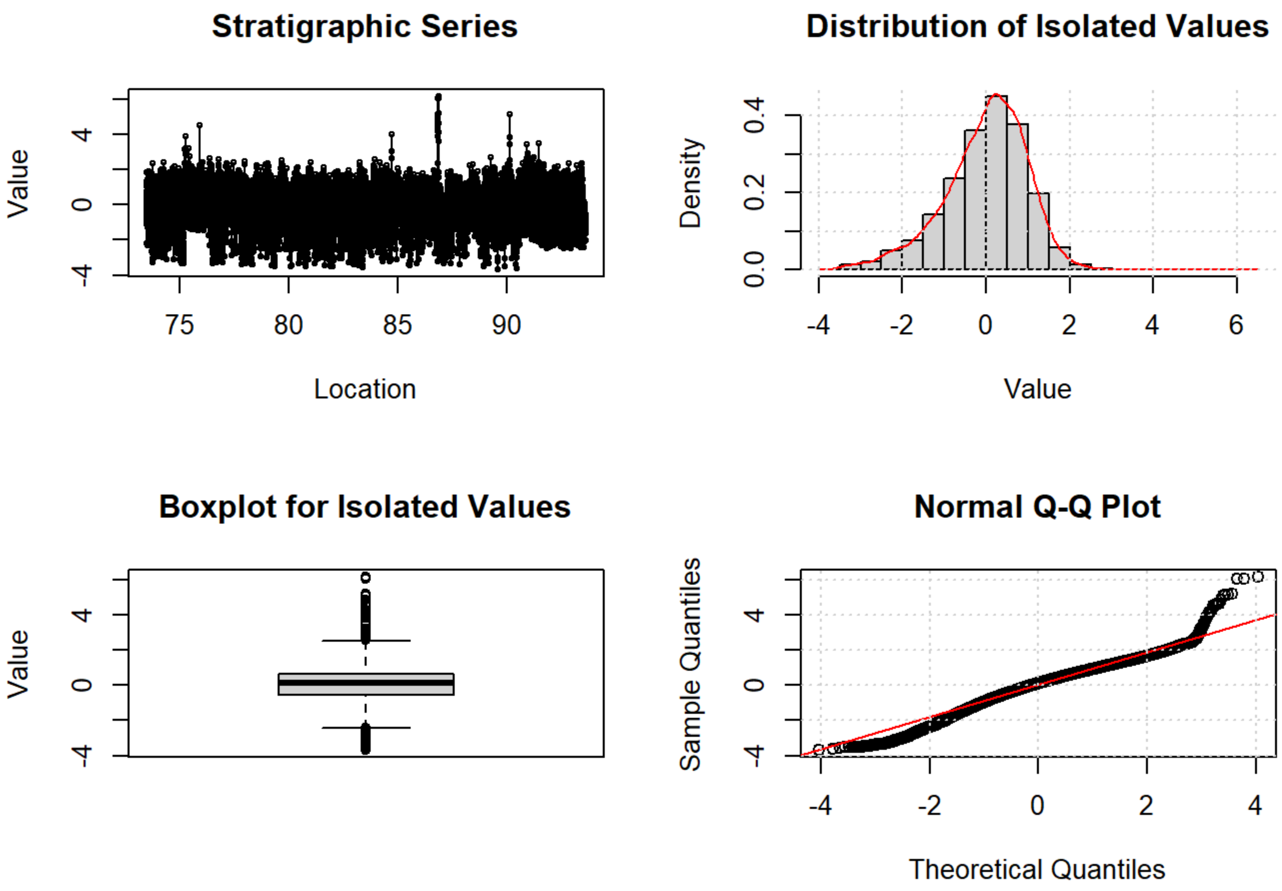


```r
alb_Al_ndet <-  linterp(alb_Al, dt=0.005, genplot=T)
```

```
----- APPLYING PIECEWISE-LINEAR INTERPOLATION TO STRATIGRAPHIC SERIES -----

 * Number of samples= 18796
 * New number of samples= 4032
```

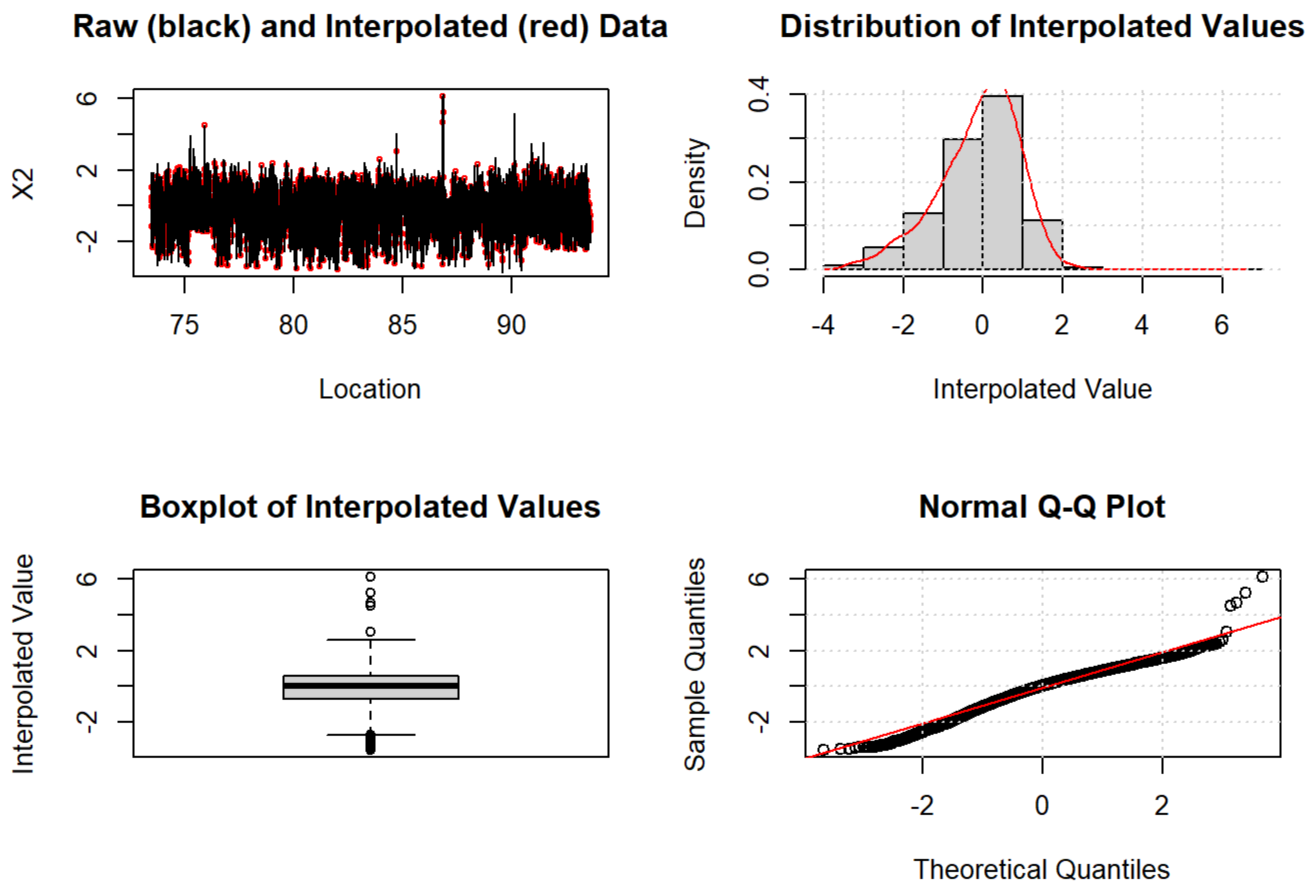


```r
#Waverider
alb_Al_wt <- analyze_wavelet(alb_Al_ndet,
                             dj = 1/100,
                             lowerPeriod = 0.5,
                             upperPeriod = 5,
                             verbose = FALSE,
                             omega_nr = 10
                             )

plot_wavelet(wavelet = alb_Al_wt,
             lowerPeriod = NULL,
             upperPeriod = NULL,
             n.levels = 100,
             palette_name = "rainbow",
             color_brewer = "grDevices",
             useRaster = TRUE,
             periodlab = "Period (metres)",
             x_lab = "Depth (metres)",
             keep_editable = FALSE,
             dev_new = F,
             add_lines = NULL,
             add_points = NULL,
             add_abline_h = NULL,
             add_abline_v = NULL,
             add_MTM_peaks = FALSE,
             add_data = TRUE,
             add_avg = TRUE,
```

```
                 add_MTM = FALSE,
                 demean_mtm = TRUE,
                 detrend_mtm = TRUE,
                 padfac_mtm = 5,
                 tbw_mtm = 3,
                 plot_horizontal = TRUE
                 )
```

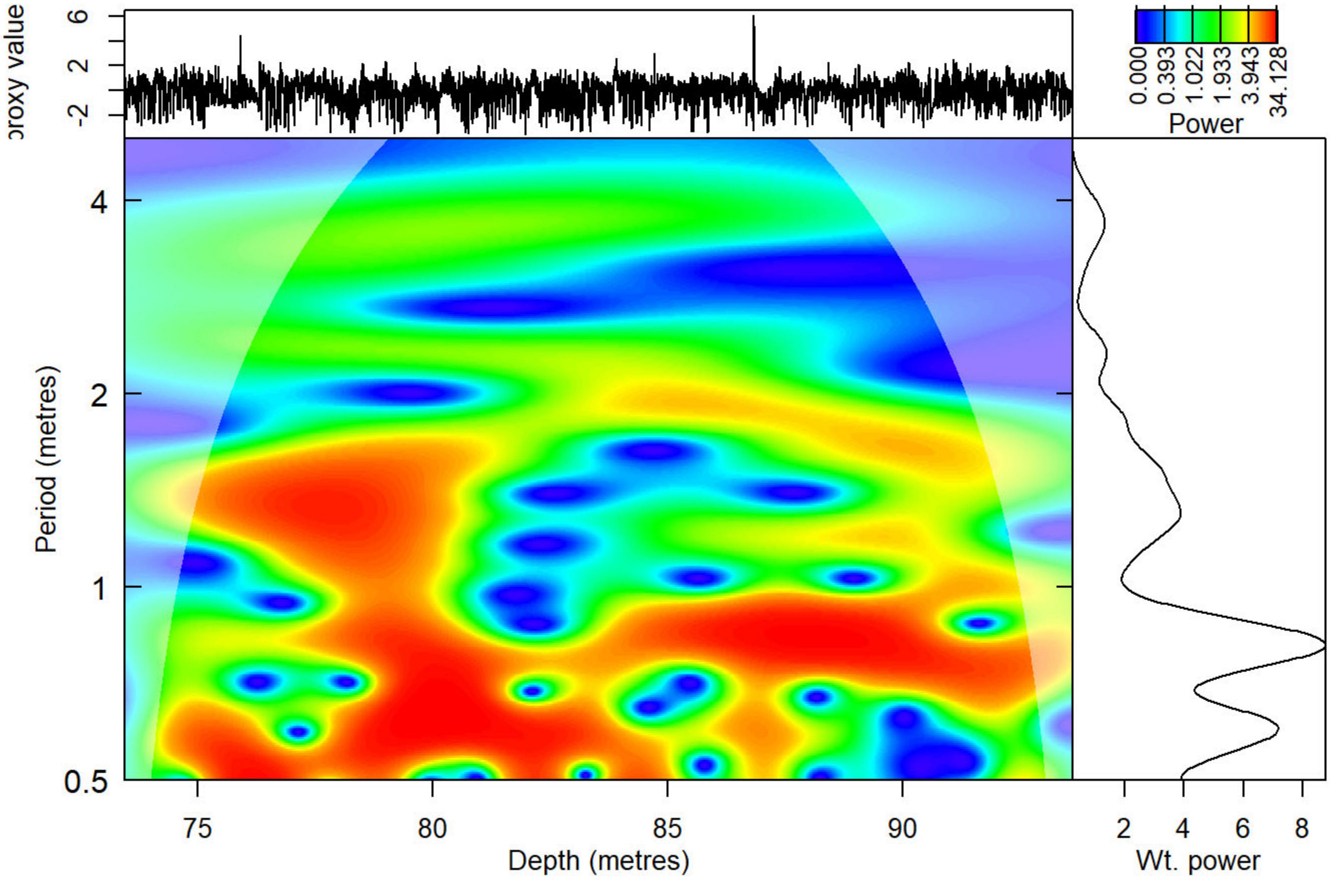


```
    #track the period (m) of the 173 kyr cycle
#   alb_Al_track_WR <- track_period_wavelet(astro_cycle = 173,
#                                           wavelet = alb_Al_wt,
#                                           n.levels = 100,
#                                           periodlab = "Period (metres)",
#                                           x_lab = "depth (metres)",
#                                           palette_name = "rainbow",
#                                           color_brewer = "grDevices",
#                                           plot_horizontal = TRUE
#                                           )

#   alb_Al_track_WR_comp <- completed_series(wavelet = alb_Al_wt,
#                                            tracked_curve = alb_Al_track_WR,
#                                            period_up = 1,
#                                            period_down = 0.70,
#                                            extrapolate = TRUE,
#                                            genplot = FALSE,
#                                            keep_editable = FALSE
#                                            )
```

```
#     alb_Al_track_WR_comp[alb_Al_track_WR_comp[,2]<0.74,2]<-0.74

#     alb_Al_track_WR_comp <- loess_auto(alb_Al_track_WR_comp)

#     write.csv(alb_Al_track_WR_comp,"alb_Al_track_WR.csv")


# Loading the tracked curve
    alb_Al_track_WR_comp <- read.csv("alb_Al_track_WR.csv")
    alb_Al_track_WR_comp <- alb_Al_track_WR_comp[,c(2,3)]

    plot_wavelet(wavelet = alb_Al_wt,
                 lowerPeriod = NULL,
                 upperPeriod = NULL,
                 n.levels = 100,
                 palette_name = "rainbow",
                 color_brewer = "grDevices",
                 useRaster = TRUE,
                 periodlab = "Period (metres)",
                 x_lab = "Depth (metres)",
                 keep_editable = FALSE,
                 dev_new = F,
                 add_lines = cbind(alb_Al_track_WR_comp[,1],
                                   alb_Al_track_WR_comp[,2]),
                 add_points = NULL,
                 add_abline_h = NULL,
                 add_abline_v = NULL,
                 add_MTM_peaks = FALSE,
                 add_data = TRUE,
                 add_avg = TRUE,
                 add_MTM = FALSE,
                 demean_mtm = TRUE,
                 detrend_mtm = TRUE,
                 padfac_mtm = 5,
                 tbw_mtm = 3,
                 plot_horizontal = TRUE
                 )
```

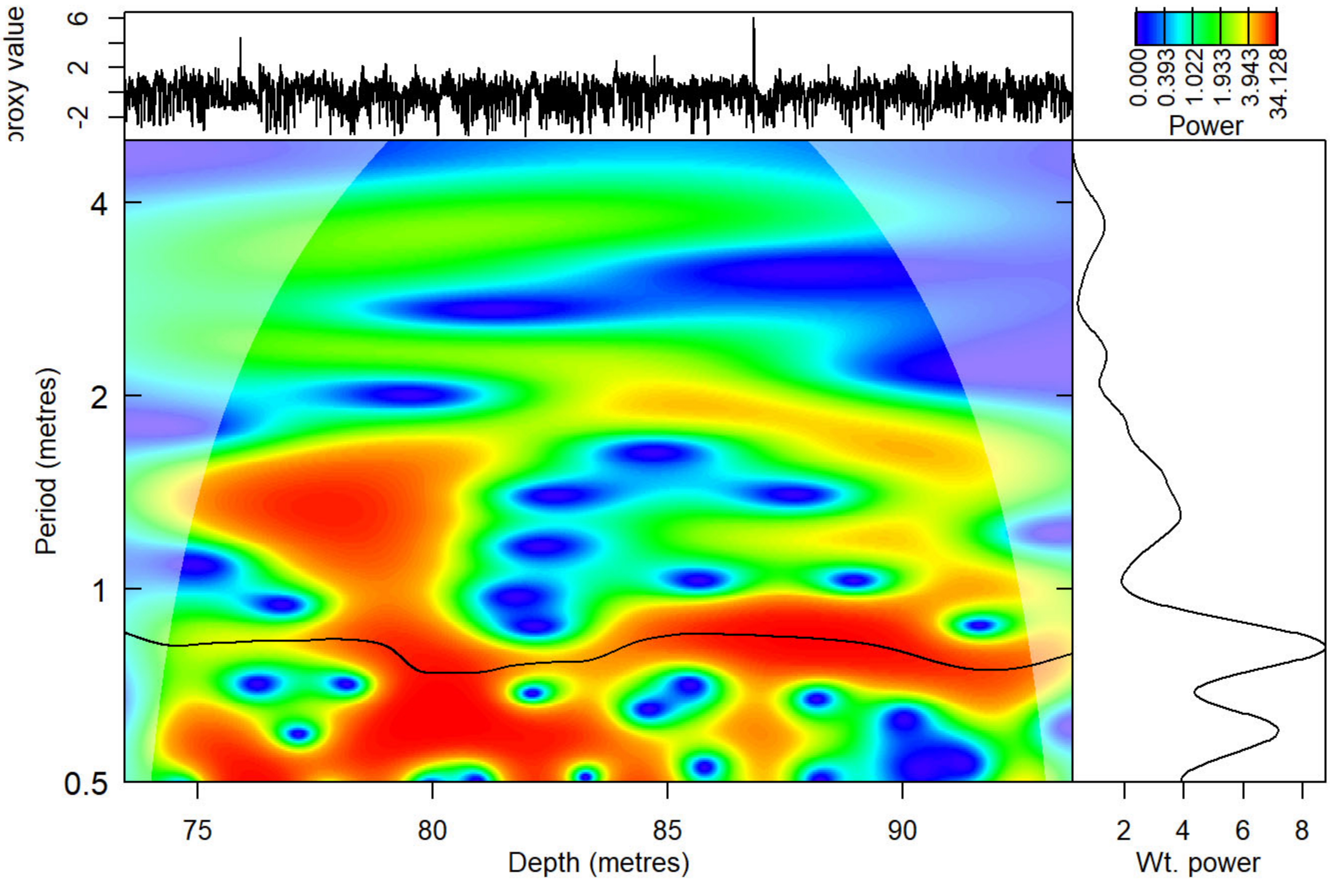


### 11.2.1.4. Potassium

```
alb_K <- cbind(Alb$DepthAdj,Alb$K)
alb_K <- na.omit(alb_K)
alb_K[!is.finite(alb_K)] <- NA
alb_K <- na.omit(alb_K)
alb_K <- iso(dat=alb_K, xmin=73.453, xmax=100)
```

```
----- ISOLATE STRATIGRAPHIC DATA BY LOCATION -----
 * Number of data points= 19311
 * Number of columns= 2
 * Minimum= 73.453 , Maximum= 93.61
 * Isolating data between 73.453 and 100
 * Number of data points following culling= 19311
```

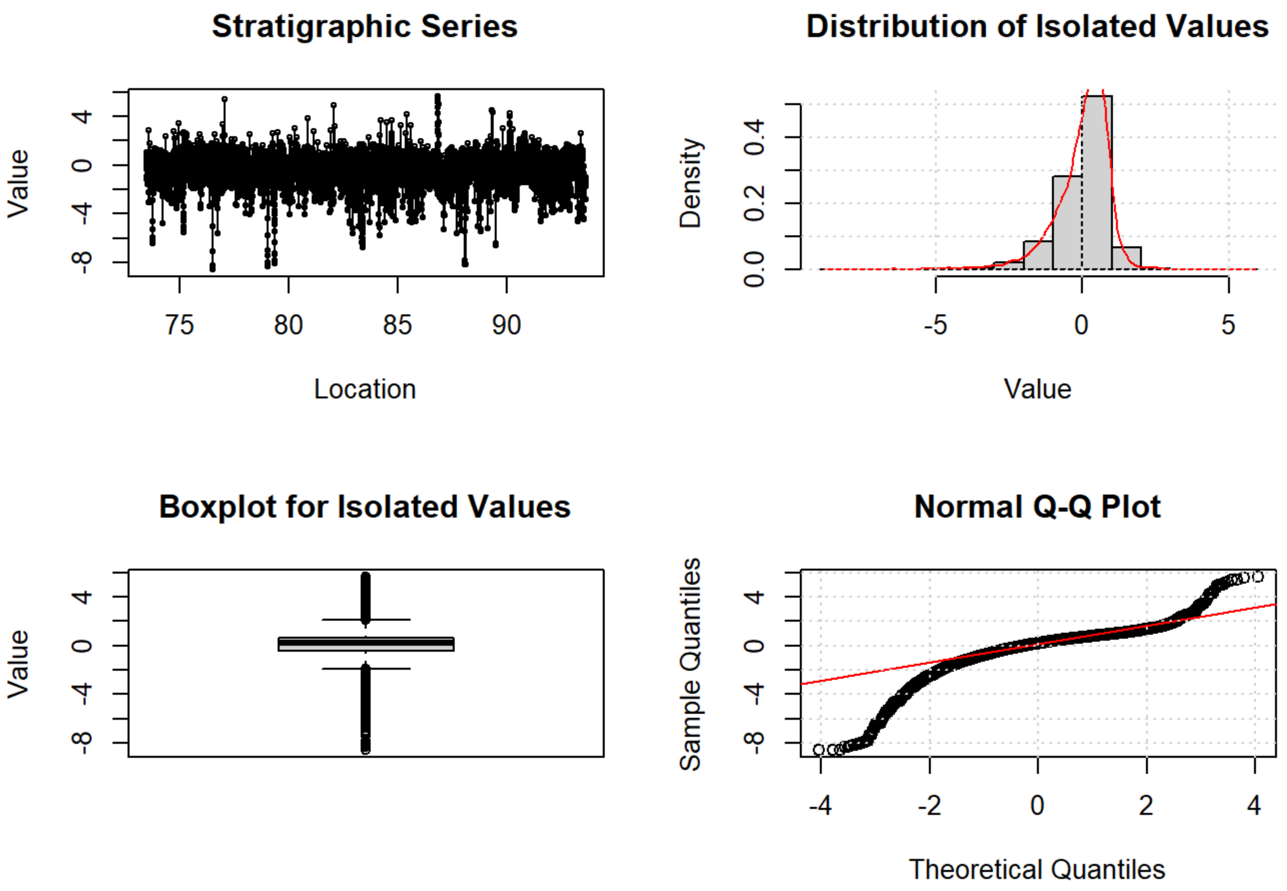


```r
alb_K_ndet <-  linterp(alb_K, dt=0.005, genplot=T)
```

```
----- APPLYING PIECEWISE-LINEAR INTERPOLATION TO STRATIGRAPHIC SERIES -----

 * Number of samples= 19311
 * New number of samples= 4032
```

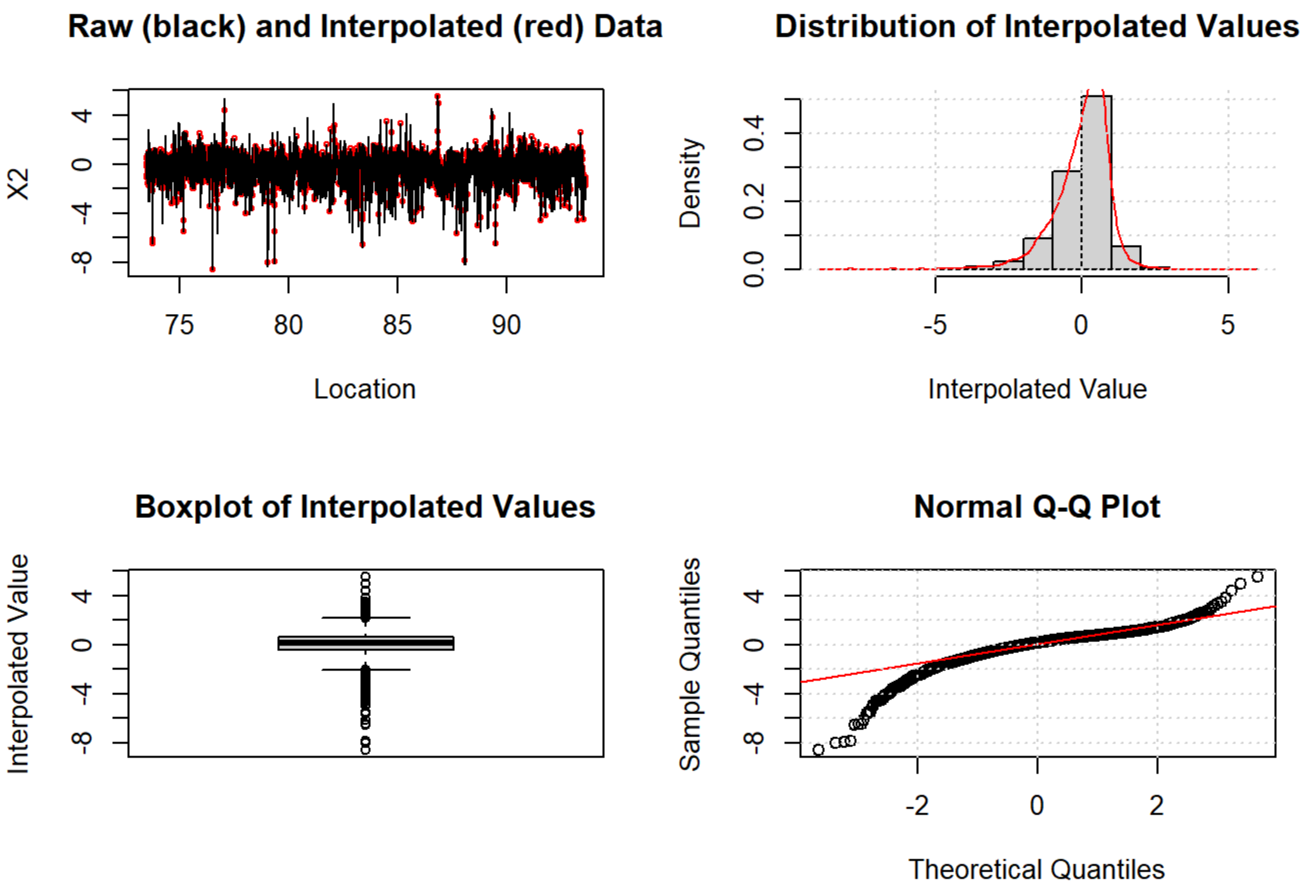


```r
#Waverider
alb_K_wt <- analyze_wavelet(alb_K_ndet,
                            dj = 1/100,
                            lowerPeriod = 0.5,
                            upperPeriod = 5,
                            verbose = FALSE,
                            omega_nr = 10
                            )

plot_wavelet(wavelet = alb_K_wt,
             lowerPeriod = NULL,
             upperPeriod = NULL,
             n.levels = 100,
             palette_name = "rainbow",
             color_brewer = "grDevices",
             useRaster = TRUE,
             periodlab = "Period (metres)",
             x_lab = "Depth (metres)",
             keep_editable = FALSE,
             dev_new = F,
             add_lines = NULL,
             add_points = NULL,
             add_abline_h = NULL,
             add_abline_v = NULL,
             add_MTM_peaks = FALSE,
             add_data = TRUE,
             add_avg = TRUE,
```

```
                add_MTM = FALSE,
                demean_mtm = TRUE,
                detrend_mtm = TRUE,
                padfac_mtm = 5,
                tbw_mtm = 3,
                plot_horizontal = TRUE
                )
```

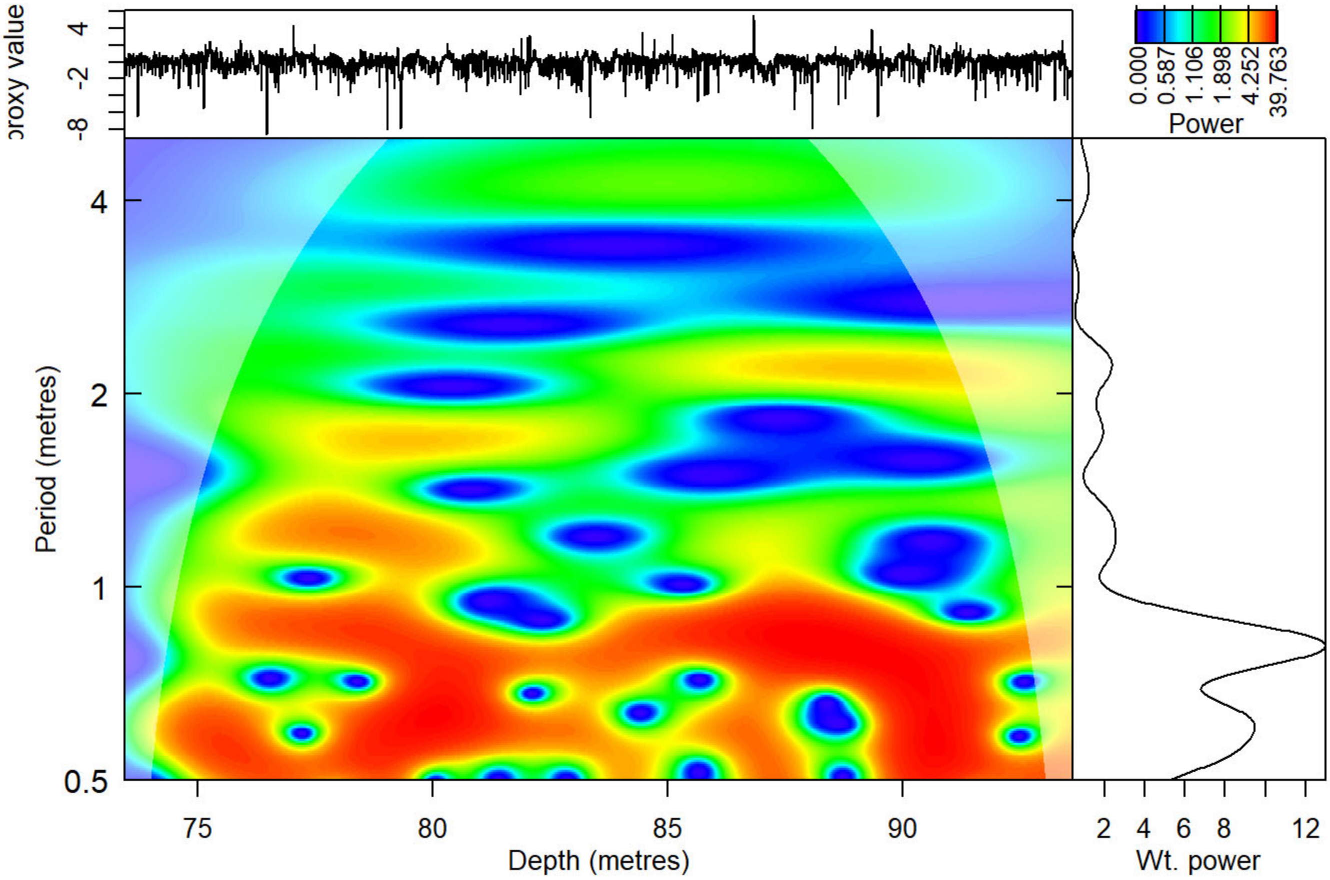


```
    #track the period (m) of the 173 kyr cycle
#alb_K_track_WR <- track_period_wavelet(astro_cycle = 173,
#                                       wavelet = alb_K_wt,
#                                       n.levels = 100,
#                                       periodlab = "Period (metres)",
#                                       x_lab = "depth (metres)",
#                                       palette_name = "rainbow",
#                                       color_brewer = "grDevices",
#                                       plot_horizontal = TRUE
#                                       )

#   alb_K_track_WR_comp <- completed_series(wavelet = alb_K_wt,
#                                           tracked_curve = alb_K_track_WR,
#                                           period_up = 1,
#                                           period_down = 0.70,
#                                           extrapolate = TRUE,
#                                           genplot = FALSE,
#                                           keep_editable = FALSE
#                                           )
```

```r
#    alb_K_track_WR_comp[alb_K_track_WR_comp[,2]<0.74,2]<-0.74

    #To keep a similar trend in the K data in the 73-80 m interval, when tracking,
    #we implemented a highpass filter for all the values above 0.875m to keep a
    #similar trend between K and the other detrital elements

#     alb_K_track_WR_comp[alb_K_track_WR_comp[,2]>0.875,2]<-0.875

#     alb_K_track_WR_comp <- loess_auto(alb_K_track_WR_comp)

#     write.csv(alb_K_track_WR_comp,"alb_K_track_WR.csv")


# Loading the tracked curve
    alb_K_track_WR_comp <- read.csv("alb_K_track_WR.csv")
    alb_K_track_WR_comp <- alb_K_track_WR_comp[,c(2,3)]

    plot_wavelet(wavelet = alb_K_wt,
                 lowerPeriod = NULL,
                 upperPeriod = NULL,
                 n.levels = 100,
                 palette_name = "rainbow",
                 color_brewer = "grDevices",
                 useRaster = TRUE,
                 periodlab = "Period (metres)",
                 x_lab = "Depth (metres)",
                 keep_editable = FALSE,
                 dev_new = F,
                 add_lines = cbind(alb_K_track_WR_comp[,1],
                                   alb_K_track_WR_comp[,2]),
                 add_points = NULL,
                 add_abline_h = NULL,
                 add_abline_v = NULL,
                 add_MTM_peaks = FALSE,
                 add_data = TRUE,
                 add_avg = TRUE,
                 add_MTM = FALSE,
                 demean_mtm = TRUE,
                 detrend_mtm = TRUE,
                 padfac_mtm = 5,
                 tbw_mtm = 3,
                 plot_horizontal = TRUE
                 )
```

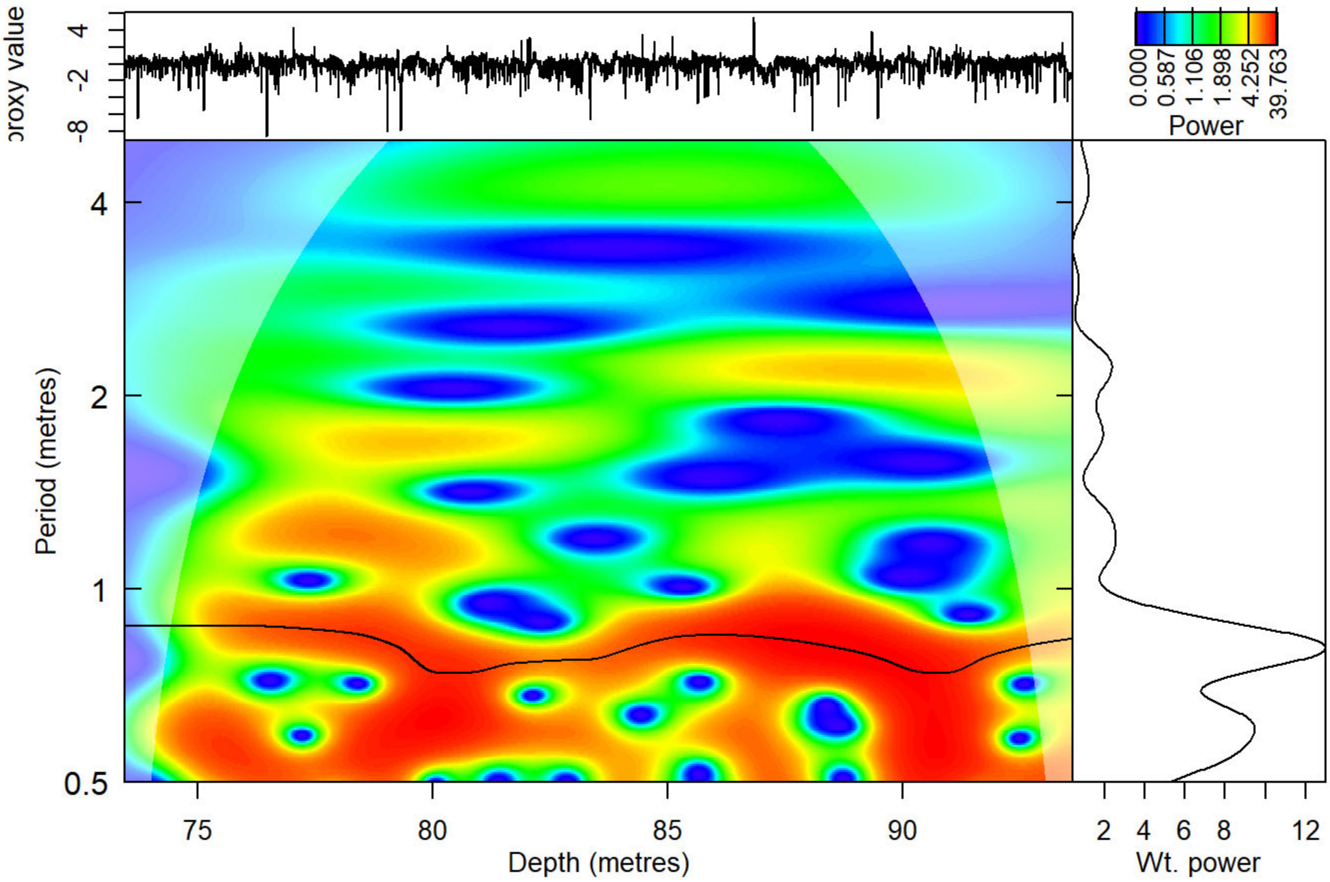


### 11.2.1.5. Depth model resulting from the 173 kyr cycle tracking of the 4 detrital elements

```
alb_Ti_track_comp1 <- read.csv("alb_Ti_track_WR.csv")
alb_Si_track_comp1 <- read.csv("alb_Si_track_WR.csv")
alb_Al_track_comp1 <- read.csv("alb_Al_track_WR.csv")
alb_K_track_comp1 <- read.csv("alb_K_track_WR.csv")

plot(alb_Ti_track_comp1[,2],
     100*(alb_Ti_track_comp1[,3]),
     type="l",col="black",
     lwd= 2,
     xlab = "Depth adjusted (m)",
     ylab = "Period (cm)",
     ylim = c(70,92)
     )

lines(alb_Si_track_comp1[,2],
      100*(alb_Si_track_comp1[,3]),
      col="red",
      lwd=2
      )

lines(alb_Al_track_comp1[,2],
      100*(alb_Al_track_comp1[,3]),
      col="green",
      lwd=2
```

```
      )

lines(alb_K_track_comp1[,2],
      100*(alb_K_track_comp1[,3]),
      col="blue",
      lwd=2
      )

track_comp_1 <- cbind(alb_Ti_track_comp1[,3],
                      alb_Si_track_comp1[,3],
                      alb_Al_track_comp1[,3],
                      alb_K_track_comp1[,3]
                      )

depth<-alb_Ti_track_comp1[,2]
curve_mean <- rowMeans(100*(track_comp_1))
curve_sd <-  rowSds(100*(track_comp_1))

  # Add polygon for mean ± SD
polygon(c(depth, rev(depth)),
        c(curve_mean + curve_sd, rev(curve_mean - curve_sd)),
        col = rgb(0.7, 0.7, 0.7, 0.4), border = NA)

  # Optionally overlay mean line
lines(depth, curve_mean, col = "yellow", lwd = 2)

legend("bottomleft",
       legend = c("Ti", "Si", "K", "Al", "Mean", "Mean ± SD"),
       col = c("black", "red", "blue", "green","yellow", rgb(0.7, 0.7, 0.7, 0.8)),
       lty = c(1, 1, 1, 1, 1,NA),
       lwd = c(2, 2, 2, 2, 2,NA),
       pch = c(NA, NA, NA, NA,NA, 15),
       pt.cex = 2,
       bty = "n",          # No box around legend
       cex = 0.75)          # Larger legend text
```

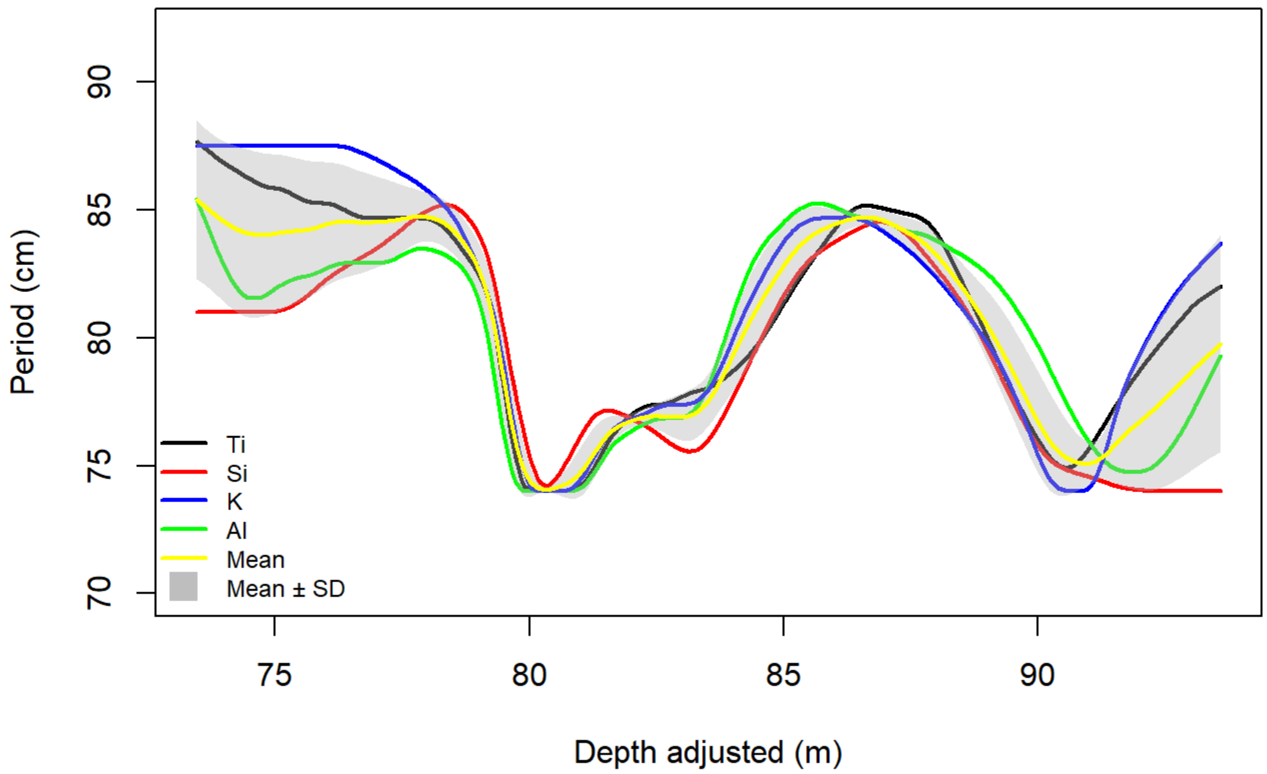


## 11.2.2. Age model

```
#Age model
track_comp_1_mean <- rowMeans(1/track_comp_1)

track_comp_1_sds <- rowSds(1/track_comp_1)

track_comp_1_plus_2Sd <- cbind(alb_Ti_track_comp1[,2],
                               1/(track_comp_1_mean+(2*track_comp_1_sds)))

track_comp_1_min_2Sd <- cbind(alb_Ti_track_comp1[,2],
                              1/(track_comp_1_mean-(2*track_comp_1_sds)))

track_comp_1_mean <- cbind(alb_Ti_track_comp1[,2],1/track_comp_1_mean)

time_track_plus_2Sd <- curve2time(track_comp_1_plus_2Sd,tracked_cycle_period = 173)

time_track_min_2Sd <- curve2time(track_comp_1_min_2Sd,tracked_cycle_period = 173)

time_mean <- curve2time(track_comp_1_mean,tracked_cycle_period = 173)

Mean_AgeModel_WR<-time_mean
Max_AgeModel_WR<-time_track_plus_2Sd
Min_AgeModel_WR<-time_track_min_2Sd

plot(Mean_AgeModel_WR,type="l", xlab="Depth adjusted (m)", ylab="Time (ka)")
lines(Max_AgeModel_WR,col="green")
```

```
lines(Min_AgeModel_WR,col="blue")

 legend("bottomright",
       legend = c("Maximum age (+2 SD)","Mean age", "Minimum age (-2 SD)"),
       col = c("green","black","blue"),
       lty = c(1, 1, 1),
       lwd = c(2, 2, 2),
       pch = c(NA, NA, NA),
       pt.cex = 2,
       bty = "n",          # No box around legend
       cex = 1)          # Larger legend text
```

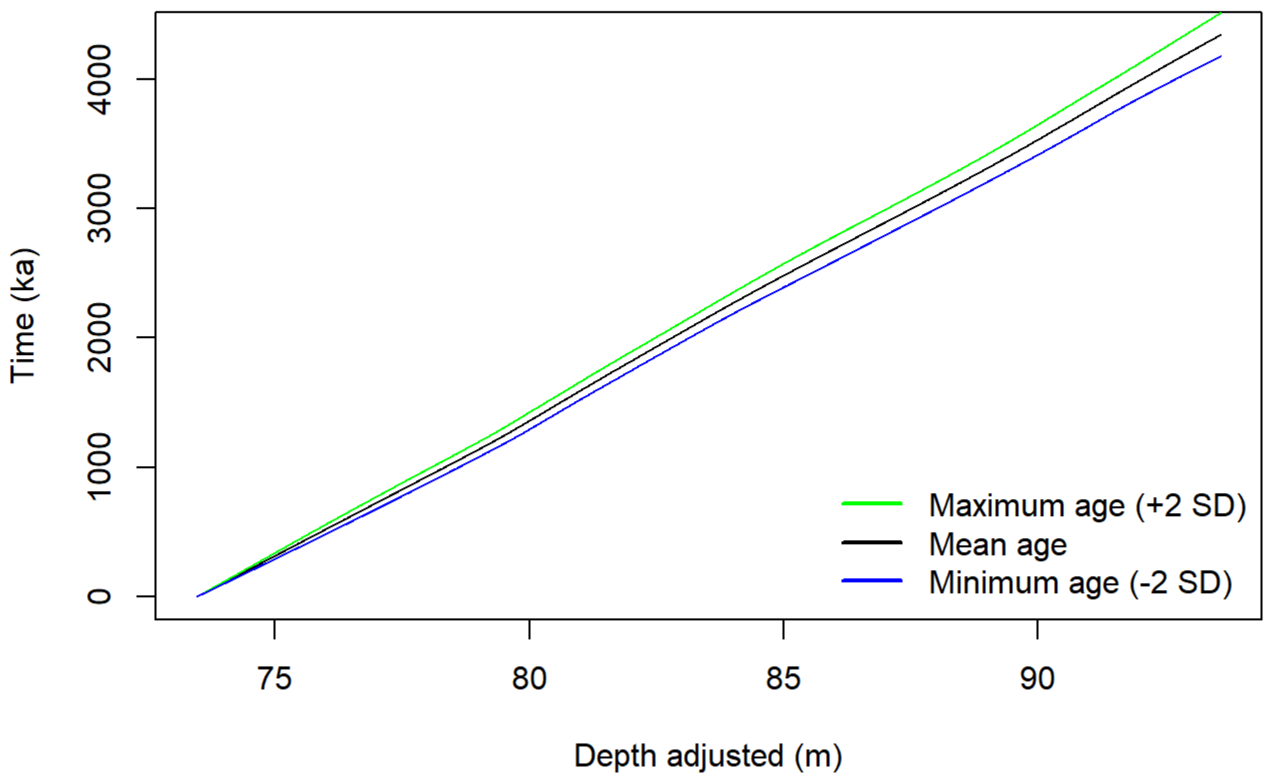


```
Age_Model_WR <- cbind(Mean_AgeModel_WR,Max_AgeModel_WR[,2],Min_AgeModel_WR[,2])
colnames(Age_Model_WR)<- cbind("Adjusted depth (m)",
                               "Mean age (ka)",
                               "Max age (ka)",
                               "Min age (ka)"
                               )
```

```
#Interpolation every 1mm of the Age-depth model to have a direct comparison
#between the age model and the original sampling rate of 1mm
Mean_Age_Model_WR_1mm <- linterp(Mean_AgeModel_WR, dt=0.001, genplot=T)
```

```
----- APPLYING PIECEWISE-LINEAR INTERPOLATION TO STRATIGRAPHIC SERIES -----
```

```
 * Number of samples= 4032
 * New number of samples= 20155
```

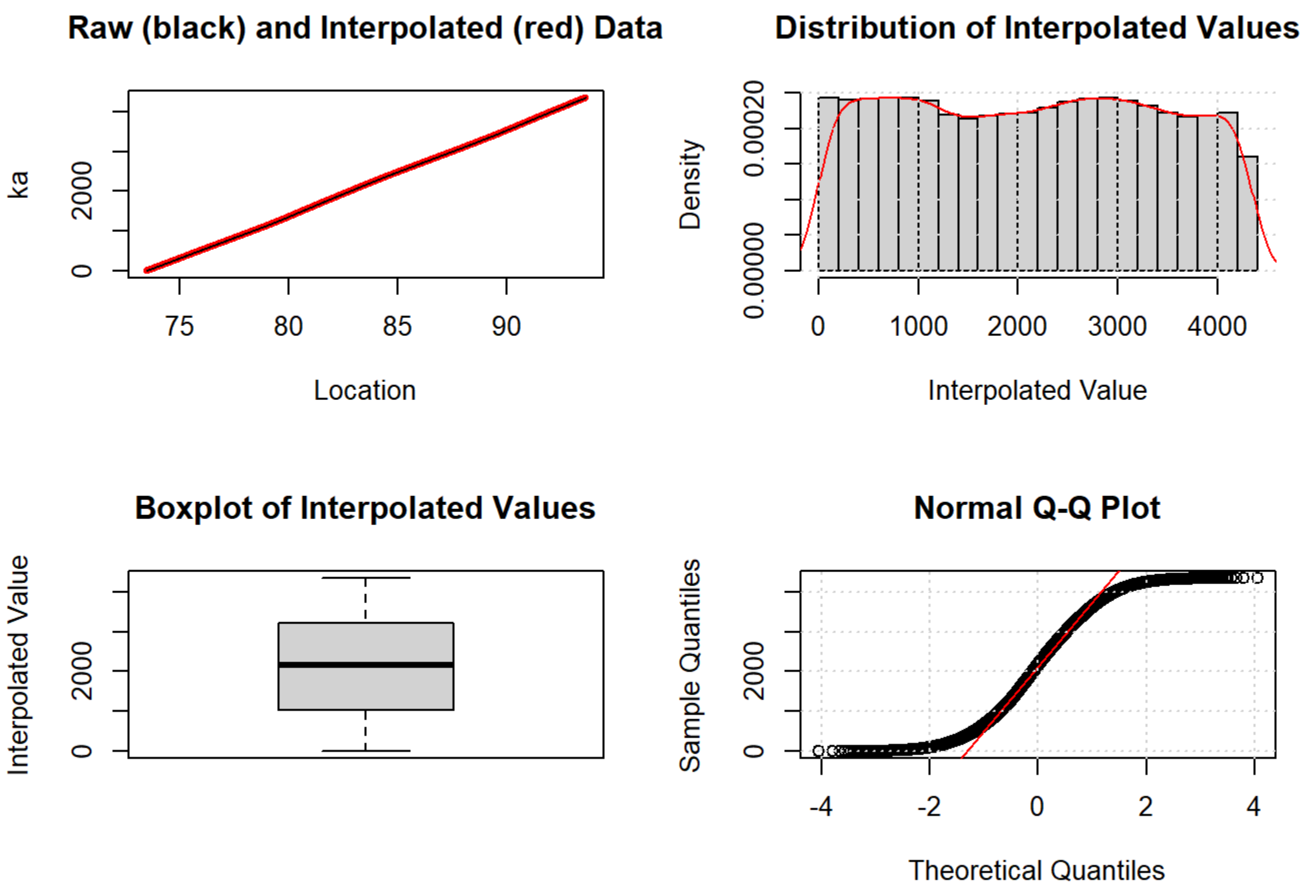


```
Max_Age_Model_WR_1mm <- linterp(Max_AgeModel_WR, dt=0.001, genplot=T)
```

```
----- APPLYING PIECEWISE-LINEAR INTERPOLATION TO STRATIGRAPHIC SERIES -----

 * Number of samples= 4032
 * New number of samples= 20155
```

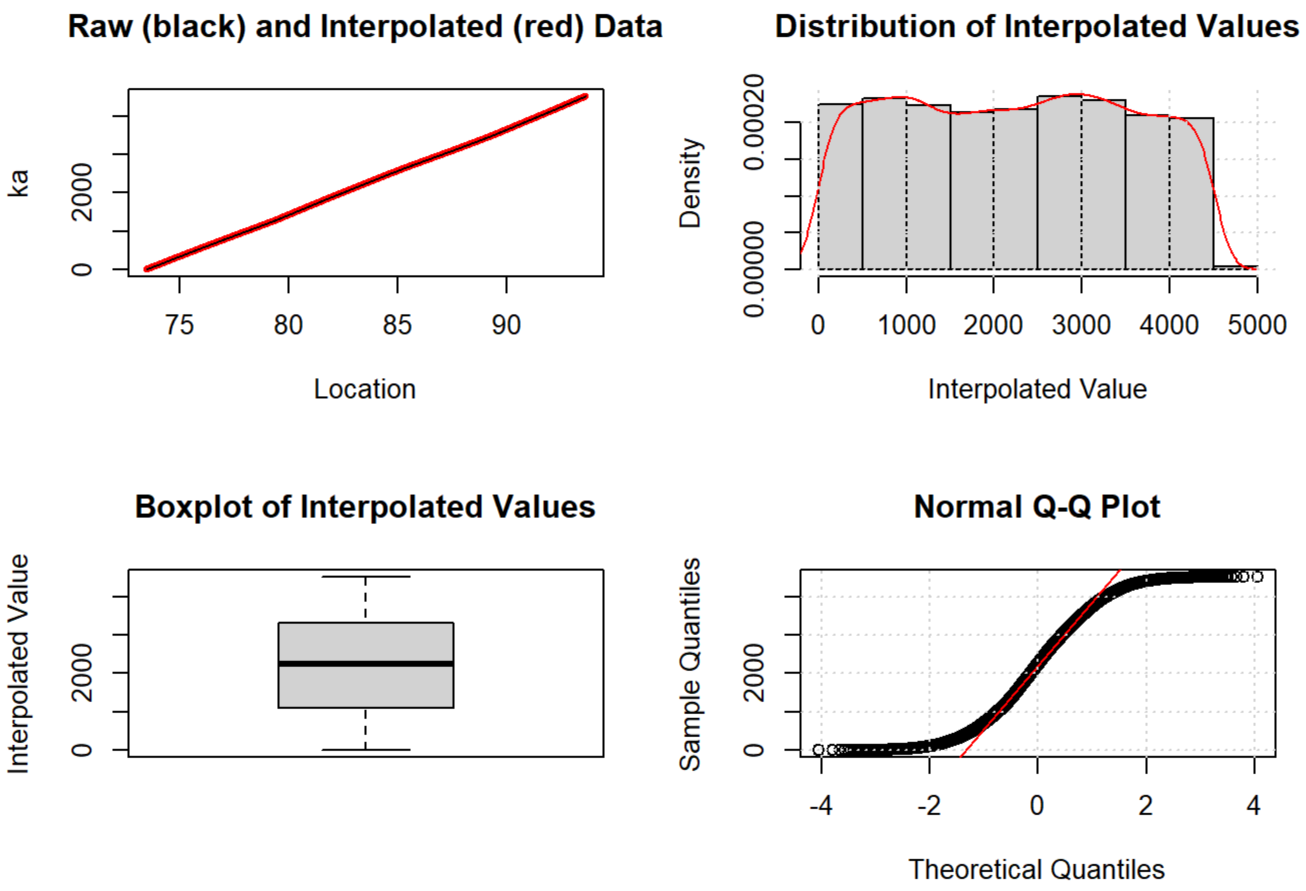


```
Min_Age_Model_WR_1mm <- linterp(Min_AgeModel_WR, dt=0.001, genplot=T)
```

```
----- APPLYING PIECEWISE-LINEAR INTERPOLATION TO STRATIGRAPHIC SERIES -----

 * Number of samples= 4032
 * New number of samples= 20155
```

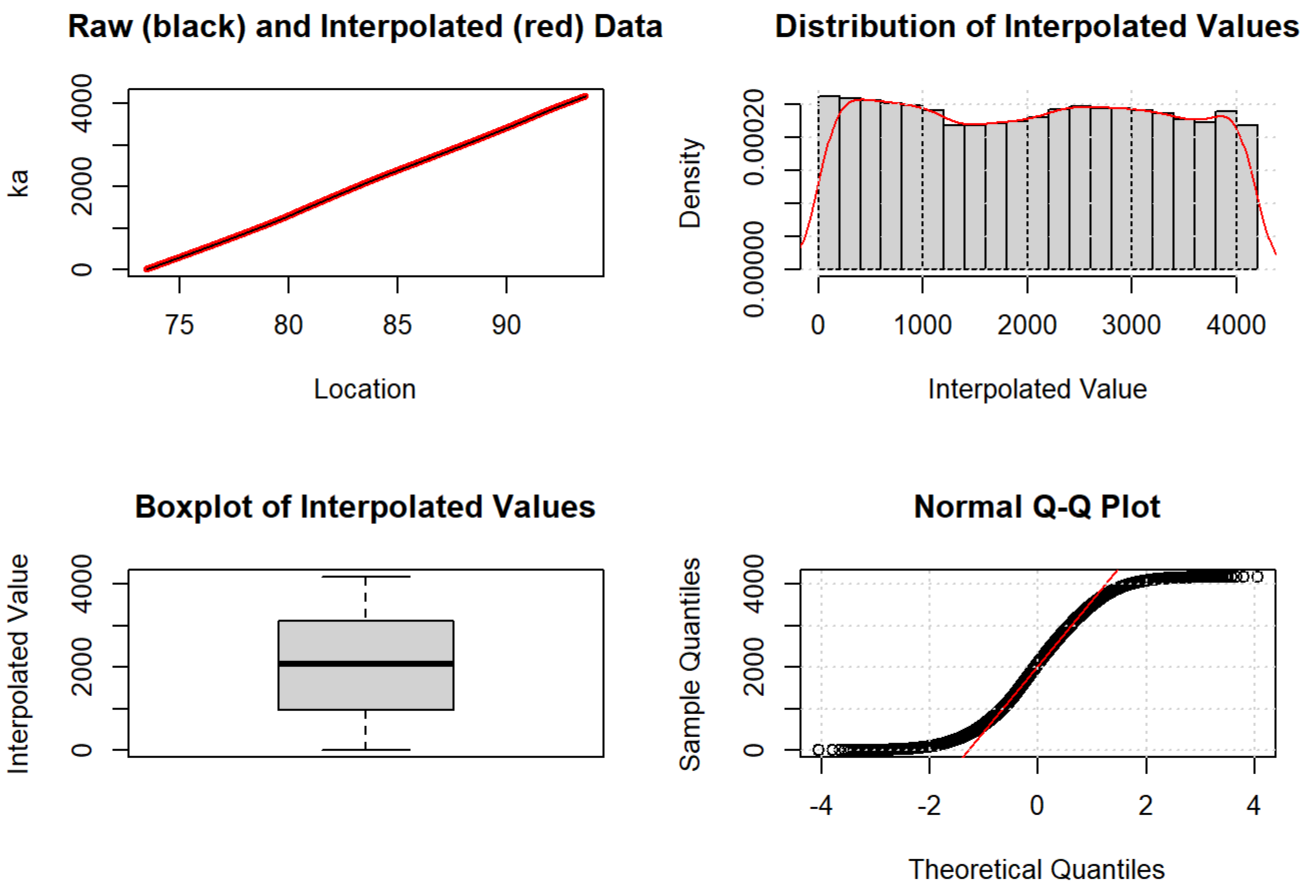


```
  Age_Model_WR_1mm <- cbind(Mean_Age_Model_WR_1mm,
                            Max_Age_Model_WR_1mm[,2],
                            Min_Age_Model_WR_1mm[,2]
                            )

  colnames(Age_Model_WR)<- cbind("Adjusted depth (m)",
                                 "Mean age (ka)",
                                 "Max age (ka)",
                                 "Min age (ka)"
                                 )

  #Saving of the age model as .csv files
#  write.csv(Age_Model_WR_1mm,"AgeModel_WR_rsp1mm.csv")
```

## 11.2.3. Sedimentation rate

```
  plot(alb_Ti_track_comp1[,2],
       (100*(alb_Ti_track_comp1[,3]))/173,
       type="l",
       col="black",
       lwd= 2,
       xlab = "Depth adjusted (m)",
       ylab = "Sed rate (cm/kyr)",
       ylim = c(0.4,0.55)
       )
```

```r
lines(alb_Si_track_comp1[,2],
      (100*(alb_Si_track_comp1[,3]))/173,
      col="red",
      lwd=2
      )

lines(alb_Al_track_comp1[,2],
      (100*(alb_Al_track_comp1[,3]))/173,
      col="green",
      lwd=2
      )

lines(alb_K_track_comp1[,2],
      (100*(alb_K_track_comp1[,3]))/173,
      col="blue",
      lwd=2
      )

lines(alb_K_track_comp1[,2],
      (alb_K_track_comp1[,3])-(alb_K_track_comp1[,3])+0.493,
      col="red",
      lwd=2,
      lty=3
      )

legend("bottomleft",
       legend = c("Ti", "Si", "K", "Al", "ASM = 0.493 cm/kyr (theoretical)" ),
       col = c("black", "red", "blue", "green","red"),
       lty = c(1, 1, 1, 1, 3),
       lwd = c(2, 2, 2, 2, 2),
       pch = c(NA, NA, NA, NA),
       pt.cex = 2,
       bty = "n",         # No box around legend
       cex = 0.75)         # Larger legend text
```

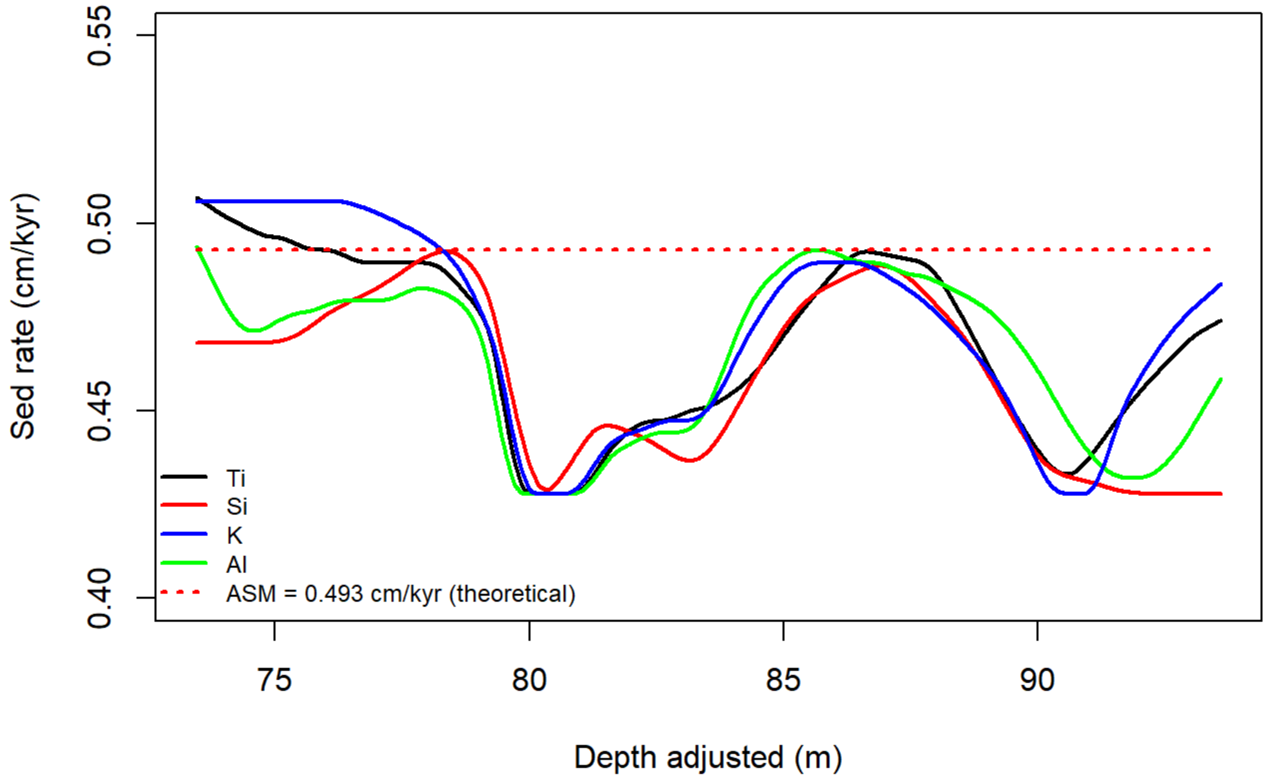


---

# Step 12: Astronomical Time Scale

This step is performed in Microsoft Excel. The ATS is presented in Supplementary Materials 6.

The built age-model data are set along the composite ACL-ACU core at the corresponding adjusted depth (from 73.452 to 93.609 m).

The data from 73.452 to 73.72 are overlapping with the one of Zhao et al (2022b).

From 62.111 m to 73.72 m the absolute age of Zhao et al (2022b) are used. The absolute age of 499.9115 ± 0.9 Ma located at 73.72 m is used to anchor both ACU and ACL in time domain.

From 73.721 to 93.609 m, the time data used are from our newly built age-model.

---

# Step 13: Milankovitch filters of the 1 kyr resampled Miaolingian composite dataset

## 13.1. Creation of a ".csv" file

This step is conducted in Microsoft Excel.

The created file contains the Adjusted depth, the Time in kyr and the Ti Z-score values as follows:

AdjDepth; Time; Ti

62.111; 497083.4; 1.57549322

62.112; 497083.6; 1.10323208

62.113; 497083.7; 0.63097093

…; …; …

93.609; 504154.084; -0.5940352

93.610; 504154.296; -2.3646753

I called this file alb_Ti_Mia_WR.csv as this includes all the Ti data for the entire Miaolingian Epoch recorded in the core tuned using the WaverideR age-depth model.

## 13.2. Reading of the file in R and resampling every 1 kyr of the time interval

```
alb_Ti_Mia <- read.csv("alb_Ti_Mia_WR.csv",sep=";")
alb_Ti_Mia <- cbind(alb_Ti_Mia$Time,alb_Ti_Mia$Ti)
alb_Ti_Mia <- na.omit(alb_Ti_Mia)
alb_Ti_Mia[!is.finite(alb_Ti_Mia)] <- NA
alb_Ti_Mia <- na.omit(alb_Ti_Mia)

MIA<- iso(dat=alb_Ti_Mia, xmin=497000, xmax=505000)
```

```
----- ISOLATE STRATIGRAPHIC DATA BY LOCATION -----
 * Number of data points= 30577
 * Number of columns= 2
 * Minimum= 497083.4 , Maximum= 504197.4
 * Isolating data between 497000 and 505000
 * Number of data points following culling= 30577
```

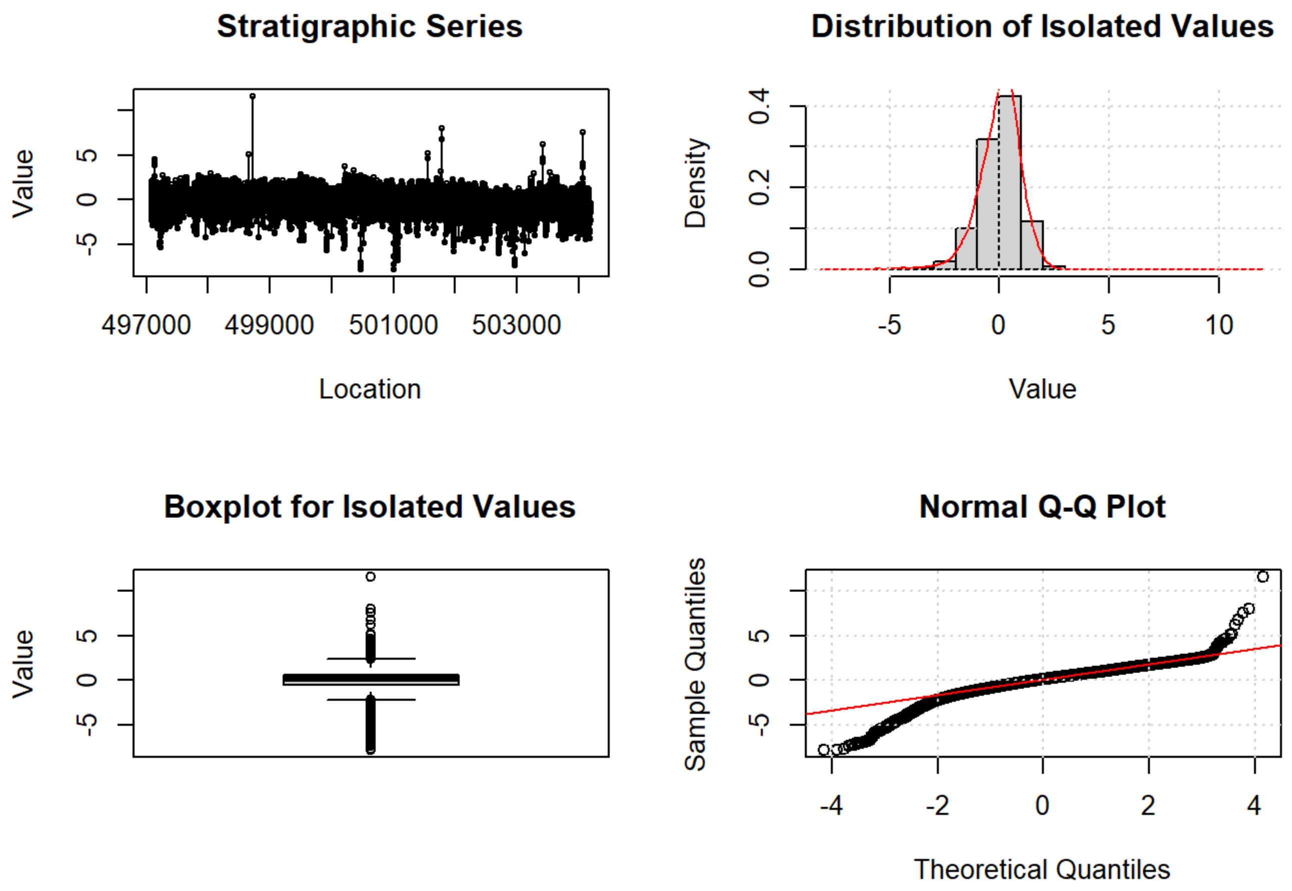


```
MIA <- linterp(MIA,dt=1, genplot=T)
```

```
----- APPLYING PIECEWISE-LINEAR INTERPOLATION TO STRATIGRAPHIC SERIES -----

 * Number of samples= 30577
 * New number of samples= 7115
```

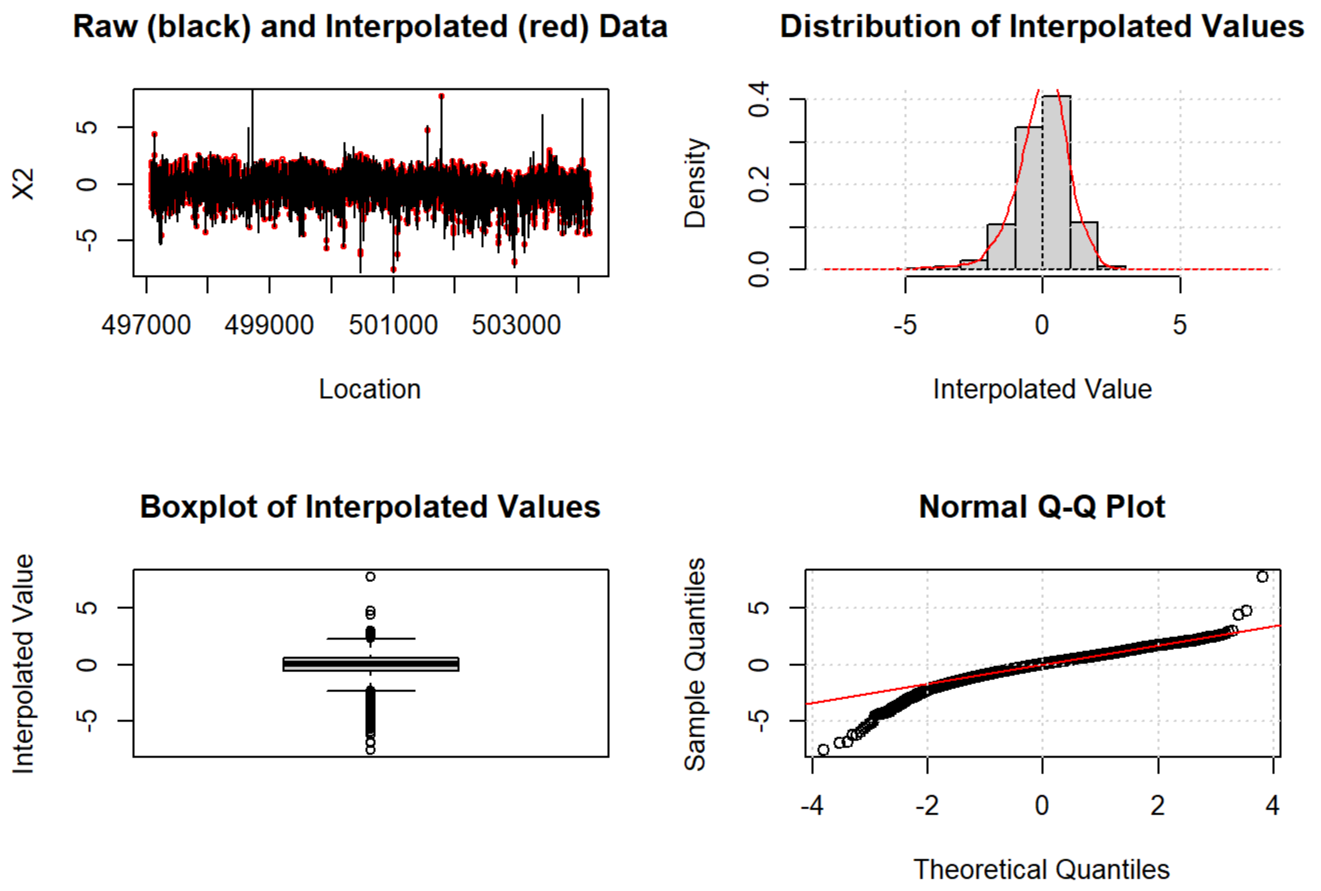


```
MIA_Ti_tuned <- linterp(MIA, genplot=T)
```

```
----- APPLYING PIECEWISE-LINEAR INTERPOLATION TO STRATIGRAPHIC SERIES -----

 * Number of samples= 7115
 * Determining median sampling interval for series
 * Will interpolate to median sampling interval of 1
 * New number of samples= 7115
```

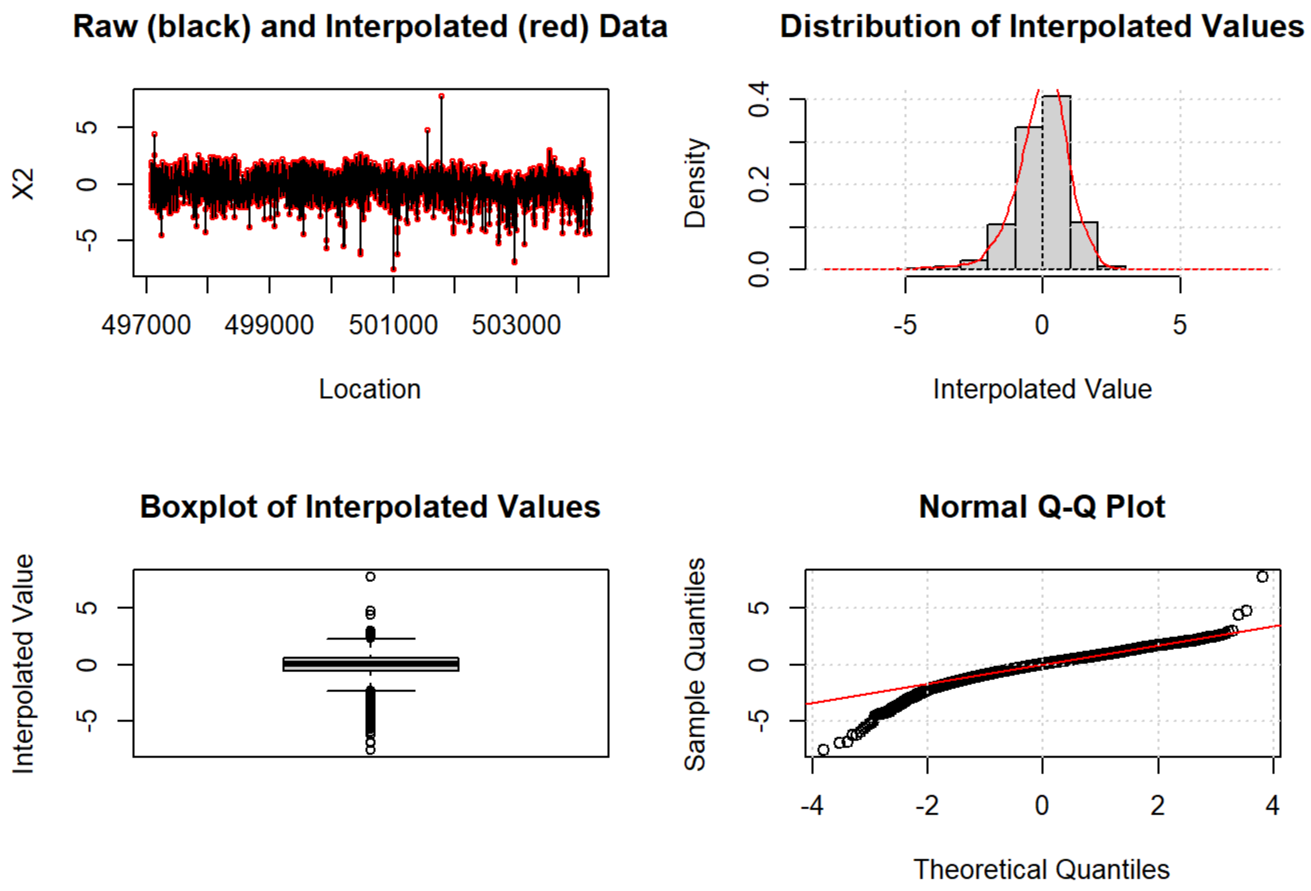


## 13.3. Resampling every 1 kyr and Milankovitch filters

```
#Resampling every 1 kyr
MIA<- iso(dat=alb_Ti_Mia, xmin=497000, xmax=505000)
```

```
----- ISOLATE STRATIGRAPHIC DATA BY LOCATION -----
 * Number of data points= 30577
 * Number of columns= 2
 * Minimum= 497083.4 , Maximum= 504197.4
 * Isolating data between 497000 and 505000
 * Number of data points following culling= 30577
```

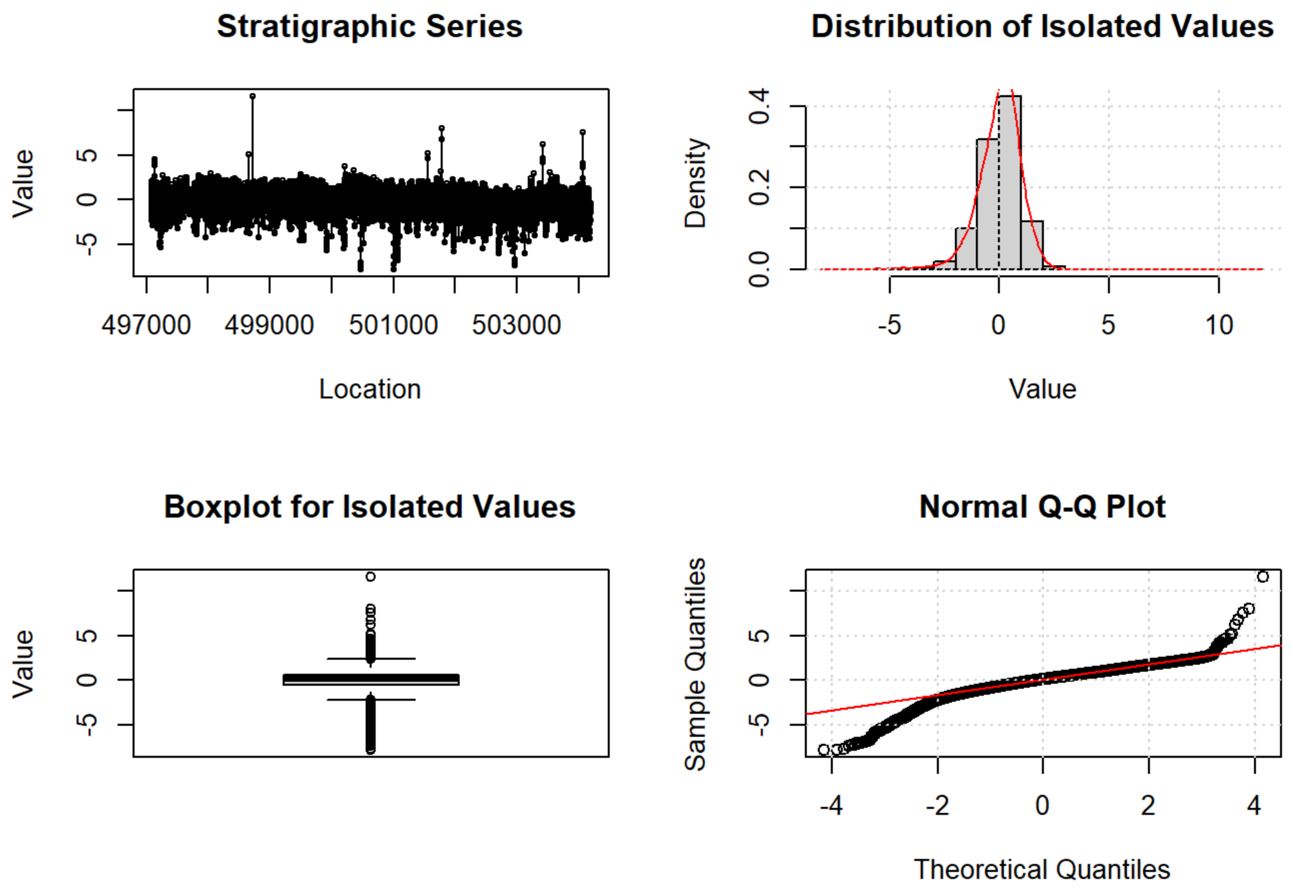


```
MIA <- linterp(MIA,dt=1, genplot=T)
```

```
----- APPLYING PIECEWISE-LINEAR INTERPOLATION TO STRATIGRAPHIC SERIES -----

 * Number of samples= 30577
 * New number of samples= 7115
```

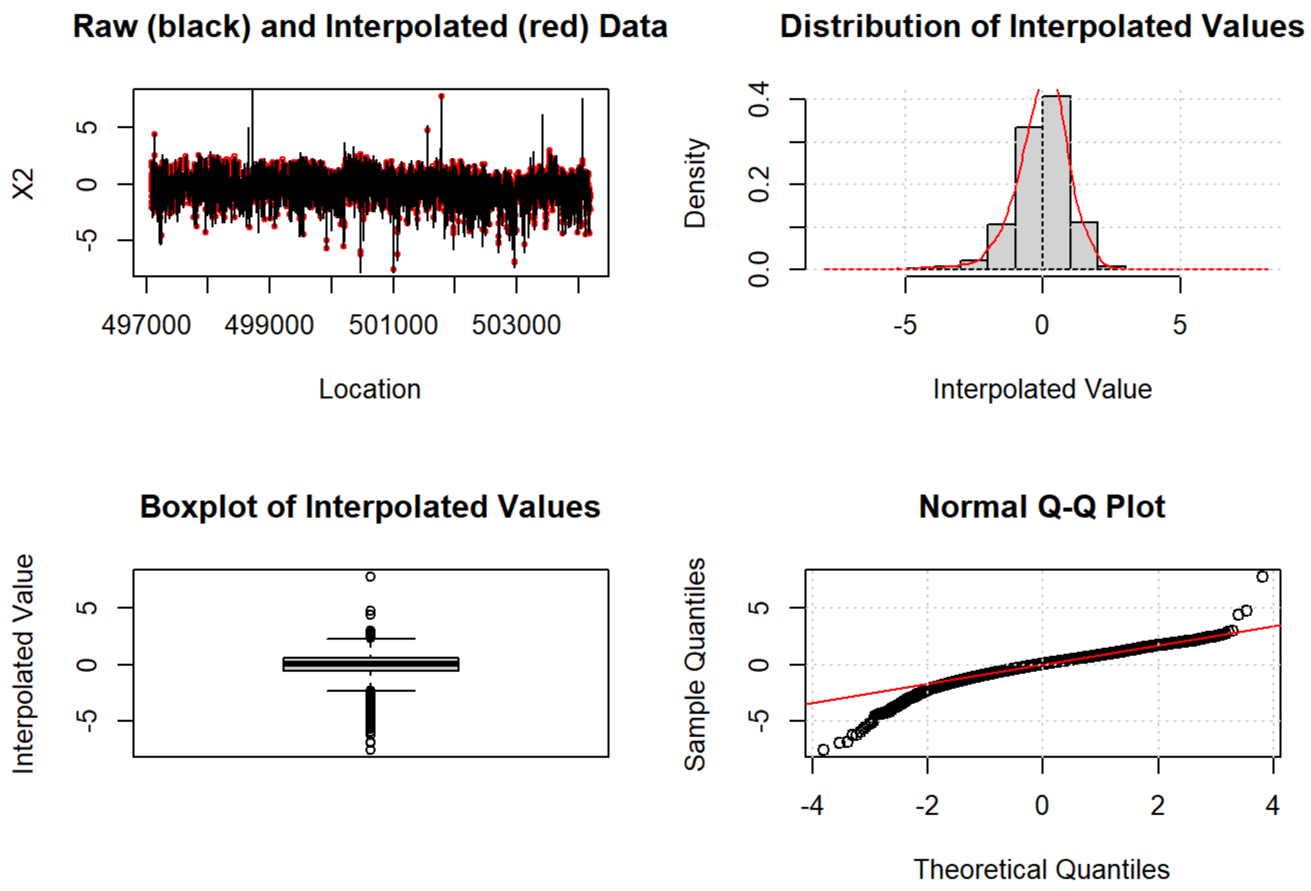


```
MIA_Ti_tuned <- linterp(MIA, genplot=T)
```

```
----- APPLYING PIECEWISE-LINEAR INTERPOLATION TO STRATIGRAPHIC SERIES -----

 * Number of samples= 7115
 * Determining median sampling interval for series
 * Will interpolate to median sampling interval of 1
 * New number of samples= 7115
```

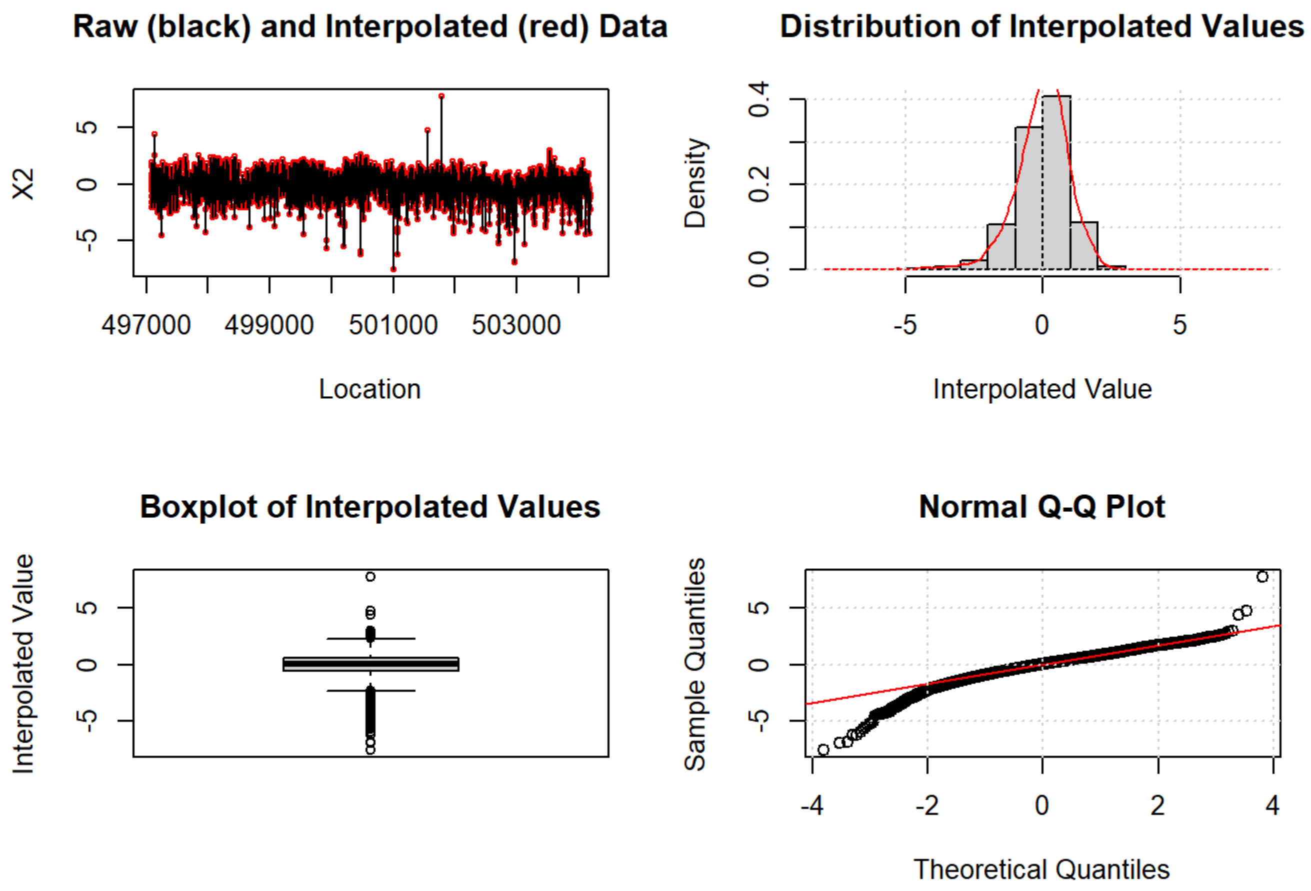


```
#Filter out the long eccentricity cycle
MIA_Ti_1200 <- taner(MIA_Ti_tuned,xmax=0.01, fhigh=1/1150, flow=1/1300, demean = T)
```

```
----- TANER BANDPASS FILTERING STRATIGRAPHIC SERIES-----
 * Number of data points= 7115
 * Sample interval= 1
 * Mean value removed= -0.03173093
```

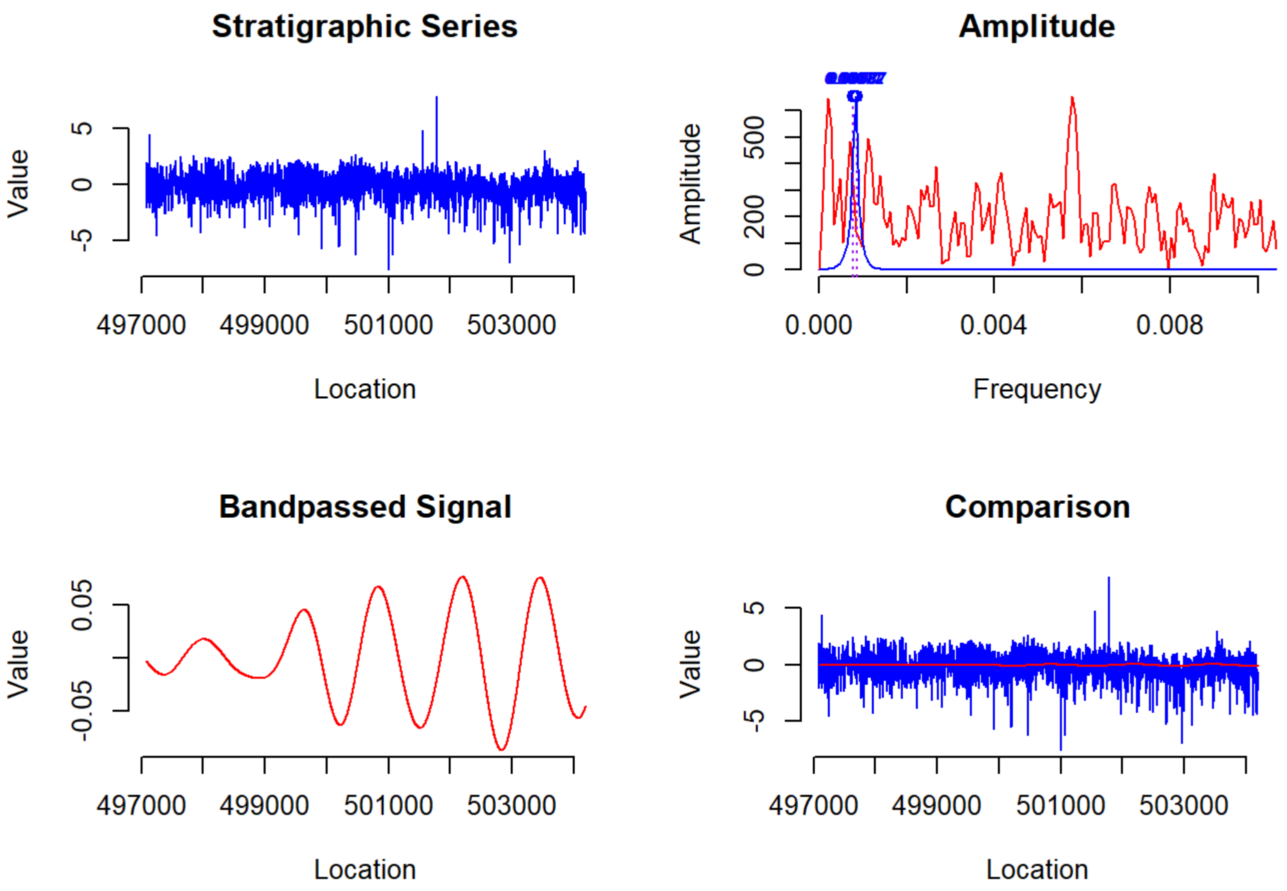


```
#Filter out the long eccentricity cycle
MIA_Ti_405 <- taner(MIA_Ti_tuned,xmax=0.01, fhigh=1/450, flow=1/350, demean = T)
```

```
----- TANER BANDPASS FILTERING STRATIGRAPHIC SERIES-----
 * Number of data points= 7115
 * Sample interval= 1
 * Mean value removed= -0.03173093
```

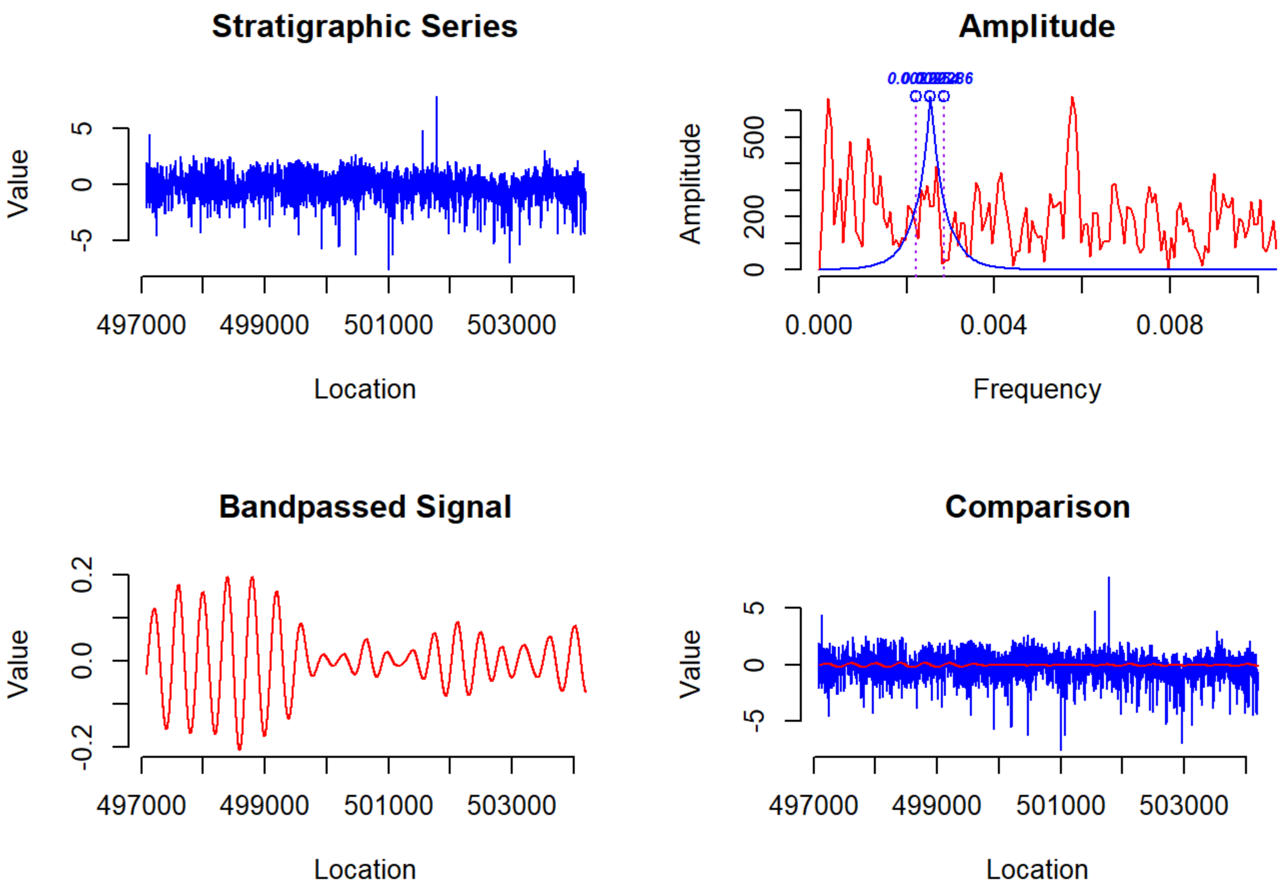


```
#Filter out the 173-kyr obliquity cycle
MIA_Ti_173 <- taner(MIA_Ti_tuned, xmax=0.01, fhigh=1/193, flow=1/150, demean=T)
```

```
----- TANER BANDPASS FILTERING STRATIGRAPHIC SERIES-----
 * Number of data points= 7115
 * Sample interval= 1
 * Mean value removed= -0.03173093
```

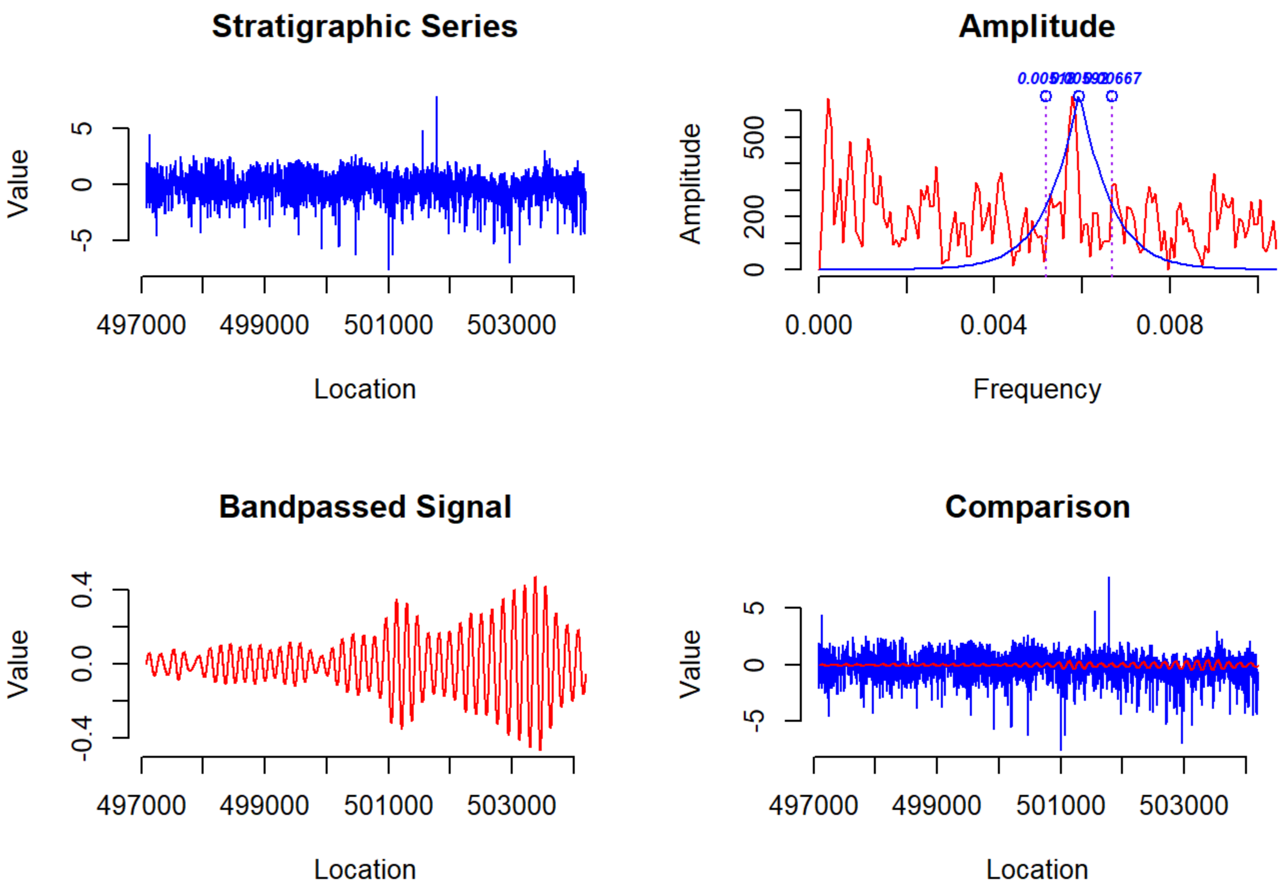


```
#Filter out the short eccentricity cycle
MIA_Ti_100 <- taner(MIA_Ti_tuned, xmax=0.02, fhigh=1/85, flow=1/140, demean = T)
```

```
----- TANER BANDPASS FILTERING STRATIGRAPHIC SERIES-----
 * Number of data points= 7115
 * Sample interval= 1
 * Mean value removed= -0.03173093
```

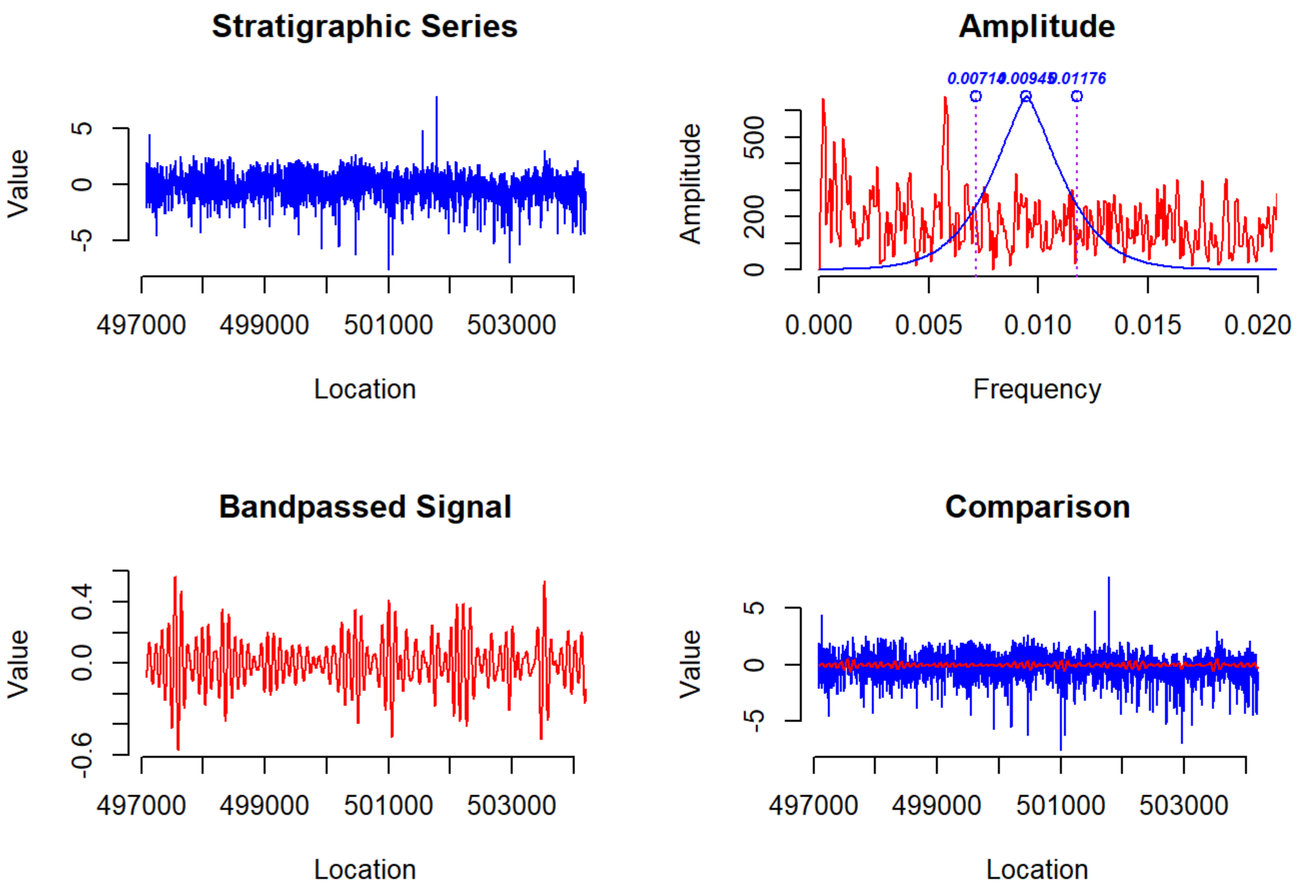


```r
#Filter out the entire obliquity band
MIA_Ti_31_all <- taner(MIA_Ti_tuned, xmax=0.05, fhigh=1/24, flow=1/40, demean=T)
```

```
----- TANER BANDPASS FILTERING STRATIGRAPHIC SERIES-----
 * Number of data points= 7115
 * Sample interval= 1
 * Mean value removed= -0.03173093
```

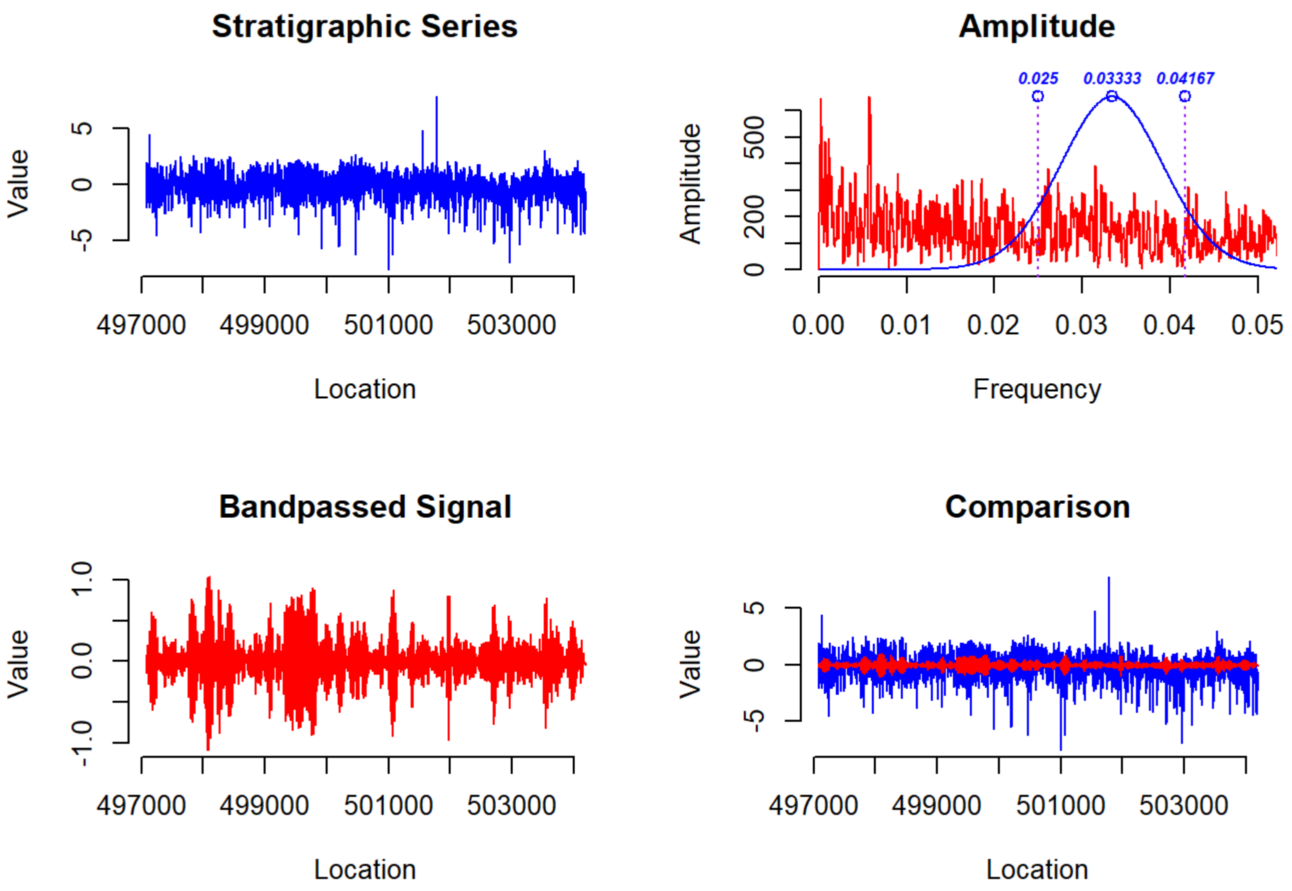


```
#Filter out the obliquity cycle that generate the 173 kyr modulation
MIA_Ti_31 <- taner(MIA_Ti_tuned, xmax=0.05, fhigh=1/27, flow=1/40, demean=T)
```

```
----- TANER BANDPASS FILTERING STRATIGRAPHIC SERIES-----
 * Number of data points= 7115
 * Sample interval= 1
 * Mean value removed= -0.03173093
```

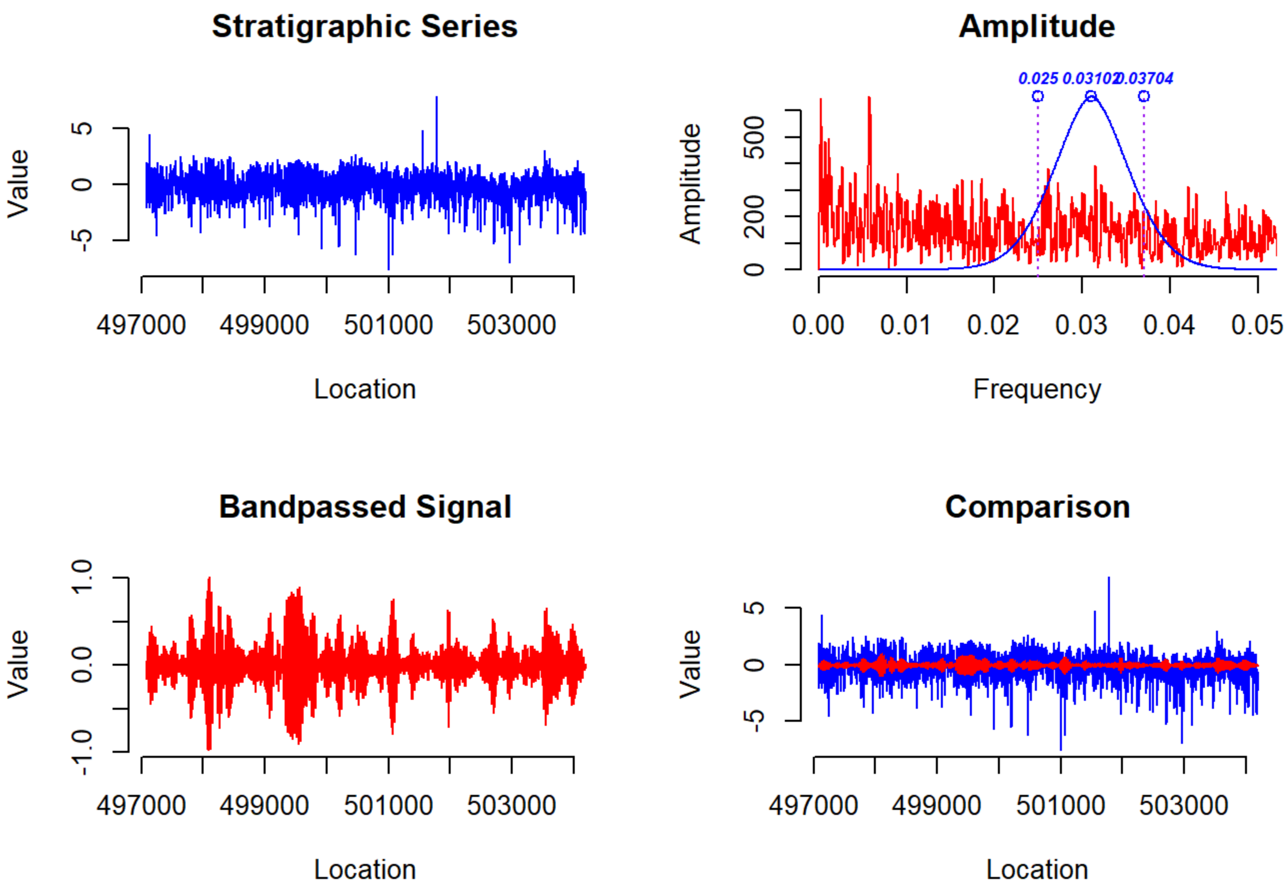


```r
#Filter out the precession cycle
MIA_Ti_20 <- taner(MIA_Ti_tuned,xmax=0.07, fhigh=1/15, flow=1/21, demean=T)
```

```
----- TANER BANDPASS FILTERING STRATIGRAPHIC SERIES-----
 * Number of data points= 7115
 * Sample interval= 1
 * Mean value removed= -0.03173093
```

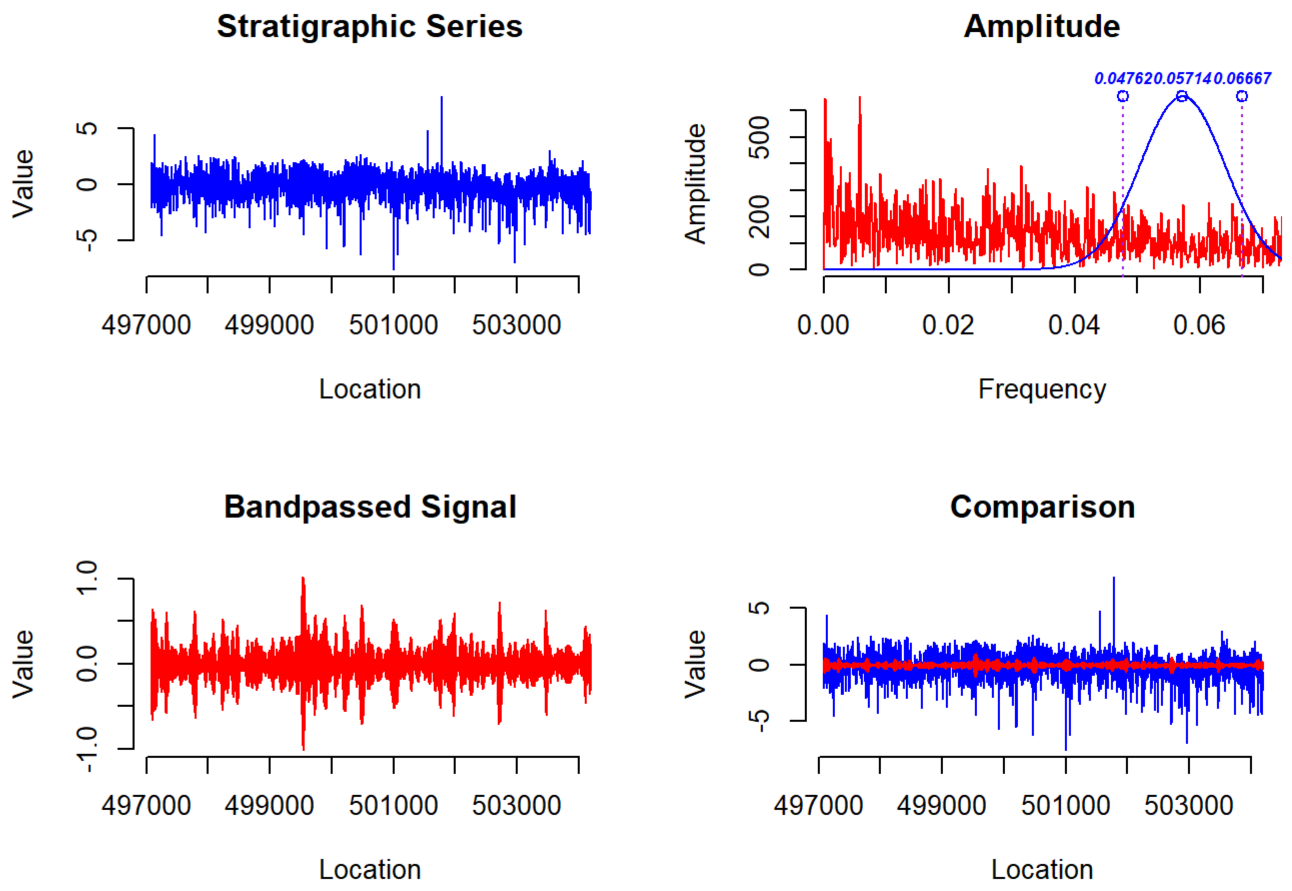


```
#Saving of the filtered Milankovitch periodicities
#write.csv(MIA_Ti_tuned,"MIA_WR_rsp_1kyr.csv")
#write.csv(MIA_Ti_1200,"MIA_WR_1200_rsp_1kyr.csv")
#write.csv(MIA_Ti_405,"MIA_WR_405_rsp_1kyr.csv")
#write.csv(MIA_Ti_173,"MIA_WR_173_rsp_1kyr.csv")
#write.csv(MIA_Ti_100,"MIA_WR_100_rsp_1kyr.csv")
#write.csv(MIA_Ti_31,"MIA_WR_31_rsp_1kyr.csv")
#write.csv(MIA_Ti_31_all,"MIA_WR_31_all_rsp_1kyr.csv")
#write.csv(MIA_Ti_20,"MIA_WR_20_rsp_1kyr.csv")
```

---

# Step 14: Lag-1 autcorrelation of the Miaolingian Series

## 14.1. Resampling of the dataset every 5 kyr

```
alb_Ti_Mia <- read.csv("alb_Ti_Mia_WR.csv",sep=";")
alb_Ti_Mia <- cbind(alb_Ti_Mia$Time,alb_Ti_Mia$Ti)
alb_Ti_Mia <- na.omit(alb_Ti_Mia)
alb_Ti_Mia[!is.finite(alb_Ti_Mia)] <- NA
alb_Ti_Mia <- na.omit(alb_Ti_Mia)

MIA<- iso(dat=alb_Ti_Mia, xmin=497000, xmax=505000)
```

```
----- ISOLATE STRATIGRAPHIC DATA BY LOCATION -----
 * Number of data points= 30577
 * Number of columns= 2
 * Minimum= 497083.4 , Maximum= 504197.4
 * Isolating data between 497000 and 505000
 * Number of data points following culling= 30577
```

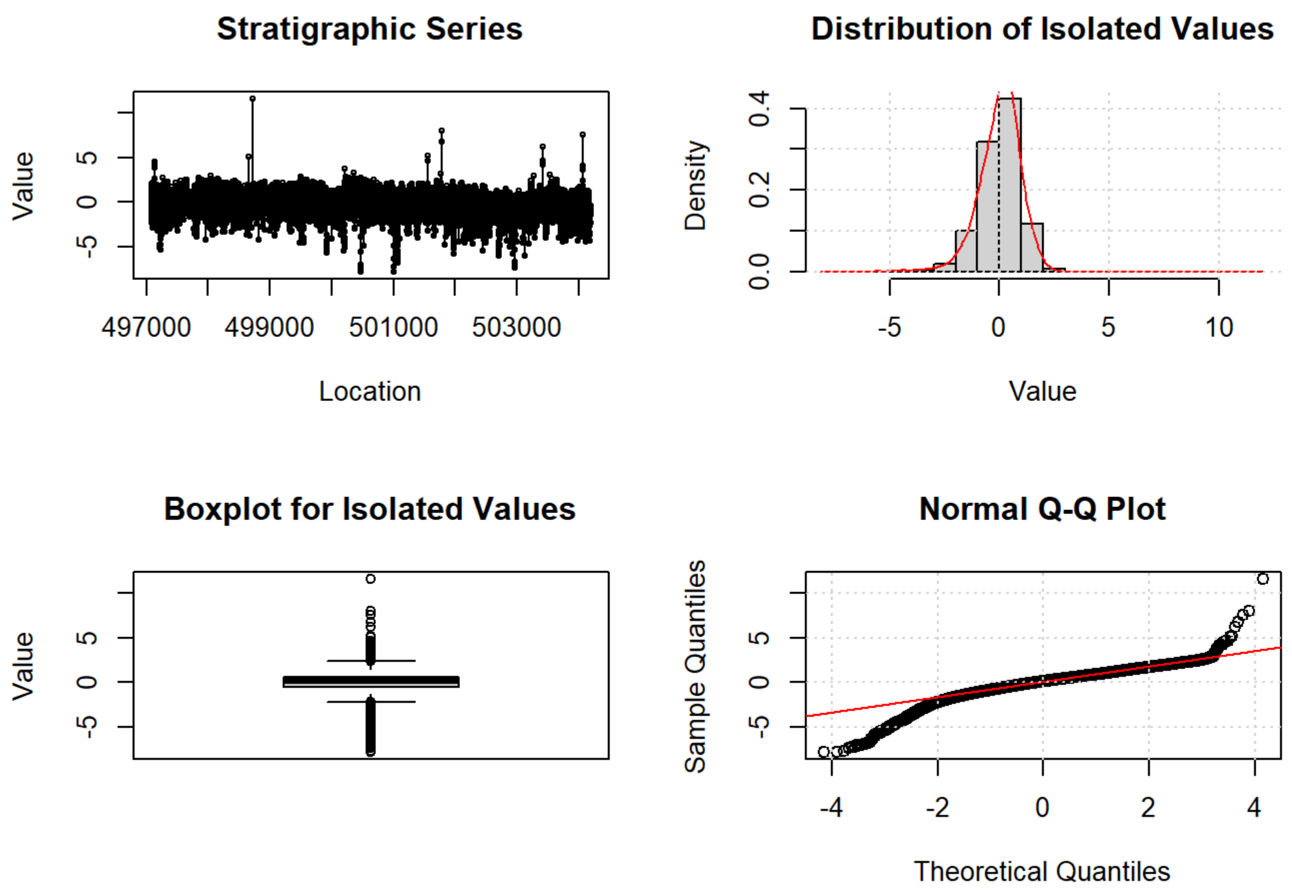


```
MIA <- linterp(MIA,dt=5, genplot=T)
```

```
----- APPLYING PIECEWISE-LINEAR INTERPOLATION TO STRATIGRAPHIC SERIES -----

 * Number of samples= 30577
 * New number of samples= 1423
```

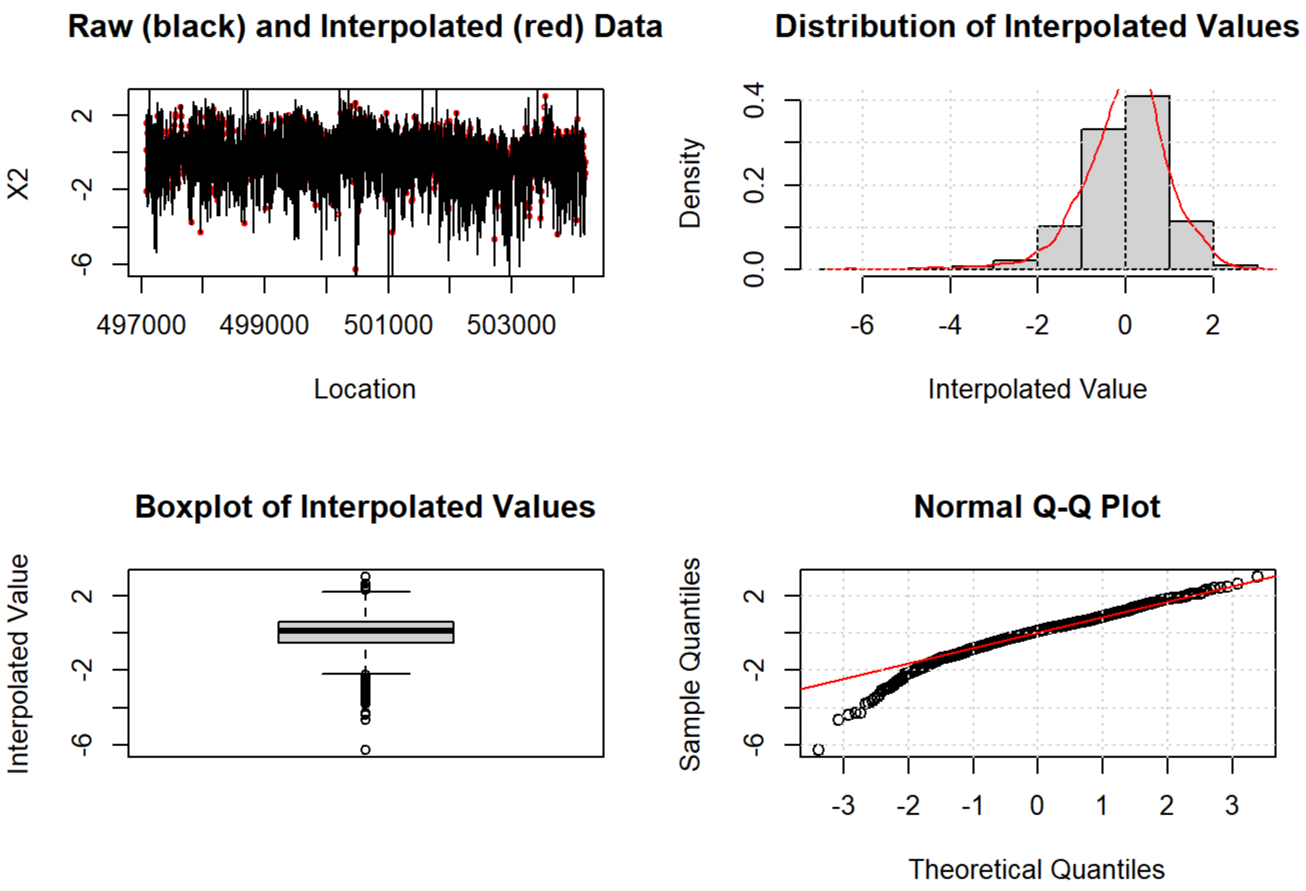


```
MIA_Ti_tuned <- linterp(MIA, genplot=T)
```

```
----- APPLYING PIECEWISE-LINEAR INTERPOLATION TO STRATIGRAPHIC SERIES -----

 * Number of samples= 1423
 * Determining median sampling interval for series
 * Will interpolate to median sampling interval of 5
 * New number of samples= 1423
```

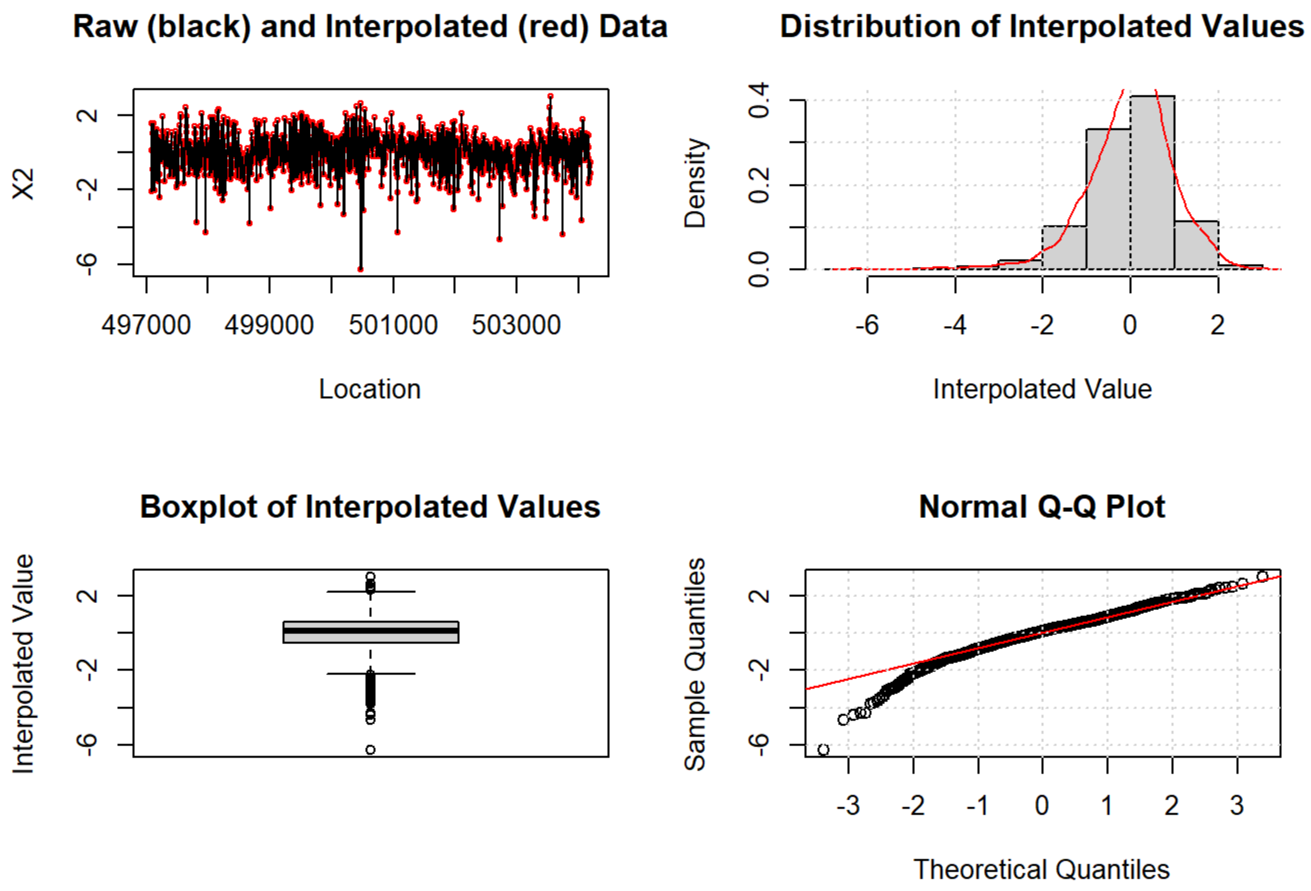


## 14.2. Lag-1 autocorrelation

Lag-1 window set from 20 to 90 kyr to avoid the 5-20 kyr high frequency noise and limit to 90 kyr that correspond to the lower boundary of the short eccentricity band

```
win_min <- 20
win_max <- 90
n_sim <- 100
dat <- data.frame(MIA_Ti_tuned)
dt2 <- dat[2, 1] - dat[1, 1]
dat <- dat[order(dat[, 1], na.last = NA, decreasing = F), ]
npts <- length(dat[, 1])
start <- dat[1, 1]
end <- dat[length(dat[, 1]), 1]
x1 <- dat[1:(npts - 1), 1]
x2 <- dat[2:(npts), 1]
dx = x2 - x1
dt = mean(dx)
sdt = sd(dx)
xout <- seq(start, end, by = dt)
npts <- length(xout)
interp <- approx(dat[, 1], dat[, 2], xout, method = "linear", n = npts)
d <- as.data.frame(interp)
mat_sim <- matrix(data = NA,
                  nrow = nrow(d),
                  ncol = n_sim)
```

```r
xout_vals <- d[, 1]
i <- 1
npts <- length(xout_vals)
new_sampling_rate <- NULL

fit <- for(i in 1:n_sim){

  if (sdt == 0) {
    new_sampling_rate <- runif(n=1, min = dt, max = win_min)}
  else if ((dt - (2 * sdt)) > 0) {
    new_sampling_rate <- truncnorm::rtruncnorm(
      n = 1,
      a = dt - 2 * sdt,
      b = dt + (2 * sdt),
      mean = dt,
      sd = sdt
    )}else {
      new_sampling_rate <- truncnorm::rtruncnorm(
        n = 1,
        a = dt / 2,
        b = dt + (2 * sdt),
        mean = dt,
        sd = sdt
      )
    }




  win_size <- stats::runif(n = 1,
                           min = min(c(win_min, win_max)),
                           max = max(c(win_min, win_max)))


  dt_new <- new_sampling_rate

  xout_new <- seq(from = start, to = end, by = dt_new)

  npts_new <- length(xout_new)

  interp_new <- approx(dat[, 1],
                       dat[, 2],
                       xout_new,
                       method = "linear",
                       n = npts
                       )

  d_new <- as.data.frame(interp_new)
  d_new[, 3] <- NA
  for (k in 1:nrow(d_new)) {
    row_nr_1 <- DescTools::Closest(d_new[, 1],
                                   d_new[k, 1] - (win_size / 2),
```

```
                                    which = TRUE)
    row_nr_2 <- DescTools::Closest(d_new[, 1],
                                   d_new[k, 1] + (win_size / 2),
                                   which = TRUE)
    data_sel <- d_new[row_nr_1[1]:row_nr_2[1], ]
    corr <- acf(data_sel[, 2], plot = F)
    a <- as.numeric(unlist(corr[1])[1])
    d_new[k, 3] <- a
  }
  yleft_comp <- d_new[1, 3]
  yright_com <- d_new[nrow(d_new), 3]
  app <- approx(
    d_new[, 1],
    d_new[, 3],
    xout_vals,
    method = "linear",
    n = npts,
    yleft = yleft_comp,
    yright = yright_com
  )
  app_res <- cbind(app$y)
  app_res_norm <- (app_res - min(app_res, na.rm = TRUE)) /
    (max(app_res, na.rm = TRUE) - min(app_res, na.rm = TRUE))
  mat_sim[,i] <- app_res_norm
}

mat_sim_mean <- rowMeans(mat_sim)
mat_sim_sd <- rowSds(mat_sim)
results <- cbind(xout_vals, mat_sim_mean, mat_sim_sd)

lag_1 <- results
```

```
plot(lag_1,
     type = "l",
     main = "Lag 1 over time",
     xlab = "Age Before Present (kyr)",
     ylab = "Lag 1 power"
     )
```

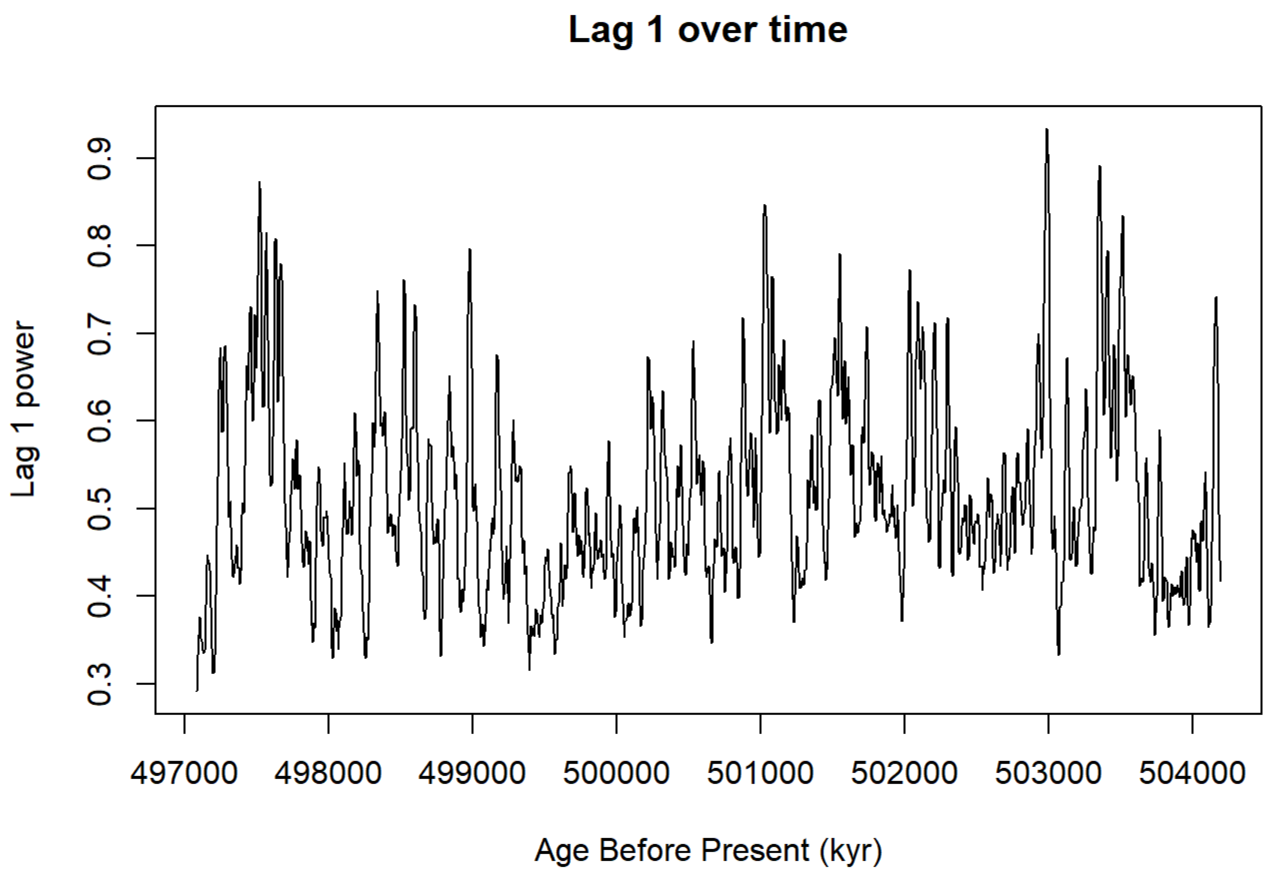


```
#    write.csv(lag_1,"lag1-rsp5kyr-window20-90kyr.csv")
```

## 14.3. Filtering of the Milankovitch cycles and comparison with lag-1 results

```
MIA_Ti__405 <- taner(MIA_Ti_tuned[,c(1,2)],flow=1/350,fhigh=1/450,roll=10^20,xmax=1/50)
```

```
----- TANER BANDPASS FILTERING STRATIGRAPHIC SERIES-----
 * Number of data points= 1423
 * Sample interval= 5
 * Mean value removed= -0.00624567
```

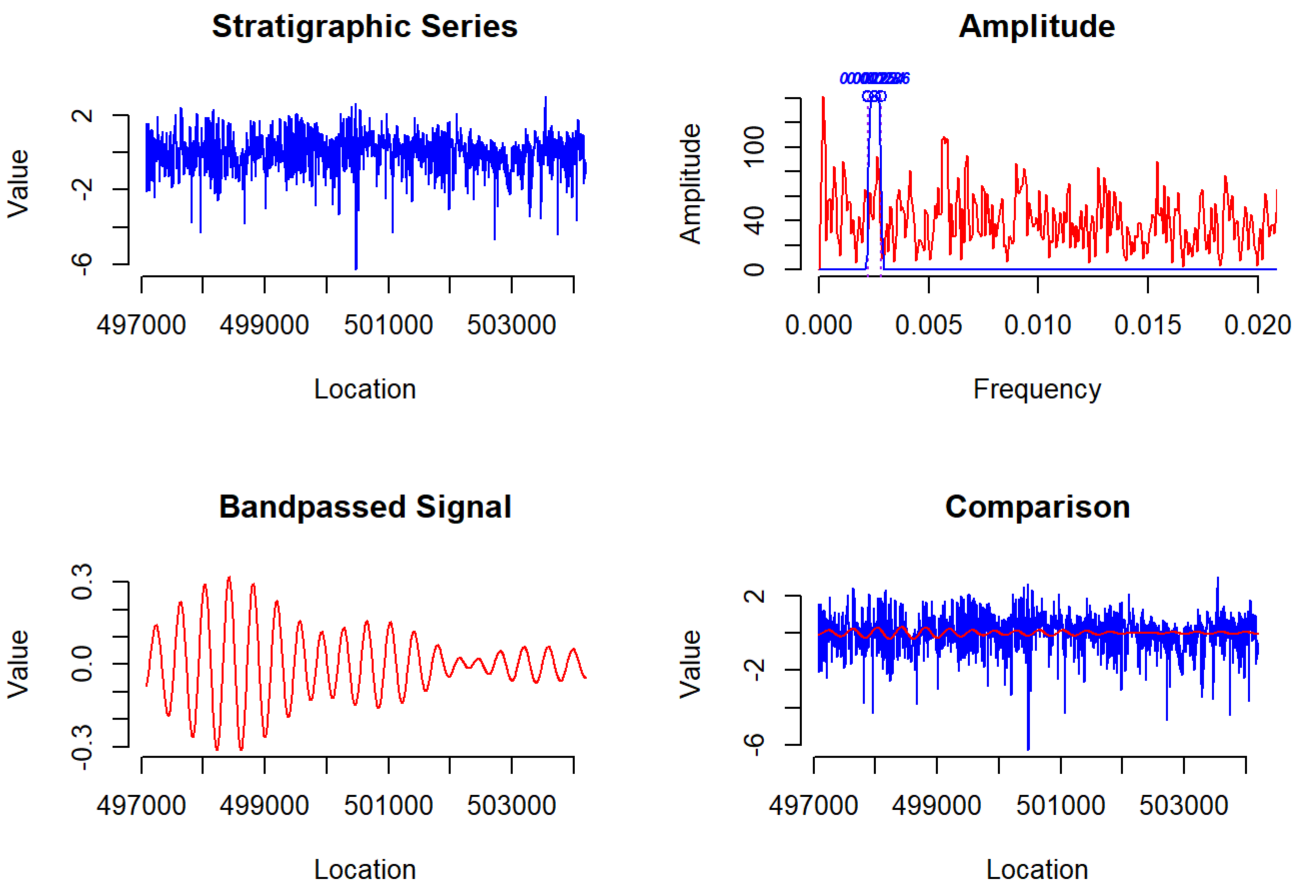


```
MIA_Ti__173<- taner(MIA_Ti_tuned[,c(1,2)],flow=1/150,fhigh=1/193,roll=10^20,xmax=1/50)
```

```
----- TANER BANDPASS FILTERING STRATIGRAPHIC SERIES-----
 * Number of data points= 1423
 * Sample interval= 5
 * Mean value removed= -0.00624567
```

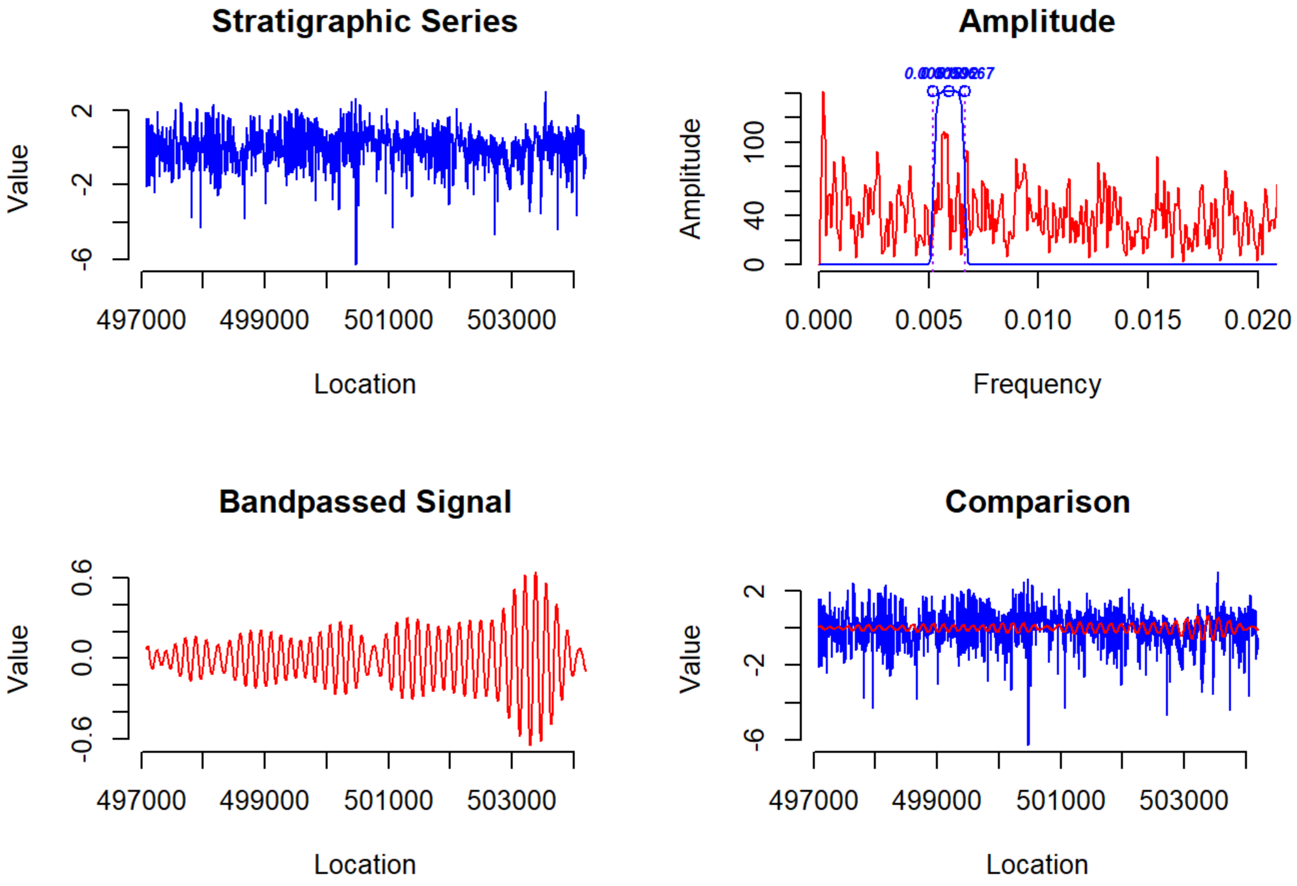


```
MIA_Ti__100<- taner(MIA_Ti_tuned[,c(1,2)],flow=1/140,fhigh=1/85,roll=10^20,xmax=1/50)
```

```
----- TANER BANDPASS FILTERING STRATIGRAPHIC SERIES-----
 * Number of data points= 1423
 * Sample interval= 5
 * Mean value removed= -0.00624567
```

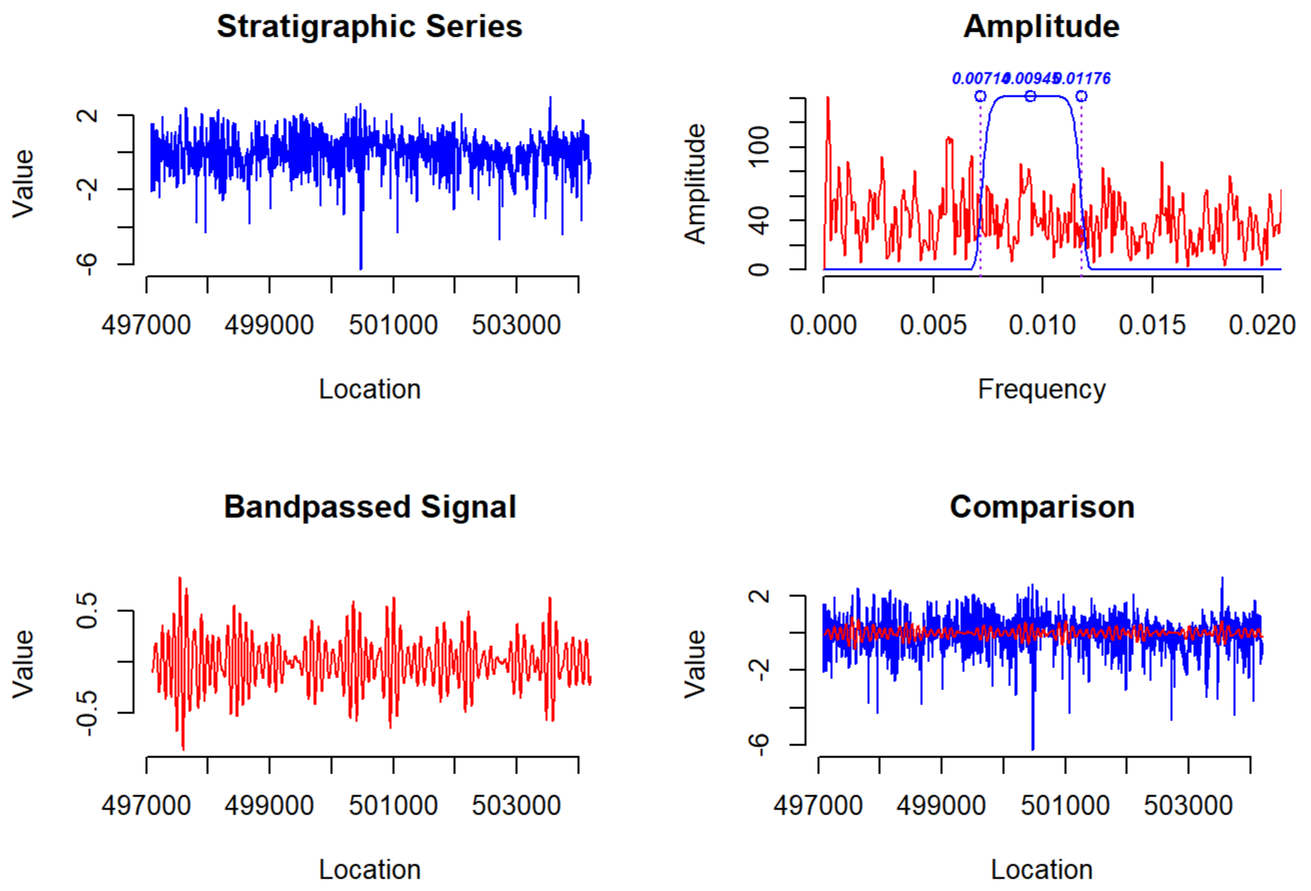


```
MIA_Ti__31 <- taner(MIA_Ti_tuned[,c(1,2)],flow=1/27,fhigh=1/40,roll=10^20,xmax=1/50)
```

```
----- TANER BANDPASS FILTERING STRATIGRAPHIC SERIES-----
 * Number of data points= 1423
 * Sample interval= 5
 * Mean value removed= -0.00624567
```

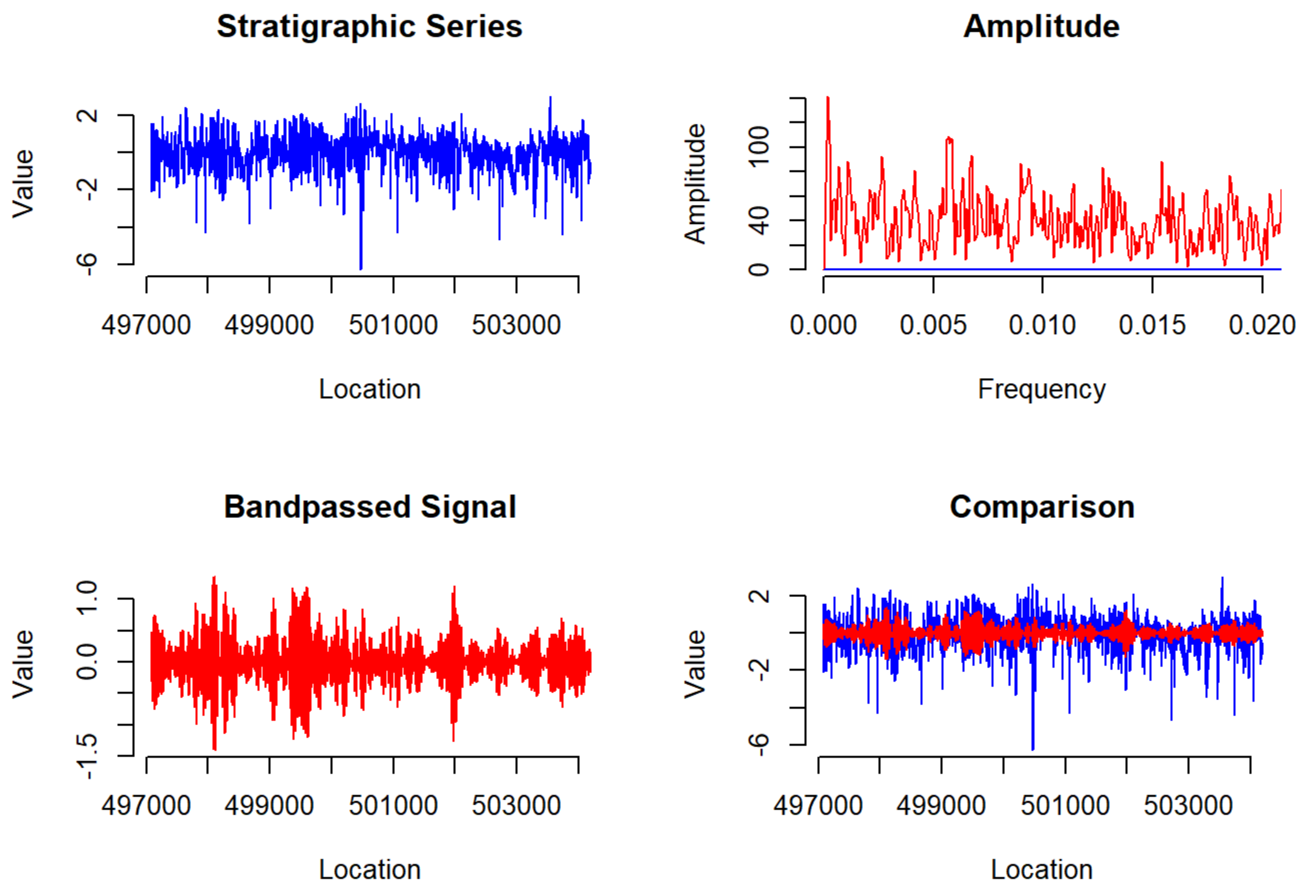


```
lag_1_background<- taner(lag_1[,c(1,2)],flow=1/500,fhigh=0,roll=10^20,xmax=1/50)
```

```
----- TANER BANDPASS FILTERING STRATIGRAPHIC SERIES-----
 * Number of data points= 1423
 * Sample interval= 5
 * Mean value removed= 0.5150584
```

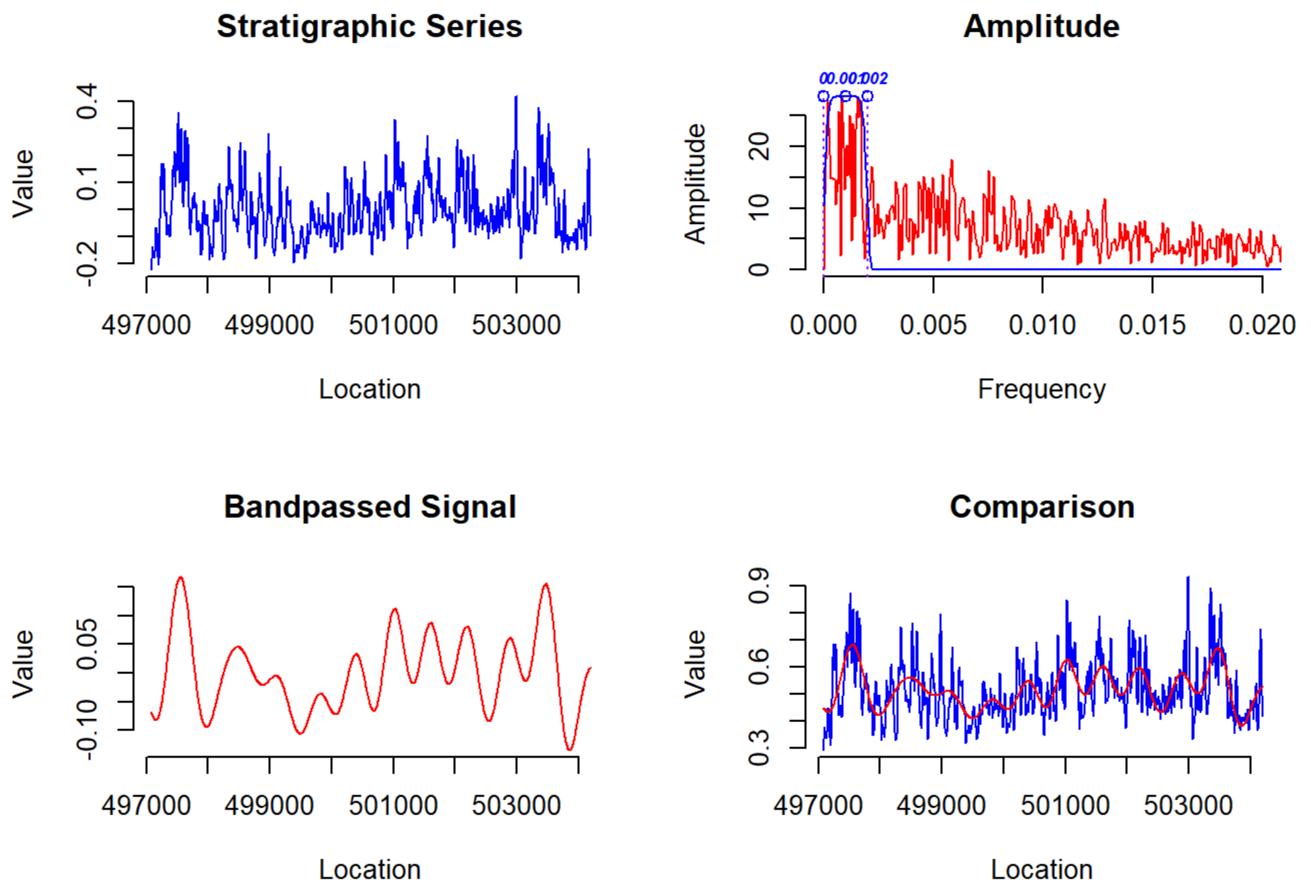


```
MIA_Ti__405_hilbert <- hilbert(MIA_Ti__405)
```

```
----- PERFORMING HILBERT TRANSFORM ON STRATIGRAPHIC SERIES -----
 * Number of data points= 1423
 * Sample interval= 5
 * Mean value removed= -0.005499469
```

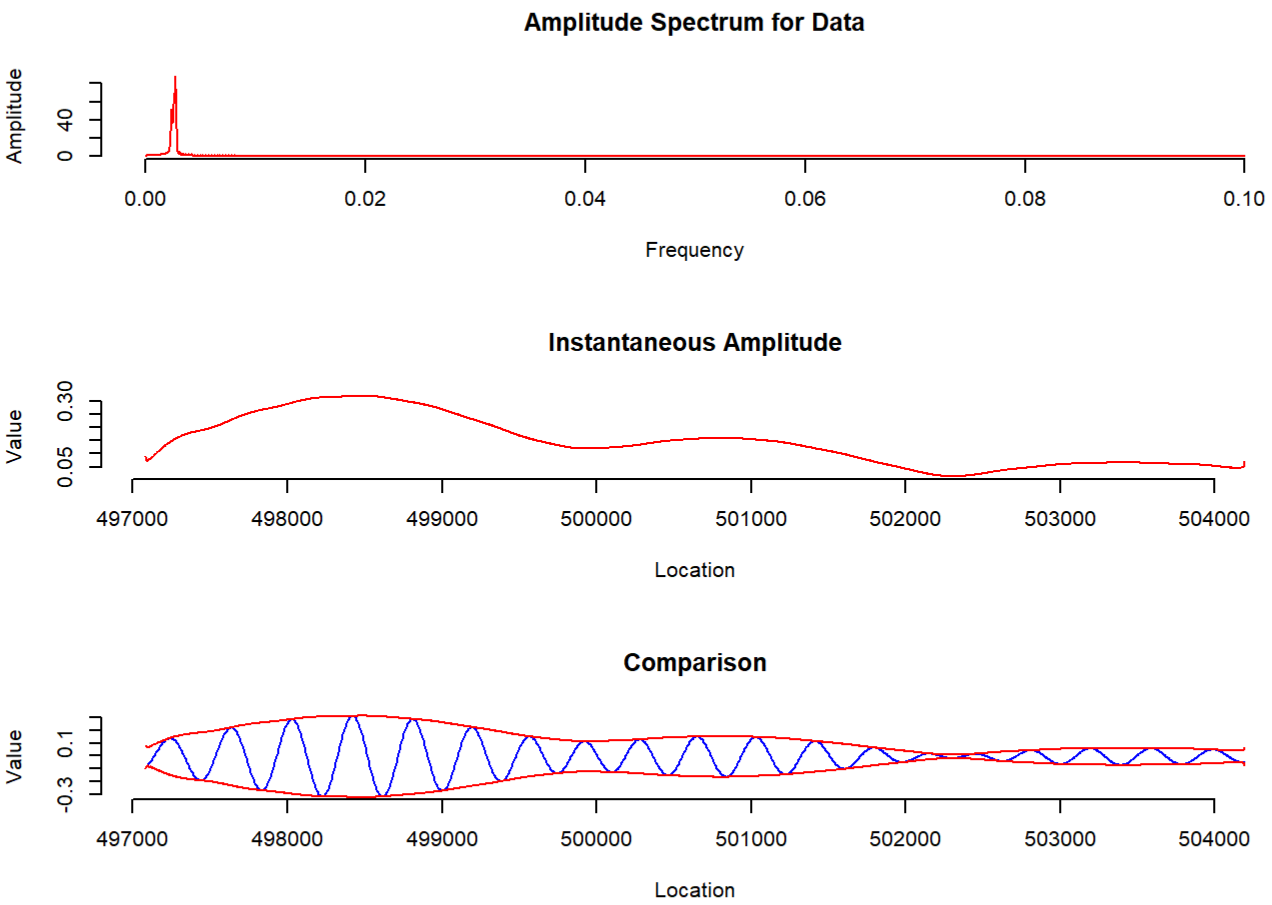


```r
MIA_Ti__173_hilbert <- hilbert(MIA_Ti__173)
```

```
----- PERFORMING HILBERT TRANSFORM ON STRATIGRAPHIC SERIES -----
 * Number of data points= 1423
 * Sample interval= 5
 * Mean value removed= -0.005971174
```

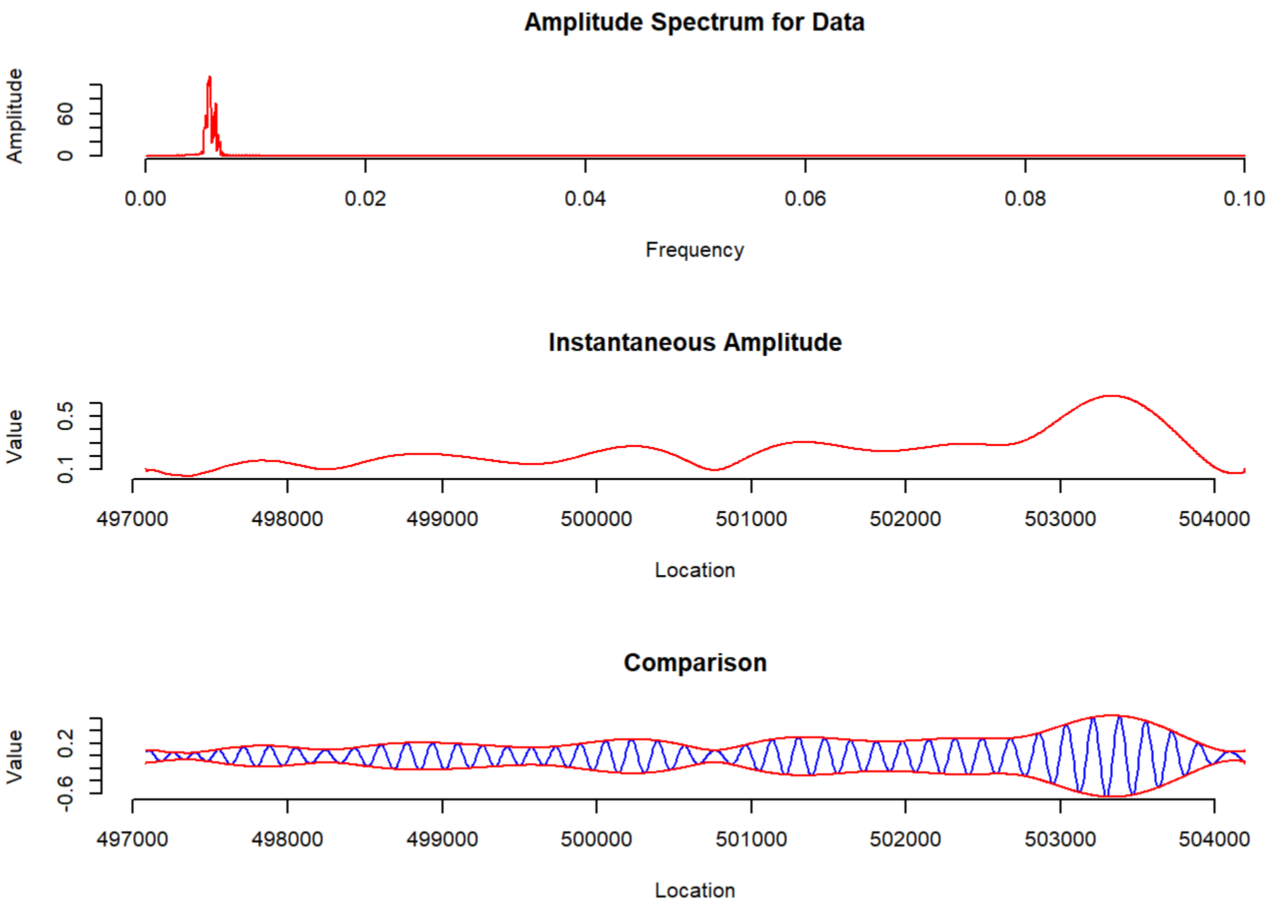


```
MIA_Ti__100_hilbert <- hilbert(MIA_Ti__100)
```

```
----- PERFORMING HILBERT TRANSFORM ON STRATIGRAPHIC SERIES -----
 * Number of data points= 1423
 * Sample interval= 5
 * Mean value removed= -0.006772776
```

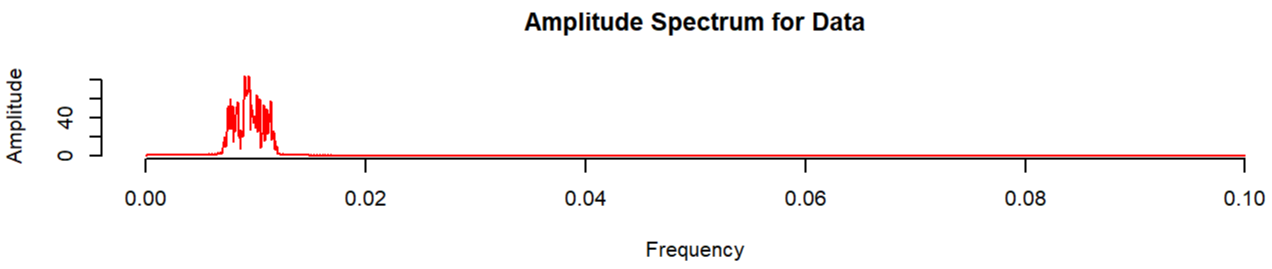


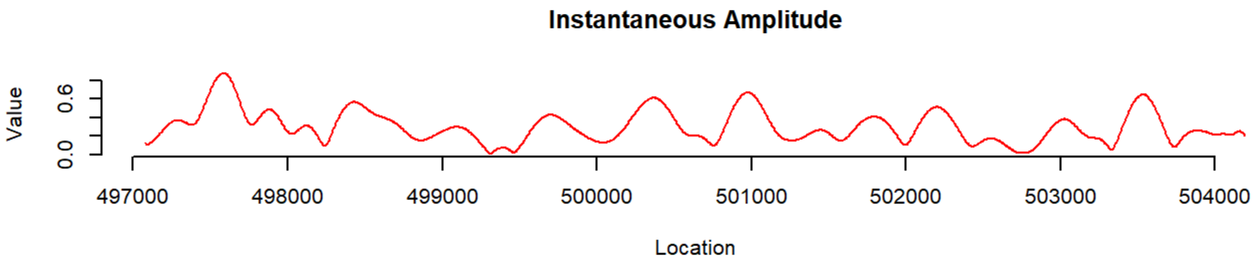


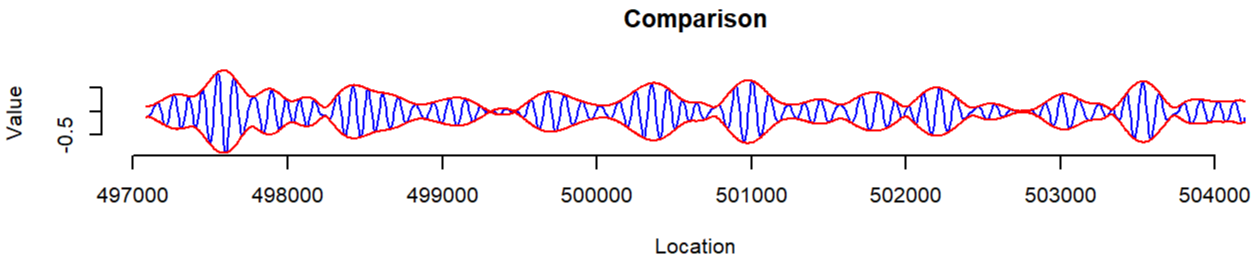


```
MIA_Ti__31_hilbert <- hilbert(MIA_Ti__31)
```

```
----- PERFORMING HILBERT TRANSFORM ON STRATIGRAPHIC SERIES -----
 * Number of data points= 1423
 * Sample interval= 5
 * Mean value removed= -0.006150106
```

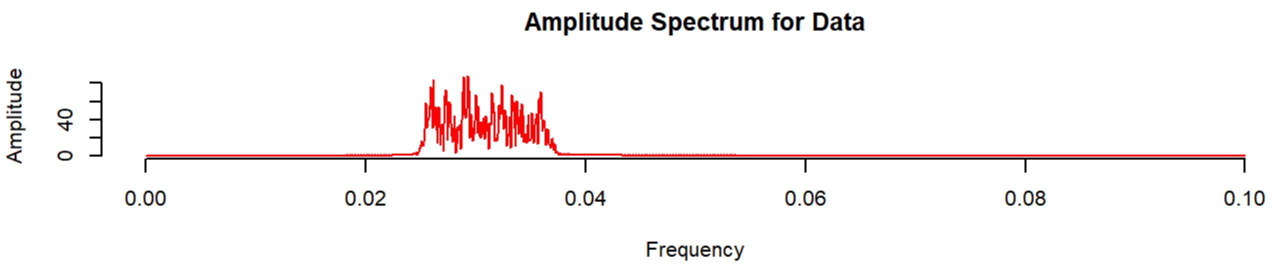


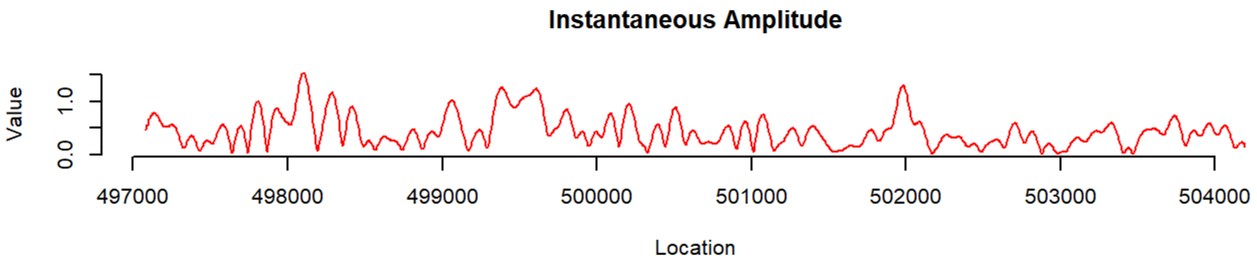


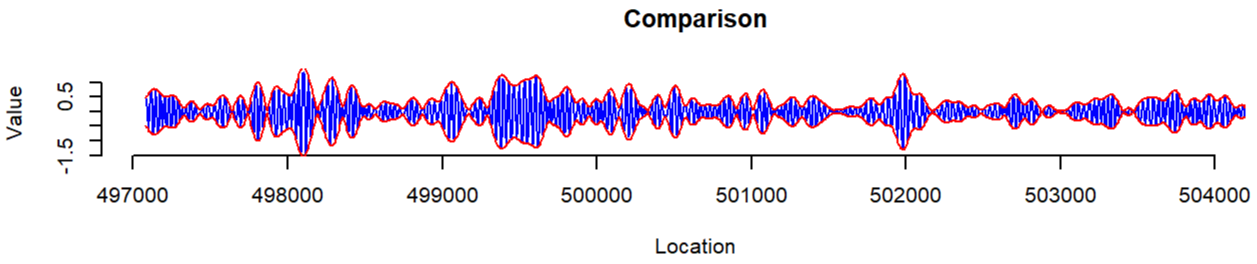


```
  #Creation of a matrix to display the lag 1 results and the Milankovitch filters
  #in one single figure
    layout.matrix <- matrix(c(1,2,3,4,5), nrow = 5, ncol = 1)
    graphics::layout(mat = layout.matrix,
heights = c(1), widths = c(1))

  #Lag 1 results
    par(mar = c(0,3,2,1))

    plot(lag_1[,c(1,2)],
         type="l",
         ylab="",
         xlab = "",
         ylim=c(0.3,0.9),
         bty = "n",
         yaxt = "n",
         xaxt = "n"
         )

    axis(2, seq(from = 0.3, to = 0.9, by = 0.2), cex.axis = 0.5, las = 1)

    title(ylab="Lag 1 power", line=2.2, cex.lab=0.75)

    title(main ="Lag 1 and sea level trend", line=0, cex.main=0.75)

    lines(lag_1_background[,1],(lag_1_background[,2]),col="blue", lwd=1.5)
```

```
# 405 kyr cycle and envelope
par(mar = c(0,3,0,1))

plot(MIA_Ti__405_hilbert[,1],
     (MIA_Ti__405_hilbert[,2]),
     type = "l",
     col="red",
     lwd=1.5,
     ylim = c(-0.6,0.6),
     xlab = "",
     ylab = "",
     xaxt ="n",
     yaxt = "n",
     bty = "n"
     )

lines(MIA_Ti__405[,1],(MIA_Ti__405[,2]),col="grey", lwd=0.5)

axis(2, seq(from = -0.3, to = 0.3, by = 0.3), cex.axis = 0.5, las=1)

title(ylab="filter output", line=2.2, cex.lab=0.75)

title(main ="405 kyr cycle and envelope", line=-2.5, cex.main=0.75)

#173 kyr cycle and envelope
 par(mar = c(0,3,0,1))

 plot(MIA_Ti__173_hilbert[,1],
      (MIA_Ti__173_hilbert[,2]),
      col="purple",
      lwd=1.5,
      type = "l",
      ylab = "",
      xlab = "",
      yaxt ="n",
      xaxt = "n",
      bty = "n",
      ylim = c(-0.6,0.7)
      )

lines(MIA_Ti__173[,1],
      (MIA_Ti__173[,2]-mean(MIA_Ti__173[,2])),
      col="grey",
      lwd=0.5
      )

axis(2, seq(from = -0.6, to = 0.6, by = 0.3), cex.axis = 0.5, las=1)

title(ylab="filter output", line=2.2, cex.lab=0.75)

title(main ="173 kyr cycle and envelope", line=-2, cex.main=0.75)

#100 kyr cycle and envelope
```

```
 par(mar = c(0,3,0,1))

plot(MIA_Ti__100_hilbert[,1],
     (MIA_Ti__100_hilbert[,2]),
     col="green",
     lwd=1.5,
     type = "l",
     ylim = c(-1.1,1.1),
     bty = "n",
     ylab ="",
     xlab = "",
     yaxt = "n",
     xaxt = "n"
     )

lines(MIA_Ti__100[,1],(MIA_Ti__100[,2]),col="grey", lwd=0.5)

axis(2, seq(from = -0.9, to = 0.9, by = 0.45), cex.axis = 0.5, las=1)

title(ylab="filter output", line=2.2, cex.lab=0.75)

title(main ="100 kyr cycle and envelope", line=-1, cex.main=0.75)

#31 kyr cycle and envelope
 par(mar = c(3,3,0,1))

plot(MIA_Ti__31_hilbert[,1],
     (MIA_Ti__31_hilbert[,2]),
     col="orange",
     lwd=1.5,
     type = "l",
     ylim = c(-1.6,1.6),
     bty = "n",
     ylab ="",
     xlab ="",
     yaxt = "n",
     xaxt = "n"
     )

lines(MIA_Ti__31[,1],(MIA_Ti__31[,2]),col="grey", lwd=0.5)

axis(2, seq(from = -1.5, to = 1.5, by = 0.75), cex.axis = 0.5, las=1)

title(ylab="filter output", line=2.2, cex.lab=0.75)

axis(1, seq(from = 497000, to = 504000, by = 1000), cex.axis = 0.5)

title(xlab="Age Before Present (kyr)", line=2, cex.lab=0.75)

title(main ="31 kyr cycle and envelope", line=-0.5, cex.main=0.75)
```

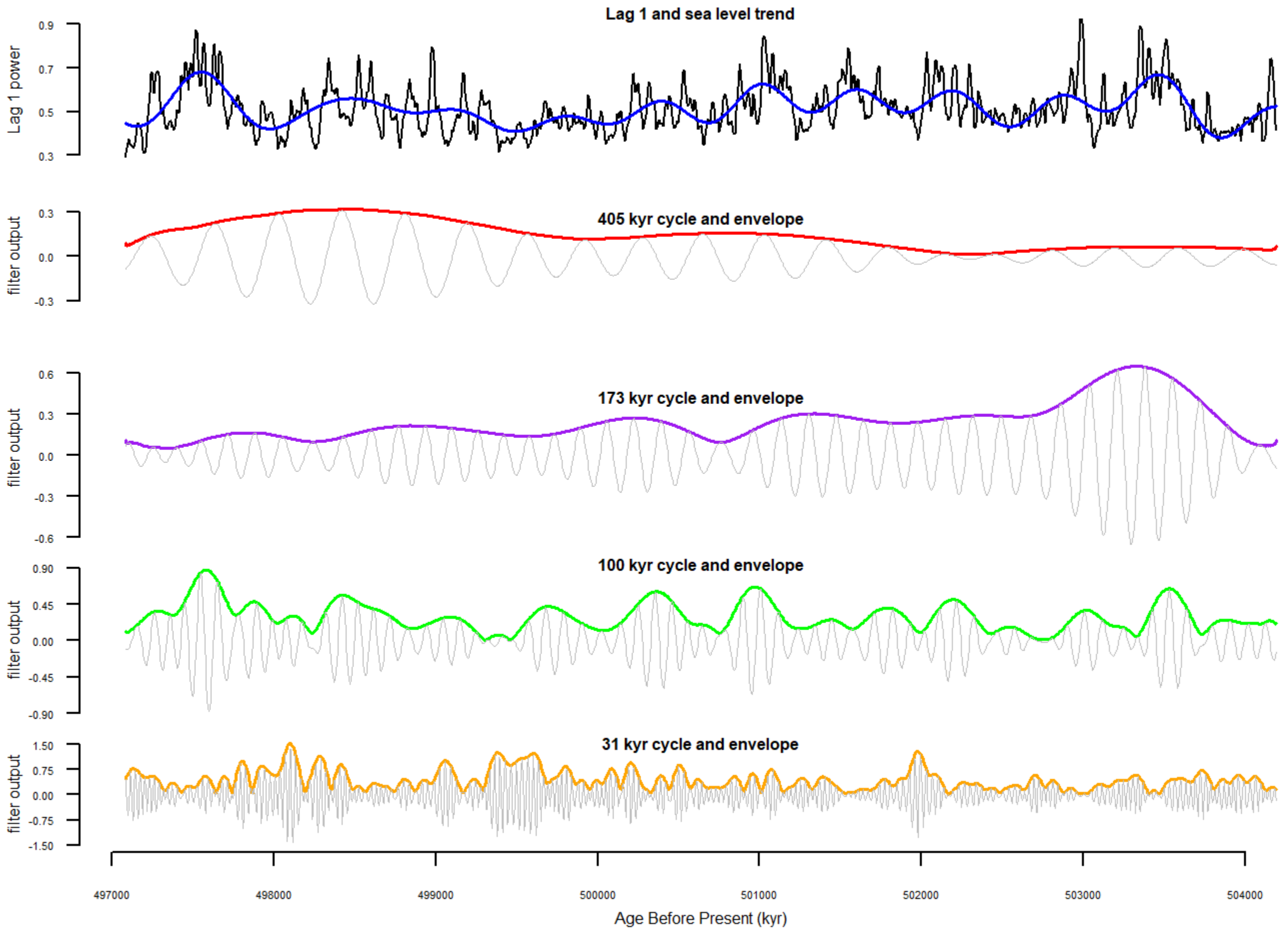


```
    #Saving the lag-1 results and Milankovitch filters
#    write.csv(lag_1_background,"Lag1_20-90window_background_0-1over500kyr.csv")
#    write.csv(MIA_Ti__405_hilbert,"MIA_TI_rsp_5kyr_405kyr_hilbert.csv")
#    write.csv(MIA_Ti__173_hilbert,"MIA_TI_rsp_5kyr_173kyr_hilbert.csv")
#    write.csv(MIA_Ti__100_hilbert,"MIA_TI_rsp_5kyr_100kyr_hilbert.csv")
#    write.csv(MIA_Ti__31_hilbert,"MIA_TI_rsp_5kyr_31kyr_hilbert.csv")
```
